# Nucleophilicity of 4‐(Alkylthio)‐3‐imidazoline Derived Enamines

**DOI:** 10.1002/chem.202302764

**Published:** 2023-11-15

**Authors:** Magenta J. Hensinger, Andreas Eitzinger, Oliver Trapp, Armin R. Ofial

**Affiliations:** ^1^ Department Chemie Ludwig-Maximilians-Universtität München Butenandtstrasse 5–13 81377 München Germany; ^2^ Max-Planck-Institute for Astronomy Königstuhl 17 69117 Heidelberg Germany

**Keywords:** enamines, kinetics, linear free energy relationships, quantum-chemical calculations, thermodynamics

## Abstract

Imidazolidine‐4‐thiones (ITOs) are cyclic, secondary amines that were considered as potential prebiotic organocatalysts for light‐driven α‐alkylations of aldehydes by bromoacetonitrile (BAN). Recent studies showed that the initially supplied ITOs represent the pre‐catalyst because they undergo *S*‐alkylation with BAN to give 4‐(alkylthio)‐3‐imidazolines (TIMs). Given that the same reagent mix that undergoes light‐driven α‐alkylations is also effective in the dark, we synthesized ten ITO‐ or TIM‐derived enamines of aldehydes and characterized their nucleophilic reactivities by kinetic studies in acetonitrile. The experimental second‐order rate constants *k*_2_ for reactions of enamines with benzhydrylium ions (reference electrophiles) were evaluated by the Mayr‐Patz equation, lg *k*_2_(20 °C)=*s*_N_(*N*+*E*). The determined nucleophilicities *N* (and *s*_N_) reveal the reactivity profiles of these enamines under prebiotically relevant conditions as well as their potential for use in organocatalytic synthesis.

## Graphical abstract

**Enamine activation** is a key method to enhance the reactivity of carbonyl compounds in α‐alkylation reactions. In spite of their possible role in prebiotic chemistry, enamines derived from aldehydes and 4‐(alkylthio)‐3‐imidazolines (TIMs) have scarcely been studied. Their reactivities have now been classified on the Mayr nucleophilicity scale by kinetic measurements. In addition, their Lewis basicities have been determined by DFT calculations and equilibrium measurements.

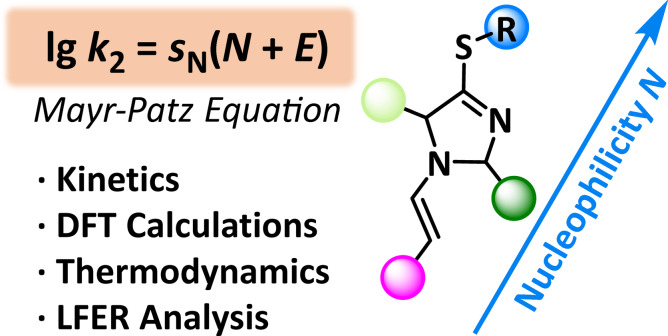

## Introduction

Enamine activation has become an established and widely used concept in modern organocatalysis. In particular, the use of chiral cyclic secondary amines plays a key role in the development of efficient asymmetric derivatizations of carbonyl compounds.^[1]^

**Figure 1:**
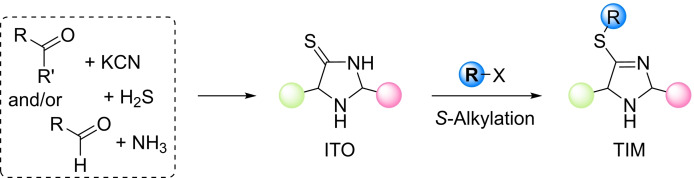
General structures of ITOs and TIMs and their generation from educts.

The Trapp group has recently shown that imidazolidine‐4‐thiones (ITOs, Figure 1)^[2]^ assemble reversibly under prebiotically plausible conditions from KCN, H_2_S, ammonia, and carbonyl compounds as the building blocks.[^3^, ^4^, ^5^] ITOs have only occasionally been used in synthetic organocatalytic reactions.^[6]^ However, the reversible formation of ITOs implies that they are capable of structural adaption to the environment.

Owing to the fact that ITO‐catalyzed reactions of first generation carbonyl compounds could generate a second generation of structurally modified carbonyl compounds,^[3]^ the dynamics of ITO formation would thus pave the way to next generation ITO catalysts. In total, this feedback of products on the catalyst structure resembles a simple evolutionary process and may have led to the non‐enzymatic formation of more complex carbonyl compounds on Early Earth.^[3]^

**Scheme 1:**
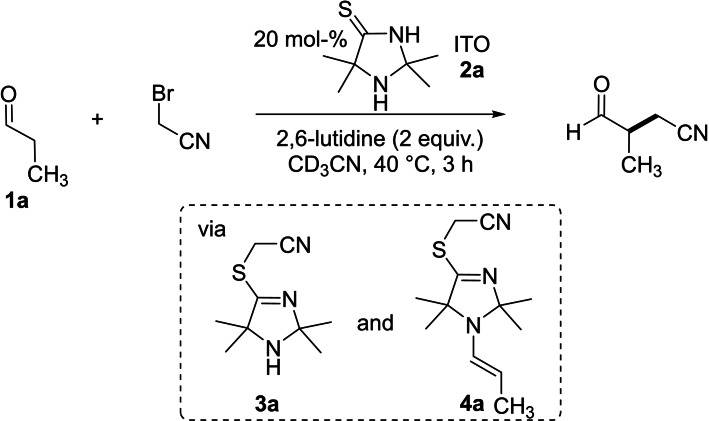
ITO‐promoted α‐alkylation of propanal in the dark via the intermediates TIM **3 a** and the TIM‐derived enamine **4 a** (detected by NMR spectroscopy, Ref. [7]).

Monitoring the ITO‐promoted[^3^, ^4^] α‐alkylation of propanal (**1 a**)^[5c]^ with bromoacetonitrile^[5e]^ in acetonitrile^[5d]^ by in situ NMR spectroscopy provided insight on possible intermediates formed during the organocatalytic cycle (Scheme 1).[^4^, ^7^, ^8^] We detected that ITO **2 a** was first *S*‐alkylated[^9^, ^10^] to give a 4‐(alkylthio)‐3‐imidazoline (TIM) **3 a**, which in a second step condensed with **1 a** to yield the enamine intermediate **4 a**. Finally, the TIM‐derived enamine **4 a** underwent a nucleophilic substitution with a second equivalent of bromoacetonitrile which ultimately led to the formation of the α‐cyanomethylated product aldehyde.^[7]^

We sought to gain further insight on factors that influence the reactivity of the enamine intermediates,^[11]^ which are the crucial nucleophilic species in the carbon‐carbon bond‐forming step in Scheme 1. Hence, we prepared and spectroscopically characterized a series of ITO‐ and TIM‐derived enamines and determined their nucleophilicity by using Mayr's benzhydrylium methodology and the linear free energy relationship in Equation 1.^[12]^

(1)

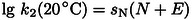




As suggested by Mayr and Patz,^[12a]^ Equation (1) is used to convert experimentally determined second‐order rate constants of electrophile‐nucleophile reactions in a given solvent at 20 °C, that is, *k*_2_(20 °C), to reactivity descriptors of the reaction partners. Following this concept, the Mayr‐Patz Equation (1) has already successfully been used to quantify the nucleophilicity parameters *N* (and *s*_N_) of various enamines derived from simple cyclic, secondary amines as well as from well‐established classes of cyclic, secondary amine organocatalysts.[^12f^, ^13^] Embedding ITO‐ and TIM‐derived enamines on the Mayr nucleophilicity scales will thus reveal the impact of structural changes in enamines **4**, define their scope in organic synthesis, and facilitate their comparison with enamines derived from more frequently used organocatalysts.

## Results and Discussion

### Preparation of enamines 4

**Figure 2:**
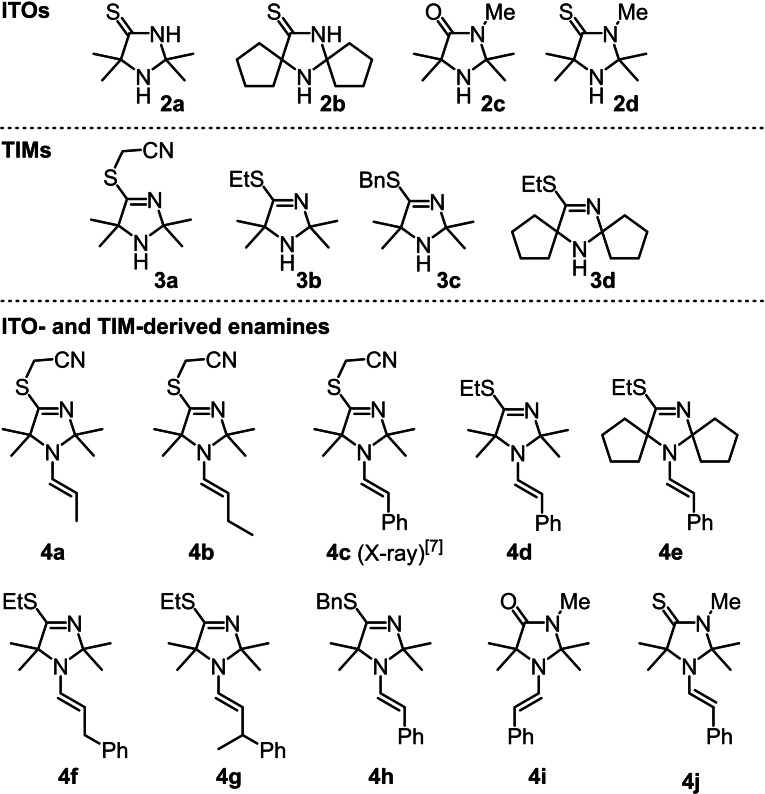
Structures of ITOs **2**, TIMs **3**, and ITO‐ and TIM‐derived enamines **4 a**–**4 j**.

Detection of the TIM **3 a** under the prebiotically plausible reaction conditions reported in ref.^[7]^ inspired us to study enamines **4 a**–**4 c**, which are derived from **3 a** and propanal, butanal, or phenylacetaldehyde, respectively (Figure 2). Further patterns of *S*‐alkylation comprised enamines with *S*‐ethyl (**4 d**–**4 g**) and *S*‐benzyl groups (**4 h**), which were generated by condensation of phenylacetaldehyde, 3‐phenylpropanal, or 3‐phenylbutanal with the *S*‐ethylated and *S*‐benzylated TIMs **3 b** and **3 c**, respectively. Usually, the 2,2,5,5‐tetramethyl‐substitution pattern at the TIM moiety in **4** was kept, except for the spirocyclic bis‐tetramethylene substituted enamine **4 e**. Finally, enamine **4 i** resembles a 4‐imidazolidinone analogue of the ITO‐derived enamines of phenylacetaldehyde. In enamine **4 j**, the oxygen atom in **4 i** is exchanged for a sulfur atom and, thus, enables one to study the effect of *N*‐alkylation of the parent ITO **2 a** on the enamine reactivity.

Classical enamine syntheses and subsequent purification by column chromatography on basic or neutral aluminum oxide furnished pure samples of the phenylacetaldehyde‐derived enamines **4 c**,^[7]^ **4 d**, **4 e**, **4 h**, **4 i**, and **4 j**. The enamines **4 f** and **4 g** prepared in almost quantitative yields by condensation of *S*‐ethyl TIM **3 b** with 3‐phenylpropanal or 3‐phenylbutanal, respectively, were used without further purification because they decomposed on contact with silica gel or aluminum oxide during attempts of chromatographic purifications. With the low molecular weight aldehydes propanal and butanal, solutions of the enamines **4 a** and **4 b** were prepared under irradiation with light (LED with 420 nm emission) from mixtures of TIM **3 a**, the respective aldehydes, 2,6‐lutidine and 4 Å molecular sieves in acetonitrile. At the end of the reactions, volatiles with high vapor pressure (mainly the aldehydes) were removed in the vacuum. The resulting mixtures of enamines, 2,6‐lutidine, and TIM in acetonitrile were stable when stored under a dry argon atmosphere (glovebox). The enamine content in the individual samples was determined by ^1^H NMR spectroscopy (in CD_3_CN) shortly before using them for further preparative or kinetic investigations.

As indicated by the uniform coupling constants of the olefinic protons (^3^ *J*=14.4 to 14.9 Hz), the carbon‐carbon double bonds of all enamine moieties in **4 a**–**4 j** are in (*E*)‐configuration. The *s‐cis* or *s‐trans* orientation of the N−C(α) bond of the enamines was identified as a key factor for controlling the enantioselectivity in radical additions to imidazolidinone‐derived enamines.^[14]^ In the single‐crystal X‐ray structure of enamine **4 c** an *s‐cis* conformation at the N−C(α) bond was observed.^[7]^ In solution, the NOESY spectrum of **4 h** in CD_3_CN indicated free rotation around the N−C(α) bond. The NOESY spectrum of the imidazolidinone‐derived enamine **4 i** suggested that it slightly prefers an *s‐trans* conformation in CDCl_3_ solution.^[15]^ Given that the positions 2 and 5 at the heterocycles of the enamines **4 a**–**4 j** are disubstituted with identical alkyl groups, almost equal Boltzmann distributions between *s*‐*cis*‐ and *s*‐*trans* conformers and fast equilibration of both forms can be expected. Thus, we considered the orientation at the N−C(α) bond of the enamines to be insignificant for the subsequent reactivity studies and depicted the molecular structures of enamines **4** in an arbitrarily chosen orientation.

### Quantum‐chemical characteristics of enamines 4

**Figure 3:**
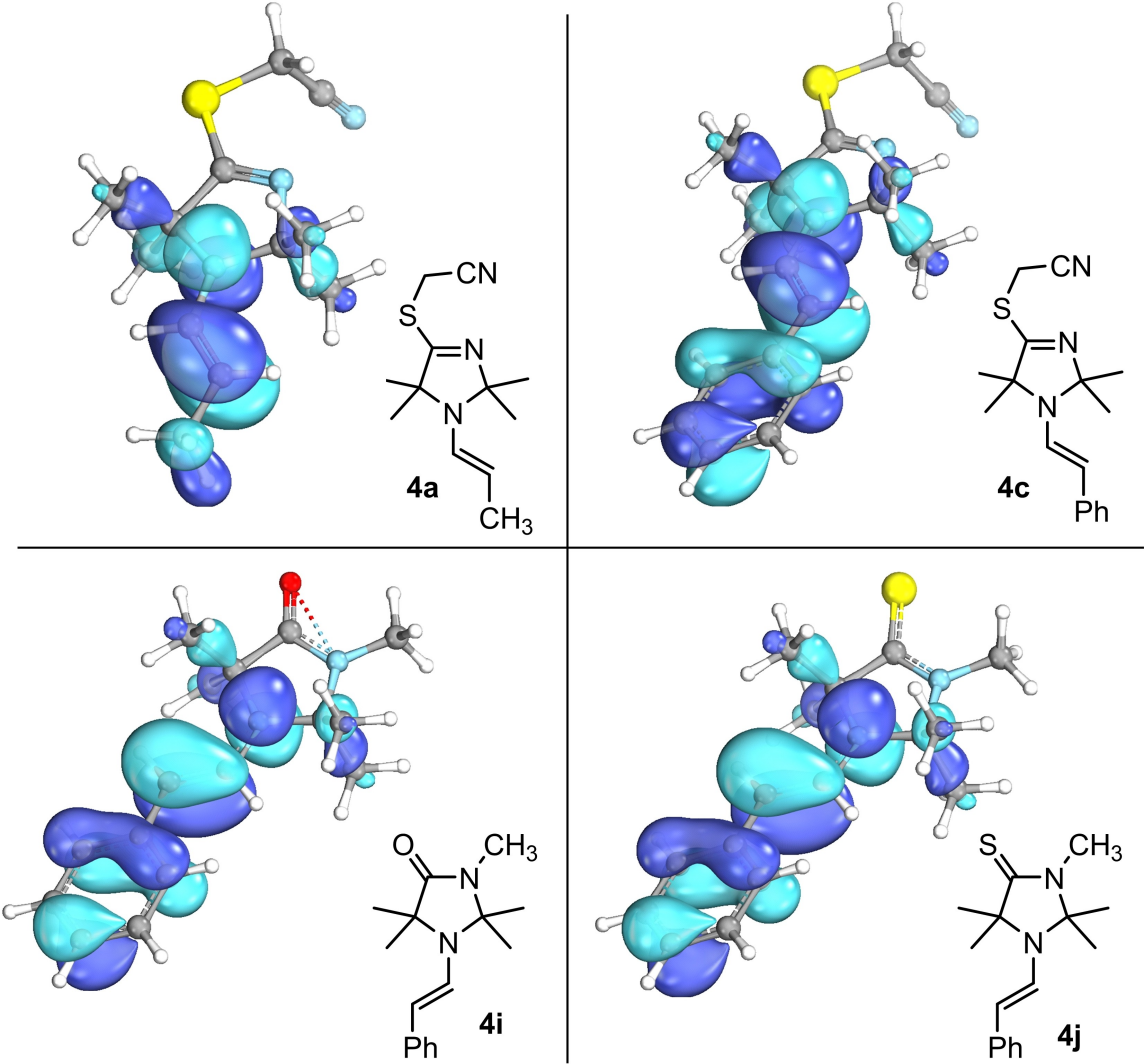
HOMOs of selected enamines **4** calculated on the SMD(acetonitrile)/PBE0‐D3/def2‐TZVP//PBE0‐D3/def2‐TZVP level of theory and visualized using IboView.^[16]^

Owing to the different structures of the cyclic amine moieties in the enamines **4**, we first analyzed their general electronic properties by quantum‐chemical calculations. HOMO energies of **4 a**, **4 c**, **4 i**, and **4 j** were calculated from single point calculations on the SMD(acetonitrile)/PBE0‐D3/def2‐TZVP//PBE0‐D3/def2‐TZVP level of theory.^[15]^ The visualization in Figure 3 illustrates^[16]^ that the HOMO of all types of enamines is centered around the enamine moieties in accord with the anticipated general reactivity as carbon‐centered nucleophiles.

**Figure 4:**
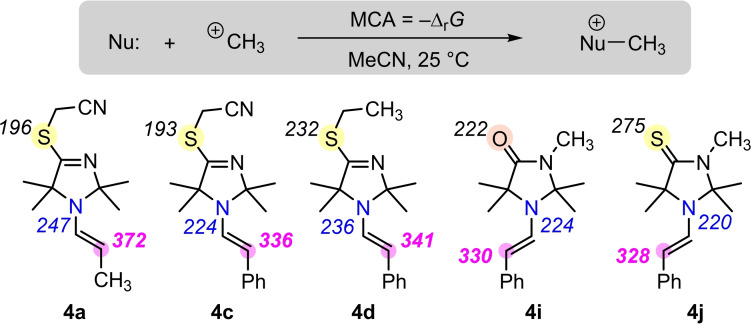
Methyl cation affinities (MCA) of enamines **4** (MCA values are given as Boltzmann‐weighted averages of relevant conformers in kJ mol^−1^, Ref. [15]).

Nevertheless, the highly functionalized enamines **4** feature more than one nucleophilic (or Lewis basic) center. We, therefore, investigated the thermodynamics of bond‐forming reactions at the nitrogen and the carbon of the enamine moiety as well as at the Lewis basic sulfur and oxygen atoms, which are attached to the 4‐position of the heterocycles. We used methyl cations as models for potential carbon‐centered electrophilic reaction partners of the nucleophiles **4** and calculated the Gibbs energies of the reactions (Δ_r_ *G*°) in acetonitrile (SMD model) by using DFT methods at the SMD(acetonitrile)/B3LYP/6‐311++G(3df,2pd)//B3LYP/6‐31G(d,p) level of theory^[15]^ that was previously used for pyrrolidine, piperidine‐ and morpholine‐derived enamines.[^13e^, ^17^] Methyl cation affinities (MCA) reported in Figure 4 reflect the negative value of Δ_r_ *G*°(25 °C).

The MCA values for **4 a**, **4 c**, **4 d**, **4 i**, and **4 j** clearly show that alkylation at the carbon atom of the enamine unit is thermodynamically favored over competing reactions at other Lewis basic sites of the same molecules. In particular, the steric demand introduced by the four methyl groups in direct vicinity to the enamine nitrogen makes *N*‐methylation of the enamine units by more than 105 kJ mol^−1^ less exothermic than *C*‐methylation. Sulfur or oxygen at the 4‐position of the heterocycle are also only weakly Lewis basic. Even in the thiolactam **4 j** methylation at the S‐atom is still delivering less thermodynamic driving force (MCA=275 kJ mol^−1^) than the analogous reaction at the enamine's carbon atom (328 kJ mol^−1^).

Next, we used Mayr's benzhydrylium methodology to calibrate the nucleophilicities of the ITO‐ and TIM‐derived enamines **4 a**–**4 j**.

### Reactions of enamines 4 with benzhydrylium salts 5

**Figure 5:**
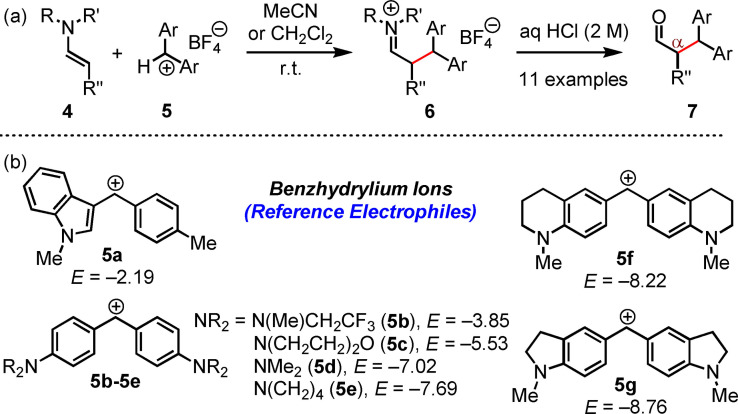
(a) Enamines **4** reacted with benzhydrylium tetrafluoroborates **5** to furnish iminium salts **6**, which were hydrolyzed to α‐alkylated aldehydes **7**. (b) Structures of benzhydrylium ions **5 a**–**5 g** (counterion: tetrafluoroborate) used in this study with their Mayr electrophilicity parameters *E* (from refs. [12b, 18, 19]).

**Product studies**. First, we studied the products of selected reactions of the enamines **4 a**–**4 j** with the benzhydrylium tetrafluoroborates **5 a**–**5 g** (Figure 5), which have been established by H. Mayr et al. as reference electrophiles with solvent‐independent and reliably determined electrophilicity parameters *E*.[^12b^, ^12e^]

In accord with previously studied enamine/benzhydrylium ion adduct formations,[^7^, ^13^] the reaction of the enamine **4 d** with **5 c** generated an iminium salt (as a mixture of (*E*)‐ and (*Z*)‐configured **6**) as detected by NMR spectroscopy in CD_3_CN.^[15]^ Upon aqueous workup, further combinations of enamines **4** with benzhydrylium tetrafluoroborates **5** furnished uniformly the corresponding α‐benzhydrylated aldehydes **7** in unoptimized yields of 19 to 79 %.^[15]^ In line with the results of the MCA calculations, exclusively products of *C*‐alkylation of **4** by **5** were observed for all investigated electrophile/nucleophile combinations.

**Kinetics**. According to Mayr's benzhydrylium methodology,^[12e]^ following the kinetics of the reactions of enamines **4** with a series of benzhydrylium salts **5** yields second‐order rate constants *k*_2_ for the carbon‐carbon bond‐forming reactions under defined conditions, that is, in a given solvent at a certain temperature. Substituting those rate constants of the enamine/benzhydrylium reactions as well as the electrophilicity parameters *E* of the benzhydrylium ions **5** in Equation (1) allows one to calculate the nucleophilicity parameters *N* (and *s*_N_) for the enamines **4**. By positioning them in Mayr's nucleophilicity scales, the reactivities of **4 a**–**4 j** become comparable with those of other enamines that are constructed from secondary amine organocatalysts and aldehydes.^[13]^

**Figure 6:**
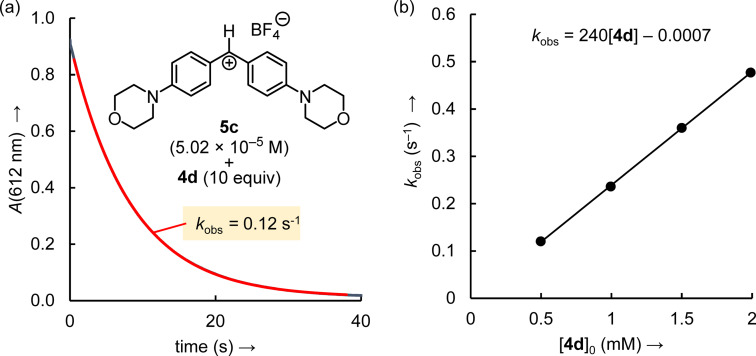
(a) Exponential decay of the absorbance *A* at 612 nm for the reaction of **4 d** (*c*_0_=4.98×10^−4^ M) with **5 c** (*c*_0_=5.02×10^−5^ M) in MeCN at 20 °C. (b) Determination of the second‐order rate constant *k*_2_ for the reaction of **4 d** with **5 c** from the slope of the linear correlation between *k*_obs_ and [**4 d**].

The kinetics of the reactions of **4 a**–**4 j** (≥10 equiv) with **5 a**–**5 g** (reference electrophiles) in acetonitrile at 20 °C were monitored photometrically by following the consumption of **5** (stopped‐flow method, 434 nm≤*λ*_max_≤674 nm). Under these conditions, the (pseudo)‐first‐order rate constants *k*_obs_ (s^−1^) were derived from a least squares fit of the function *A*_t_=*A*_0_ exp(−*k*_obs_ *t*)+*C* to the time‐dependent absorbances of the electrophiles (Figure 6a). The second‐order rate constants *k*_2_ (M^−1^ s^−1^) were obtained as the slopes of the linear correlations of *k*_obs_ with the concentration of the enamines (Figure 6b).

Table 1 lists the second‐order rate constants *k*_2_ for reactions in acetonitrile. Details of the individual kinetic measurements and analogous kinetic studies of reactions in dichloromethane are given in the Supporting Information.

**Table 1:** Second‐order rate constants *k*_2_ for the reactions of the enamines **4** with the benzhydrylium tetrafluoroborates **5** (acetonitrile, 20 °C).

Enamines	Electrophiles	*k*_2_ [M^−1^ s^−1^]	*N*, *s*_N_
**4 a** ^[a]^	**5 d**	7.06×10^1^	9.25, 0.84^[a]^
	**5 e**	2.14×10^1^	
	**5 f**	7.74	
	**5 g**	2.42	
**4 b**	**5 d**	4.24×10^1^	9.23, 0.74
	**5 e**	1.35×10^1^	
	**5 f**	5.53	
**4 c** ^[a]^	**5 a**	6.92×10^4^	7.74, 0.87^[a]^
	**5 b**	1.96×10^3^	
	**5 c**	8.84×10^1^	
		(5.49×10^1^)^[b]^	
**4 d**	**5 b**	5.36×10^3^	8.12, 0.88
	**5 c**	2.40×10^2^	
		(3.33×10^2^)^[b]^	
	**5 d**	8.33	
**4 e**	**5 b**	2.59×10^3^	7.79, 0.87
	**5 c**	9.49×10^1^	
	**5 d**	4.55	
**4 f**	**5 b**	1.15×10^4^	9.64, 0.70
	**5 c**	7.36×10^2^	
	**5 d**	6.36×10^1^	
	**5 e**	2.48×10^1^	
**4 g**	**5 a**	1.48×10^4^	7.92, 0.73
	**5 b**	9.68×10^2^	
	**5 c**	5.45×10^1^	
**4 h**	**5 b**	5.48×10^3^	8.30, 0.84
	**5 c**	2.34×10^2^	
		(2.71×10^2^)^[b]^	
	**5 d**	1.16×10^1^	
**4 i**	**5 a**	1.06×10^4^	7.10, 0.82
	**5 b**	4.31×10^2^	
	**5 c**	1.98×10^1^	
**4 j**	**5 a**	3.85×10^3^	6.49, 0.83
	**5 b**	1.37×10^2^	
	**5 c**	6.61	

[a] With data from Ref. [7]. [b] In dichloromethane.

**Figure 7:**
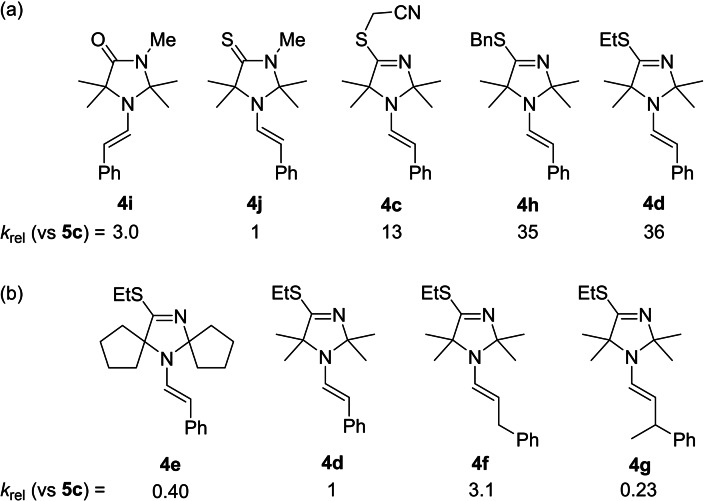
Comparison of relative rate constants *k*_rel_ for the reactions of enamines **4** with **5 c** (with data from Table 1) for (a) phenylacetaldehyde‐derived enamines and (b) *S*‐ethyl TIM‐derived enamines **4 d**–**4 g**.

Enamines **4 i** and **4 j** provide a direct comparison of the reactivity of enamines derived from an *N*‐methyl imidazolidinone and a structurally analogous sulfur derivative. Enamine **4 i** is about three times more reactive towards the cationic electrophiles **5**, such as **5 c**, than its sulfur analogue **4 j**. Figure 7a illustrates, however, that all TIM‐derived enamines are stronger nucleophiles than **4 i** and **4 j**. The reactivities of the *S*‐benzyl (**4 h**) and *S*‐ethyl (**4 d**) derivatives are by a factor of 35 and 36 higher, respectively, than that of the *N*‐methylated **4 j**. Even when the sulfur is substituted with an electron‐withdrawing cyanomethyl group, as in **4 c**, the reactivity still exceeds by a factor of 13 that of **4 j** (Figure 7a).

To further probe the nucleophilicity of the enamines, we modified the TIM ring system and the substituents on the enamine portion of the molecule (Figure 7b). When bulkier substituents were introduced at the 2‐ and 5‐positions of the TIM, the reactivity of the enamine decreased by a factor of 2.5 (**4 e** vs **4 d**), as expected. When the tether length of the carbon chain on the enamine was extended, the reactivity increased by a factor of 3 (**4 f** vs **4 d**). This may be due to a decrease in steric hindrance at the nucleophilic reaction center, as when an additional methyl group is introduced adjacent to the enamine moiety, the reactivity decreases by one order of magnitude (**4 g** vs **4 f**). The increase in reactivity from **4 d** to **4 f** may also be due to electronic factors, as the π‐system of the slightly electron withdrawing phenyl ring (σ_m_=0.06)^[20]^ is no longer in conjugation with the nucleophilic π‐system of the enamine. Accordingly, those enamines which are derived from purely aliphatic aldehydes (**4 a** and **4 b**) are by roughly 1.5 orders of magnitude more nucleophilic than enamine **4 c**. As enamines derived from phenylacetaldehyde are thermodynamically more stable than those derived from entirely aliphatic aldehydes,^[21]^ this increase in nucleophilicity is not surprising.

**Solvent effects**. The kinetics of the reactions of some enamines **4** with **5** were also determined in dichloromethane solution.^[15]^ As exemplified in Table 1, enamines **4 d** and **4 h** are slightly less reactive in acetonitrile than in dichloromethane (*k*_rel_=0.72 and 0.86, respectively), while enamine **4 c** is more reactive by a factor of 1.6. Thus, the nucleophilic reactivities of enamines **4** do not differ significantly in these two polar aprotic solvents, in accord with previous observations for enamines.^[13e]^ Further kinetic data along with *N* (and *s*_N_) parameters for enamine reactivities in dichloromethane are reported in the Supporting Information.

**Figure 8:**
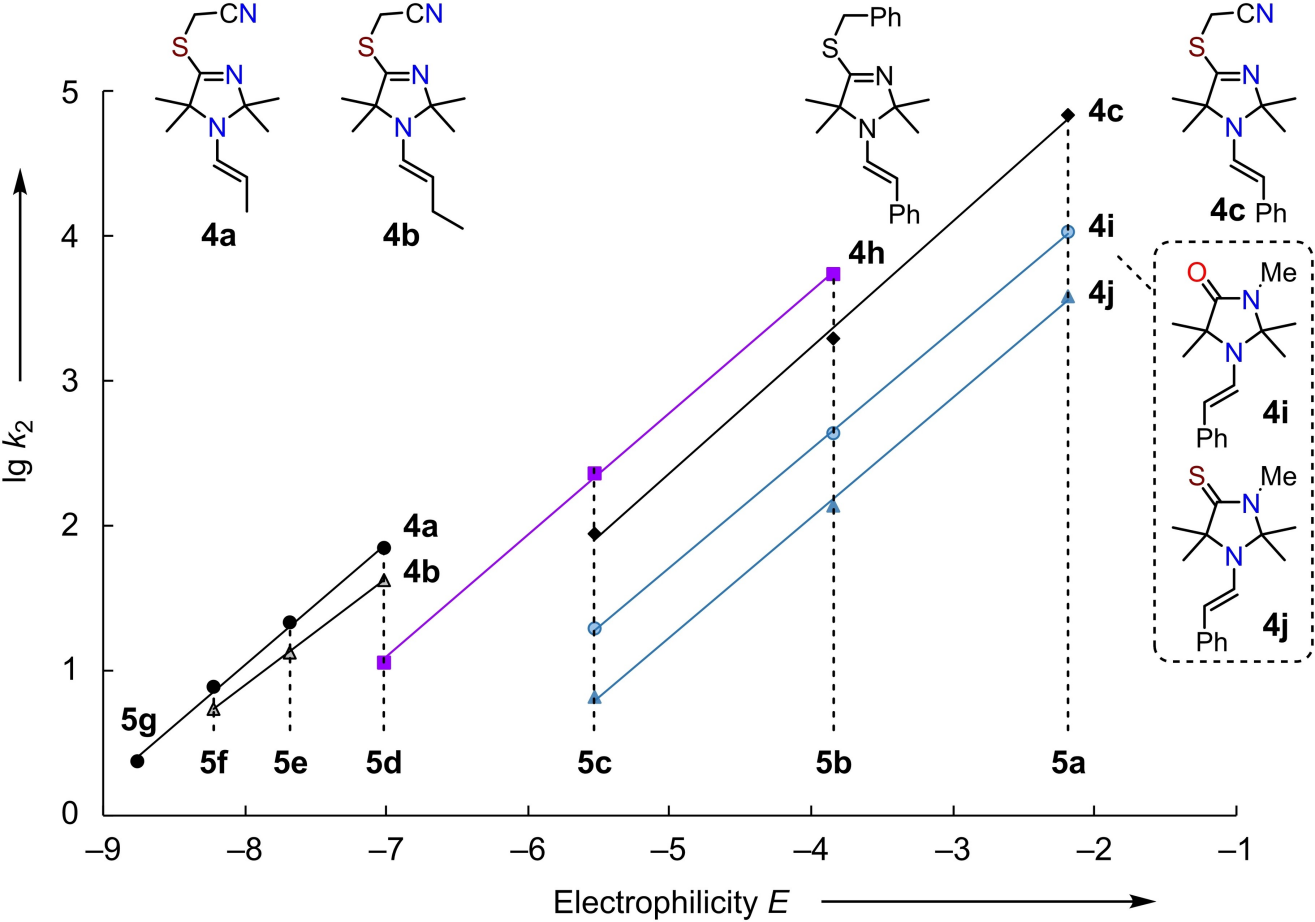
Linear correlations of second‐order rate constants (lg *k*_2_) for the reactions of enamines **4** with benzhydrylium ions **5** (MeCN, 20 °C) with the reported electrophilicities *E* of **5**.

**Nucleophilicities of enamines 4**. Figure 8 illustrates that the second‐order rate constants (lg *k*_2_) for the reactions of the enamines **4** with the electrophiles **5** in acetonitrile (from Table 1) correlate linearly with the previously reported electrophilicities *E* of the reference electrophiles **5 a**–**5 g** (cf. Figure 5). The corresponding plot of lg *k*_2_ vs *E* for enamines **4 d**–**4 g** is shown in Figure S1 (Supporting Information). The slopes of the correlation lines correspond to the nucleophilic‐specific susceptibilities *s*_N_. The nucleophilicity parameters *N* for the enamines **4** are graphically reflected by the negative values of the intercepts on the abscissa (that is, *N*=−*E* if lg *k*_2_=0). The resulting descriptors *N* and *s*_N_ for the nucleophilic reactivity of the enamines **4 a**–**4 j** are tabulated in Table 1.

The now available, experimentally determined nucleophilicity parameters *N* of the enamines **4 a**–**4 j** cover a range from 6.49 to 9.64. The same range of reactivities for **4 a**–**4 j** (6.59<*N*<9.64) is predicted by the current version of the reactivity structure and physicochemical (rSPOC) machine‐learning algorithm for the prediction of Mayr reactivity parameters, probably because a large set of kinetic data for enamines from Mayr's reactivity database^[12f]^ was available for the training of the artificial intelligence (AI) tool.^[22]^ However, comparison of individual experimental and predicted *N* values of the enamines shows some scatter (at average: Δ*N*=0.89, maximum Δ*N*=2.24 for **4 j**), which may partially be explained by the neglect of considering the individual susceptibility factors *s*_N_ of enamines in the prognostic AI model.^[22]^

**Figure 9:**
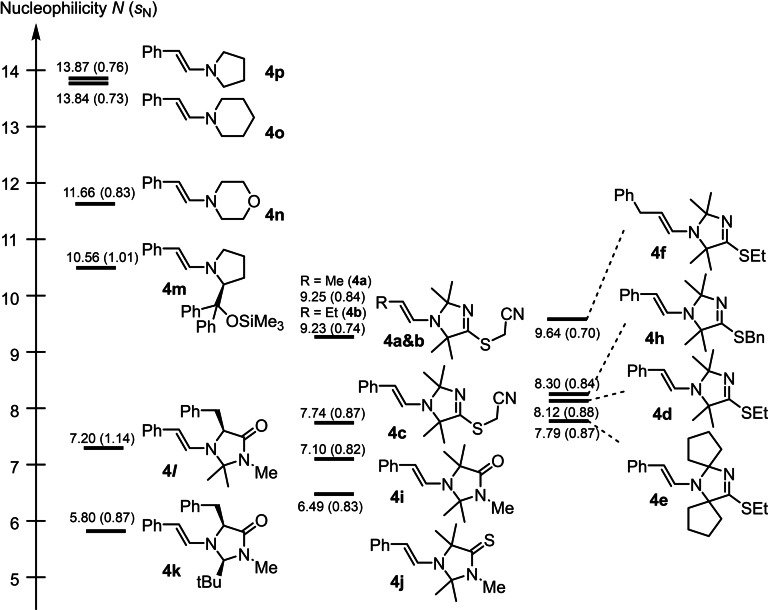
Comparison of nucleophilic reactivity between enamines derived from imidazolidinethiones, TIMs, MacMillan's 1^st^ and 2^nd^ generation organocatalyst, and the Jørgensen‐Hayashi organocatalyst (MeCN, 20 °C, with data from Table 1 and refs. [12f, 13]).

The experimentally determined nucleophilicities *N* facilitate a comparison of the reactivities of the enamines **4 a**–**4 j** with those of previously studied enamines, which are relevant in organocatalytic reactions or synthetic organic chemistry. As the *s*_N_ values of most enamines derived from aldehydes and cyclic secondary amines are in a narrow range (0.73<*s*_N_<0.88, except for **4** ***l*** and **4 m**), we can directly compare their nucleophilic reactivity along the nucleophilicity scale (Figure 9). Enamine **4 j**, which is derived from the *N*‐methylated ITO **2 d**, is only slightly more nucleophilic than the 2^nd^ generation MacMillan catalyst‐derived **4 k**, which is ‐ to date ‐ the enamine with the weakest nucleophilicity in Mayr's reactivity scales. Yet, **4 j** is still surpassed in reactivity by the structurally analogous enamine **4** ***l***, derived from the 1^st^ generation MacMillan catalyst.

Enamines **4 c**, **4 d**, and **4 h** derived from *S*‐alkylated ITOs (that is, TIMs) gain 2 to 2.5 orders of magnitude in reactivity if compared with **4 k**. However, **4 c**, **4 d**, and **4 h** are still less nucleophilic than the enamine **4 m** derived from the same aldehyde and diphenylprolinol trimethylsilyl ether (Hayashi‐Jørgensen catalyst).

**Lewis basicity of enamines 4**. It was previously shown that equilibrium constants *K* of reactions of benzhydrylium ions with many nucleophiles (or Lewis bases), including enamines,^[13e]^ can be calculated by using Equation (2).^[23]^ Equation (2) comprises only two parameters: one descriptor for Lewis acidity (*LA*) and one descriptor for Lewis basicity (*LB*). Individual sets of *LA* and *LB* parameters are used to calculate the equilibrium constant *K* for adduct formation in a specific solvent. We note, therefore, that in this work, we solely refer to *LA* and *LB* for reactions in acetonitrile.
(2)

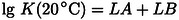




We observed that the slow reaction of enamine **4 d** with the benzhydrylium salt **5 d** did not reach completion when we studied the kinetics of this electrophile‐nucleophile combination. In order to measure the Lewis basicity of enamine **4 d**, we determined the equilibrium constants *K*, as defined in Equation (3), of reactions of **4 d** with **5 d** as well as with the less Lewis acidic benzhydrylium ions **5 e** and **5 f**.

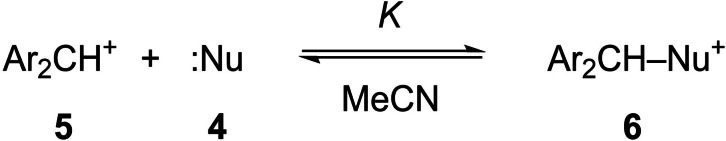




Analogous to previous studies on Lewis basicities of enamines,^[13e]^ we used photometric titrations in acetonitrile at 20 °C, in which enamine **4 d** was added in small portions to the colored solutions of **5**. After each titration step *i* the remaining absorbance *A*_eq,*i*_ of **5** was determined. Assuming the validity of the Beer–Lambert law for the dilute solutions, the equilibrium constant was then derived from Equation 4, the initial absorbance *A*_0_*,^[24]^ and the absorbance at equilibrium *A*_eq,*i*_ after titration step *i*.^[15]^


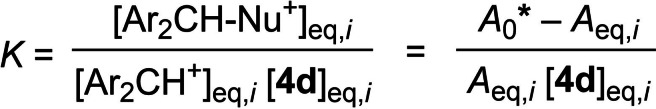




The linear relationships of (*A*_0_*−*A*_eq,*i*_)/*A*_eq,*i*_ with the concentration of **4 d** at equilibrium [Equation (5)] allowed us to determine the equilibrium constants *K* for reactions with **5 d**–**5 f** (Table 2) from the slopes of these correlations.^[15]^

(5)

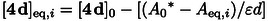




**Table 2:** Lewis acidities *LA* of benzhydrylium ions **5** and the equilibrium constants *K* for the reactions of **5** with enamine **4 d** (in acetonitrile at 20 °C).

Ar_2_CH^+^	*LA*_MeCN_^[a]^	*K* [M^−1^]
**5 d**	−9.82	914
**5 e**	−10.83	58.8
**5 f**	−11.27	24.5

[a] From Ref. [23].

**Figure 10:**
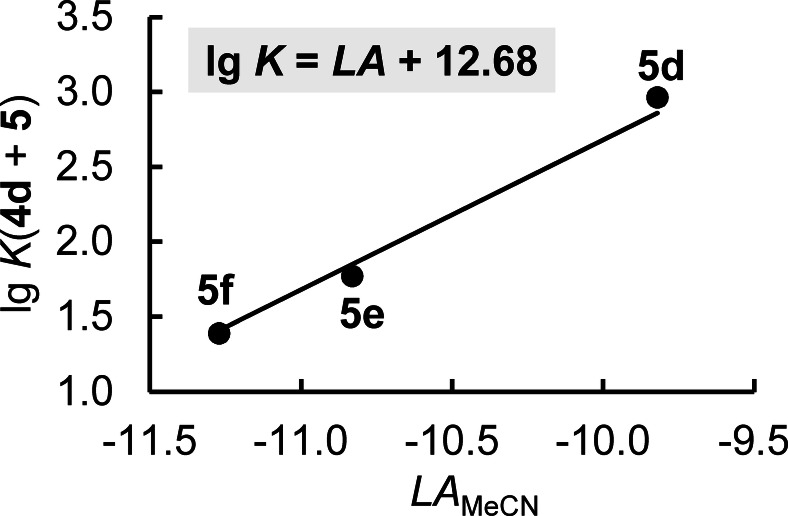
Determination of *LB*_MeCN_ for **4 d** from a plot of lg *K*(**4 d**+**5**) vs *LA*_MeCN_ of **5** (with data from Table 2). The correlation line was drawn with a slope fixed to unity.

**Figure 11:**
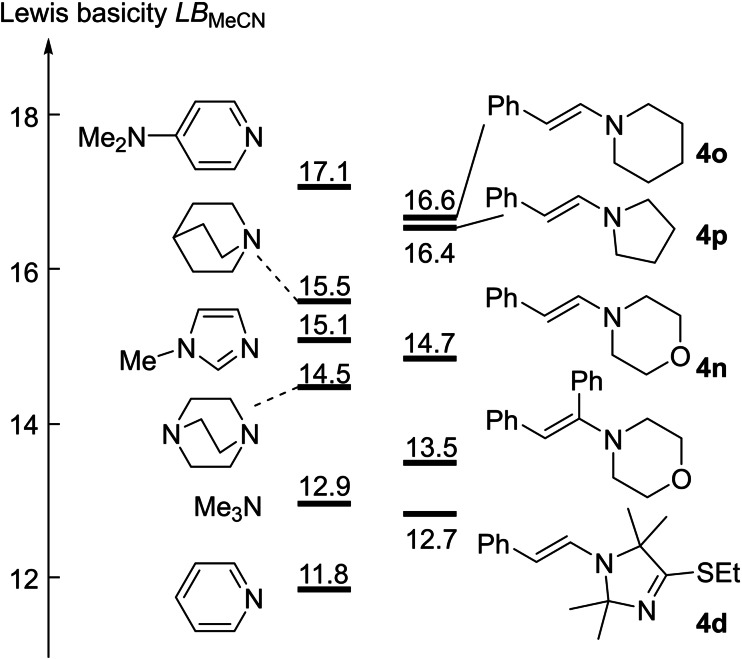
Comparison of the Lewis basicities *LB* (in MeCN) of α‐ and/or β‐phenyl‐substituted enamines with *LB*_MeCN_ for *tert*. amines, pyridines, and imidazoles (with data from Refs. [13e, 23]).

The Lewis basicity *LB*_MeCN_=12.68 of **4 d** was derived from a least squares analysis of the data in Table 2 by using Equation (2) and corresponds to the intercept of the correlation equation in Figure 10. With *LB*_MeCN_ a further comparison of the Lewis basic properties of enamine **4 d** with other enamines becomes possible. As depicted in Figure 11, **4 d** is the so far least Lewis basic enamine characterized by the benzhydrylium method, which also reflects its location in the nucleophilicity scale (Figure 9). Yet, **4 d** is still more Lewis basic towards C‐centered Lewis acids than pyridine, which is an often used, efficient Lewis base catalyst, for example for acylation reactions.^[25]^

**Figure 12:**
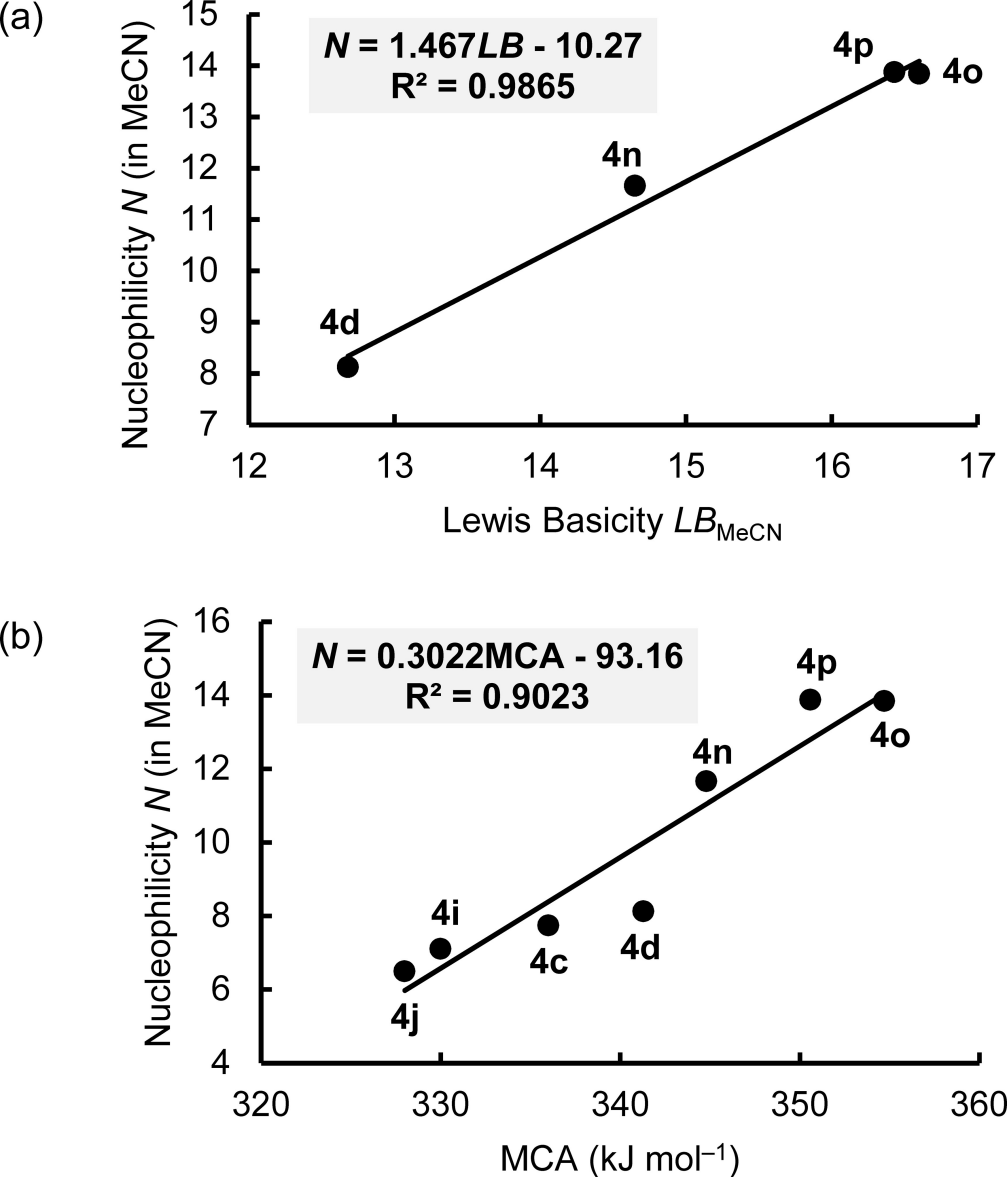
The nucleophilicity parameters *N* of β‐aminostyrenes correlate linearly with (a) their experimental Lewis basicities *LB* and (b) their DFT‐calculated MCA values (MeCN, 20 °C, with MCA values for **4 n** (344.8), **4 o** (354.7), and **4 p** (350.6) from Ref. [13e]).

Intuition may suggest that structural variations in electron‐rich species, which increase Lewis basicities, also increase nucleophilicities. However, the relationship of kinetics and thermodynamic driving force is complicated by many factors that can have an influence on the nucleophilicity in solution.^[26]^ That is why correlations between nucleophilicity and basicity occasionally collapse even when reactions of nucleophiles with the same central atom are compared.[^23b^, ^27^] By restricting structural variation to a minimum, that is, to changes only in the nitrogen‐containing heterocyclic part of the phenylacetaldehyde‐derived enamines **4 d**, **4 n**, **4 o**, and **4 p**, it is possible to achieve a linear correlation of the enamine nucleophilicities *N* with their experimental *LB* values in acetonitrile (Figure 12a).

The correlation of nucleophilicity parameters *N* with the DFT‐calculated MCA values of six phenylacetaldehyde‐derived enamines in Figure 12b shows an additional option to interconnect kinetics with the thermodynamics of the carbon‐carbon bond formation. Owing to the fact that MCA values can be efficiently computed for further types of enamines, the correlation in Figure 12b provides a useful tool for the design of further enamines with predictable reactivity toward electrophiles.

**Nucleofugality of enamines 4**. Having the Gibbs energy of activation Δ*G* ^≠^ for the forward reactions of **4 d** with benzhydrylium ions **5 d**–**f** and the Gibbs reaction energy Δ_r_ *G*° available for the same reactions makes it possible to calculate the energetic barrier for the reverse additions, Δ*G* ^≠^_rev_ (Table 3). Analogously, unimolecular rate constants for the collapse of the enamine/benzhydrylium‐adduct can be calculated from the relationship *k*_rev_ (s^−1^)=*k*_2_/*K*.

**Table 3:** Fugality parameters for retroadditions of enamines **4** to benzhydrylium ions **5** in MeCN at 20 °C (Gibbs energies in kJ mol^−1^).

Reactions	*k*_2_ [M^−1^ s^−1^]	Δ*G* ^≠^	*K* [M^−1^]	Δ_r_ *G*°	Δ*G* ^≠^_rev_	*k*_rev_ [s^−1^]	Electrofugality *E*_f_^[a]^	Nucleofugality *N*_f_, *s*_f_
**4 d+5 d**	8.33	66.6	9.14×10^2^	‐16.6	83.2	9.2×10^−3^	4.84	−6.35, 1.34
**4 d+5 e**	2.4^[b]^	69.6	5.88×10^1^	‐9.9	79.5	4.2×10^−2^	5.35	
**4 d+5 f**	0.82^[b]^	72.2	2.45×10^1^	‐7.8	80.0	3.4×10^−2^	5.22	
**4 n+5 e**	2.00×10^3[c]^	53.2	6.61×10^3[d]^	‐21.4	74.6	3.1×10^−1^	5.35	(−5.73)^[e]^
**4 o+5 e**	3.02×10^4[c]^	46.6	5.89×10^5[d]^	‐32.4	79.0	5.1×10^−2^	5.35	(−6.31)^[e]^
**4 p+5 e**	4.31×10^4[c]^	45.7	3.98×10^5[d]^	‐31.4	77.1	1.1×10^−1^	5.35	(−6.06)^[e]^

[a] Electrofugalities *E*_f_ for **5 d**‐**f** from Ref. [28]. [b] Calculated by substituting *E*, *N*, and *s*_N_ in Equation (1). [c] From Ref. [13e]. [d] Calculated with *LA* and *LB* values from Ref. [13e] by using Equation (2). [e] Estimated *N*_f_ of enamines **4 n**–**p** by assuming *s*_f_=1.34 as for **4 d**.

**Figure 13:**
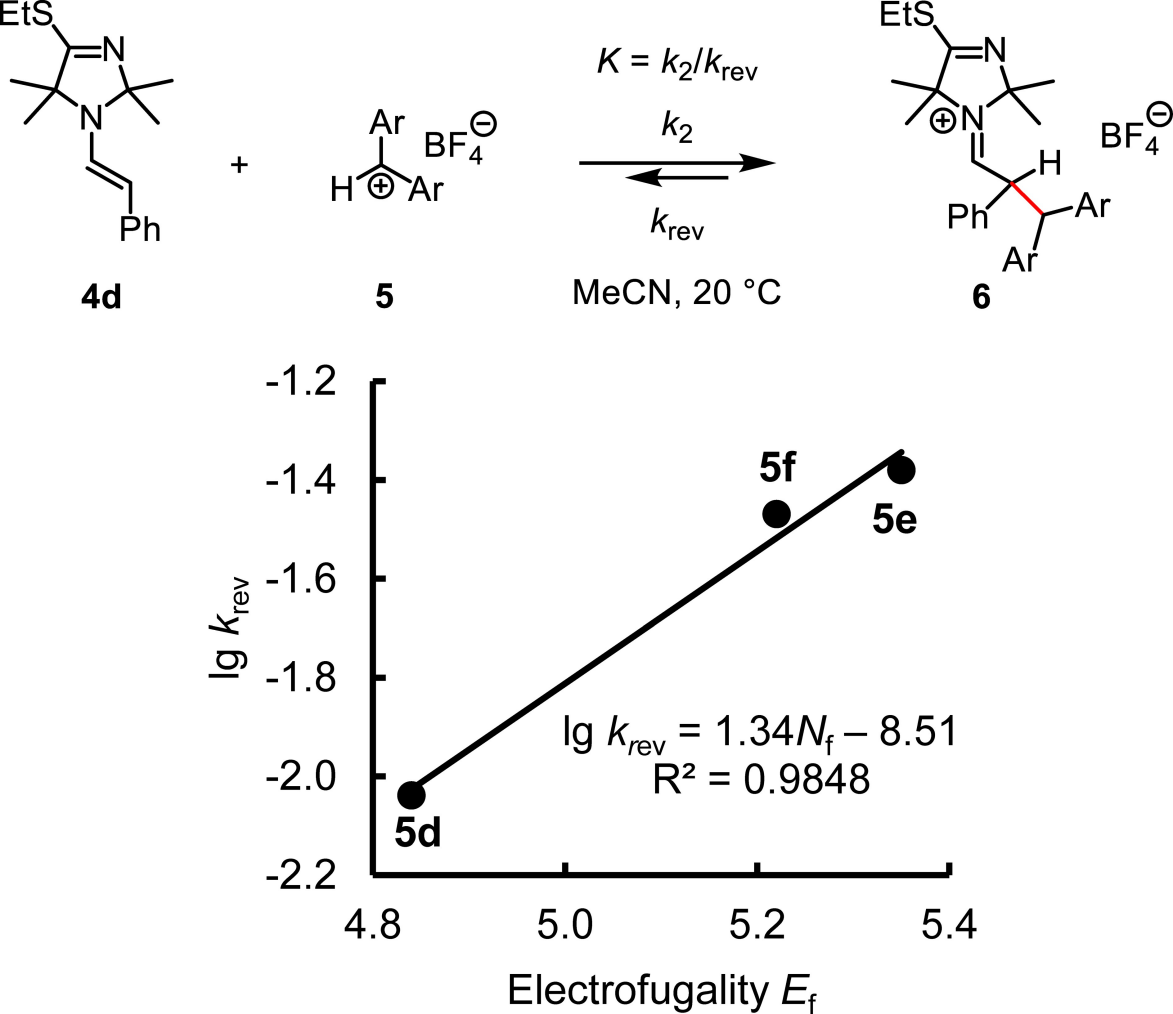
Nucleofugality of **4 d** in MeCN determined from the linear correlation of lg *k*_rev_ (s^−1^) with the electrofugality *E*_f_ of benzhydrylium ions [with data from Table 3 and by using Equation (6)].

With the thus determined *k*_rev_ and by using the Mayr‐Kronja Equation 6, ^[28]^

(6)

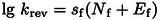




we can quantify the leaving group ability of enamine **4 d** in acetonitrile by the nucleofugality parameters *N*_f_ and *s*_f_, because benzhydrylium ions **5** have already been established as reliable reference electrofuges and their solvent‐independent *E*_f_ values have been reported.[^28^, ^29^] Figure 13 shows the linear correlation of lg *k*_rev_ with *E*_f_(**5**), the slope of which corresponds to the *s*_f_ parameter (*s*_f_=1.34). The nucleofugality *N*_f_=−6.35 is then determined by division of the intercept −8.51 by *s*_f_ (*N*_f_=−8.51/1.34=−6.35).

The nucleofugalities *N*_f_ of the enamines **4 n**, **4 o**, and **4 p** can be estimated by assuming the same *s*_f_ values as for **4 d**. Though the enamines **4 d**, **4 n**, **4 o**, and **4 p** spread over a nucleophilicity range from *N*=8.12 to 13.87 and vary in Lewis basicities *LB* from 12.7 to 16.6, their leaving group abilities expressed by the nucleofugalities *N*_f_ are within the same order of magnitude. This comparison shows that TIM‐derived enamines, such as **4 d**, are weak nucleofuges with comparable leaving group abilities as typically found also for enamines derived from the same carbonyl compound but with more classical conventional amine moieties.

**Oxidation potentials of enamines 4**. It has previously been discussed that electrochemically determined oxidation potentials *E*_ox_ might be related to the Mayr nucleophilicity *N* of enamines.^[30]^ In lack of experimental data for tertiary enamines, the S. Luo group used experimental peak potentials *E*_p_ of secondary enamines to benchmark quantum‐chemical (DFT‐)calculations of enamine oxidation potentials. This DFT method was subsequently used to estimate *E*_ox_ of tertiary enamines. Available Mayr *N* parameters for nine tertiary enamines were shown to correlate linearly with the thus determined oxidation potentials (*r* ^2^=0.87).^[30]^

**Figure 14:**
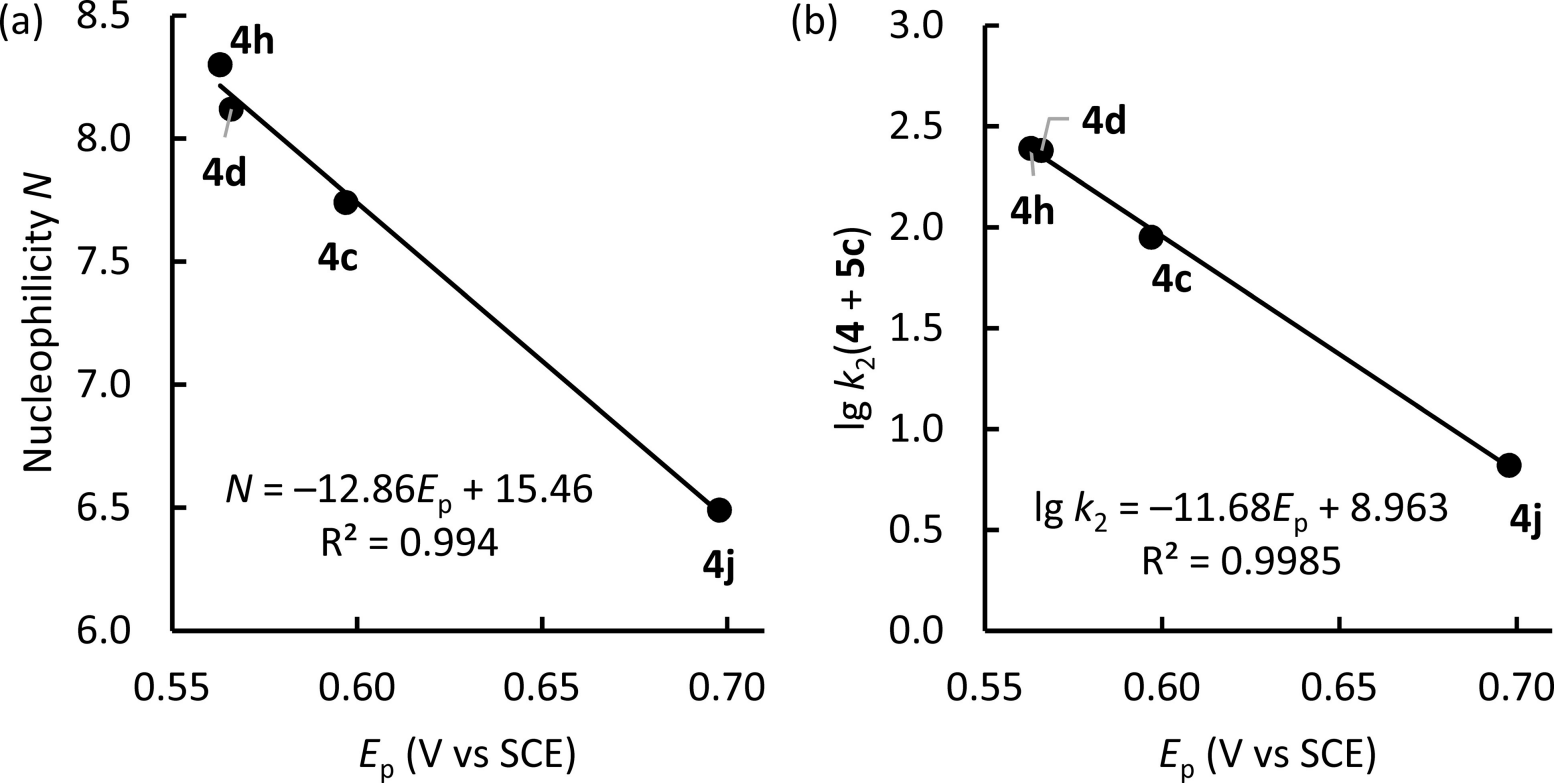
Correlations of (a) nucleophilicities *N* and (b) individual reactivities (lg *k*_2_) toward benzhydrylium ion **5 c** with peak potentials *E*_p_ for the oxidation of enamines **4** in MeCN (vs. SCE, internally referenced with fc/fc^+^).

We have now used cyclic voltammetry to experimentally determine the oxidation potentials of the phenylacetaldehyde‐derived enamines **4 c**, **4 d**, **4 h**, and **4 j** in a 0.1 M tetra‐*n*‐butyl ammonium perchlorate solution of degassed acetonitrile. Ferrocene was used as internal standard.^[31]^ Owing to irreversible one‐electron transfer only peak potentials *E*_p_ could be evaluated.^[15]^ Nevertheless, Figure 14 demonstrates that excellent correlations connect the electron‐pair donating properties of enamines, that is, their nucleophilicities, with their one‐electron donicities, expressed by *E*_p_.

## Conclusions

We have isolated and characterized a series of enamines which were derived from *S*‐alkylated imidazolidine‐4‐thiones and propanal, butanal, phenylacetaldehyde, 3‐phenylpropanal, or 3‐phenylbutanal. Such enamines are key intermediates in organocatalytic α‐alkylations of enolizable aldehydes, and their nucleophilicity in acetonitrile has been calibrated by using Mayr's benzhydrylium methodology. It is demonstrated that the determined nucleophilicity parameters *N* of the enamines correlate linearly with experimental Lewis basicities, quantum‐chemically calculated methyl cation affinities, and electrochemically measured oxidation potentials. These links between thermodynamic and kinetic data will (a) facilitate the future design of enamines with tailor‐made properties and (b) foster the understanding of prebiotic carbon chain elongations of aldehydes by α‐alkylations, which may have been catalyzed by prebiotically likely cyclic amines, such as TIMs. Their relevance in photocatalytic reactions[^4^, ^7^, ^8^] is currently investigated in our labs.

## Experimental Section

**Chemicals**. Enamines **4 a**–**4 j** were prepared as described in the Supporting Information. Benzhydrylium tetrafluoroborates **5 a**–**5 g** (reference electrophiles) were synthesized as described previously.[^12b^, ^18^, ^19^] Supporting Information contains procedures for the reactions of **4** with **5** and characterization data for iminium salt **6** and aldehydes **7**.

**Kinetics**. The kinetics of the reactions of the enamines **4** with the electrophiles **5** were followed by UV/Vis spectroscopy by using stopped‐flow techniques. Details of the kinetic experiments are given in the Supporting Information.

**Determination of equilibrium constants**. Equilibrium constants were determined photometrically through titration experiments as described in ref.^[13e]^ Details are reported in the Supporting Information.

**Cyclic voltammetry**. Cyclic voltammetry (CH Instruments 630E electrochemical analyzer) measurements with enamines **4** (Supporting Information, Figure S8) were performed under argon in a 0.1 M tetra‐*n*‐butyl ammonium perchlorate solution of degassed acetonitrile with a 2 mm diameter platinum working electrode, a platinum wire counter electrode, and a Ag wire pseudo‐reference electrode. Ferrocene was used as internal standard [*E*_1/2_(fc/fc^+^)=+0.382 V vs SCE] for referencing to SCE.^[31]^

**DFT Calculations**. Details are reported in the Supporting Information.

## Conflict of interest

The authors declare no conflict of interest.

1

## Supporting information

As a service to our authors and readers, this journal provides supporting information supplied by the authors. Such materials are peer reviewed and may be re‐organized for online delivery, but are not copy‐edited or typeset. Technical support issues arising from supporting information (other than missing files) should be addressed to the authors.

Supporting Information

## Data Availability

The data that support the findings of this study are available from the corresponding author upon reasonable request.
