# Supplementary material for: Nucleophilicity of 4‐(Alkylthio)‐3‐imidazoline Derived Enamines
Source: Chemistry. 2023 Nov 15;30(2):e202302764. doi: 10.1002/chem.202302764 (PMC10962604; doi:10.1002/chem.202302764)
Supplement: Supplementary file 1 — Supporting Information [file CHEM-30-0-s001.pdf]

Chemistry–A European Journal

Supporting Information

Nucleophilicity of 4-(Alkylthio)-3-imidazoline Derived Enamines

Magenta J. Hensinger, Andreas Eitzinger, Oliver Trapp,* and Armin R. Ofial*

Table of Contents

1. Additional Figures S2

2. General S3

3. Preparation of TIMs and Enamines S6

  3.1 Preparation of 4-(Alkylthio)-2,5-dihydro-1*H*-imidazoles **3** (TIMs) S6

  3.2 Preparation of Enamines **4** S8

4. Reactions of Enamines **4** with Benzhydrylium Tetrafluoroborates **5** S14

5. Kinetic Experiments S26

  5.1 Kinetics in Acetonitrile S26

  5.2 Kinetics in Dichloromethane S40

6. Oxidation Potentials of Enamines **4** S44

7. Determination of Equilibrium Constants S46

8. Copies of ^{1}H NMR, ^{13}C NMR, and IR Spectra S53

9. Computational Details S117

10. References S145

1. Additional Figures

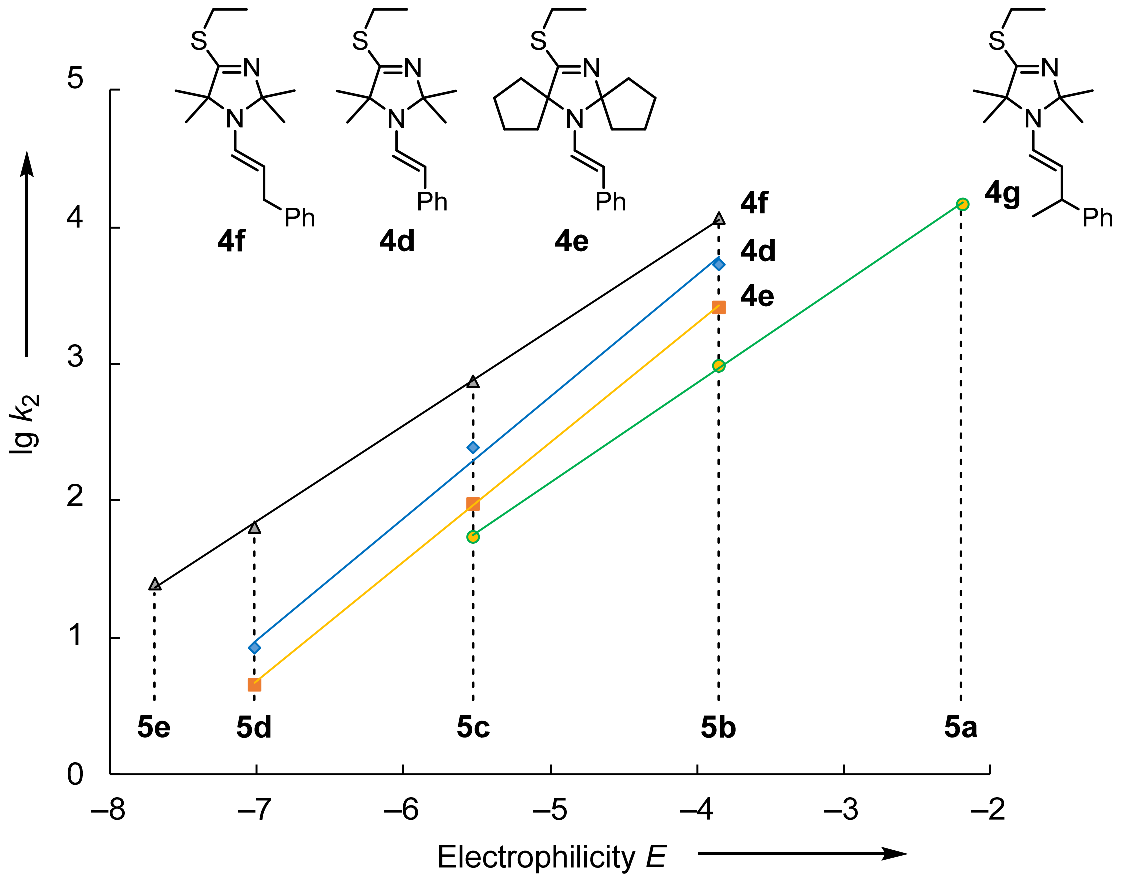

Figure S1. Linear correlations of second-order rate constants (lg k_2) for the reactions of enamines **4d-4g** with reference electrophiles **5a–5e** (MeCN, 20 °C) with the known electrophilicities E (from ref.[S1]) of the benzhydrylium ions **5** (reference electrophiles).

2. General

Chemicals. Chemicals used in the synthesis of the imidazolidinethiones, imidothioates, and enamines were purchased from commercial sources (Acros Organics b.v.b.a., Sigma-Aldrich Co. LLC, Alfa Aesar by Thermo Fischer Scientific, VWR International GmbH, and TCI Europe N.V.) and used without purification unless otherwise indicated. Tetrahydrofuran was dried using sodium metal and distilled prior to use. Commercially available acetonitrile (99% extra dry over molecular sieves, Acros Organics), toluene (≥99.7% pure, Sigma-Aldrich), or dichloromethane (HPLC grade, VWR) used in synthetic procedures were used without purification. Molecular sieves (4 Å) were heated to 350 °C under vacuum for 6 h and then stored in a desiccator. All reactions were performed using dried glassware (dried using a heat gun under vacuum) under an atmosphere of nitrogen or argon except when using aqueous reagents.

Reactions were monitored using thin layer chromatography with either silica gel 60 aluminum backed plates with F-254 fluorescence indicator or neutral aluminum oxide 60 aluminum backed plates with F-254 fluorescence indicator (both from Merck). Pentane was distilled prior to use for column chromatography. Triethylamine was used without purification. Flash column chromatography was performed on either silica gel 60 (0.040-0.063 nm) purchased from Merck or neutral aluminum oxide from Sigma Aldrich.

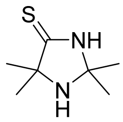

2a

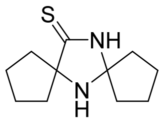

2b

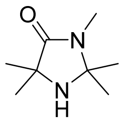

2c

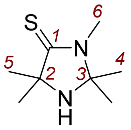

2d

2,2,5,5-Tetramethyl-4-imidazolidinthione (**2a**) was prepared as reported in ref.[S2]

6,12-Diazadispiro[4.1.4^7.2^5]tridecane-13-thione (**2b**) (MJH-I-60) was obtained as described before:[S2b] To a solution of ammonium sulfide (11.2 mL), sodium cyanide (1.25 g, 25.5 mmol), and ammonium chloride (1.39 g, 26.0 mmol) in H_2O (3.4 mL) was added cyclopentanone (4.50 mL, 51.0 mmol) dropwise. The resulting solution was heated to reflux for 6 h, and a white precipitate formed. The reaction was left to cool at room temperature and filtered. The white powder was washed with cold deionized H_2O and suction and dried: **2b** (4.43 g, yield: 83%); fluffy white powder, m.p. 200.4 °C (dec)[S2c]: R_f = 0.65 (silica gel, pentane/EtOAc 6:1. ^{1}H NMR (600 MHz, $CDCl_3$): δ = 9.31 (s, 1 H), 2.35–2.27 (m, 2 H), 2.07 (s, 1 H), 1.94–1.90 (m, 2 H), 1.88–1.73 (m, 10 H), 1.69–1.65 (m, 2 H). ^{13}C{^{1}H} NMR (151 MHz, $CDCl_3$): δ = 208.9 (C_q), 88.8 (C_q), 80.6 (C_q), 41.5 (CH_2), 39.9 (CH_2), 25.9 (CH_2), 23.8 (CH_2). IR (ATR, neat): 3253, 3108, 2959, 2865, 1511, 1431, 1351, 1328, 1186,

1157, 1113, 1087, 1059, 985, 953, 898, 845, 771 cm^{-1}. HRMS (EI): *m/z* calcd for $C_{11}H_{18}N_2S^{•+}$ [$M^{•+}$]: 210.1185; found: 210.1183.

2,2,3,5,5-Pentamethyl-4-imidazolidinone (**2c**) was prepared according to a literature procedure.[S3]

2,2,3,5,5-Pentamethyl-4-imidazolidinethione (**2d**) (MJH-I-170//MJH-II-19): According to ref.[S4] P_2S_5 (5.4 g) and Al_2O_3 (9.0 g) were ground into a homogenous powder using a mortar and pestle. To a solution of 2,2,3,5,5-pentamethylimidazolidin-4-one (916 mg, 5.86 mmol) in toluene (35 mL) was added the P_2S_5/Al_2O_3 mixture (2.4 g) and heated at reflux for 24 h. The reaction was cooled at ambient temperature, and the solution decanted from the solids. A mixture of H_2O (30 mL) and CH_2Cl_2 (20 mL) was added to the solid residual, and the suspension was vigorously shaken in a separatory funnel. The CH_2Cl_2 layer was separated, dried over $MgSO_4$, and added to the decanted toluene solution. The volatiles were removed in vacuo and left a crude brown oil, which was purified by flash chromatography (silica gel, EtOAc): **2d** (167 mg, yield: 17%); pale yellow powder,[S5] m.p. 68.1 °C; R_f = 0.21 (silica gel, EtOAc). **^{1}H NMR** (600 MHz, $CDCl_3$): δ = 3.18 (s, 3 H), 1.92 (br. s, 1 H, NH), 1.45 (s, 6 H), 1.45 (s, 6 H). **^{13}C{^{1}H} NMR** (151 MHz, $CDCl_3$): δ = 205.3 (C_q, C-1), 82.8 (C_q, C-3), 70.1 (C_q, C-2), 31.5 (CH_3, C-6), 30.4 (CH_3), 27.8 (CH_3). **IR** (ATR, neat): 3283, 2970, 2928, 1503 (br.), 1451, 1403, 1386, 1368, 1249, 1188, 1109, 1016, 888, 810 cm^{-1}. **HRMS** (EI): *m/z* calcd for $C_8H_{16}N_2S^{•+}$ [$M^{•+}$]: 172.1029; found: 172.1028.

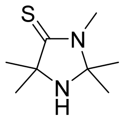

2d

Enamines **4a**–**4g** decompose over time in the presence of moisture and were, therefore, stored in a glovebox freezer (at < –30 °C) under a dry argon atmosphere.

Analytics. ^{1}H and ^{13}C{^{1}H} NMR spectra were recorded at 400, 600, or 800 MHz and at 101, 151, or 201 MHz, respectively. The chemical shifts (δ) for ^{1}H and ^{13}C nuclei are given in ppm relative to the signals of the solvents ($CDCl_3$: δ_H = 7.26 ppm and δ_C = 77.2 ppm; CD_3CN; δ_H = 1.94 ppm and δ_C = 118.3 ppm).[S6] Coupling constants are given in Hz, and the assignments of NMR signals are based on additional 2D-NMR experiments (gHSQC, gHMBC, COSY, and NOESY). Multiplicities are abbreviated as follows: s = singlet, d = doublet, t = triplet, q = quartet, p = pentet, m = multiplet, br s = broad singlet. High-resolution mass spectra (HRMS) were obtained by using a Thermo Finnigan MAT 95 instrument (EI) or a Thermo Finnigan LTQ FT (ESI). IR spectra for neat compounds were recorded on an FTIR Spectrometer SPECTRUM BX II (Perkin Elmer) with ATR probe. Melting points were measured using a Büchi melting-point M-560 device and are not corrected.

Kinetics. The kinetics of the reactions of the enamines **4** with the electrophiles **5** were followed by UV/Vis spectroscopy by using an Applied Photophysics SX.20 stopped-flow spectrophotometer (10 mm light path). A constant temperature (20.0 ± 0.2 °C) was maintained through the use of a circulating bath cryostat. All solutions were prepared under an atmosphere of argon or nitrogen with HPLC grade acetonitrile (VWR) flushed with nitrogen or freshly distilled dichloromethane.

The kinetic measurements were initiated by mixing equal volumes of acetonitrile (or dichloromethane) solutions of the nucleophiles and electrophiles. Nucleophile concentrations were at least ten times higher than electrophile concentrations to achieve pseudo-first order kinetics. The first-order rate constants k_{obs} (s^{-1}) could be obtained from the decay of the absorbance at or close to the absorption maximum of the colored reference electrophiles by least squares fitting of the equation $A_t = A_0 \exp(-k_{obs}t) + C$ to the exponential absorption decay curve. Plots of k_{obs} (s^{-1}) versus the nucleophile concentration gave the second-order rate constants k_2 (M^{-1} s^{-1}) as slopes of the linear correlations.

Determination of Equilibrium Constants. Analogous to the procedure described in ref.[S7] the equilibrium constants *K* for the addition of enamine **4d** (Lewis base) with benzhydrylium ions **5d**-**5f** (Lewis acids, counterion: tetrafluoroborate) were determined by photometrically following the decay of the Lewis acid concentration. Measurements were conducted on a J&M TIDAS diode array spectrophotometer controlled by J&M TidasDAQ 3 software (v 3.8.1) connected to a Hellma 661.502-QX quartz Suprasil immersion probe (5 nm light path) via fiber optic cables and standard SMA connectors. A constant temperature (20.0 ± 0.2) was maintained by using a circulating bath cryostat. A small volume of a stock solution of enamine **4d** (in acetonitrile) was added to a solution of the benzhydrylium tetrafluoroborate **5** (in acetonitrile) at which point the absorbance decayed from a constant A_0. After equilibration and the absorbance became constant (A_{eq}), another portion of the enamine **4d** stock solution was added. Such titrations were carried out twice for each Lewis acid **5**, and then the individual equilibrium constants *K* were averaged for each **4d** + **5** combination.

3. Preparation of TIMs and Enamines

3.1 Preparation of 4-(Alkylthio)-2,5-dihydro-1*H*-imidazoles **3** (TIMs)

General Procedure A (*GP A*).[S8] To a solution of NaH (60% dispersion in mineral oil, 2.1 equiv.) in freshly distilled THF (4 mL/mmol NaH) was added a solution of imidazolidine-4-thione **2a** or **2b** (1 equiv.) in freshly distilled THF (9 mL/mmol) dropwise at room temperature. After the complete addition, the reaction was left to stir for 10 min, and the corresponding alkylating agent (2 equiv. of bromoacetonitrile, ethyl iodide or benzyl bromide) was added dropwise to the solution. The reaction was left to stir for 90 min or until the disappearance of starting material was detected by TLC. The solvent was evaporated in vacuo, and the solid residues were dissolved in H_2O (50 mL). The aqueous phase was extracted with diethyl ether (3 × 25 mL). The combined organic phases were washed with brine (30 mL), dried over $MgSO_4$, and the solvent removed in the vacuum. The crude product was purified by column chromatography.

2-((2,2,5,5-Tetramethyl-2,5-dihydro-1*H*-imidazol-4-yl)thio)acetonitrile (**3a**)

See synthetic procedure described in ref.[S9]

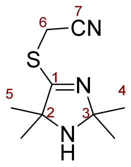

3a

4-(Ethylthio)-2,2,5,5-tetramethyl-2,5-dihydro-1*H*-imidazole (**3b**) (MJH-I-14) was obtained (*GP A*) from 2,2,5,5-tetramethylimidazolidine-4-thione (1.19 g, 7.52 mmol) and ethyl iodide (1.2 mL, 14.9 mmol). The crude product (pale yellow oil) was purified by flash chromatography (silica gel, CH_2Cl_2/Et_2O 1:1): **3b** (861 mg, yield: 61%); colorless oil; R_f = 0.31 (silica gel, EtOAc).

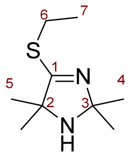

3b

^{1}H NMR (400 MHz, $CDCl_3$): δ = 3.02 (q, J = 7.3 Hz, 2 H, 7-H), 1.86 (br. s, 1 H, N-H), 1.41 (s, 6 H, 4-H), 1.33 (s, 6 H, 5-H), 1.33 (t, J = 7.8 Hz, 3 H, 7-H). **^{13}C{^{1}H} NMR** (101 MHz, $CDCl_3$): δ = 174.7 (C_q, C-1), 88.9 (C_q, C-3), 70.7 (C_q, C-2), 30.7 (CH_3, C-4), 29.1 (CH_3, C-5), 25.5 (CH_2, C-6), 14.1 (CH_3, C-7). **IR** (ATR, neat): 3330, 2971, 2929, 1592, 1461, 1443, 1374, 1363, 1212, 1168, 1028, 964, 828 cm^{-1}. **HRMS** (EI): m/z calcd for $C_9H_{19}N_2S^+$ [M+H]: 187.1263; found: 187.1259.

4-(Benzylthio)-2,2,5,5-tetramethyl-2,5-dihydro-1*H*-imidazole (**3c**) (MJH-I-76) was obtained (*GP A*) from 2,2,5,5-tetramethylimidazolidine-4-thione (916 mg, 5.79 mmol) and benzyl bromide (1.40 mL, 11.8 mmol). The crude product (pale-yellow oil) was purified by flash chromatography (silica gel, CH_2Cl_2/Et_2O 5:1): **3c** (952 mg, yield: 66%); colorless oil which solidified to an off white solid in the fridge (7 °C), m.p. 55.2 °C; R_f = 0.28 (silica gel, EtOAc).

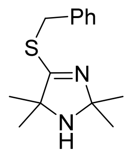

3c

^{1}H NMR (600 MHz, $CDCl_3$): δ = 7.39–7.35 (m, 2 H), 7.32–7.28 (m, 2 H), 7.26–7.23 (m, 1 H), 4.27 (s, 2 H), 1.84 (s, 1 H), 1.44 (s, 6 H), 1.33 (s, 6 H). **^{13}C{^{1}H} NMR** (151 MHz, $CDCl_3$): δ = 174.4 (C_q), 137.3 (C_q), 129.1 (CH), 128.6 (CH), 127.4 (CH), 89.0 (C_q), 70.6 (C_q), 35.8 (CH_2), 30.8 (CH_3), 29.0 (CH_3). **IR** (ATR, neat): 3312, 2971, 2926, 1590, 1494, 1443, 1425, 1366, 1212, 1164, 1143, 1032, 1002, 964, 924, 895, 824, 770, 722, 705, 694 cm^{-1}. **HRMS** (ESI): *m/z* calcd for $C_{14}H_{21}N_2S^+$ [M+H$^+$]: 249.1420; found: 249.1419.

13-(Ethylthio)-6,12-diazadispiro[4.1.4^7.2^5]tridec-12-ene (**3d**) (MJH-I-61) was obtained (*GP A*) from 6,12-diazadispiro[4.1.4^7.2^5]tridecane-13-thione (1.52 g, 7.22 mmol) and ethyl iodide (1.20 mL, 14.9 mmol). The crude product (pale yellow oil) was purified by flash chromatography (silica gel, CH_2Cl_2/Et_2O 16:1): **3d** (1.58 g, yield: 92%); colorless oil; R_f = 0.46 (silica gel, EtOAc).

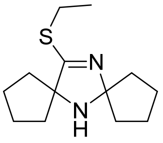

3d

^{1}H NMR (400 MHz, $CDCl_3$): δ = 3.04 (q, *J* = 7.4 Hz, 2 H), 2.04–1.93 (m, 2 H), 1.93–1.67 (m, 12 H), 1.66–1.56 (m, 4 H), 1.33 (t, *J* = 7.4 Hz, 3 H). **^{13}C{^{1}H} NMR** (151 MHz, $CDCl_3$): δ = 174.6 (C_q), 98.7 (C_q), 79.7 (C_q), 40.5 (CH_2), 39.4 (CH_2), 25.8 (CH_2), 25.6 (CH_2), 24.8 (CH_2), 14.2 (CH_3). **IR** (ATR, neat): 3294, 2956, 2870, 1582, 1448, 1438, 1413, 1266, 1192, 1050, 991, 956, 756 cm^{-1}. **HRMS** (EI): *m/z* calcd for $C_{13}H_{22}N_2S^{\bullet+}$ [M$^{\bullet+}$]: 238.1498; found: 238.1498.

3.2 Preparation of Enamines **4**

General Procedure B (*GP B*).[S10] In a two-neck round bottom flask fitted with a Dean Stark trap a solution of alkylated imidazolidinethione **3** (1 equiv.), aldehyde (1.0-1.2 equiv.), and *p*-toluenesulfonic acid (1 mol%) in dry toluene (1.6 mL/mmol to 2.5 mL/mmol) was refluxed for 2 h or until the disappearance of starting material was detected by TLC. The oil bath was removed, and the reaction was cooled at ambient temperature under an N_2 atmosphere. The solvent was evaporated under vacuum. The crude product was purified by chromatography on either neutral/basic alumina oxide or silica gel. To prevent decomposition, enamines **4** were kept under an inert argon atmosphere (glove box) for storage.

(*E*)-2-((2,2,5,5-Tetramethyl-1-(prop-1-en-1-yl)-2,5-dihydro-1*H*-imidazol-4-yl)thio)acetonitrile (**4a**)

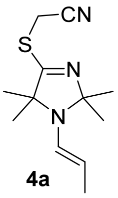

See synthetic procedure described in ref.[S9]

(*E*)-2-((1-(But-1-en-1-yl)-2,2,5,5-tetramethyl-2,5-dihydro-1*H*-imidazol-4-yl)thio)acetonitrile (**4b**) (MJH-I-127): A solution of TIM **3a** (164 mg, 0.831 mmol), butanal (370 µL, 4.11 mmol), 2,6-lutidine (770 µL, 6.61 mmol), acetonitrile (0.70 ml/mmol), and 4Å molecular sieves was stirred and irradiated for 5 h with a Roithner LaserTechnik-H2A1-H420 emitter (420 nm) light placed below the reaction flask. The entire setup was tented in aluminum foil. The solution was decanted, concentrated in vacuo, and immediately stored in air free conditions to mitigate reformation of the starting material. The resulting yellow oil (266 mg) was analyzed by ^{1}H NMR spectroscopy in CD_3CN to be a mixture of enamine **4b** (79.8 mg, 30 w/w%, yield: 38%), 2,6-lutidine (98.4 mg, 37 w/w%), and TIM **3a** (87.8 mg, 33 w/w%).

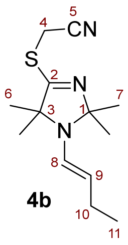

Enamine **4b**: **^{1}H NMR** (400 MHz, CD_3CN): δ = 5.93 (d, *J* = 14.5 Hz, 1 H, 8-H), 4.38 (dt, *J* = 14.5, 6.7 Hz, 1 H, 9-H), 3.88 (s, 2 H, 4-H), 2.03-1.94 (m, 2 H, 10-H), 1.43 (s, 6 H, 7-H), 1.38 (s, 6 H, 6-H), 0.96 (t, *J* = 7.4 Hz, 3 H, 11-H). **^{13}C{^{1}H} NMR** (101 MHz, CD_3CN): δ = 171.3 (C_q, C-2), 126.5 (CH, C-8), 117.9 (CH, C-9), 101.6 (CH, C-9), 90.9 (C_q, C-1), 71.1 (C_q, C-3), 27.7 (CH_3, C-7), 25.7 (CH_3, CH_2, C-6 & C-10, determined by HSQC), 16.6 (CH_3, C-11), 16.2 (CH_2, C-4). **HRMS** (EI): *m/z* calcd for $C_{13}H_{21}N_3S^{\bullet+}$ [$M^{\bullet+}$]: 251.1451; found: 251.1448.

2,6-Lutidine: **^{1}H NMR** (400 MHz, CD_3CN): δ = 7.49 (t, *J* = 7.7 Hz, 1 H), 6.98 (d, *J* = 7.7 Hz, 2 H), 2.43 (s, 6 H). **^{13}C{^{1}H} NMR** (101 MHz, CD_3CN): δ = 158.5, 137.4, 120.8, 24.5.

TIM **3a**: **^{1}H NMR** (400 MHz, CD_3CN): δ = 3.86 (s, 2 H), 1.34 (s, 6 H), 1.28 (s, 6 H). **^{13}C{^{1}H} NMR** (101 MHz, CD_3CN): δ = 172.3, 118.1, 90.1, 71.0, 30.9, 29.0, 17.3.

(*E*)-2-((2,2,5,5-Tetramethyl-1-styryl-2,5-dihydro-1*H*-imidazol-4-yl)thio)acetonitrile (**4c**)

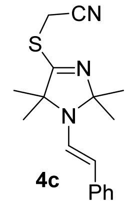

See synthetic procedure described in ref.[S9]

(*E*)-4-(Ethylthio)-2,2,5,5-tetramethyl-1-styryl-2,5-dihydro-1*H*-imidazole (**4d**) (MJH-I-39) was obtained (*GP B*) from TIM **3b** (490 mg, 2.63 mmol) and phenylacetaldehyde (314 mg, 2.61 mmol) in toluene (6.5 mL) as an orange oil. The crude product was purified by flash chromatography (basic alumina oxide, pentane/EtOAc 70:1): **4d** (314 mg, yield: 42%); yellow powder, m.p. 55.8 °C; R_f = 0.69 (silica gel, pentane/EtOAc 20:1).

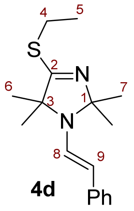

^{1}H NMR (400 MHz, CD_3CN): δ = 7.23–7.14 (m, 4 H, Ph), 6.94 (tt, *J* = 6.9, 1.6 Hz, 1 H, Ph), 6.84 (d, *J* = 14.9 Hz, 1 H, 8-H), 5.40 (d, *J* = 14.8 Hz, 1 H, 9-H), 3.03 (q, *J* = 7.3 Hz, 2 H, 4-H), 1.52 (s, 6 H, 7-H), 1.47 (s, 6 H, 6-H), 1.32 (t, *J* = 7.4 Hz, 3 H, 5-H). **^{13}C{^{1}H} NMR** (101 MHz, CD_3CN): δ = 173.6 (C_q, C-2), 141.3 (C_q, Ph), 129.3 (CH, Ph), 128.6 (CH, C-8), 124.0 (CH, Ph), 123.8 (CH, Ph), 99.0 (CH, C-9), 91.1 (C_q, C-1), 71.9 (C_q, C-3), 27.9 (CH_3, C-7), 26.2 (CH_3, C-6), 24.8 (CH_2, C-4), 14.6 (CH_2, C-5).; **IR** (ATR, neat): 2971, 2925, 1632, 1596, 1446, 1362, 1340, 1328, 1218, 1197, 1168, 1028, 942, 805, 7446, 695 cm^{-1}. **HRMS** (EI): *m*/*z* calcd for $C_{17}H_{24}N_2S^{\bullet+}$ [$M^{\bullet+}$]: 288.1655; found: 288.1648.

(*E*)-13-(Ethylthio)-6-styryl-6,12-diazadispiro[4.1.4⁷.2⁵]tridec-12-ene (4e) (MJH-I-63) was obtained (*GP B*) from TIM **3d** (719 mg, 3.02 mmol) and phenylacetaldehyde (363 mg, 3.02 mmol) in toluene (4.8 mL) as a yellow oil. The crude product was purified by flash chromatography (neutral alumina oxide, pentane/EtOAc 90:1): **4e** (731 mg, yield: 71%); viscous colorless oil; R_f = 0.62 (silica gel, pentane/EtOAc, 9:1).

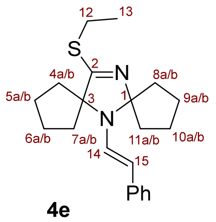

4e

^{1}H NMR (400 MHz, $CDCl_3$): δ = 7.24–7.17 (m, 2 H, Ph), 7.17–7.12 (m, 2 H, Ph), 6.99–6.95 (m, 1 H, Ph), 6.75 (d, *J* = 14.8 Hz, 1 H), 5.22 (d, *J* = 14.8 Hz, 1 H), 3.04 (q, *J* = 7.4 Hz, 2 H), 2.24–2.05 (m, 4 H), 2.00–1.78 (m, 10 H), 1.65–1.55 (m, 2 H), 1.34 (t, *J* = 7.4 Hz, 3 H). **^{1}H NMR** (400 MHz, CD_3CN): δ = 7.22–7.15 (m, 4 H, Ph), 6.97–6.92 (m, 1 H, Ph), 6.82 (d, *J* = 14.9 Hz, 1 H, 14-H), 5.25 (d, *J* = 14.9 Hz, 1 H, 15-H), 3.02 (q, *J* = 7.3 Hz, 2 H, 12-H), 2.27–2.18 (m, 2 H, 8b-H & 11b-H), 2.17-2.07 (m, 2 H, 4b-H & 7b-H), 1.97-1.86 (m, 5a-H, 6a-H, 9a/b-H, 10a/b-H, 4a-H, 7a-H, 8 H), 1.85-1.77 (m, 2 H, 5b-H & 6b-H), 1.57-1.49 (m, 2 H, 8a-H & 11a-H), 1.32 (t, *J* = 7.3 Hz, 3 H, 13-H). **^{13}C{^{1}H} NMR** (101 MHz, CD_3CN): δ = 172.9 (C_q, C-2), 141.0 (C_q, Ph), 129.4 (CH, Ph), 127.9 (CH, C-14), 124.2 (CH, Ph), 124.1 (CH, Ph), 100.3 (C_q, C-1), 100.2 (CH, C-15), 81.0 (C_q, C-3), 36.8 (CH_2, C-8 & C-11), 36.2 (CH_2, C-4 & C-7), 27.4 (CH_2, C-5 & C-6), 25.5 (CH_2, C-9 & C-10), 25.1 (CH_2, C-12), 14.6 (CH_3, C-13). **IR** (ATR, neat): 2955, 2869, 1632, 1595, 1446, 1347, 1318, 1194, 935, 794, 742, 691 cm^{-1}. **HRMS** (EI): *m/z* calcd for $C_{21}H_{28}N_2S^{•+}$ [$M^{•+}$]: 340.1968; found: 340.1961.

(*E*)-4-(Ethylthio)-2,2,5,5-tetramethyl-1-(3-phenylprop-1-en-1-yl)-2,5-dihydro-1*H*-imidazole (4f) (MJH-II-41) was obtained according to *GP B* with the modification that TIM **3b** was used in excess. A solution of **3b** (468 mg, 2.51 mmol), 3-phenylpropionaldehyde (260 µL, 1.95 mmol), and a catalytic amount of *p*-TsOH in toluene (6.3 mL) was heated to reflux for 3 h. After cooling at room temperature under N_2 the toluene was removed in vacuo. Attempts to purify **4f** by chromatography on both silica gel and alumina chromatography led to decomposition. Therefore, the remaining starting material was removed over several hours (approx. 8 h) using a vacuum pump. The enamine was immediately stored under inert conditions in a glovebox. The pale-yellow oil solidified to a waxy pale-yellow solid in the glovebox freezer (–31.5 °C), the compound remained a pale-yellow waxy solid at room temperature. In the presence of air at room temperature, however, the solid gave a yellow oil: **4f** (575 mg, yield: 97%, ^{1}H and ^{13}C NMR spectra indicate a pure product); waxy pale-yellow solid.

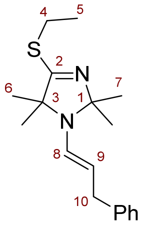

4f

^{1}**H NMR** (400 MHz, $CDCl_3$): δ = 7.28–7.25 (m, 2 H, Ph), 7.22–7.20 (m, 2 H, Ph), 7.17–7.13 (m, 1 H, Ph), 5.95 (d, J = 14.4 Hz, 1 H, 8-H), 4.43 (dt, J = 14.2, 7.0 Hz, 1 H, 9-H), 3.32 (d, J = 7.0 Hz, 2 H, 10-H), 3.02 (q, J = 7.4 Hz, 2 H, 4-H), 1.45 (s, 6 H, 7-H), 1.38 (s, 6 H, 6-H), 1.32 (t, J = 7.4 Hz, 3 H, 5-H). ^{13}**C{^{1}H} NMR** (101 MHz, $CDCl_3$): δ = 173.2 (C_q, C-2), 143.5 (C_q, Ph), 128.24 (CH, Ph), 128.22 (CH, Ph), 127.8 (CH, C-8), 125.6 (CH, Ph), 96.1 (CH, C-9), 89.7 (C_q, C-1), 70.6 (C_q, C-3), 38.2 (CH_2, C-10), 27.5 (CH_3, C-7), 25.7 (CH_3, C-6), 24.3 (CH_2, C-4), 14.1 (CH_3, C-5). **IR** (ATR, neat): 3060, 3024, 2976, 2930, 2872, 2824, 1650, 1601, 1492, 1452, 1363, 1339, 1321, 1270, 1228, 1169, 1158, 1028, 940, 750, 699 cm^{-1}. **HRMS** (EI): m/z calcd for $C_{18}H_{26}N_2S^{\bullet+}$ [$M^{\bullet+}$]: 302.1811; found: 302.1813.

(*E*)-4-(Ethylthio)-2,2,5,5-tetramethyl-1-(3-phenylbut-1-en-1-yl)-2,5-dihydro-1*H*-imidazole (4g) (MJH-II-50) was obtained (*GP B*) with the modification that TIM **3b** was used in excess. A solution of **3b** (328 mg, 1.76 mmol), 3-phenylbutyraldehyde (210 µL, 1.41 mmol), and a catalytic amount of *p*-TsOH in toluene (4.4 mL) was heated to reflux for 2.5 h. After cooling at room temperature under N_2 the toluene was removed in vacuo. Attempts to purify **4g** by chromatography on both silica gel and alumina chromatography led to decomposition. Therefore, the remaining starting material was removed over several hours (approx. 8 h) using a vacuum pump. The enamine was immediately stored under inert conditions in a glovebox. The colorless oil solidified to a white solid in the freezer (–31.5 °C) which turned to a colorless oil at room temperature: **4g** (427 mg, 96% yield, ^{1}H and ^{13}C NMR spectra indicate a pure product).

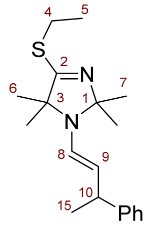

4g

^{1}**H NMR** (400 MHz, $CDCl_3$): δ = 7.29–7.23 (m, 4 H, Ph), 7.16–7.12 (m, 1 H, Ph), 5.88 (d, J = 14.5 Hz, 1 H, 8-H), 4.49 (dd, J = 14.5, 7.2 Hz, 1 H, 9-H), 3.38 (p, J = 6.9 Hz, 1 H, 10-H), 3.01 (q, J = 7.4 Hz, 2 H, 4-H), 1.42 (s, 6 H, 7-H), 1.36 (s, 6 H, 6-H), 1.36–1.30 (m, 6 H, 5-H & 15-H). ^{13}**C{^{1}H} NMR** (101 MHz, $CDCl_3$): δ = 173.2 (C_q, C-2), 148.8 (C_q, Ph), 128.3 (CH, Ph), 127.1 (CH, Ph), 125.8 (CH, C-8), 125.6 (CH, Ph), 103.3 (CH, C-9), 89.7 (C_q, C-1), 70.6 (C_q, C-3), 41.8 (CH, C-10), 27.5 (CH_3, C-7), 25.7 (CH_3, C-6), 24.3 (CH_2, C-4), 23.6 (C-15), 14.1 (CH_3, C-5). **IR** (ATR, neat): 3060, 3026, 2972, 2929, 2869, 1649, 1600, 1492, 1450, 1377, 1362, 1326, 1227, 1176, 1028, 935, 758, 698 cm^{-1}. **HRMS** (EI): m/z calcd for $C_{19}H_{28}N_2S^{\bullet+}$ [$M^{\bullet+}$]: 316.1968; found: 316.1964.

(*E*)-4-(Benzylthio)-2,2,5,5-tetramethyl-1-styryl-2,5-dihydro-1*H*-imidazole (4h) (MJH-I-78) was obtained (*GP B*) from TIM **3c** (890 mg, 3.58 mmol) and phenylacetaldehyde (452 mg, 3.76 mmol) in toluene (9.0 mL) as a colorless oil. The crude product was purified by flash chromatography (neutral alumina oxide, pentane/EtOAc 150:1): **4h** (481 mg, yield: 38%); colorless oil which upon chilling in the fridge (7 °C) crystallized to white spheres, m.p. 85.9 °C; R_f = 0.83 (silica gel, EtOAc).

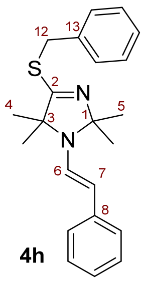

4h

^{1}H NMR (400 MHz, CD_3CN): δ = 7.44–7.38 (m, 2 H, Ph), 7.35–7.24 (m, 3 H, Ph), 7.23–7.14 (m, 4 H, Ph), 6.94 (tt, *J* = 6.8, 1.6 Hz, 1 H, Ph), 6.84 (d, *J* = 14.9 Hz, 1 H, 6-H), 5.41 (d, *J* = 14.8 Hz, 1 H, 7-H), 4.30 (s, 2 H, 12-H), 1.55 (s, 6 H, 5-H), 1.46 (s, 6 H, 4-H). **^{13}C{^{1}H} NMR** (101 MHz, CD_3CN): δ = 173.3 (C_q, C-2), 141.3 (C_q, C-8), 139.0 (C_q, C-13), 129.9 (CH, Ph), 129.4 (CH, Ph), 129.3 (CH, Ph), 128.6 (CH, C-6), 128.2 (CH, Ph), 124.1 (CH, Ph), 123.9 (CH, Ph), 99.1 (CH, C-7), 91.2 (C_q, C-1), 71.9 (C_q, C-3), 34.3 (CH_2, C-12), 28.0 (CH_3, C-5), 26.1 (CH_3, C-4). **IR** (ATR, neat): 3056, 2972, 2925, 1633, 1596, 1495, 1452, 1443, 1380, 1355, 1342, 1325, 1303, 1196, 1182, 1170, 1046, 1027, 1019, 941, 804, 775, 751, 745, 708, 692 cm^{-1}. **HRMS** (EI): *m/z* calcd for $C_{22}H_{26}N_2S^{\bullet+}$ [$M^{\bullet+}$]: 350.1811; found: 350.1811.

(*E*)-2,2,3,5,5-Pentamethyl-1-styrylimidazolidin-4-one (4i) (MJH-II-44) was obtained (*GP B*) from ITO **2c** (399 mg, 2.55 mmol) and phenylacetaldehyde (329 mg, 2.74 mmol) in toluene (6.3 mL). After removal of the volatiles in vacuo, the yellow oily residue was diluted in Et_2O in which a white solid started to precipitate. The solution was cooled with an ice bath. The precipitated solids were isolated by filtration and washed with cold Et_2O: **4i** (216 mg, yield: 33%); tan powder, m.p. 145.8 °C; R_f = 0.38 (silica gel, pentane/EtOAc 1:1).

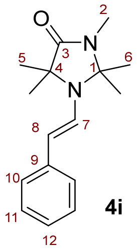

4i

^{1}H NMR (400 MHz, $CDCl_3$): δ = 7.24–7.22 (m, 2 H, 11-H), 7.20–7.17 (m, 2 H, 10-H), 7.05–7.00 (m, 1 H, 12-H), 6.73 (d, *J* = 14.9 Hz, 1 H, 7-H), 5.49 (d, *J* = 14.9 Hz, 1 H, 8-H), 2.91 (s, 3 H, 2-H), 1.57 (s, 6 H, 6-H), 1.52 (s, 6 H, 5-H). **^{13}C{^{1}H} NMR** (101 MHz, CD_3CN): δ = 173.4 (C_q, C-3), 139.6 (C_q, C-9), 128.8 (CH, C-11), 127.1 (CH, C-7), 124.0 (CH, C-12), 123.6 (CH, C-10), 100.5 (CH, C-8), 77.6 (C_q, C-1, determined by HMBC), 61.3 (C_q, C-4), 25.7 (CH_3, C-6), 25.1 (CH_3, C-2), 24.6 (CH_3, C-5). **IR** (ATR, neat): 2985, 1696, 1637, 1593, 1476, 1402, 1349, 1230, 1206, 1190, 1153, 1126, 1036, 1012, 945, 804, 756, 700, 686 cm^{-1}. **HRMS** (EI): *m/z* calcd for $C_{16}H_{22}N_2O^{\bullet+}$ [$M^{\bullet+}$]: 258.1727; found: 258.1733.

(*E*)-2,2,3,5,5-Pentamethyl-1-styrylimidazolidine-4-thione (**4j**) (MJH-I-172//MJH-II-8) was obtained (*GP B*) from ITO **2d** (173 mg, 1.00 mmol) and phenylacetaldehyde (140 mg, 1.16 mmol) in toluene (5 mL) as an orange oil. The crude product was purified by flash chromatography (sample loaded on silica gel with a minimal amount of CH_2Cl_2, pentane/EtOAc 9:1): **4j** (122 mg, yield: 44%); white powder, m.p. 180.1 °C; R_f = 0.70 (silica gel, EtOAc).

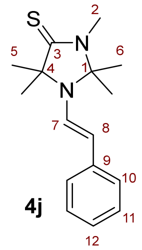

4j

^{1}H NMR (400 MHz, CD_3CN): δ = 7.27–7.22 (m, 2 H, 10-H), 7.21–7.16 (m, 2 H, 11-H), 7.08–7.01 (m, 1 H, 12-H), 6.74 (d, *J* = 14.9 Hz, 1 H, 7-H), 5.52 (d, *J* = 14.9 Hz, 1 H, 8-H), 3.29 (s, 3 H, 2-H), 1.66 (s, 6 H, 5-H), 1.66 (s, 6 H, 6-H). **^{13}C{^{1}H} NMR** (101 MHz, CD_3CN): δ = 202.1 (C_q, C-3), 139.4 (C_q, C-9), 128.8 (CH, C-10), 126.9 (CH, C-7), 124.3 (CH, C-12), 123.8 (CH, C-10), 101.2 (CH, C-8), 84.2 (C_q, C-1), 72.2 (C_q, C-4), 31.5 (CH_3, C-2), 28.2 (CH_3, C-5), 25.2 (CH_3, C-6). **IR** (ATR, neat): 3063, 3024, 2981, 2927, 1638, 1594, 1496, 1450, 1348, 1281, 1224, 1199, 1105, 946, 812, 751, 696 cm^{-1}. **HRMS** (EI): *m/z* calcd for $C_{16}H_{22}N_2S^{\bullet+}$ [$M^{\bullet+}$]: 274.1498; found: 274.1499.

4. Reactions of Enamines **4** with Benzhydrylium Tetrafluoroborates **5**

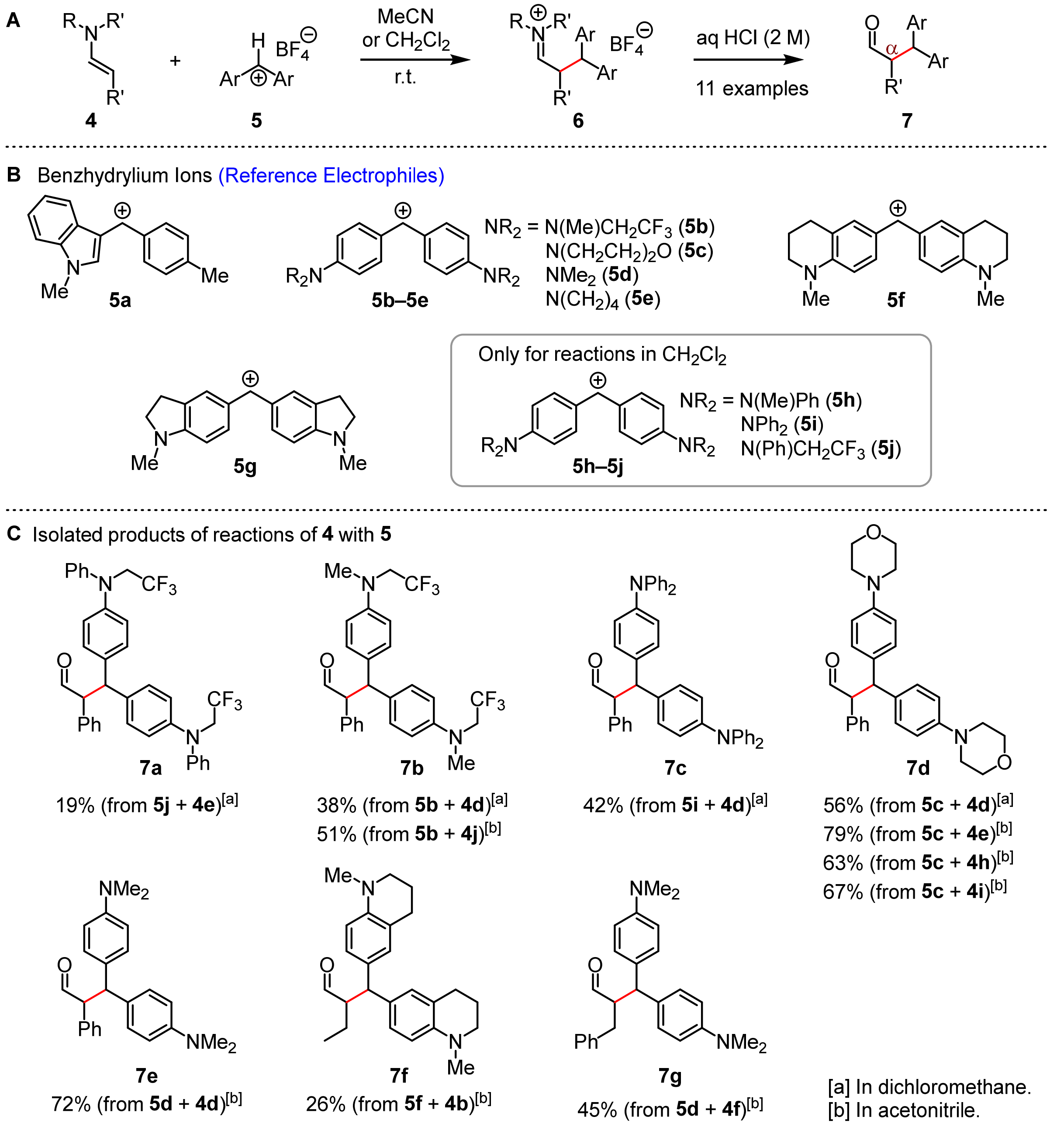

Figure S2. (A) Enamines **4** reacted with benzhydrylium tetrafluoroborates **5** to furnish iminium salts **6** which were hydrolyzed to α-alkylated aldehydes **7**. (B) Structures of benzhydrylium ions **5a**–**5j** (counterion: tetrafluoroborate) used in this study. (C) Structures of isolated aldehydes. Yields are given in parentheses for individual **4** + **5** couples. If several reactions of a certain benzhydrylium tetrafluoroborate with different enamines gave the same aldehyde, full spectral characterization was only carried out once. However, ^{1}H NMR spectra are shown for each investigated **4** + **5** pair.

1-(3,3-Bis(4-morpholinophenyl)-2-phenylpropylidene)-4-(ethylthio)-2,2,5,5-tetramethyl-2,5-dihydro-1*H*-imidazol-1-ium tetrafluoroborate 6a (MJH-III-5): The benzhydrylium tetrafluoroborate **5c** (9.7 mg) and the enamine **4d** (6.6 mg, 1 equiv.) were mixed in CD_3CN in an NMR tube under a dry argon atmosphere. After 10 min, the mixture was analyzed by NMR spectroscopy (Figure S3-S6): **6a** (*E*/*Z* = 1:1.1), quantitative.

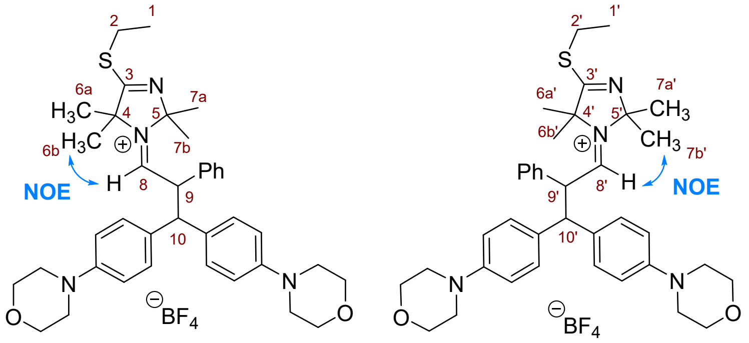

(*Z*)-**6a** (*E*)-**6a**

^{1}H NMR (400 MHz, CD_3CN): δ = 8.87 (d, *J* = 10.8 Hz, 1 H, 8'-H), 8.79 (d, *J* = 10.8 Hz, 1 H, 8-H), 7.54–7.51 (m, 4 H), 7.38–7.34 (m, 8 H), 7.32–7.29 (m, 6 H), 6.92 (d, *J* = 8.6 Hz, 4 H), 6.82 (d, *J* = 8.6 Hz, 4 H), 4.99–4.92 (m, 2 H, 9-H and 9'-H), 4.70–4.66 (m, 2 H, 10-H and 10'-H), 3.76–3.72 (m, 16 H, morpholino OCH_2), 3.08–2.99 (m, 20 H, morpholino NCH_2, 2-H, and 2'-H), 1.65 (s, 3 H, 7a-H or 7b-H), 1.63 (s, 3 H, 6a'-H or 6b'-H), 1.58 (s, 3 H, 7a'-H or 7b'-H), 1.55 (s, 3 H, 7a-H or 7b-H), 1.51 (s, 3 H, 6a-H or 6b-H), 1.50 (s, 3 H, 6a'-H or 6b'-H), 1.41 (s, 3 H, 7a'-H or 7b'-H), 1.32 (s, 3 H, 6a-H or 6b-H), 1.27 (t, *J* = 7.4 Hz, 3 H, 1-H, superimposed with 1'-H), 1.27 (t, *J* = 7.4 Hz, 3 H, 1'-H, superimposed with 1-H). **^{13}C{^{1}H} NMR** (101 MHz, CD_3CN): δ = 177.1 (CH, C-8'), 176.7 (CH, C-8), 172.8 (C_q, C-3 or C-3'), 171.0 (C_q, C-3 or C-3'), 152.2* (C_q), 150.5* (C_q), 135.5 (C_q), 135.5 (C_q), 132.4* (C_q), 131.4* (C_q), 130.7 (CH), 130.7 (CH), 130.4 (CH), 130.4 (CH), 130.2 (CH), 130.1 (CH), 129.9 (CH), 129.8 (CH), 129.8 (CH), 117.0 (CH), 116.5 (CH), 104.4 (C_q, C-5'), 101.6 (C_q, C-5), 82.3 (C_q, C-4), 78.7 (C_q, C-4'), 67.2 (CH_2), 56.7 (CH, C-10 or C-10'), 56.5 (CH, C-10 or C-10'), 53.8 (CH, C-9 or C-9'), 53.3 (CH, C-9 or C-9'), 50.1 (CH_2), 49.9 (CH_2), 29.8 (CH_3, C-7a' or C-7b'), 29.3 (CH_3, C-7a' or C-7b'), 28.7 (CH_3, C-6a or C-6b), 28.6 (CH_3, C-7a or C-7b), 28.5 (CH_3, C-7a or C-7b), 28.0 (CH_3, C-6a or C-6b), 26.8 (CH_3, C-6a' or C-6b'), 26.7 (CH_3, C-6a' or C-6b'), 26.0 (CH_3, C-2 or C-2'), 25.7 (CH_2, C-2 or C-2'), 14.2 (CH_3, C-1 or C-1'), 14.2 (CH_3, C-1 or C-1'); asterisks (*) mark resonances that were only detected in the HMBC spectrum.

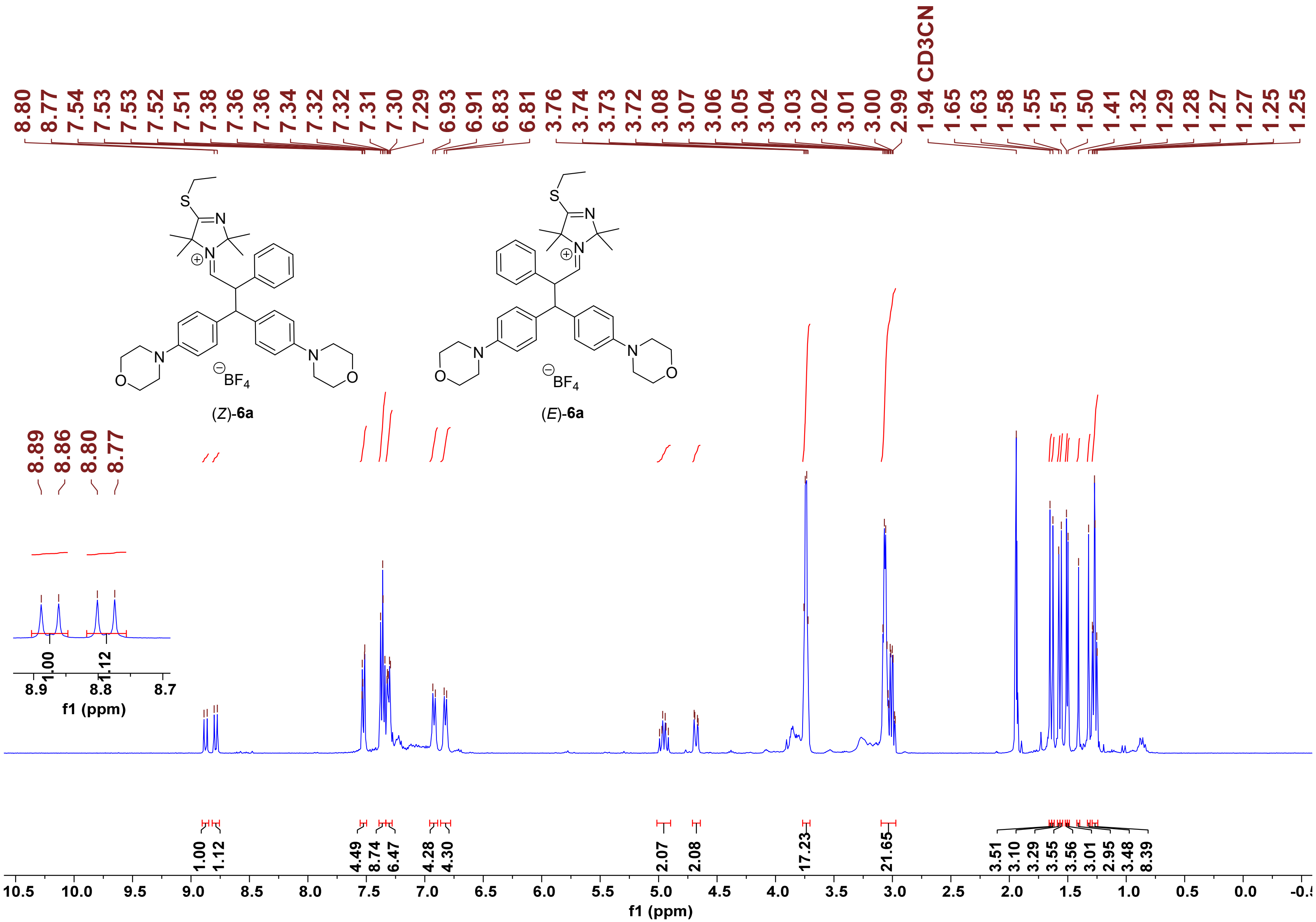

Figure S3. ^{1}H NMR spectrum (CD_3CN, 400 MHz) of **6a** (*E/Z* = 1:1.1).

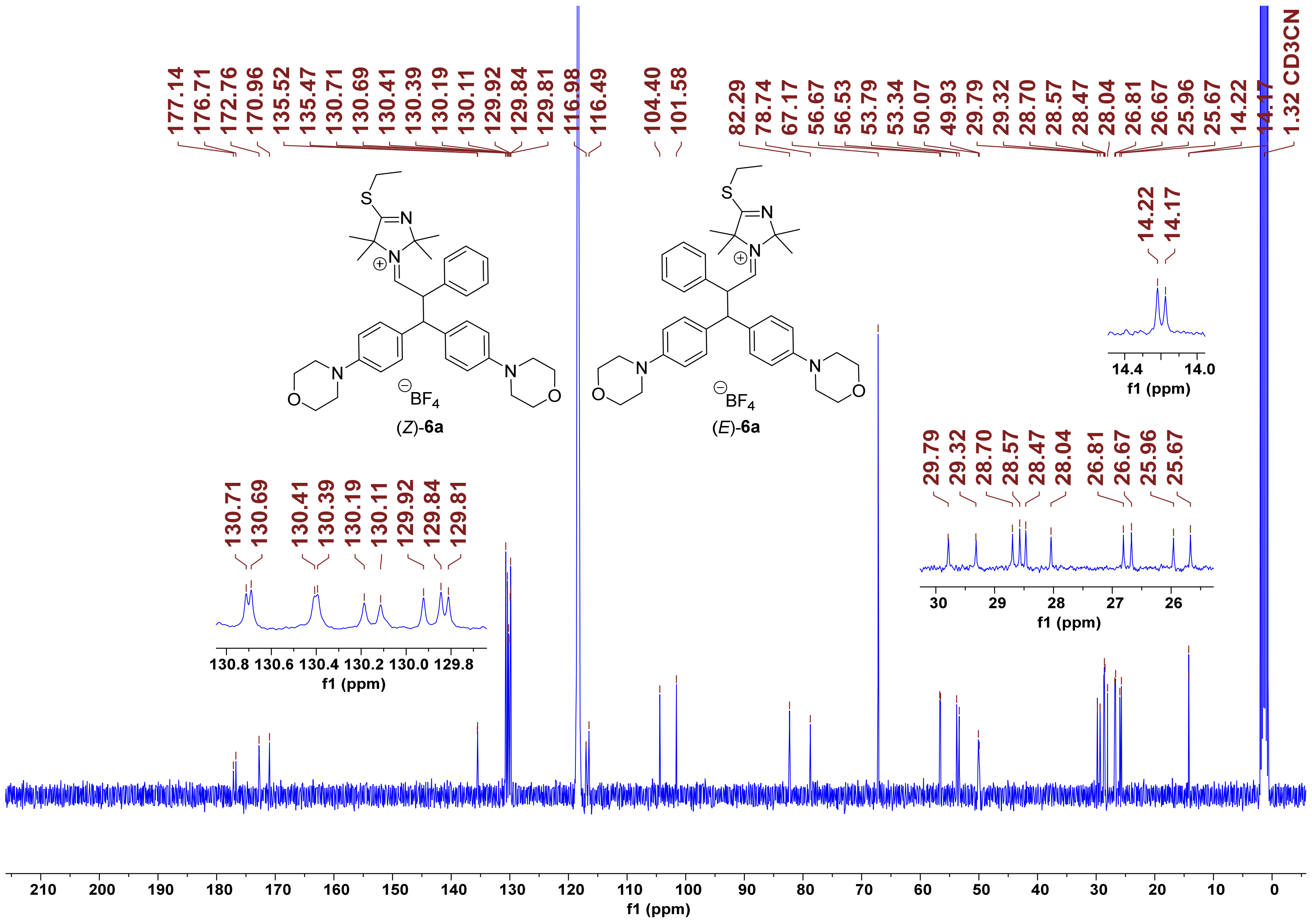

Figure S4. $^{13}C\{^{1}H\}$ NMR spectrum (CD_3CN, 101 MHz) of **6a** (*E/Z* = 1:1.1).

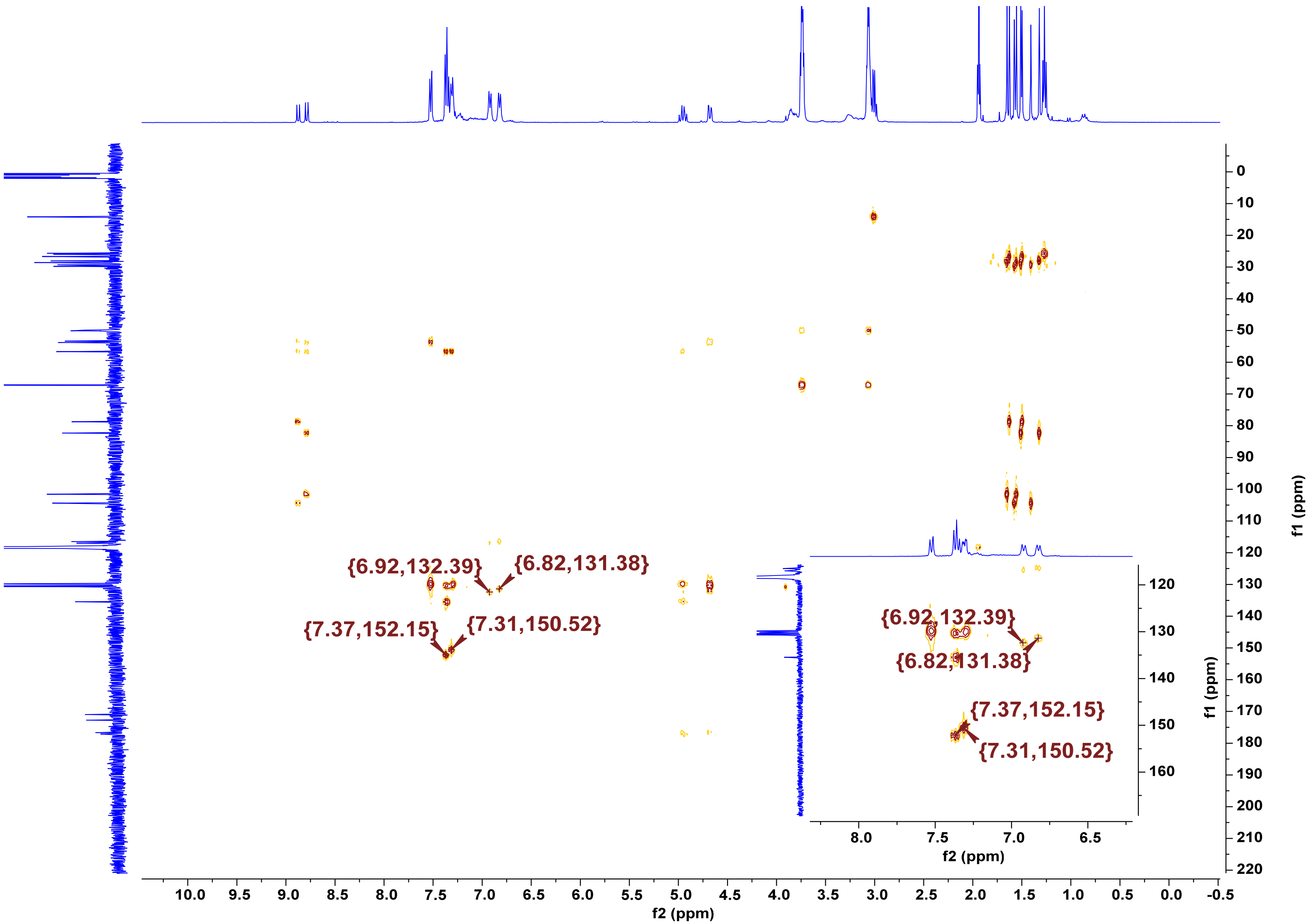

Figure S5. 400 MHz HMBC spectrum of **6a** (*E/Z* = 1:1.1). Labeled points show quaternary C atoms that were not detected in the $^{13}C\{^{1}H\}$ NMR spectrum (Figure S4).

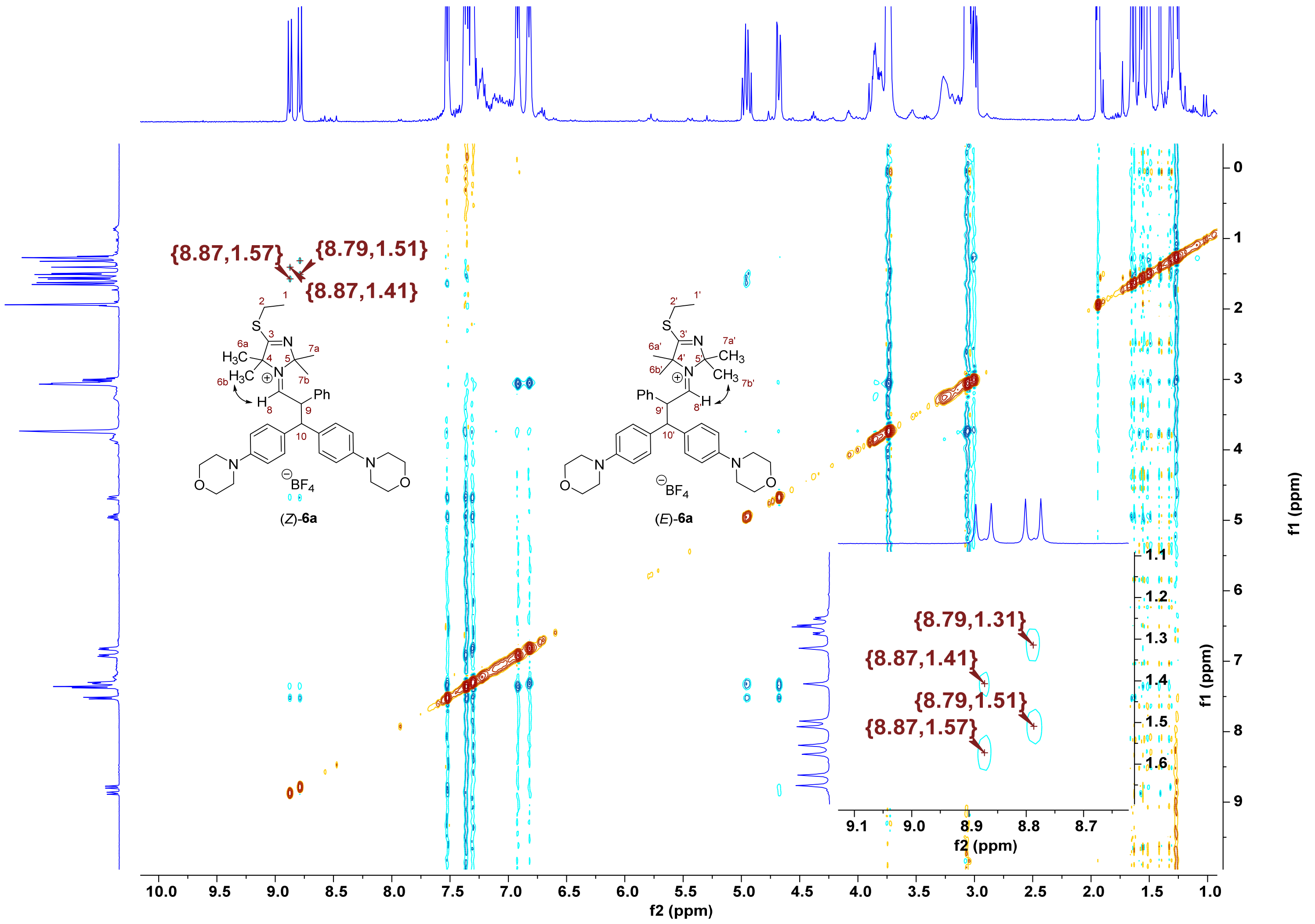

Figure S6. 800 MHz NOESY spectrum of **6a** (*E*/*Z* = 1:1.1). Labeled points represent correlations of H-8 (δ = 8.79) with H-6a/H-6b (δ = 1.31/1.51) and of H-8′ (δ = 8.87) with H-7a′/H-7b′ (δ = 1.41/1.57).

2-Phenyl-3,3-bis(4-(phenyl(2,2,2-trifluoroethyl)amino)phenyl)propanal

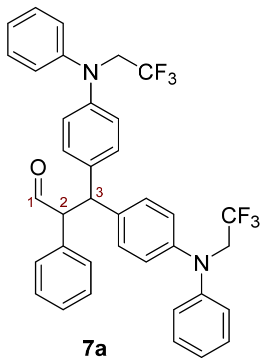

7a

7a (MJH-II-21//MJH-II-56): To a solution of **5j** (122 mg, 0.203 mmol) in CH_2Cl_2 (12 mL) was added **4e** (85.3 mg, 0.250 mmol) in CH_2Cl_2 (5.0 mL) dropwise at room temperature, at which point the blue solution turned purple. After 40 min, 2 M hydrochloric acid (3 mL) was added. The mixture was left to stir at room temperature for 30 min. The solution was then diluted in CH_2Cl_2 (10 mL) and washed with aq. sat. $NaHCO_3$ solution. The organic layer was separated, and the aqueous layer was extracted with CH_2Cl_2 (3 × 20 mL). The combined organic layers were dried over $MgSO_4$, and the solvent was evaporated. The crude dark purple oil was purified by flash chromatography (silica gel, pentane/CH_2Cl_2 = 3:2). The isolated sample was further purified by flash chromatography (silica gel, pentane/ Et_2O = 6:1) to give a yellow oil (26.1 mg): **7a** (25.0 mg, yield: 19%), contaminated with traces of 2-phenylacetaldehyde (yield based on the integrals in the ^{1}H NMR spectrum); R_f = 0.27 (silica gel, pentane/CH_2Cl_2 = 3:2).

^{1}H NMR (400 MHz, $CDCl_3$): δ = 9.70 (d, J = 3.2 Hz, 1 H, 1-H), 7.40–7.29 (m, 5 H, ArH), 7.24–7.20 (m, 4 H, ArH), 7.17–7.15 (m, 2 H, ArH), 7.08 (t, J = 7.4 Hz, 1 H, ArH), 7.04–6.96 (m, 5 H, ArH), 6.90 (d, J = 8.2 Hz, 2 H, ArH), 6.84 (d, J = 8.0 Hz, 2 H, ArH), 6.73 (d, J = 8.1 Hz, 2 H, ArH), 4.67 (d, J = 11.9 Hz, 1 H, 3-H), 4.39 (dd, J = 11.9, 3.3 Hz, 1 H, 2-H), 4.27 (q, $J_{H,F}$ = 8.7 Hz, 2 H, NCH_2), 4.17 (q, $J_{H,F}$ = 8.7 Hz, 2 H, NCH_2). **$^{13}C\{^1H\}$ NMR** (101 MHz, $CDCl_3$): δ = 199.1 (CH, C-1), 147.4 (C_q), 147.1 (C_q), 146.6 (C_q), 145.7 (C_q), 136.2 (C_q), 135.8 (C_q), 134.6 (C_q), 129.8 (CH), 129.6 (CH), 129.5 (CH), 129.4 (CH), 129.2 (CH), 129.0 (CH), 127.7 (CH), 125.3 (C_q, q, $J_{C,F}$ = 283.0 Hz, CF_3), 125.2 (C_q, q, $J_{C,F}$ = 282.8 Hz, CF_3), 123.6 (CH), 122.7 (CH), 122.6 (CH), 121.4 (CH), 121.1 (CH), 120.5 (CH), 63.8 (CH, C-2), 54.1 (CH_2, q, $J_{C,F}$ = 33.2 Hz, NCH_2), 54.0 (CH_2, q, $J_{C,F}$ = 33.2 Hz, NCH_2), 50.9 (CH, C-3). **^{19}F NMR** (377 MHz, $CDCl_3$): δ = –69.51 (t, $J_{F,H}$ = 8.7 Hz), –69.61 (t, $J_{F,H}$ = 8.7 Hz). **HRMS** (ESI): m/z calcd for $C_{37}H_{29}F_6N_2O^-$ [M^-]: 631.2190; found: 631.2184.

3,3-Bis(4-(methyl(2,2,2-trifluoroethyl)amino)phenyl)-2-phenylpropanal 7b

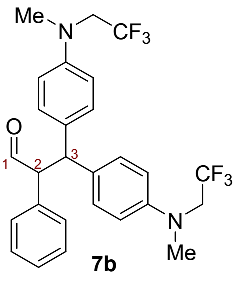

7b

(MJH-I-43): To a solution of **5b** (133 mg, 0.279 mmol) in CH_2Cl_2 (5.6 mL) was added **4d** (80.5 mg, 0.279 mmol) in CH_2Cl_2 (5.6 mL) dropwise at room temperature. The reaction was stirred at room temperature for 1 h. Most of the solvent was evaporated in vacuo and hydrochloric acid (2 M) was added to the oily residual. The mixture was left to stir at room temperature for 30 min. The solution was neutralized using dilute NaOH (2 M). The aqueous layer was washed with CH_2Cl_2 (3 × 20 mL), dried over $MgSO_4$, and the solvents were evaporated in vacuo. The crude

material was purified by flash chromatography (silica gel, pentane/EtOAc = 8:1): **7b** (53.6 mg, yield: 38%), yellow powder, m.p. 57.4 °C; R_f = 0.83 (silica gel, CH_2Cl_2/Et_2O = 3:1).

^{1}H NMR (600 MHz, $CDCl_3$): δ = 9.64 (d, *J* = 3.8 Hz, 1 H, 1-H), 7.28–7.15 (m, 7 H, ArH), 6.96 (d, *J* = 8.8 Hz, 2 H, ArH), 6.73 (d, *J* = 8.7 Hz, 2 H, ArH), 6.54 (d, *J* = 8.7 Hz, 2 H, ArH), 4.63 (d, *J* = 11.9 Hz, 1 H, 3-H), 4.38 (dd, *J* = 12.0, 3.8 Hz, 1 H, 2-H), 3.80 (q, $J_{H,F}$ = 9.0 Hz, 2 H, CH_2CF_3), 3.70 (q, $J_{H,F}$ = 9.0 Hz, 2 H, CH_2CF_3), 3.00 (s, 3 H, CH_3), 2.91 (s, 3 H, CH_3). **^{13}C{^{1}H} NMR** (151 MHz, $CDCl_3$): δ = 199.5 (CH, C-1), 147.6 (C_q), 147.1 (C_q), 135.1 (C_q), 132.3 (C_q), 131.9 (C_q), 129.5 (CH), 129.00 (CH), 128.95 (CH), 127.6 (CH), 125.7 (C_q, q, $J_{C,F}$ = 282.8 Hz, CF_3), 125.6 (C_q, q, $J_{C,F}$ = 282.7 Hz, CF_3), 113.2 (CH), 112.9 (CH), 63.8 (CH, C-2), 54.7 (CH_2, q, $J_{C,F}$ = 32.4 Hz, NCH_2), 54.6 (CH_2, q, $J_{C,F}$ = 32.6 Hz, NCH_2), 50.2 (CH, C-3), 39.3 (CH_3, NCH_3), 39.2 (CH_3, NCH_3). **^{19}F NMR** (376 MHz, $CDCl_3$): δ = –70.59 (t, $J_{F,H}$ = 8.9 Hz), –70.65 (t, $J_{F,H}$ = 8.9 Hz). **IR** (ATR, neat): 2917, 1720, 1612, 1516, 1371, 1262, 1137, 1092, 985, 809, 750, 698, 660 cm^{-1}. **HRMS** (EI): *m/z* calcd for $C_{27}H_{26}F_6N_2O^{•+}$ [M$^{•+}$]: 508.1944; found: 508.1944.

3,3-Bis(4-(methyl(2,2,2-trifluoroethyl)amino)phenyl)-2-phenylpropanal 7b (MJH-I-189): To a solution of **5b** (97.3 mg, 0.204 mmol) in CH_3CN (4.0 mL) was added **4j** (55.0 mg, 0.200 mmol) in CH_3CN (4.0 mL) in one portion at room temperature. The blue reaction mixture was stirred at room temperature for 1 h and 2 M hydrochloric acid (3 mL) was added. The mixture was left to stir at room temperature for 30 min. Then, aq. NH_4OH (3 mL) was added and the color of the solution changed from blue to colorless. The aqueous layer was extracted with CH_2Cl_2 (3 x 20 mL), dried over $MgSO_4$, and the solvents were evaporated in vacuo. The crude red-orange oil was purified by flash chromatography (silica gel, pentane/Et_2O = 8:1): **7b** (51.8 mg, yield: 51%), light-yellow powder.

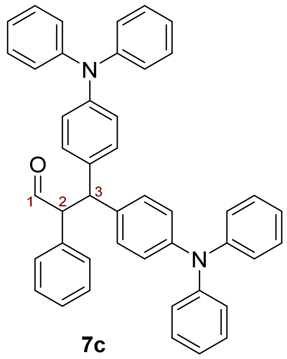

7c

3,3-Bis(4-diphenylaminophenyl)-2-phenylpropanal 7c (MJH-I-88): To a solution of **5i** (147 mg, 0.250 mmol) in CH_2Cl_2 (5.0 mL) was added **4d** (72.4 mg, 0.251 mmol) in CH_2Cl_2 (5.0 mL) dropwise at room temperature. The reaction was stirred at room temperature for 1 h. Most of the solvent was evaporated in vacuo and to the oily residue was added dilute HCl (2 M). The mixture was left to stir at room temperature for 30 min and then neutralized by addition of dilute aq. NaOH (2 M). The aqueous layer was extracted with CH_2Cl_2 (3 × 20 mL), dried over $MgSO_4$, and the solvents were evaporated in vacuo. The crude blue oil was purified by flash chromatography (silica gel, pentane/EtOAc = 8:1). The fractions were collected, and then further purified by flash chromatography (silica gel, pentane/CH_2Cl_2 = 1:1): **7c** (65.5 mg, yield: 42

%), blue solid; R_f = 0.86 (silica gel, CH_2Cl_2/Et_2O = 3:1); ^{1}H and ^{13}C NMR spectral data agree with those previously reported.[S11]

^{1}H NMR (400 MHz, $CDCl_3$): δ = 9.67 (d, *J* = 3.2 Hz, 1 H, 1-H), 7.37 (d, *J* = 8.6 Hz, 2 H), 7.30–7.20 (m, 13 H), 7.12 (d, *J* = 8.6 Hz, 2 H), 7.06–6.97 (m, 10 H), 6.87–6.85 (m, 4 H), 6.76 (d, *J* = 8.5 Hz, 2 H), 4.76 (d, *J* = 12.3 Hz, 1 H, 3-H), 4.58 (dd, *J* = 12.3, 3.3 Hz, 1 H, 2-H); **^{13}C{^{1}H} NMR** (101 MHz, $CDCl_3$): δ = 199.4, 147.8, 146.6, 146.0, 136.1, 136.0, 134.9, 129.7, 129.4, 129.22, 129.17, 129.0, 128.9, 127.6, 124.5, 124.1, 124.0, 123.9, 123.0, 122.7, 63.9, 51.3; **IR** (ATR, neat): 3029, 1720, 1586, 1504, 1487, 1314, 1272, 751, 693 cm^{-1}.

3,3-Bis(4-morpholinophenyl)-2-phenylpropanal 7d (MJH-I-181): To a solution of **5c** (81.2 mg, 0.191 mmol) in CH_3CN (3.8 mL) was added **4e** (65.6 mg, 0.193 mmol) in CH_3CN (4 mL) in one portion at room temperature. The color of the solution turned dark purple as the blue solution stirred at room temperature. After 20 min, dilute hydrochloric acid (2 M, 3 mL) was added. The mixture was left to stir at room temperature for 20 min. The solution was then diluted in CH_2Cl_2 (10 mL) and washed with aq saturated $NaHCO_3$ solution. The organic layer was separated, the aqueous layer was extracted with CH_2Cl_2 (3 × 20 mL). The combined organic layers were dried over $MgSO_4$, and the solvent was evaporated in vacuo. The crude dark purple oil was purified by flash chromatography (silica gel, pentane/EtOAc = 2:1 → pure EtOAc): **7d** (69.0 mg, yield: 79%), white powder, m.p. 87.4 °C; R_f = 0.34 (silica gel, CH_2Cl_2/Et_2O = 3:1). The ^{1}H NMR spectral data agree with those reported in ref.[S9]

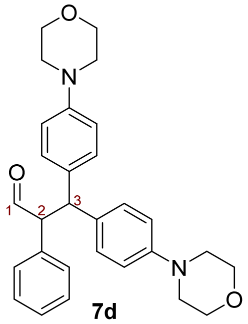

7d

^{1}H NMR (400 MHz, $CDCl_3$): δ = 9.64 (d, *J* = 3.7 Hz, 1 H, 1-H), 7.28–7.14 (m, 7 H), 6.98 (d, *J* = 8.7 Hz, 2 H), 6.84 (d, *J* = 8.7 Hz, 2 H), 6.64 (d, *J* = 8.7 Hz, 2 H), 4.65 (d, *J* = 12.0 Hz, 1 H, 3-H), 4.39 (dd, *J* = 11.9, 3.7 Hz, 1 H, 2-H), 3.84-3.82 (m, 4 H), 3.78-3.76 (m, 4 H), 3.12-3.09 (m, 4 H), 3.02–2.99 (m, 4 H).

3,3-Bis(4-morpholinophenyl)-2-phenylpropanal 7d (MJH-I-45): To a solution of **5c** (110 mg, 0.259 mmol) in CH_2Cl_2 (5.2 mL) was added **4d** (75.5 mg, 0.262 mmol) in CH_2Cl_2 (5 mL) dropwise at room temperature. The reaction was stirred at room temperature for 1 h. Most of the solvent was evaporated in vacuo and to the resulting oil was added dilute aq. HCl (2 M). The mixture was left to stir at room temperature for 30 min. The solution was neutralized using dilute NaOH (2M). The aqueous layer was washed with CH_2Cl_2 (3 × 20 mL), dried over $MgSO_4$, and the solvent was evaporated in vacuo. The crude dark purple oil was purified by flash chromatography (silica gel, pentane/EtOAc = 1:1): **7d** (65.6 mg, yield: 56%), tan powder.

3,3-Bis(4-morpholinophenyl)-2-phenylpropanal 7d (MJH-I-182): To a solution of **5c** (78.2 mg, 0.184 mmol) in CH_3CN (3.7 mL) was added **4h** (63.2 mg, 0.180 mmol) in CH_3CN (4 mL) in one portion at room temperature. The color of the solution turned dark purple as the blue solution stirred at room temperature. After 20 min, dilute aq HCl (2 M, 3 mL) was added. The mixture was left to stir at room temperature for 30 min. The solution was then diluted in CH_2Cl_2 (10 mL) and washed with aq saturated $NaHCO_3$ solution. The organic layer was separated, and the aqueous layer was extracted with CH_2Cl_2 (3 × 20 mL). The combined organic layers were dried over $MgSO_4$, and the solvent was evaporated in vacuo. The crude dark purple oil was purified by two-fold flash chromatography (silica gel, CH_2Cl_2/Et_2O = 6:1): **7d** (51.8 mg, yield: 63%), white powder.

3,3-Bis(4-morpholinophenyl)-2-phenylpropanal 7d (MJH-II-49): To a solution of **5c** (100 mg, 0.236 mmol) in CH_3CN (4.8 mL) was added **4i** (58.2 mg, 0.225 mmol) in CH_3CN (4 mL) in one portion at room temperature. After 2 h, dilute aq HCl (2 M, 3 mL) was added and the dark blue solution turned dark purple. The mixture was left to stir at room temperature for 30 min. The solution was then diluted in CH_2Cl_2 (10 mL) and washed with aq saturated $NaHCO_3$ solution. The organic layer was separated, the aqueous layer was extracted with CH_2Cl_2 (3 × 20 mL), the combined organic layers were dried over $MgSO_4$, and the solvent was evaporated in vacuo. The crude dark purple oil was purified by flash chromatography (silica gel, CH_2Cl_2/Et_2O = 6:1): **7d** (68.9 mg, yield: 67%), white powder.

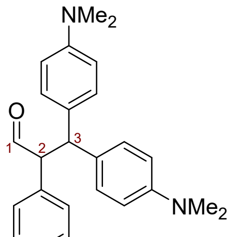

7e

3,3-Bis(4-(dimethylamino)phenyl)-2-phenylpropanal 7e (MJH-I-153): To a solution of **5d** (60.8 mg, 0.179 mmol) in CH_3CN (1.8 mL) was added **4d** (53.5 mg, 0.185 mmol) in CH_3CN (4 mL) dropwise at room temperature. The reaction was stirred at room temperature for 2 h. Most of the solvent was evaporated in vacuo, and to the oily residual was added dilute aq. HCl (2 M, 3 mL). The mixture was left to stir at room temperature for 30 min. The solution was neutralized by adding dilute NaOH (2 M) and further diluted in CH_2Cl_2 (10 mL). The aqueous layer was extracted with CH_2Cl_2 (3 × 20 mL), dried over $MgSO_4$, and the solvent was evaporated in vacuo. The crude dark blue oil was purified by flash chromatography (silica gel, pentane/EtOAc = 5:1): **7e** (47.8 mg, yield: 72%), yellow solid. ^{1}H and ^{13}C NMR spectral data agree with those previously reported.[S12]

^{1}H NMR (400 MHz, $CDCl_3$): δ = 9.63 (d, *J* = 4.0 Hz, 1 H, 1-H), 7.25–7.14 (m, 7 H), 6.95 (d, *J* = 8.7 Hz, 2 H), 6.67 (d, *J* = 8.3 Hz, 2 H), 6.49 (d, *J* = 8.2 Hz, 2 H), 4.60 (d, *J* = 11.9 Hz, 1 H, 3-H), 4.37 (dd, *J* = 12.0, 4.0 Hz, 1 H, 2-H), 2.89 (s, 6 H), 2.80 (s, 6 H). **^{13}C{^{1}H} NMR** (101 MHz, $CDCl_3$): δ = 200.0, 149.5,

148.9, 135.6, 130.7, 130.4, 129.4, 128.91, 128.86, 128.77, 127.4, 113.1, 112.7, 63.8, 50.3, 40.8, 40.7.

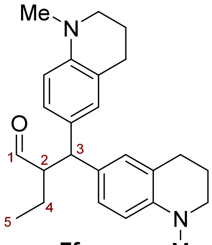

7f

2-(Bis(1-methyl-1,2,3,4-tetrahydroquinolin-6-yl)methyl)butanal 7f (MJH-II-22): In a separate flask, 298 mg of mixture containing **4b**, 2,6-lutidine, and the secondary amine TIM **3a** was weighed, in which 95.3 mg of this mixture was estimated to be **4b** based on the relative integral heights the ^{1}H NMR spectrum of the sample.

To a solution of **5f** (90.7 mg, 0.231 mmol) in CH_3CN (5 mL) was added the solution which contained **4b** (95.3 mg, 0.379 mmol) in CH_3CN (4 mL) in one portion at room temperature. The color of the reaction mixture turned from dark blue to dark green. After 1 h, dilute aq. HCl (2 M, 3 mL) was added. The mixture was left to stir at room temperature for 30 min. The solution was diluted in CH_2Cl_2 (10 mL), and then washed with aq. saturated $NaHCO_3$ solution. The organic layer was separated, and the aqueous layer was extracted with CH_2Cl_2 (3 × 20 mL). The combined organic layers were dried over $MgSO_4$, and the solvent was evaporated in vacuo. The crude material was purified by flash chromatography (silica gel, loaded with CH_2Cl_2 then eluted with pentane/Et_2O = 5:1): **7f** (22.7 mg, yield: 26%), colorless oil; R_f = 0.67 (silica gel, CH_2Cl_2/Et_2O = 4:1).

^{1}H NMR (400 MHz, $CDCl_3$): δ = 9.44 (d, *J* = 4.6 Hz, 1 H, 1-H), 6.95 (d, *J* = 2.3 Hz, 1 H), 6.93 (d, *J* = 2.3 Hz, 1 H), 6.80 (s, 1 H), 6.79 (s, 1 H), 6.51 (d, *J* = 8.4 Hz, 1 H), 6.47 (d, *J* = 8.4 Hz, 1 H), 3.82 (d, *J* = 11.2 Hz, 1 H, 3-H), 3.16–3.12 (m, 4 H), 2.97–2.89 (m, 1 H, 2-H), 2.84 (s, 3 H), 2.81 (s, 3 H), 2.74–2.67 (m, 4H), 1.98–1.89 (m, 4 H), 1.58–1.46 (m, 2 H, 4-H), 0.85 (t, *J* = 7.5 Hz, 3 H, 5-H). **^{13}C{^{1}H} NMR** (101 MHz, $CDCl_3$) δ = 205.5 (CH, C-1), 145.44 (C_q), 145.38 (C_q), 130.9 (C_q), 130.7 (C_q), 128.7 (CH), 128.6 (CH), 126.4 (CH), 126.3 (CH), 123.11 (CH), 123.08 (CH), 111.23 (CH), 111.16 (CH), 57.9 (CH, C-2), 51.39 (CH_2), 51.35 (CH_2), 50.7 (CH, C-3), 39.3 (CH_3), 39.2 (CH_3), 28.01 (CH_2), 27.96 (CH_2), 22.60 (CH_2), 22.57 (CH_2), 21.9 (CH_2, C-4), 11.6 (CH_3, C-5). **IR** (ATR, neat): 2925, 2874, 2816, 1718, 1611, 1508, 1464, 1320, 1297, 1205, 1092, 1004, 908, 804, 769, 731 cm^{-1}. **HRMS** (EI): *m/z* calcd for $C_{25}H_{32}N_2O^{\bullet+}$ [$M^{\bullet+}$]: 376.2509; found: 376.2506.

2-Benzyl-3,3-bis(4-(dimethylamino)phenyl)propanal 7g (MJH-II-48): To a solution of **5d** (70.6 mg, 0.208 mmol) in CH_3CN (4 mL) was added **4f** (60.8 mg, 0.201 mmol) in CH_3CN (4 mL) in one portion at room temperature. After 2 h, dilute aq. HCl (2 M, 3 mL) was added. The reaction mixture was left to stir at room temperature for 30 min. The solution was then diluted in CH_2Cl_2 (10 mL) and washed with aq. saturated $NaHCO_3$ solution. The organic layer was separated, the aqueous layer was extracted with CH_2Cl_2 (3 × 20 mL), the combined organic layers were dried over $MgSO_4$, and the solvent was evaporated in vacuo. The crude dark green oil was purified by flash chromatography (silica gel, CH_2Cl_2/Et_2O = 20:1): **7g** (34.8 mg, yield: 45%), yellow oil. ^{1}H and ^{13}C NMR spectral data agree with those previously reported.[S13]

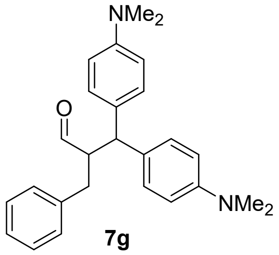

7g

^{1}H NMR (400 MHz, $CDCl_3$): δ = 9.54 (d, *J* = 3.6 Hz, 1 H), 7.25–7.17 (m, 5 H), 7.13 (d, *J* = 8.7 Hz, 1 H), 7.09-7.06 (m, 2 H), 6.72 (d, *J* = 8.7 Hz, 1 H), 6.63 (d, *J* = 8.8 Hz, 1 H), 4.03 (d, *J* = 10.7 Hz, 1 H), 3.50 (ddt, *J* = 10.8, 9.5, 3.8 Hz, 1 H), 2.85–2.79 (m, 1 H). **^{13}C{^{1}H} NMR** (101 MHz, $CDCl_3$): δ = 205.0, 149.4, 149.4, 139.2, 130.9, 130.3, 129.5, 129.2, 128.7, 128.7, 128.5, 126.3, 113.1, 113.1, 113.0, 57.9, 50.8, 40.8, 40.7, 35.0.

5. Kinetic Experiments

5.1 Kinetics in Acetonitrile

Table S1. Kinetics of the reactions of **4b** with **5d** in MeCN at 20 °C (stopped-flow, λ = 606 nm)

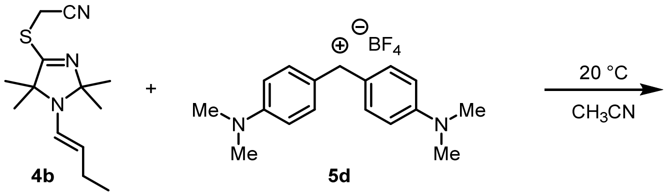

[**4b**][a] (M)	[**5d**] (M)	k_{obs} (s^{-1})
7.24×10^{-4}	2.47×10^{-5}	3.06×10^{-2}
1.43×10^{-3}		6.06×10^{-2}
2.14×10^{-3}		9.09×10^{-2}
2.86×10^{-3}		1.21×10^{-2}

$k_2 = 4.24 \times 10^{1}\ M^{-1}\ s^{-1}$

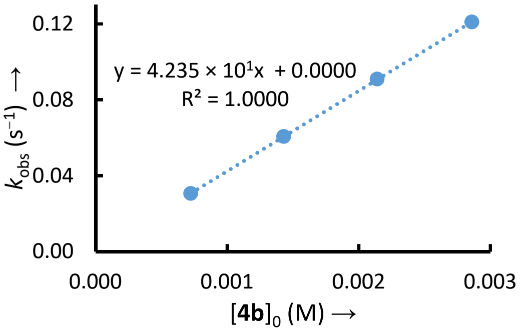

[a] The content of **4b** in a mixture containing 2,6-lutidine, **3a**, and **4b** (total: 118.6 mg) was determined to be 51.3 mg (see Figure S7 for the ^{1}H NMR spectrum of the sample). This mass was used to calculate the concentrations of the enamine **4b**.

Table S2. Kinetics of the reactions of **4b** with **5e** in MeCN at 20 °C (stopped-flow, λ = 612 nm)

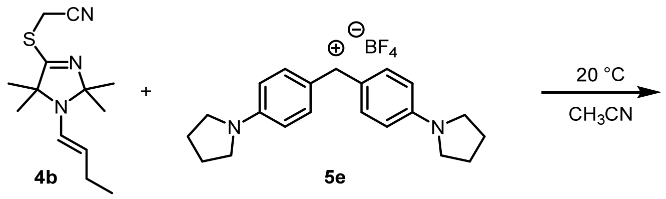

4b **5e**

[**4b**][a] (M)	[**5e**] (M)	k_{obs} (s^{-1})
7.24×10^{-4}		1.02×10^{-2}
1.43×10^{-3}	2.52×10^{-5}	1.99×10^{-2}
2.14×10^{-3}		2.94×10^{-2}
2.86×10^{-3}		3.91×10^{-2}

$k_2 = 1.35 \times 10^1$ M^{-1} s^{-1}

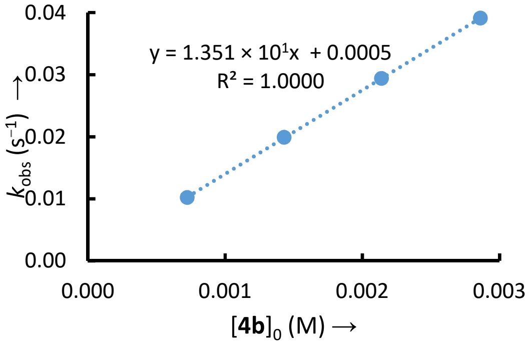

[a] The content of **4b** in a mixture containing 2,6-lutidine, **3a**, and **4b** (total: 118.6 mg) was determined to be 51.3 mg (see Figure S7 for the ^{1}H NMR spectrum of the sample). This mass was used to calculate the concentrations of the enamine **4b**.

Table S3. Kinetics of the reactions of **4b** with **5f** in MeCN at 20 °C (stopped-flow, λ = 620 nm)

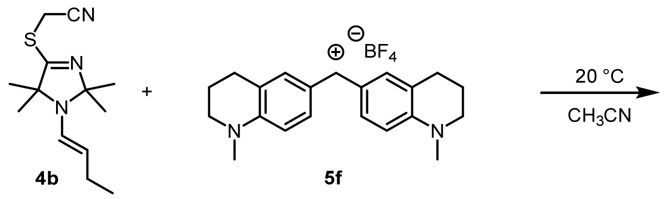

4b **5f**

[**4b**][a] (M)	[**5f**] (M)	k_{obs} (s^{-1})
7.24×10^{-4}		4.49×10^{-3}
1.43×10^{-3}	2.49×10^{-5}	8.44×10^{-3}
2.14×10^{-3}		1.24×10^{-2}
2.86×10^{-3}		1.63×10^{-2}

$k_2 = 5.53$ M^{-1} s^{-1}

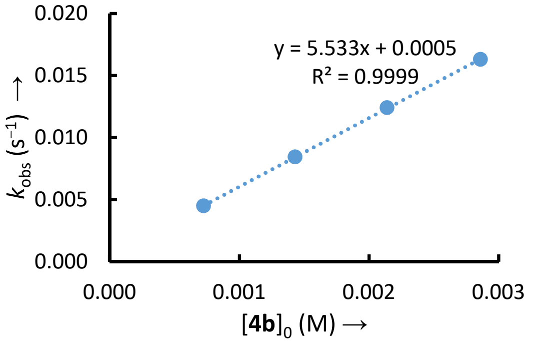

[a] The content of **4b** in a mixture containing 2,6-lutidine, **3a**, and **4b** (total: 118.6 mg) was determined to be 51.3 mg (see Figure S7 for the ^{1}H NMR spectrum of the sample). This mass was used to calculate the concentrations of the enamine **4b**.

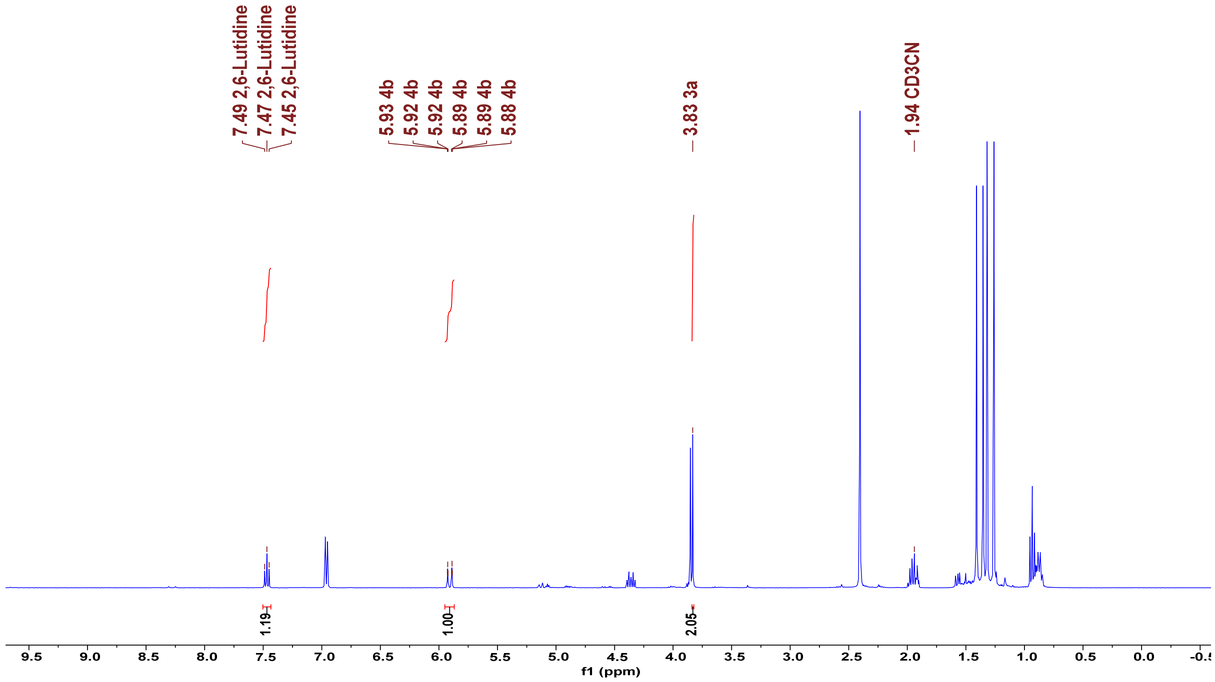

Figure S7. ^{1}H NMR (CD_3CN, 400 MHz) spectrum of the **4b** sample used for the kinetic measurements in Tables S1-S3. Labelled resonances were used to calculate the ratio of components in the sample.

Table S4. Kinetics of the reactions of **4d** with **5b** in MeCN at 20 °C (stopped-flow, λ = 586 nm)

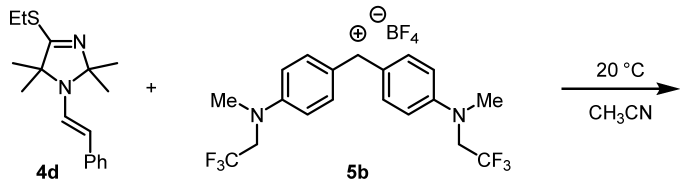

[**4d**] (M)	[**5b**] (M)	k_{obs} (s^{-1})
4.98×10^{-4}	4.99×10^{-5}	2.68
9.96×10^{-4}		5.30
1.50×10^{-3}		8.23
1.99×10^{-3}		1.06×10^{1}

$k_2 = 5.36 \times 10^3\ M^{-1}\ s^{-1}$

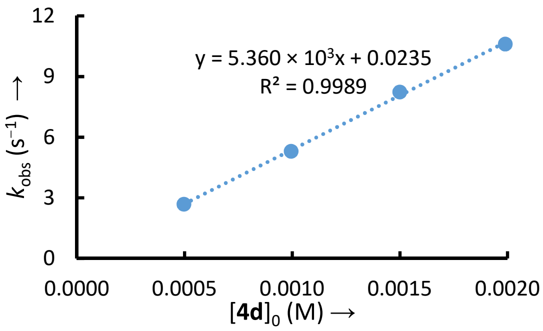

Table S5. Kinetics of the reactions of **4d** with **5c** in MeCN at 20 °C (stopped-flow, λ = 612 nm)

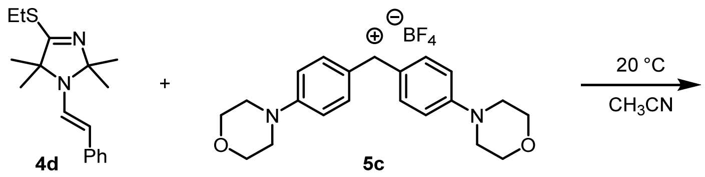

[**4d**] (M)	[**5c**] (M)	k_{obs} (s^{-1})
4.98×10^{-4}	5.02×10^{-5}	1.20×10^{-1}
9.96×10^{-4}		2.36×10^{-1}
1.50×10^{-3}		3.60×10^{-1}
1.99×10^{-3}		4.77×10^{-1}

$k_2 = 2.40 \times 10^2\ M^{-1}\ s^{-1}$

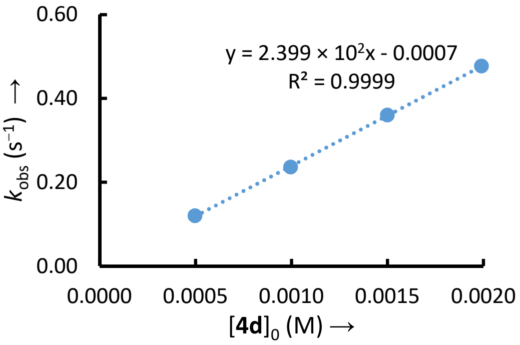

Table S6. Kinetics of the reactions of **4d** with **5d** in MeCN at 20 °C (stopped-flow, λ = 606 nm)

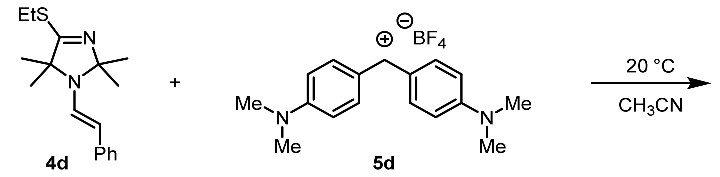

[**4d**] (M)	[**5d**] (M)	k_{obs} (s^{-1})
4.98×10^{-4}		2.53×10^{-2}
9.96×10^{-4}	2.37×10^{-5}	2.87×10^{-2}
1.50×10^{-3}		3.33×10^{-2}
1.99×10^{-3}		3.76×10^{-2}

$k_2 = 8.33\ M^{-1}\ s^{-1}$

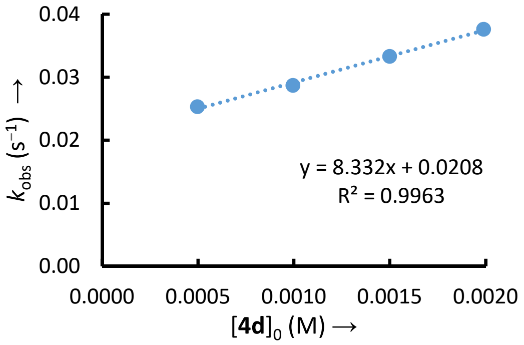

Table S7. Kinetics of the reactions of **4e** with **5b** in MeCN at 20 °C (stopped-flow, λ = 586 nm)

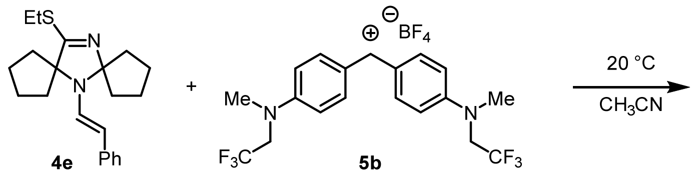

[**4e**] (M)	[**5b**] (M)	k_{obs} (s^{-1})
9.95×10^{-4}		2.56
1.99×10^{-3}	5.02×10^{-5}	4.87
2.98×10^{-3}		7.40
3.98×10^{-3}		1.03×10^{1}

$k_2 = 2.59 \times 10^{3}\ M^{-1}\ s^{-1}$

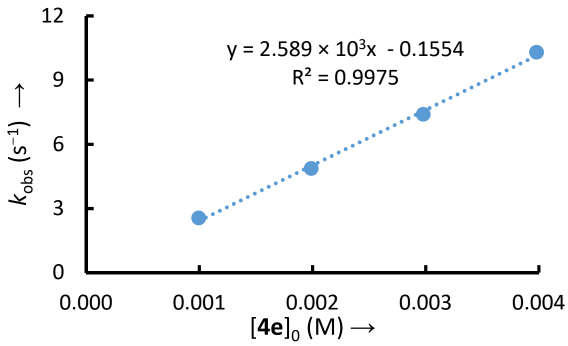

Table S8. Kinetics of the reactions of **4e** with **5c** in MeCN at 20 °C (stopped-flow, λ = 612 nm)

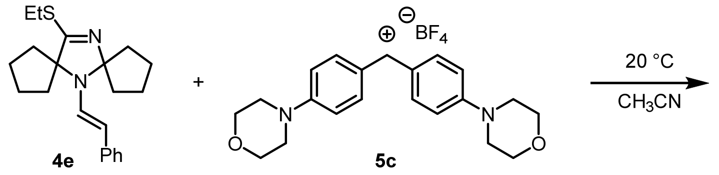

[**4e**] (M)	[**5c**] (M)	k_{obs} (s^{-1})
9.95×10^{-4}		9.98×10^{-2}
1.99×10^{-3}	4.97×10^{-5}	2.00×10^{-1}
2.98×10^{-3}		2.88×10^{-1}
3.98×10^{-3}		3.85×10^{-1}

$k_2 = 9.49 \times 10^1\ M^{-1}\ s^{-1}$

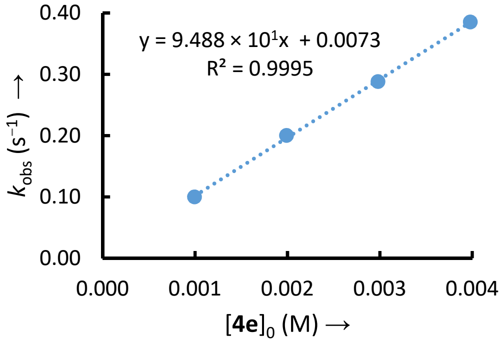

Table S9. Kinetics of the reactions of **4e** with **5d** in MeCN at 20 °C (stopped-flow, λ = 606 nm)

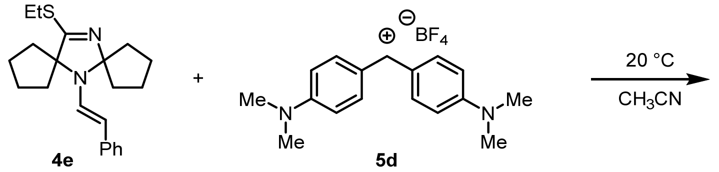

[**4e**] (M)	[**5d**] (M)	k_{obs} (s^{-1})
1.68×10^{-3}		5.20×10^{-2}
3.41×10^{-3}	2.48×10^{-5}	5.91×10^{-2}
5.10×10^{-3}		6.75×10^{-2}
6.79×10^{-3}		7.50×10^{-2}

$k_2 = 4.55\ M^{-1}\ s^{-1}$

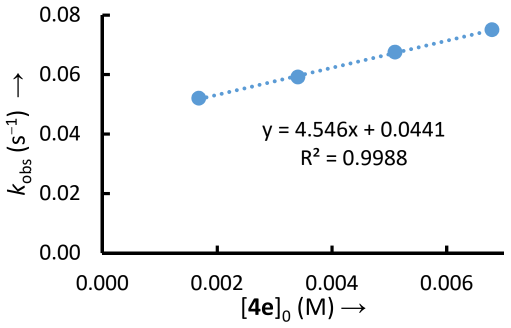

Table S10. Kinetics of the reactions of **4f** with **5b** in MeCN at 20 °C (stopped-flow, λ = 586 nm)

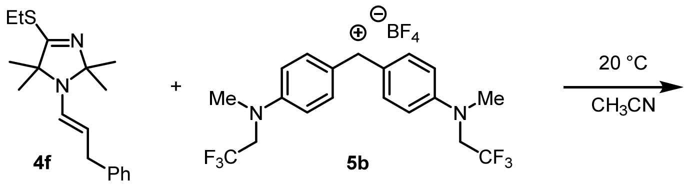

[**4f**] (M)	[**5b**] (M)	k_{obs} (s^{-1})
3.00×10^{-4}		3.14
6.00×10^{-4}	2.97×10^{-5}	6.56
9.01×10^{-4}		1.00×10^{1}
1.18×10^{-3}		1.33×10^{1}

$k_2 = 1.15 \times 10^4\ M^{-1}\ s^{-1}$

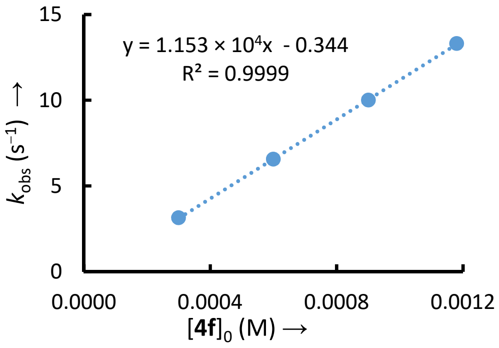

Table S11. Kinetics of the reactions of **4f** with **5c** in MeCN at 20 °C (stopped-flow, λ = 612 nm)

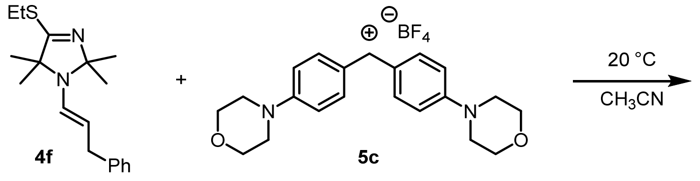

[**4f**] (M)	[**5c**] (M)	k_{obs} (s^{-1})
3.00×10^{-4}		2.13×10^{-1}
6.00×10^{-4}	2.93×10^{-5}	4.36×10^{-1}
9.01×10^{-4}		6.59×10^{-1}
1.18×10^{-3}		8.60×10^{-1}

$k_2 = 7.36 \times 10^2\ M^{-1}\ s^{-1}$

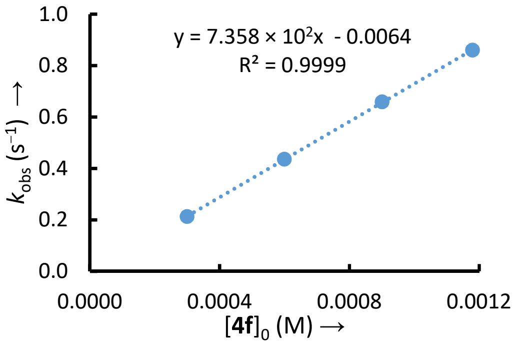

Table S12. Kinetics of the reactions of **4f** with **5d** in MeCN at 20 °C (stopped-flow, λ = 606 nm)

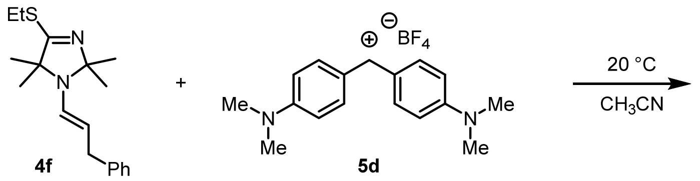

[**4f**] (M)	[**5d**] (M)	k_{obs} (s^{-1})
3.00×10^{-4}		1.75×10^{-2}
6.00×10^{-4}	3.03×10^{-5}	3.67×10^{-2}
9.01×10^{-4}		5.58×10^{-2}
1.18×10^{-3}		7.35×10^{-2}

$k_2 = 6.36 \times 10^1$ M^{-1} s^{-1}

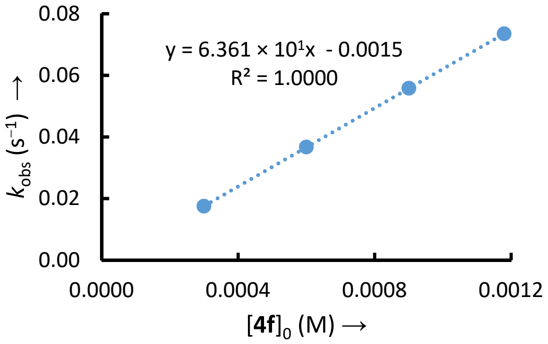

Table S13. Kinetics of the reactions of **4f** with **5e** in MeCN at 20 °C (stopped-flow, λ = 612 nm)

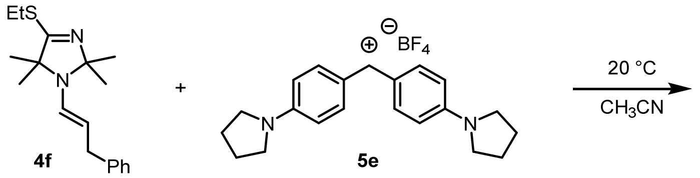

[**4f**] (M)	[**5e**] (M)	k_{obs} (s^{-1})
2.51×10^{-4}		6.50×10^{-3}
5.02×10^{-4}	2.48×10^{-5}	1.30×10^{-2}
7.60×10^{-4}		1.93×10^{-2}
1.00×10^{-3}		2.51×10^{-2}

$k_2 = 2.48 \times 10^1$ M^{-1} s^{-1}

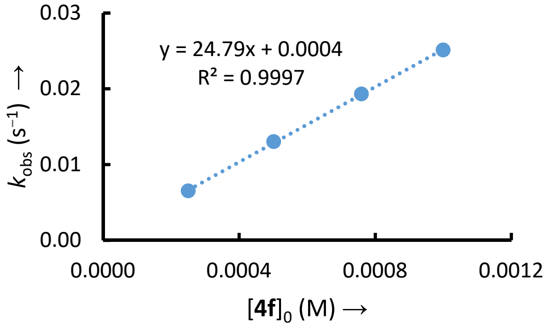

Table S14. Kinetics of the reactions of **4g** with **5a** in MeCN at 20 °C (stopped-flow, λ = 434 nm)

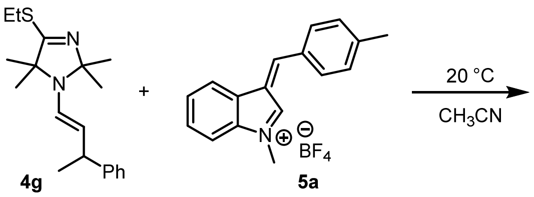

[**4g**] (M)	[**5a**] (M)	k_{obs} (s^{-1})
2.51×10^{-4}		3.61
4.99×10^{-4}	2.49×10^{-5}	6.55
7.51×10^{-4}		1.06×10^{1}
9.99×10^{-4}		1.46×10^{1}

$k_2 = 1.48 \times 10^4$ M^{-1} s^{-1}

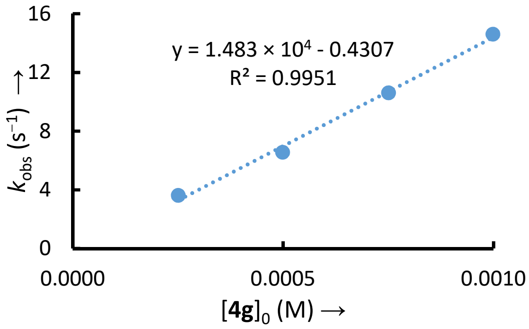

Table S15. Kinetics of the reactions of **4g** with **5b** in MeCN at 20 °C (stopped-flow, λ = 586 nm)

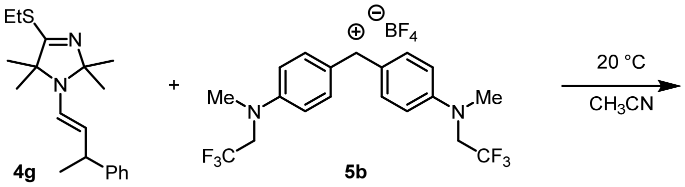

[**4g**] (M)	[**5b**] (M)	k_{obs} (s^{-1})
2.51×10^{-4}		2.35×10^{-1}
4.99×10^{-4}	2.50×10^{-5}	4.81×10^{-1}
7.51×10^{-4}		7.15×10^{-1}
9.99×10^{-4}		9.62×10^{-1}

$k_2 = 9.68 \times 10^2$ M^{-1} s^{-1}

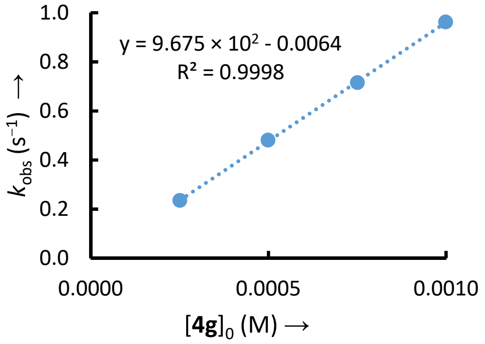

Table S16. Kinetics of the reactions of **4g** with **5c** in MeCN at 20 °C (stopped-flow, λ = 612 nm)

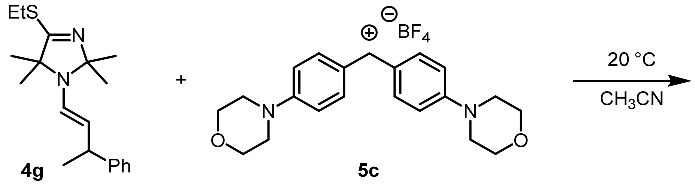

[**4g**] (M)	[**5c**] (M)	k_{obs} (s^{-1})
2.51×10^{-4}	2.57×10^{-5}	1.38×10^{-2}
4.99×10^{-4}		2.87×10^{-2}
7.51×10^{-4}		4.14×10^{-2}
9.99×10^{-4}		5.49×10^{-2}

$k_2 = 5.45 \times 10^1\ M^{-1}\ s^{-1}$

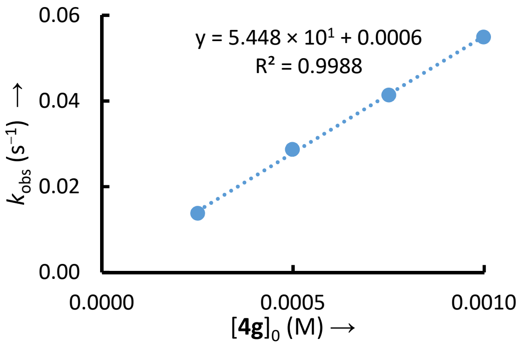

Table S17. Kinetics of the reactions of **4h** with **5b** in MeCN at 20 °C (stopped-flow, λ = 586 nm)

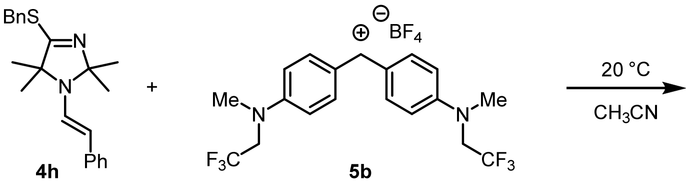

[**4h**] (M)	[**5b**] (M)	k_{obs} (s^{-1})
5.14×10^{-4}	5.02×10^{-5}	2.56
1.03×10^{-3}		5.31
1.54×10^{-3}		8.03
2.05×10^{-3}		1.10×10^1

$k_2 = 5.48 \times 10^3\ M^{-1}\ s^{-1}$

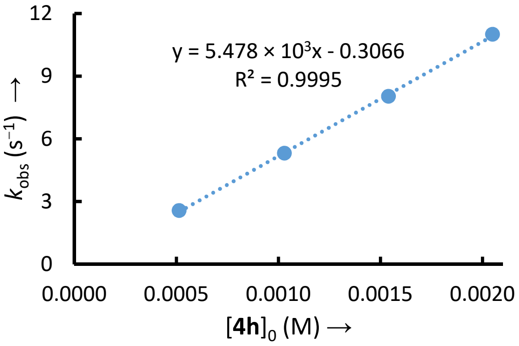

Table S18. Kinetics of the reactions of **4h** with **5c** in MeCN at 20 °C (stopped-flow, λ = 612 nm)

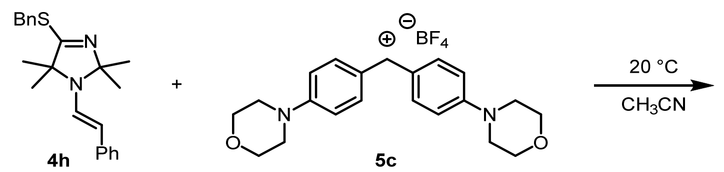

[**4h**] (M)	[**5c**] (M)	k_{obs} (s^{-1})
5.14×10^{-4}		1.15×10^{-1}
1.03×10^{-3}	4.99×10^{-5}	2.34×10^{-1}
1.54×10^{-3}		3.50×10^{-1}
2.05×10^{-3}		4.75×10^{-1}

$k_2 = 2.34 \times 10^2\ M^{-1}\ s^{-1}$

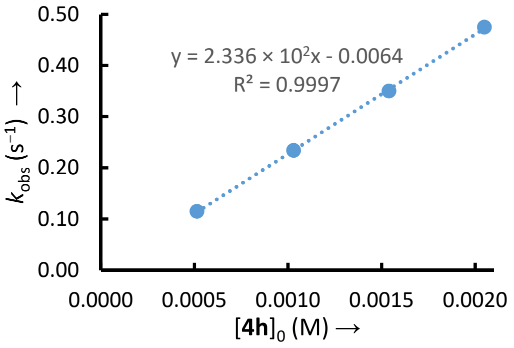

Table S19. Kinetics of the reactions of **4h** with **5d** in MeCN at 20 °C (stopped-flow, λ = 606 nm)

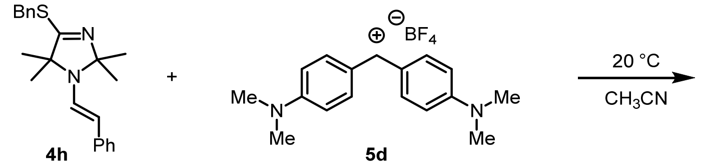

[**4h**] (M)	[**5d**] (M)	k_{obs} (s^{-1})
5.14×10^{-4}		2.52×10^{-2}
1.03×10^{-3}	9.83×10^{-6}	3.03×10^{-2}
1.54×10^{-3}		3.61×10^{-2}
2.05×10^{-3}		4.30×10^{-2}

$k_2 = 1.16 \times 10^1\ M^{-1}\ s^{-1}$

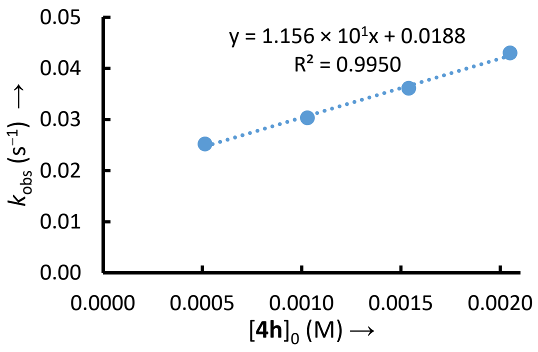

Table S20. Kinetics of the reactions of **4i** with **5a** in in MeCN at 20 °C (stopped-flow, λ = 434 nm)

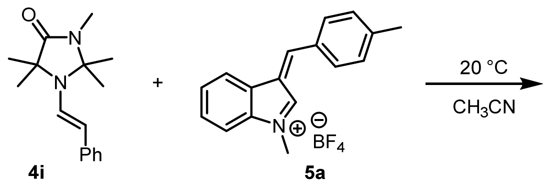

[**4i**] (M)	[**5a**] (M)	k_{obs} (s^{-1})
5.00×10^{-4}		4.90
1.00×10^{-3}	5.00×10^{-5}	1.10×10^{1}
1.50×10^{-3}		1.55×10^{1}
2.00×10^{-3}		2.11×10^{1}

$k_2 = 1.06 \times 10^4$ M^{-1} s^{-1}

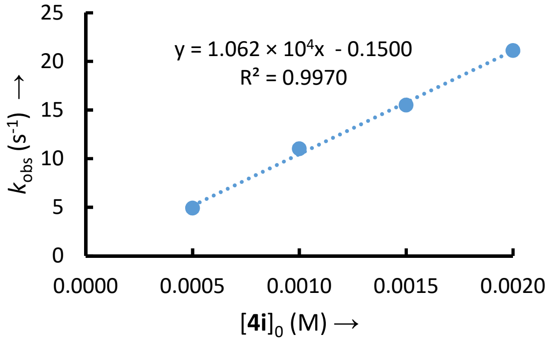

Table S21. Kinetics of the reactions of **4i** with **5b** in MeCN at 20 °C (stopped-flow, λ = 586 nm)

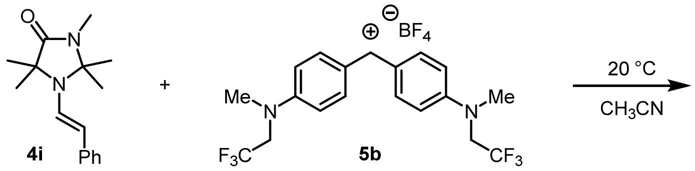

[**4i**] (M)	[**5b**] (M)	k_{obs} (s^{-1})
5.00×10^{-4}		2.10×10^{-1}
1.00×10^{-3}	5.03×10^{-5}	4.33×10^{-1}
1.50×10^{-3}		6.52×10^{-1}
2.00×10^{-3}		8.56×10^{-1}

$k_2 = 4.31 \times 10^2$ M^{-1} s^{-1}

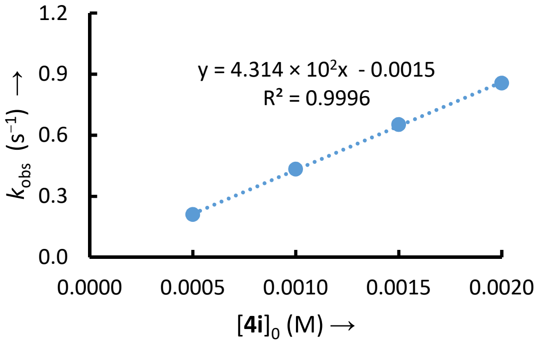

Table S22. Kinetics of the reactions of **4i** with **5c** in MeCN at 20 °C (stopped-flow, λ = 612 nm)

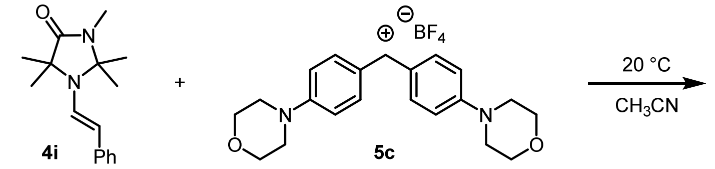

[**4i**] (M)	[**5c**] (M)	k_{obs} (s^{-1})
5.00×10^{-4}		1.44×10^{-2}
1.00×10^{-3}	4.98×10^{-5}	2.43×10^{-2}
1.50×10^{-3}		3.42×10^{-2}
2.00×10^{-3}		4.41×10^{-2}

$k_2 = 1.98 \times 10^1$ M^{-1} s^{-1}

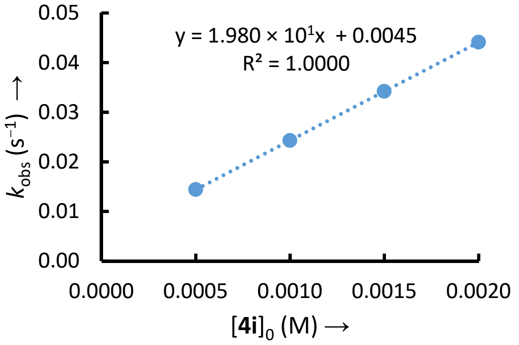

Table S23. Kinetics of the reactions of **4j** with **5a** in MeCN at 20 °C (stopped-flow, λ = 434 nm)

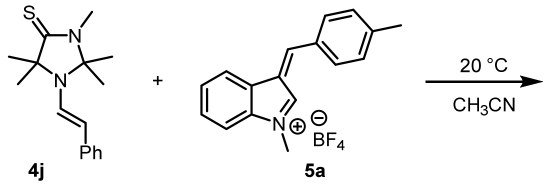

[**4j**] (M)	[**5a**] (M)	k_{obs} (s^{-1})
4.98×10^{-4}		2.08
1.00×10^{-3}	4.87×10^{-5}	3.94
1.49×10^{-3}		5.81
2.00×10^{-3}		7.87

$k_2 = 3.85 \times 10^3$ M^{-1} s^{-1}

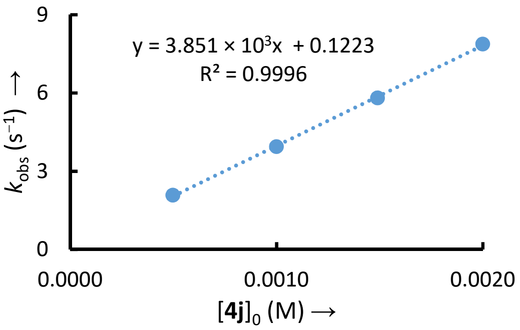

Table S24. Kinetics of the reactions of **4j** with **5b** in MeCN at 20 °C (stopped-flow, λ = 586 nm)

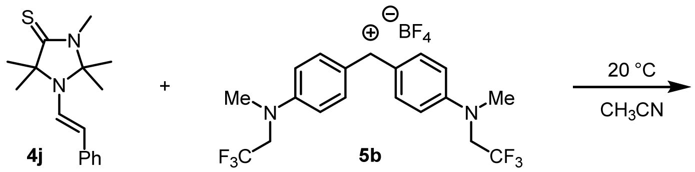

[**4j**] (M)	[**5b**] (M)	k_{obs} (s^{-1})
2.47×10^{-4}		5.71×10^{-2}
1.00×10^{-3}	4.99×10^{-5}	1.57×10^{-1}
1.50×10^{-3}		2.27×10^{-1}
2.00×10^{-3}		2.98×10^{-1}

$k_2 = 1.37 \times 10^2$ M^{-1} s^{-1}

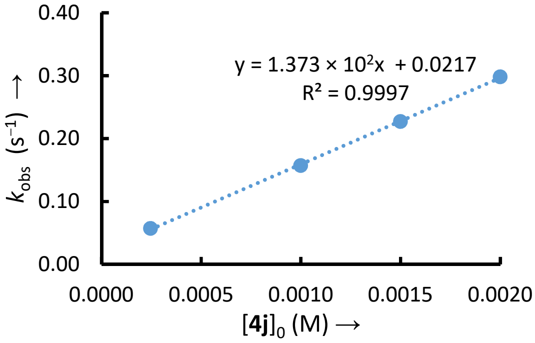

Table S25. Kinetics of the reactions of **4j** with $(mor)_2CH^+$ **5c** in MeCN at 20 °C (stopped-flow, λ = 612 nm)

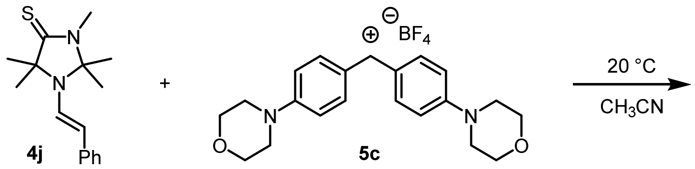

[**4j**] (M)	[**5c**] (M)	k_{obs} (s^{-1})
2.47×10^{-4}		1.29×10^{-2}
1.00×10^{-3}	2.47×10^{-5}	1.81×10^{-2}
1.50×10^{-3}		2.13×10^{-2}
2.00×10^{-3}		2.45×10^{-2}

$k_2 = 6.61$ M^{-1} s^{-1}

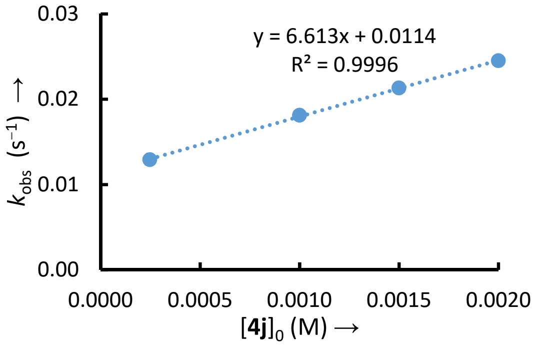

5.2 Kinetics in Dichloromethane

Table S26. Kinetics of the reactions of **4d** with **5b** in CH_2Cl_2 at 20 °C (stopped-flow, λ = 594 nm)

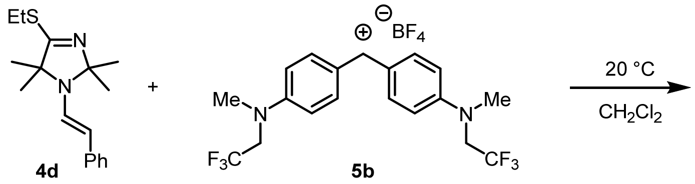

[**4d**] (M)	[**5b**] (M)	k_{obs} (s^{-1})
5.01×10^{-4}		6.74
1.00×10^{-3}	4.99×10^{-5}	1.38×10^{1}
1.50×10^{-3}		2.10×10^{1}
2.00×10^{-3}		2.86×10^{1}

$k_2 = 1.46 \times 10^4$ M^{-1} s^{-1}

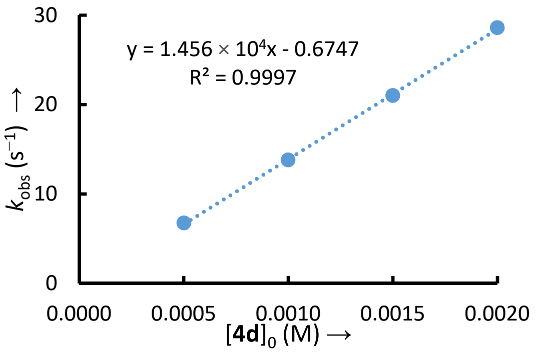

Table S27. Kinetics of the reactions of **4d** with **5c** in CH_2Cl_2 at 20 °C (stopped-flow, λ = 622 nm)

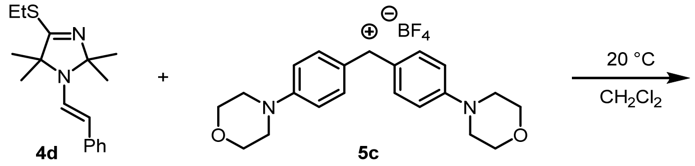

[**4d**] (M)	[**5c**] (M)	k_{obs} (s^{-1})
5.01×10^{-4}		2.00×10^{-1}
1.00×10^{-3}	4.98×10^{-5}	3.66×10^{-1}
1.50×10^{-3}		5.36×10^{-1}
2.00×10^{-3}		6.98×10^{-1}

$k_2 = 3.33 \times 10^2$ M^{-1} s^{-1}

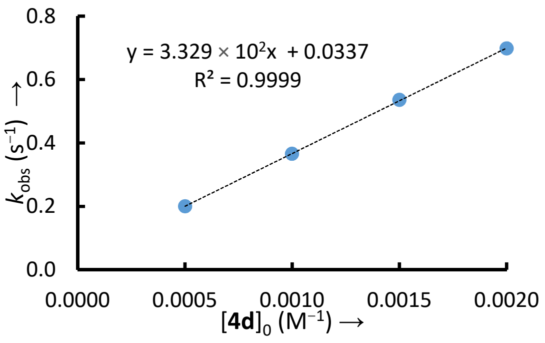

Table S28. Kinetics of the reactions of **4d** with **5h** in CH_2Cl_2 at 20 °C (stopped-flow, λ = 624 nm)

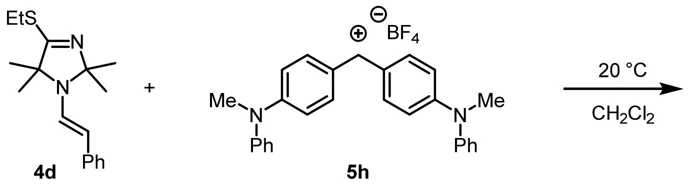

[**4d**] (M)	[**5h**] (M)	k_{obs} (s^{-1})
5.01×10^{-4}	4.97×10^{-5}	2.10×10^{-1}
1.00×10^{-3}		2.88×10^{-1}
1.50×10^{-3}		3.64×10^{-1}
2.00×10^{-3}		4.41×10^{-1}

$k_2 = 1.54 \times 10^2$ M^{-1} s^{-1}

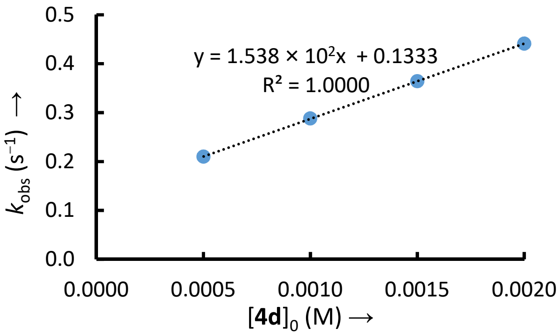

Table S29. Kinetics of the reactions of **4h** with **5j** in CH_2Cl_2 at 20 °C (stopped-flow, λ = 602 nm)

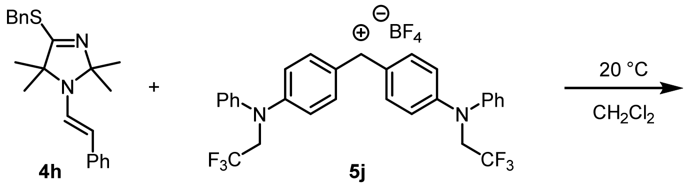

[**4h**] (M)	[**5j**] (M)	k_{obs} (s^{-1})
4.82×10^{-4}	4.98×10^{-5}	3.13×10^{1}
9.63×10^{-4}		6.27×10^{1}
1.44×10^{-3}		9.52×10^{1}
1.93×10^{-3}		1.24×10^{2}

$k_2 = 6.44 \times 10^4$ M^{-1} s^{-1}

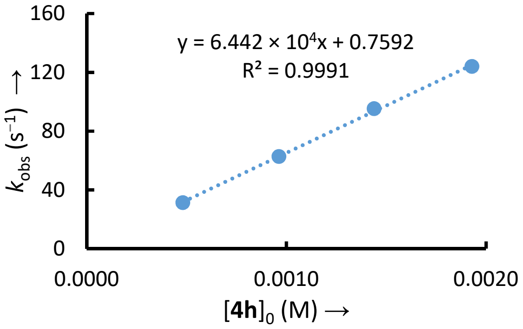

Table S30. Kinetics of the reactions of **4h** with **5b** in CH_2Cl_2 at 20 °C (stopped-flow, λ = 594 nm)

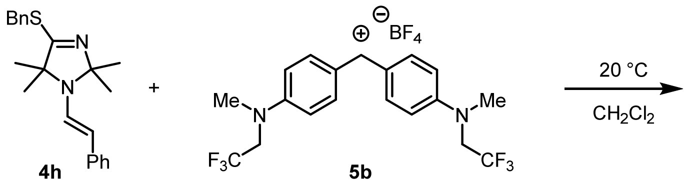

[**4h**] (M)	[**5b**] (M)	k_{obs} (s^{-1})
4.82×10^{-4}		4.54
9.63×10^{-4}	5.08×10^{-5}	9.48
1.44×10^{-3}		1.39×10^{1}
1.93×10^{-3}		1.84×10^{1}

$k_2 = 9.54 \times 10^{3}$ M^{-1} s^{-1}

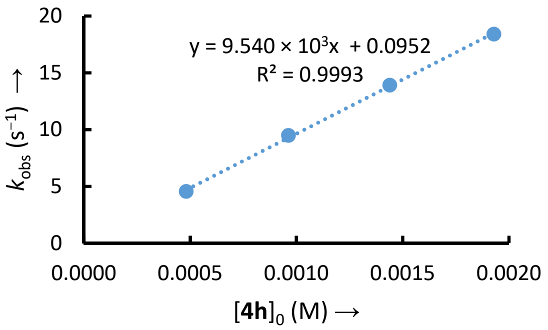

Table S31. Kinetics of the reactions of **4h** with **5c** in CH_2Cl_2 at 20 °C (stopped-flow, λ = 622 nm)

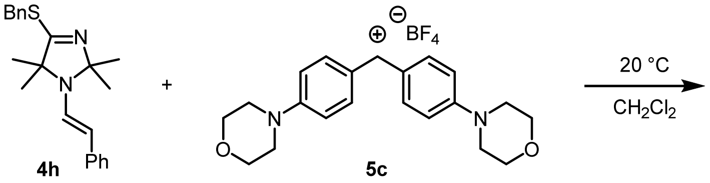

[**4h**] (M)	[**5c**] (M)	k_{obs} (s^{-1})
4.82×10^{-4}		1.60×10^{-1}
9.63×10^{-4}	4.97×10^{-5}	2.92×10^{-1}
1.44×10^{-3}		4.23×10^{-1}
1.93×10^{-3}		5.52×10^{-1}

$k_2 = 2.71 \times 10^{2}$ M^{-1} s^{-1}

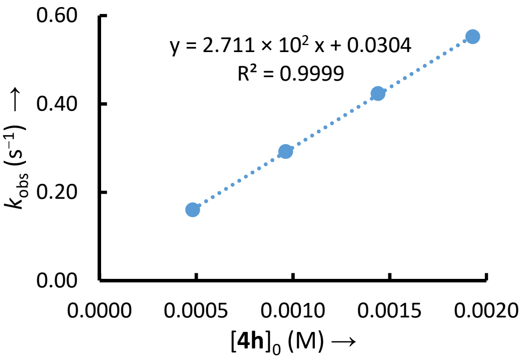

Table S32. Second-order rate constants k_2 for the reactions of the enamines **4d** and **4h** with benzhydrylium tetrafluoroborates **5** (CH_2Cl_2, 20 °C).

Enamines	Electrophiles	Electrophilicity E	k_2 (M^{-1} s^{-1})	N, s_N
**4d**	**5b**	–3.85	1.46×10^4	8.13, 0.97
	**5c**	–5.53	3.33×10^2	
	**5h**	–5.89	1.54×10^2	
**4h**	**5j**	–3.14	6.44×10^4	7.99, 0.98
	**5b**	–3.85	9.54×10^3	
	**5c**	–5.89	2.71×10^2	

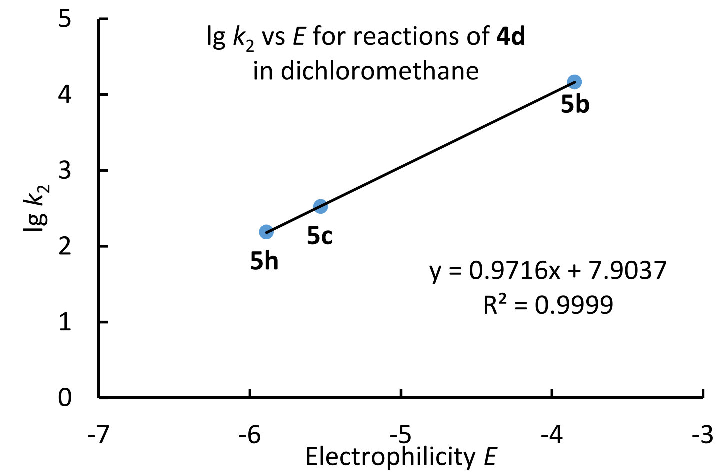

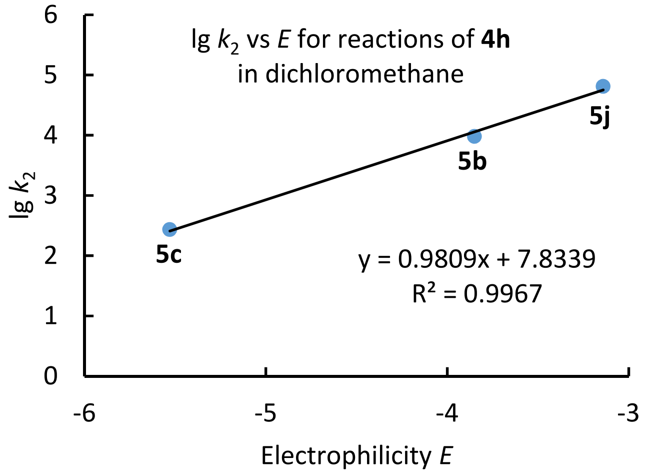

6. Oxidation Potentials of Enamines 4

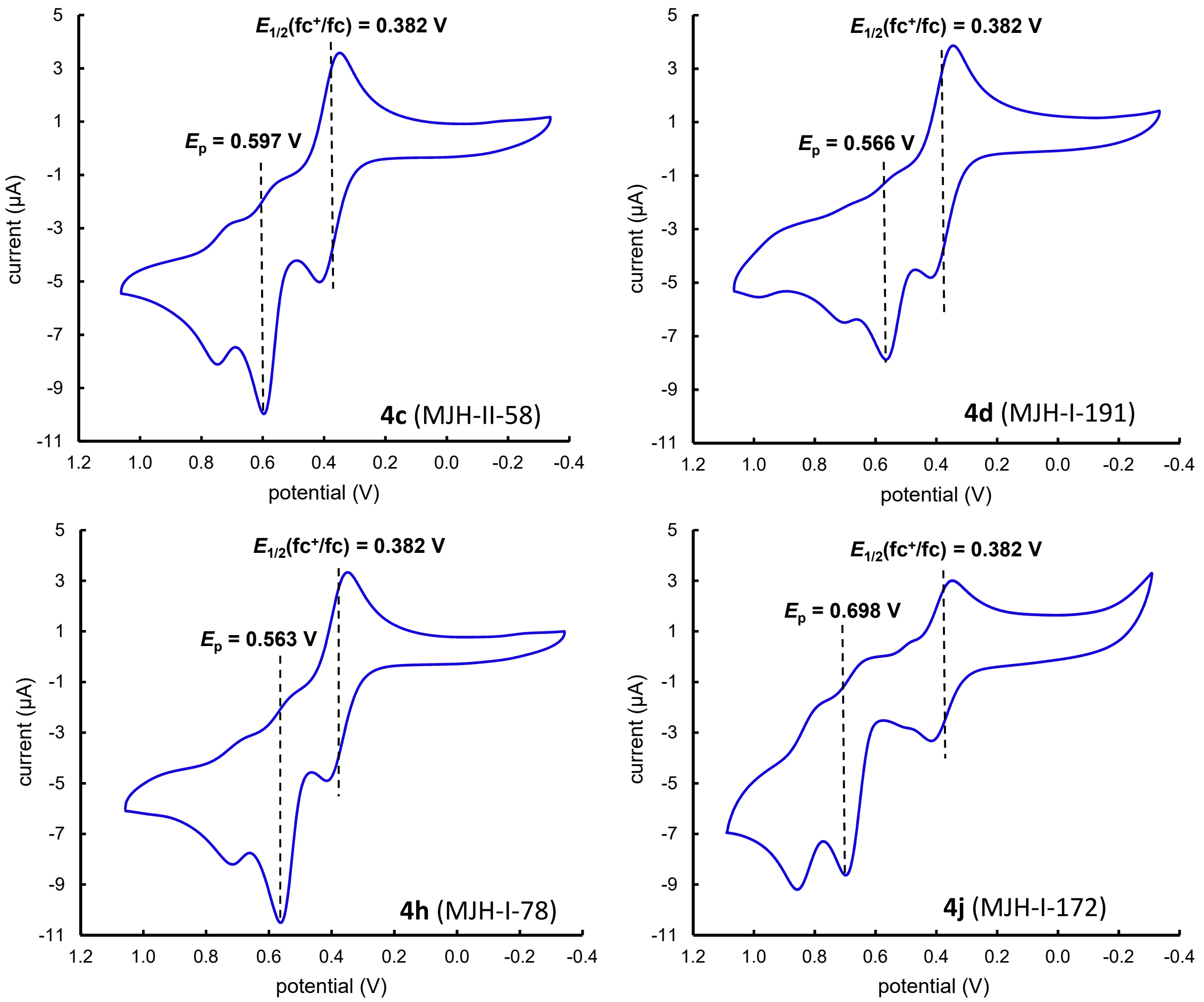

Figure S8. Cyclic voltammograms of enamines **4c**, **4d**, **4h**, and **4j** (in acetonitrile, ferrocene as internal standard).[S14]

The oxidation potential $E_{p,ox}$ of enamines are tabulated in Table S33 along with their nucleophilicity parameters *N* (in MeCN) and individual reactivities towards the benzhydrylium ion **5c**, that is, the data needed to construct Figure 14 in the main text.

Table S33. Oxidation potentials of enamines **4** and reactivity data in acetonitrile (used t).

Enamine	Oxidation Potential E_p (vs SCE)	Nucleophilicity *N*	lg k_2(**4**+**5c**)
**4c** (MJH-II-58)	+0.597 V	7.74	1.95
**4d** (MJH-I-191)	+0.566 V	8.12	2.38
**4h** (MJH-I-78)	+0.563 V	8.30	2.39
**4j** (MJH-I-172)	+0.698 V	6.49	0.82

7. Determination of Equilibrium Constants

Table S34. Determination of the equilibrium constant between **4d** with **5d** in CH_3CN at 20 °C (observed at λ = 606 nm). Stock solutions: **4d** (11.3 mg) in 10.0 mL CH_3CN; **5d** (9.5 mg) in 10.0 mL CH_3CN. Step 0: 65.0 µL **5d** stock solution in 13.3 g CH_3CN.

ε(**5d**) = 1.698 × 10^5 L mol^{-1} cm^{-1}

Step *i*	V_{4d} (mL)	ΣV_{4d} (mL)	V_i (mL)	$A_{eq,i}$	A_0*[a]	A_0*–$A_{eq,i}$	[**4d**]$_{0,i}$ (M)	[**4d**]$_{eq,i}$ (M)	(A_0*–$A_{eq,i}$)/$A_{eq,i}$
0	0.0	0.0	16.9	0.896	0.896	0.0	0.0	0.0	0.0
1	0.2	0.2	17.1	0.851	0.886	0.035	4.57 × 10^{-5}	4.53E-05	0.041
2	0.5	0.7	17.6	0.757	0.860	0.103	1.56E-04	1.54E-04	0.137
3	0.5	1.2	18.1	0.682	0.837	0.155	2.59E-04	2.57E-04	0.227
4	0.5	1.7	18.6	0.62	0.814	0.194	3.57E-04	3.55E-04	0.313
5	0.5	2.2	19.1	0.568	0.793	0.225	4.51E-04	4.48E-04	0.396
6	1.0	3.2	20.1	0.486	0.754	0.268	6.23E-04	6.20E-04	0.551
7	1.0	4.2	21.1	0.424	0.718	0.294	7.79E-04	7.75E-04	0.693
8	1.0	5.2	22.1	0.377	0.685	0.308	9.21E-04	9.17E-04	0.818
9	1.0	6.2	23.1	0.341	0.656	0.315	1.05E-03	1.05E-03	0.923
10	1.0	7.2	24.1	0.310	0.629	0.319	1.17E-03	1.17E-03	1.03

[a] Dilution of the reaction mixture is considered in A_0* = A_0(V_0/V_i).

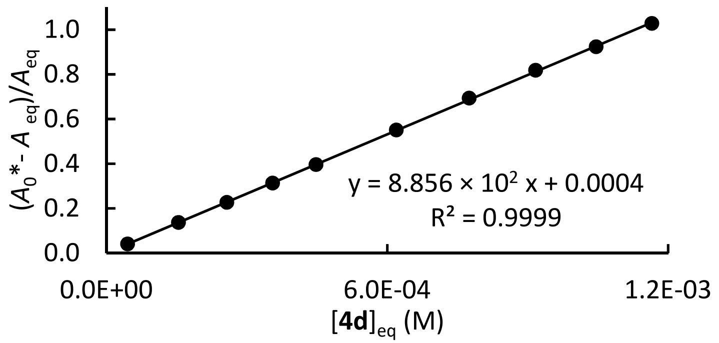

***K* = 885.6 M^{-1}**

Table S35. Determination of the equilibrium constant between **4d** with **5d** in CH_3CN at 20 °C (observed at λ = 606 nm). Stock solutions: **4d** (9.8 mg) in 10.0 mL CH_3CN; **5d** (8.6 mg) in 10.0 mL CH_3CN. Step 0: 70.0 µL **5d** stock solution in 11.2 g CH_3CN.

ε(**5d**) = 1.698×10^5 L mol^{-1} cm^{-1}

Step i	$V_{\mathbf{4d}}$ (mL)	$\Sigma V_{\mathbf{4d}}$ (mL)	V_i (mL)	$A_{eq,i}$	A_0*[a]	A_0*–$A_{eq,i}$	[**4d**]$_{0,i}$ (M)	[**4d**]$_{eq,i}$ (M)	(A_0*–$A_{eq,i}$)/$A_{eq,i}$
0	0.0	0.0	14.3	0.905	0.905	0.0	0.0		
1	0.5	0.5	14.8	0.800	0.874	0.074	1.09E-04	1.08E-04	0.093
2	0.5	1.0	15.3	0.713	0.846	0.133	2.11E-04	2.09E-04	0.186
3	0.5	1.5	15.8	0.642	0.819	0.177	3.06E-04	3.04E-04	0.276
4	0.8	2.3	16.6	0.553	0.780	0.227	4.47E-04	4.44E-04	0.410
5	1.0	3.3	17.6	0.472	0.735	0.263	6.04E-04	6.01E-04	0.558
6	1.0	4.3	18.6	0.412	0.696	0.284	7.45E-04	7.42E-04	0.689
7	0.7	5.0	19.3	0.378	0.671	0.293	8.35E-04	8.32E-04	0.774

[a] Dilution of the reaction mixture is considered in $A_0^* = A_0(V_0/V_i)$.

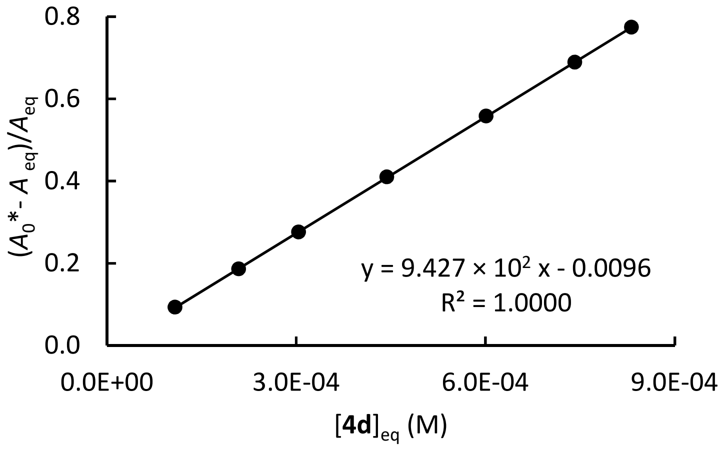

***K* = 942.7 M^{-1}**

Table S36. Determination of the equilibrium constant between **4d** with **5e** in CH_3CN at 20 °C (observed at λ = 612 nm). Stock solutions: **4d** (17.8 mg) in 10.0 mL CH_3CN; **5e** (11.3 mg) in 10.0 mL CH_3CN. Step 0: 55.0 µL **5e** stock solution in 12.08 g CH_3CN.

ε(**5e**) = 1.738 × 10^5 L mol^{-1} cm^{-1}

Step i	$V_{\mathbf{4d}}$ (mL)	$\Sigma V_{\mathbf{4d}}$ (mL)	V_i (mL)	$A_{eq,i}$	A_0*[a]	$A_0^*-A_{eq,i}$	[**4d**]$_{0,i}$ (M)	[**4d**]$_{eq,i}$ (M)	$(A_0^*-A_{eq,i})/A_{eq,i}$
0	0.0	0.0	15.4	0.832	0.832	0.0	0.0	0.0	0.0
1	0.2	0.2	15.6	0.810	0.821	0.0113	7.90E-05	7.89E-05	0.014
2	0.5	0.7	16.1	0.776	0.796	0.0199	2.68E-04	2.68E-04	0.026
3	1.0	1.7	17.1	0.717	0.749	0.0324	6.13E-04	6.12E-04	0.045
4	1.0	2.7	18.1	0.666	0.708	0.0421	9.19E-04	9.19E-04	0.063
5	1.5	4.2	19.6	0.604	0.654	0.0499	1.32E-03	1.32E-03	0.083
6	1.5	5.7	21.1	0.553	0.607	0.0545	1.67E-03	1.66E-03	0.099
7	1.5	7.2	22.6	0.508	0.567	0.0592	1.96E-03	1.96E-03	0.117
8	2.0	9.2	24.6	0.456	0.521	0.0651	2.31E-03	2.30E-03	0.143

[a] Dilution of the reaction mixture is considered in $A_0^* = A_0(V_0/V_i)$.

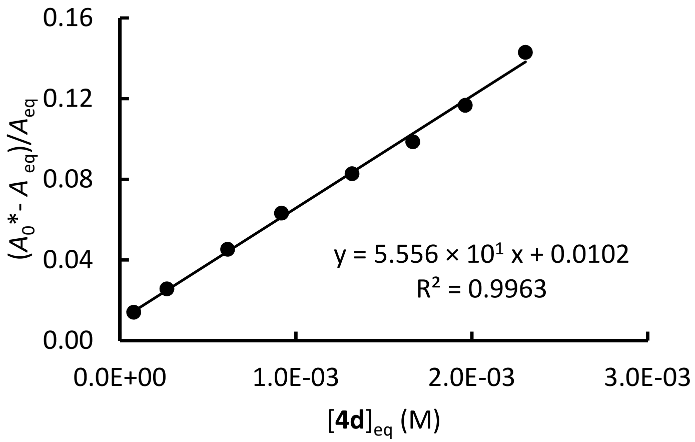

***K* = 55.56 M^{-1}**

Table S37. Determination of the equilibrium constant between **4d** with **5e** in CH_3CN at 20 °C (observed at λ = 612 nm). Stock solutions: **4d** (36.3 mg) in 10.0 mL CH_3CN; **5e** (11.3 mg) in 10.0 mL CH_3CN. Step 0: 55.0 µL **5e** stock solution in 13.18 g CH_3CN.

ε(**5e**) = 1.738×10^5 L mol^{-1} cm^{-1}

Step *i*	V_{4d} (mL)	ΣV_{4d} (mL)	V_i (mL)	$A_{eq,i}$	A_0*[a]	$A_0^*-A_{eq,i}$	[**4d**]$_{0,i}$ (M)	[**4d**]$_{eq,i}$ (M)	$(A_0^*-A_{eq,i})/A_{eq,i}$
0	0.0	0	16.9	0.854	0.854	0.0	0.0		
1	0.3	0.3	17.2	0.827	0.839	0.012	2.20E-04	2.20E-04	0.015
2	0.5	0.8	17.7	0.787	0.815	0.028	5.70E-04	5.70E-04	0.036
3	1.0	1.8	18.7	0.717	0.772	0.055	1.21E-03	1.21E-03	0.076
4	1.0	2.8	19.7	0.658	0.732	0.074	1.79E-03	1.79E-03	0.113
5	1.5	4.3	21.2	0.587	0.680	0.093	2.56E-03	2.56E-03	0.159
6	1.5	5.8	22.7	0.529	0.635	0.106	3.22E-03	3.22E-03	0.201
7	1.5	7.3	24.2	0.482	0.596	0.114	3.80E-03	3.80E-03	0.236
8	2.0	9.3	26.2	0.431	0.550	0.119	4.47E-03	4.47E-03	0.277

[a] Dilution of the reaction mixture is considered in $A_0^* = A_0(V_0/V_i)$.

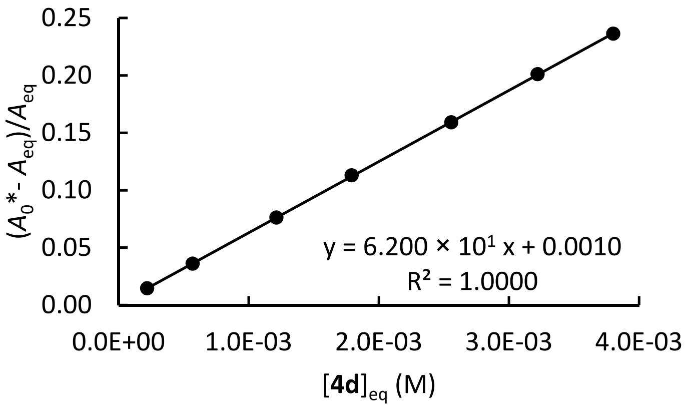

***K* = 62.00 M^{-1}**

Table S38. Determination of the equilibrium constant between **4d** with **5f** in CH_3CN at 20 °C (observed at λ = 620 nm). Stock solutions: **4d** (54.2 mg) in 10.0 mL CH_3CN; **5f** (12.0 mg) in 10.0 mL CH_3CN. Step 0: 65.0 µL **5f** stock solution in 14.3 g CH_3CN.

ε(**5f**) = 1.778×10^5 L mol^{-1} cm^{-1}

Step i	$V_{\mathbf{4d}}$ (mL)	$\Sigma V_{\mathbf{4d}}$ (mL)	V_i (mL)	$A_{eq,i}$	A_0*[a]	$A_0^*-A_{eq,i}$	[**4d**]$_{0,i}$ (M)	[**4d**]$_{eq,i}$ (M)	$(A_0^*-A_{eq,i})/A_{eq,i}$
0	0.0	0.0	18.2	0.782	0.782	0.0	0.0	0.0	0.0
1	0.5	0.5	18.7	0.751	0.761	0.0101	5.01E-04	5.01E-04	0.014
2	1.0	1.5	19.7	0.700	0.723	0.0226	1.43E-03	1.43E-03	0.032
3	1.0	2.5	20.7	0.650	0.688	0.0378	2.26E-03	2.26E-03	0.058
4	1.5	4.0	22.2	0.590	0.641	0.0514	3.38E-03	3.38E-03	0.087
5	1.5	5.5	23.7	0.542	0.601	0.0589	4.35E-03	4.35E-03	0.109
6	2.0	7.5	25.7	0.488	0.554	0.0662	5.47E-03	5.47E-03	0.136
7	2.5	10.0	28.2	0.436	0.505	0.0691	6.65E-03	6.65E-03	0.159

[a] Dilution of the reaction mixture is considered in $A_0^* = A_0(V_0/V_i)$.

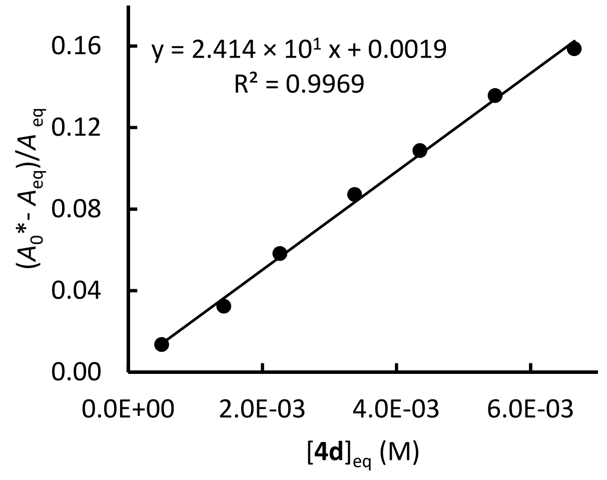

***K* = 24.14 M^{-1}**

Table S39. Determination of the equilibrium constant between **4d** with **5f** in CH_3CN at 20 °C (observed at λ = 620 nm). Stock solutions: **4d** (63.2 mg) in 10.0 mL CH_3CN; **5f** (12.0 mg) in 10.0 mL CH_3CN. Step 0: 65.0 µL **5f** stock solution in 13.0 g CH_3CN.

ε(**5f**) = 1.778×10^5 L mol^{-1} cm^{-1}

Step i	$V_{\mathbf{4d}}$ (mL)	$\Sigma V_{\mathbf{4d}}$ (mL)	V_i (mL)	$A_{eq,i}$	A_0*[a]	$A_0^*–A_{eq,i}$	[**4d**]$_{0,i}$ (M)	[**4d**]$_{eq,i}$ (M)	$(A_0^*–A_{eq,i})/A_{eq,i}$
0	0.0	0.0	16.6	0.868	0.868	0.0	0.0	0.0	0.0
1	1.0	1.0	17.6	0.793	0.819	0.0258	1.24E-03	1.24E-03	0.033
2	1.0	2.0	18.6	0.729	0.775	0.0459	2.35E-03	2.35E-03	0.063
3	1.5	3.5	20.1	0.651	0.717	0.0662	3.81E-03	3.81E-03	0.102
4	1.5	5.0	21.6	0.588	0.667	0.0794	5.06E-03	5.06E-03	0.135
5	2.0	7.0	23.6	0.521	0.611	0.0900	6.49E-03	6.49E-03	0.173
6	2.5	9.5	26.1	0.462	0.553	0.0905	7.96E-03	7.96E-03	0.196

[a] Dilution of the reaction mixture is considered in $A_0^* = A_0(V_0/V_i)$.

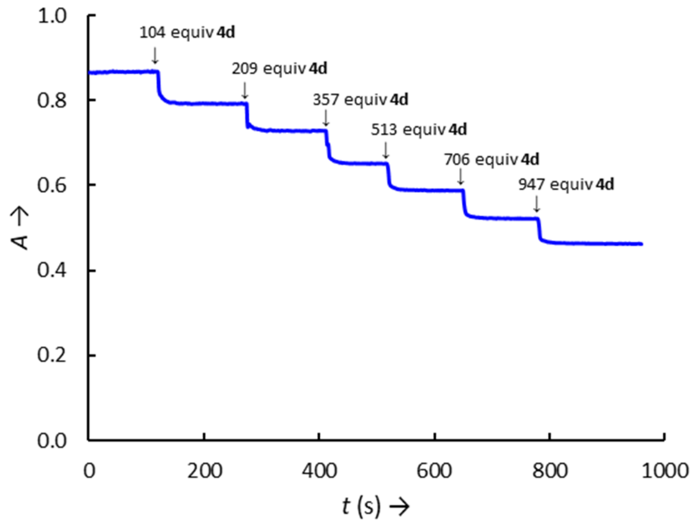

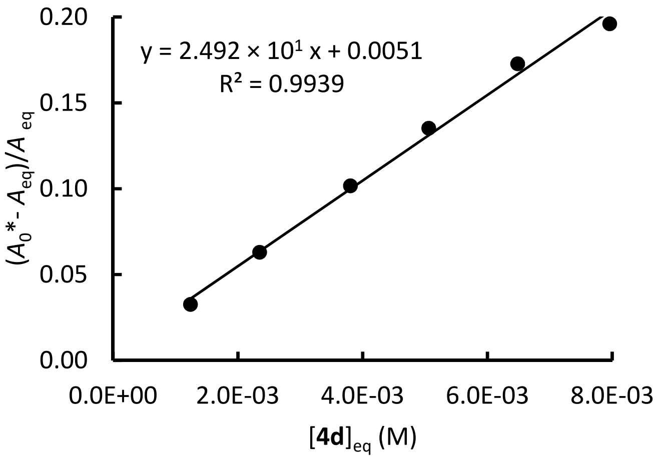

***K* = 24.92 M^{-1}**

Determination of the Lewis Basicity of the enamine 4d (in acetonitrile)

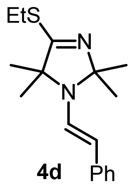

4d

Lewis acid	LA_{MeCN}	K (M^{-1})		Average K (M^{-1})	lg K	LB_{MeCN}	Ave LB_{MeCN}
		Trial #1	Trial #2				
**5d**	–9.82	942	886	914	2.96	12.78	
**5e**	–10.83	55.6	62.0	58.8	1.77	12.60	12.68
**5f**	–11.27	24.1	24.9	24.5	1.39	12.66	

8. Copies of ^{1}H NMR, ^{13}C NMR, and IR Spectra

6,12-Diazadispiro[4.1.4^{7}.2^{5}]tridecane-13-thione (2b) (MJH-I-60)

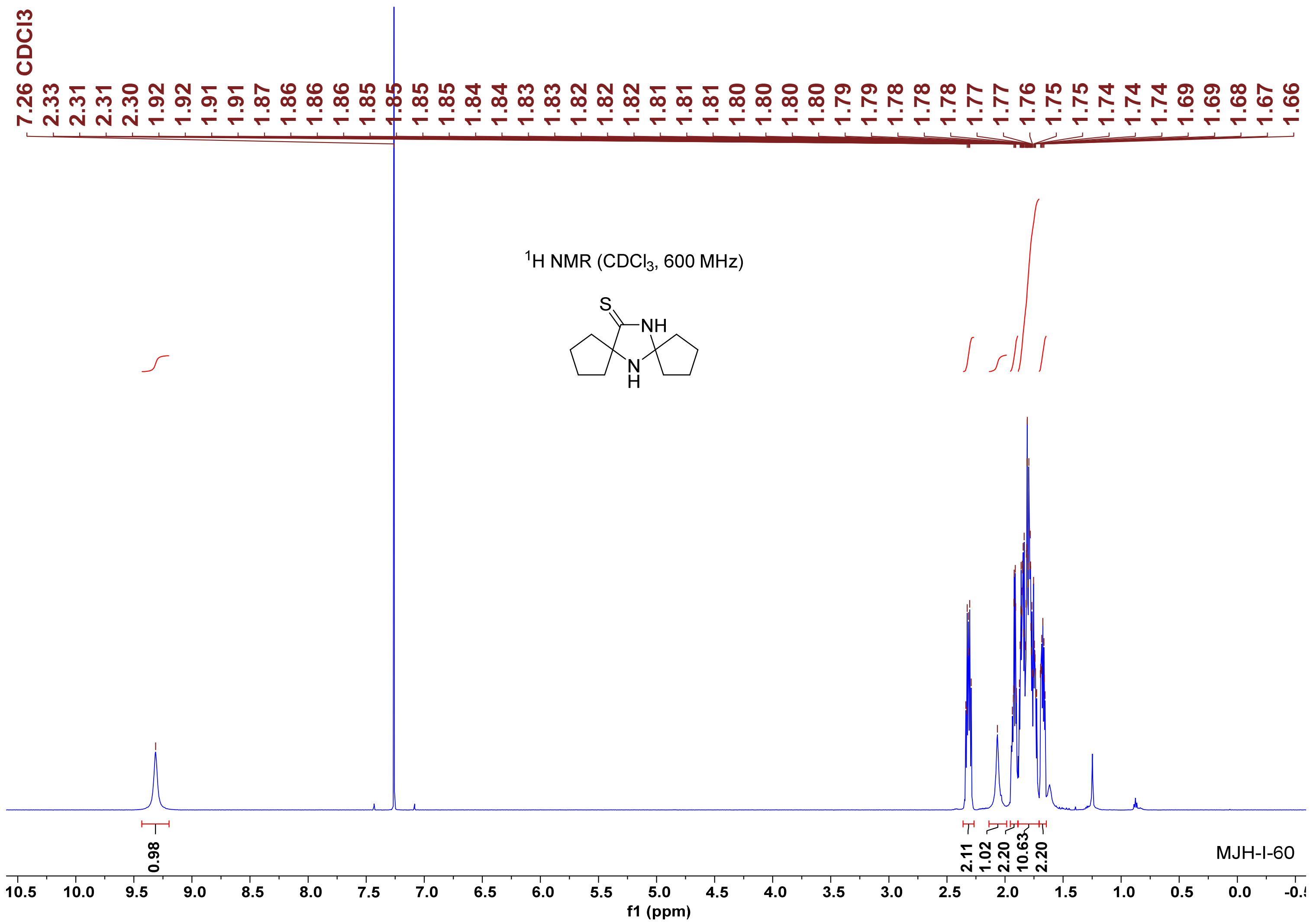

6,12-Diazadispiro[4.1.4^7.2^5]tridecane-13-thione (2b) (MJH-I-60)

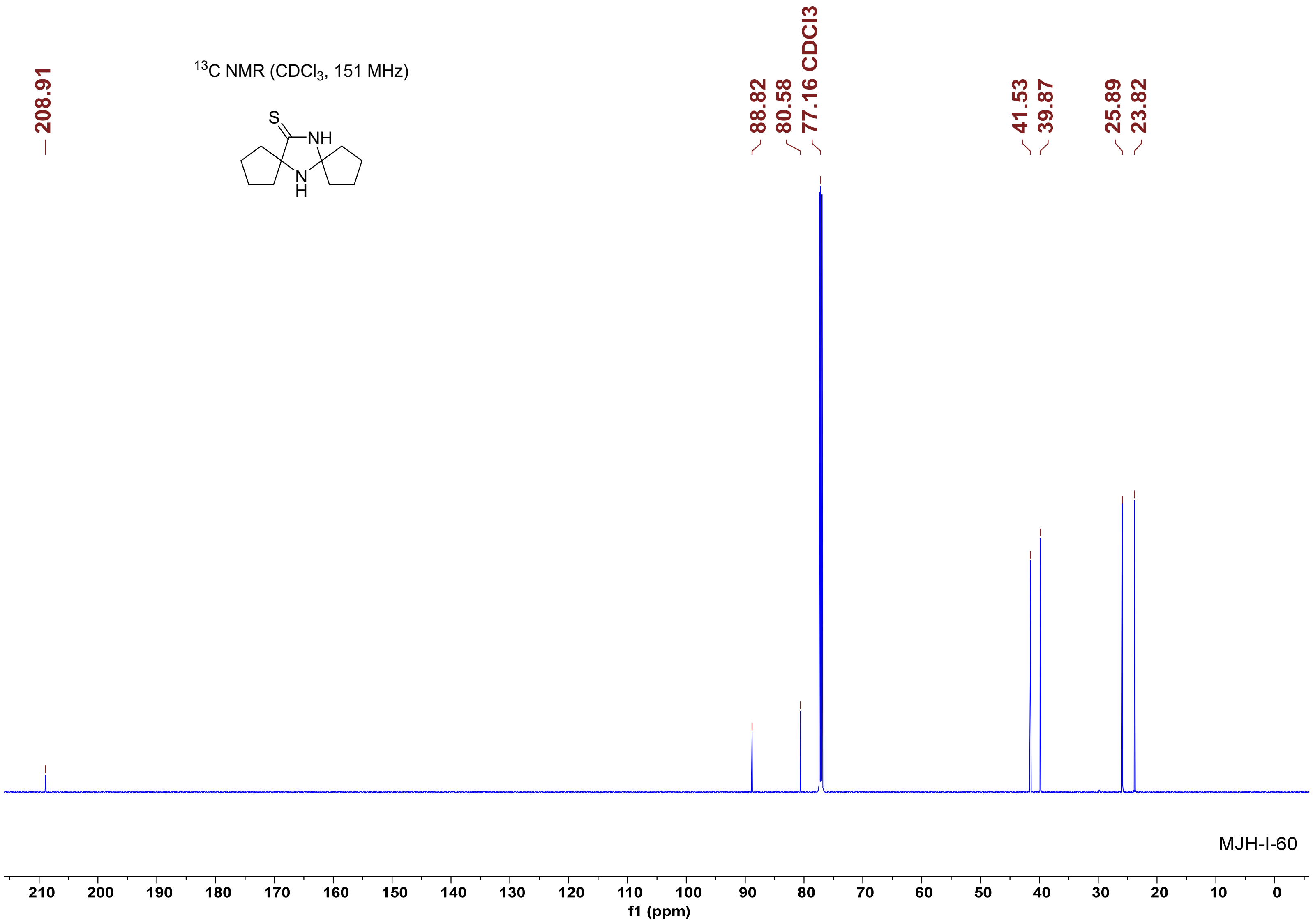

6,12-Diazadispiro[4.1.4^7.2^5]tridecane-13-thione (2b) (MJH-I-60)

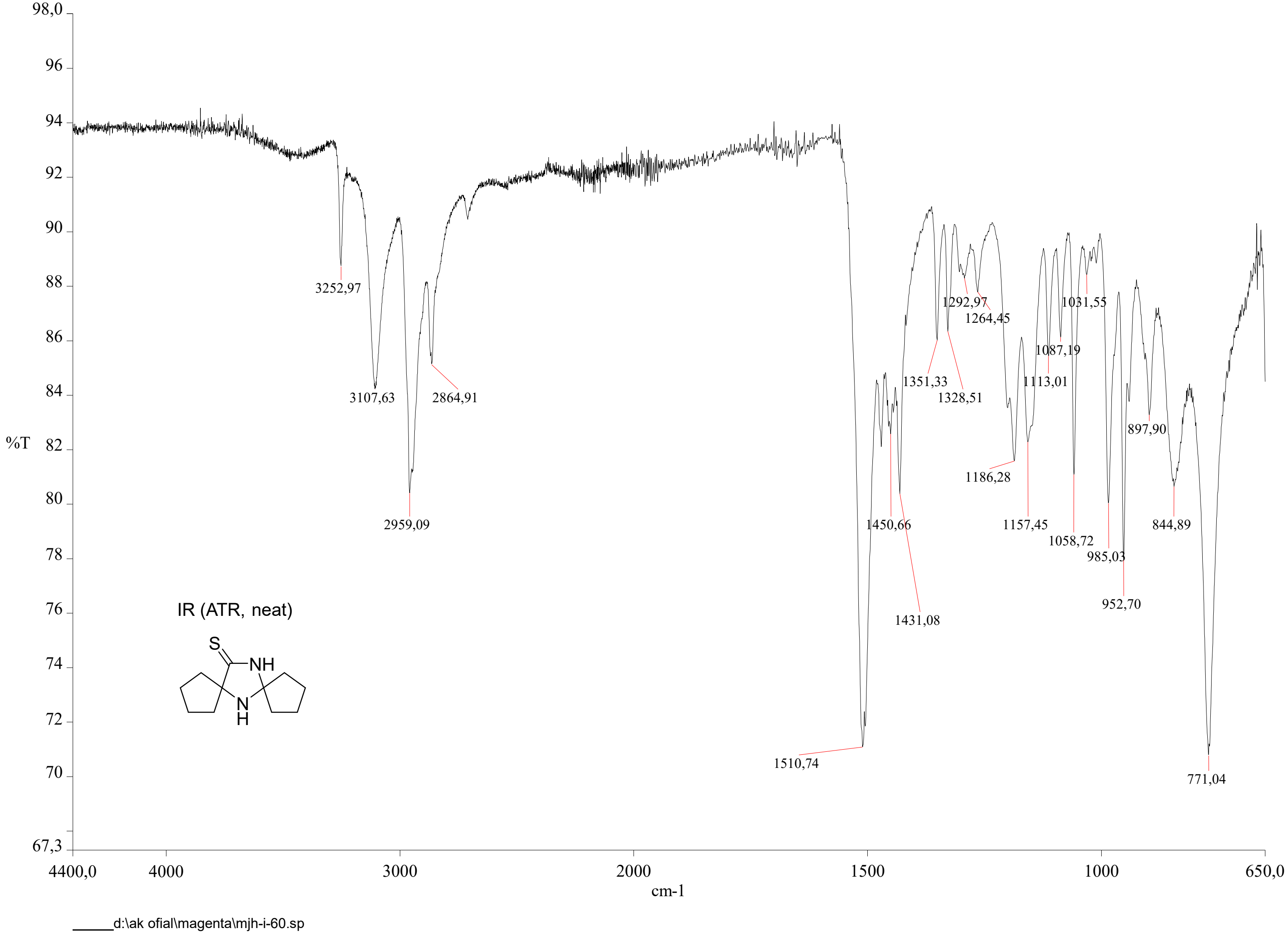

+**2,2,3,5,5-Pentamethyl-imidazolidine-4-thione** (**2d**) (MJH-I-170//MJH-II-19)

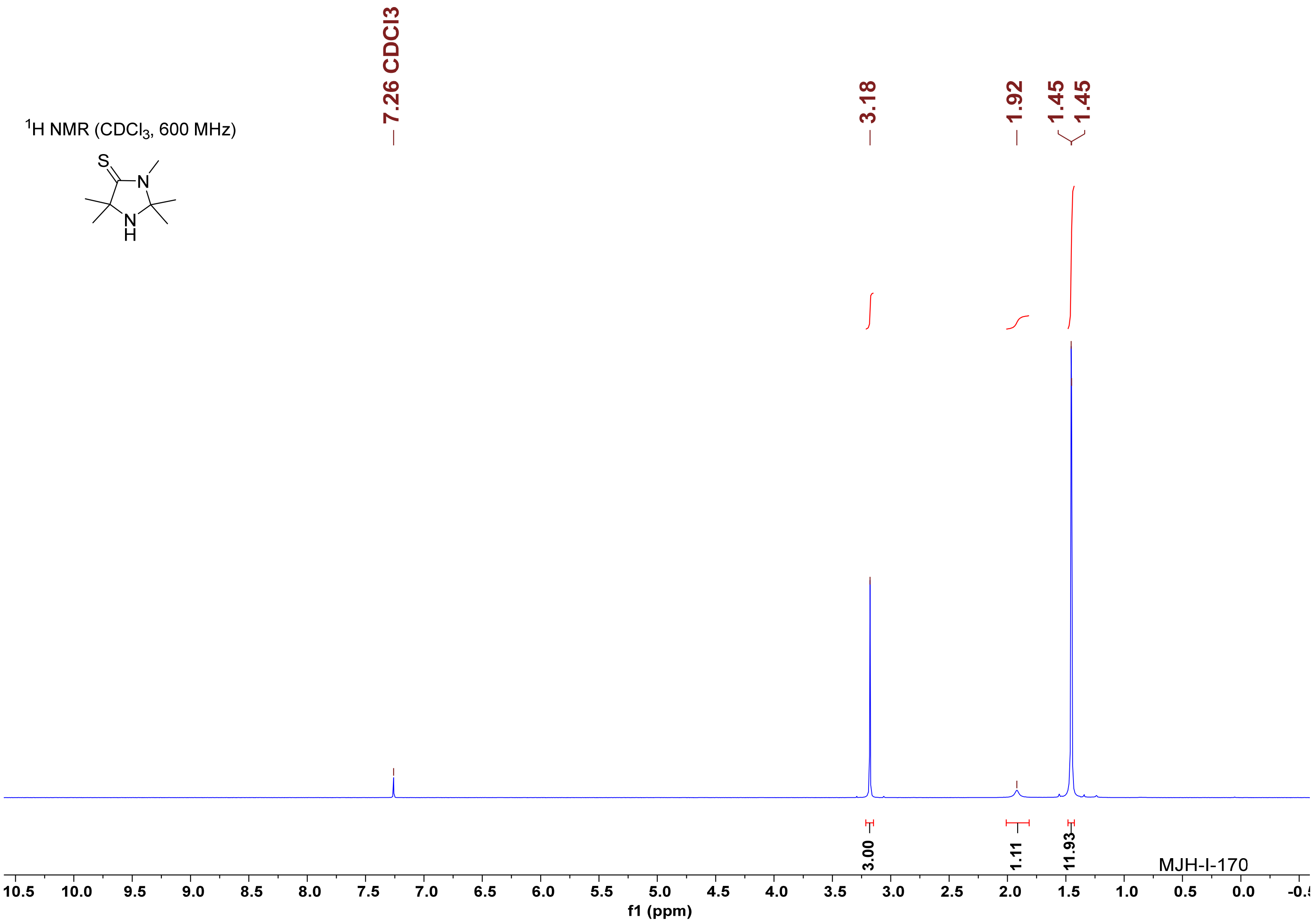

2,2,3,5,5-Pentamethyl-imidazolidine-4-thione (**2d**) (MJH-I-170//MJH-II-19)

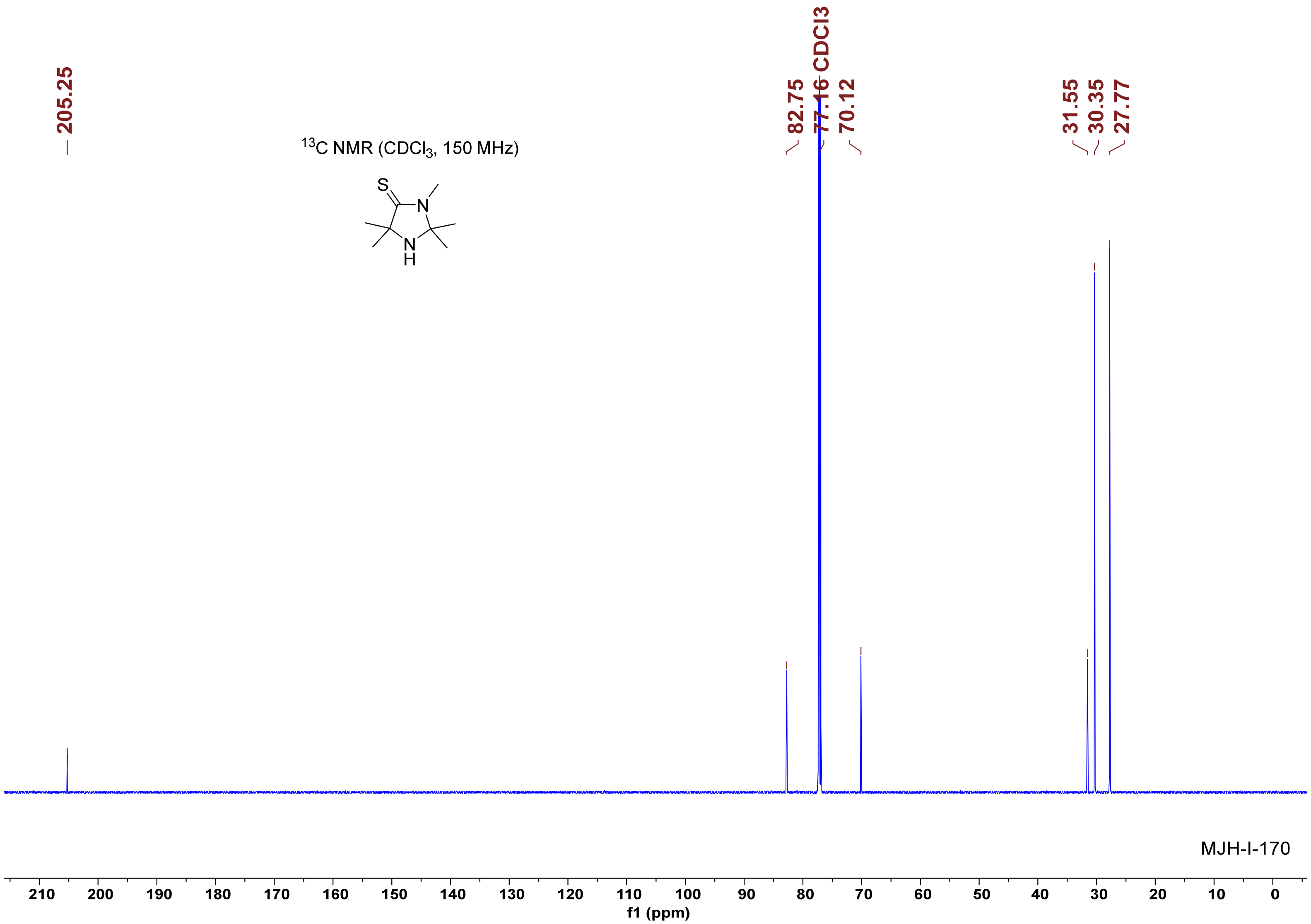

2,2,3,5,5-Pentamethyl-imidazolidine-4-thione (2d) (MJH-I-170//MJH-II-19)

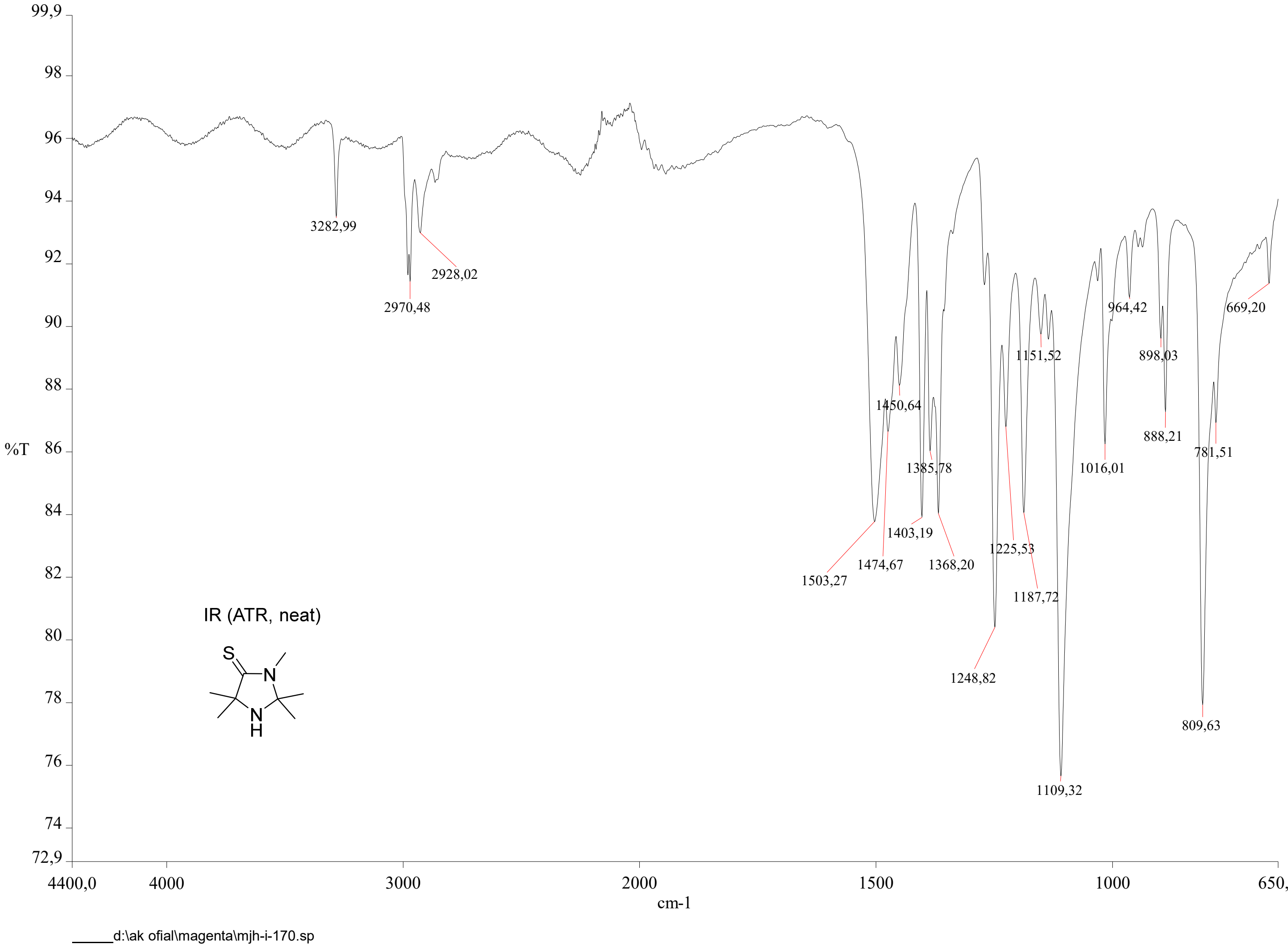

4-(Ethylthio)-2,2,5,5-tetramethyl-2,5-dihydro-1*H*-imidazole (3b) (MJH-I-14)

^{1}H NMR ($CDCl_3$, 400 MHz)

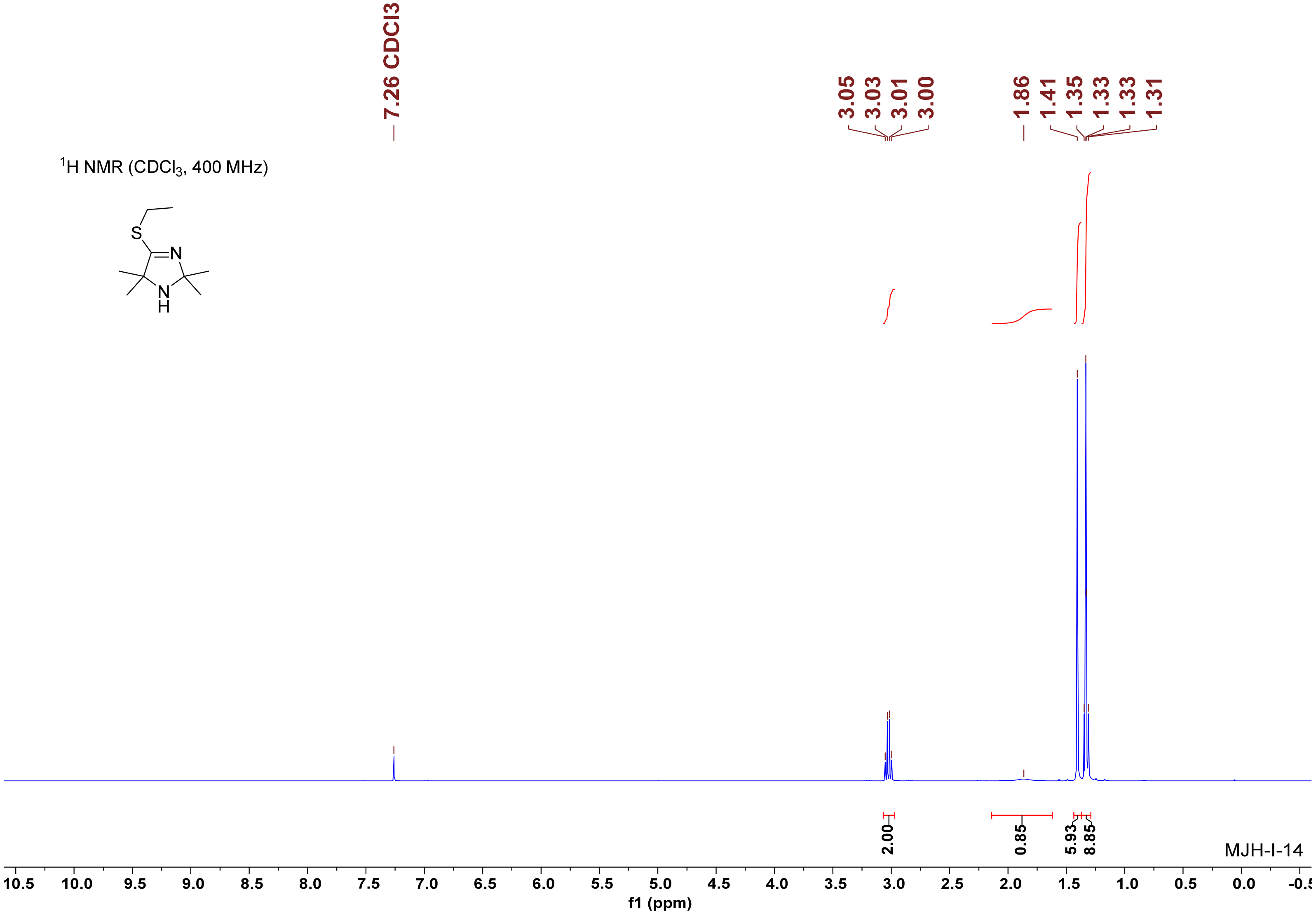

4-(Ethylthio)-2,2,5,5-tetramethyl-2,5-dihydro-1*H*-imidazole (3b) (MJH-I-14)

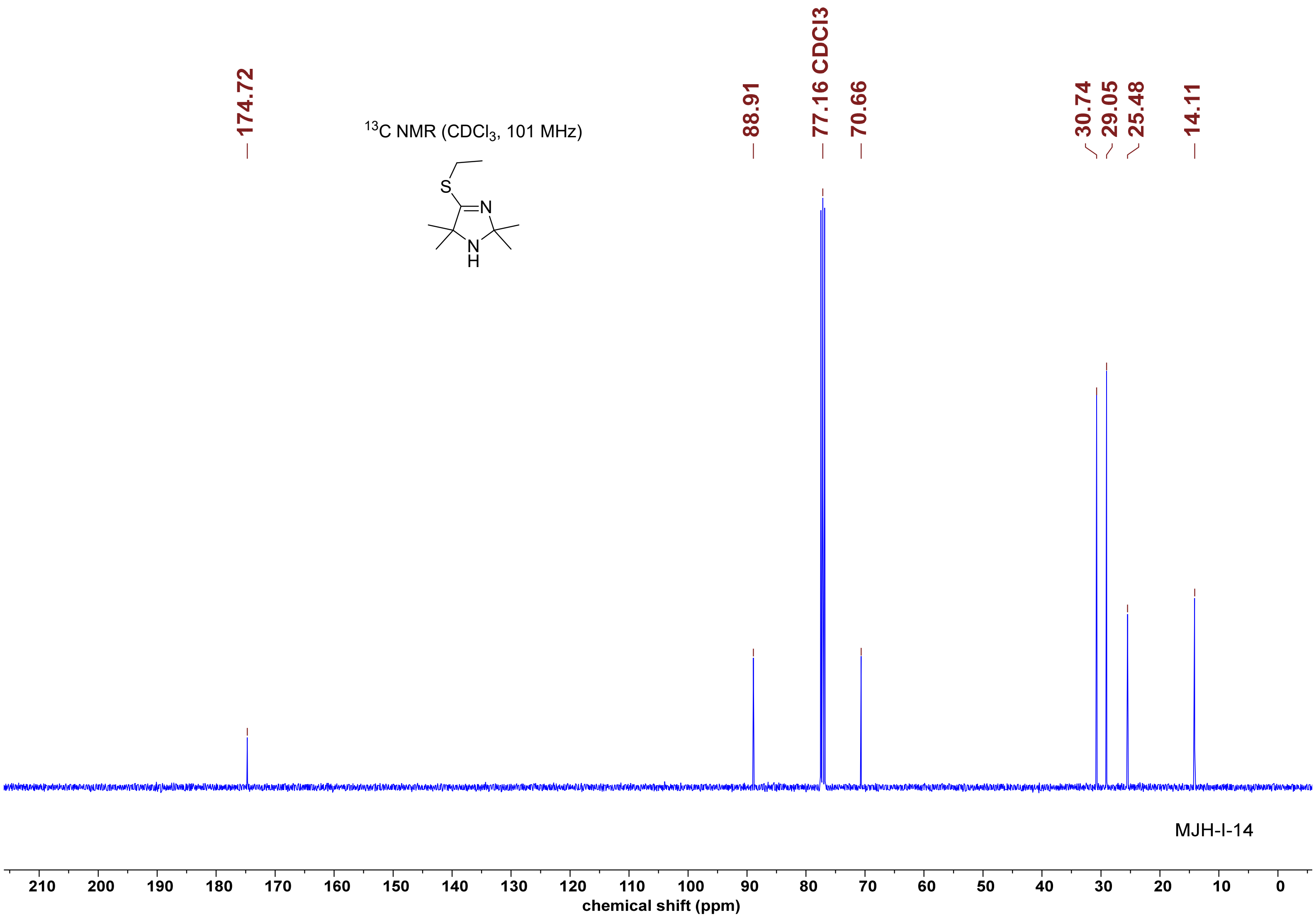

4-(Ethylthio)-2,2,5,5-tetramethyl-2,5-dihydro-1*H*-imidazole (**3b**) (MJH-I-14)

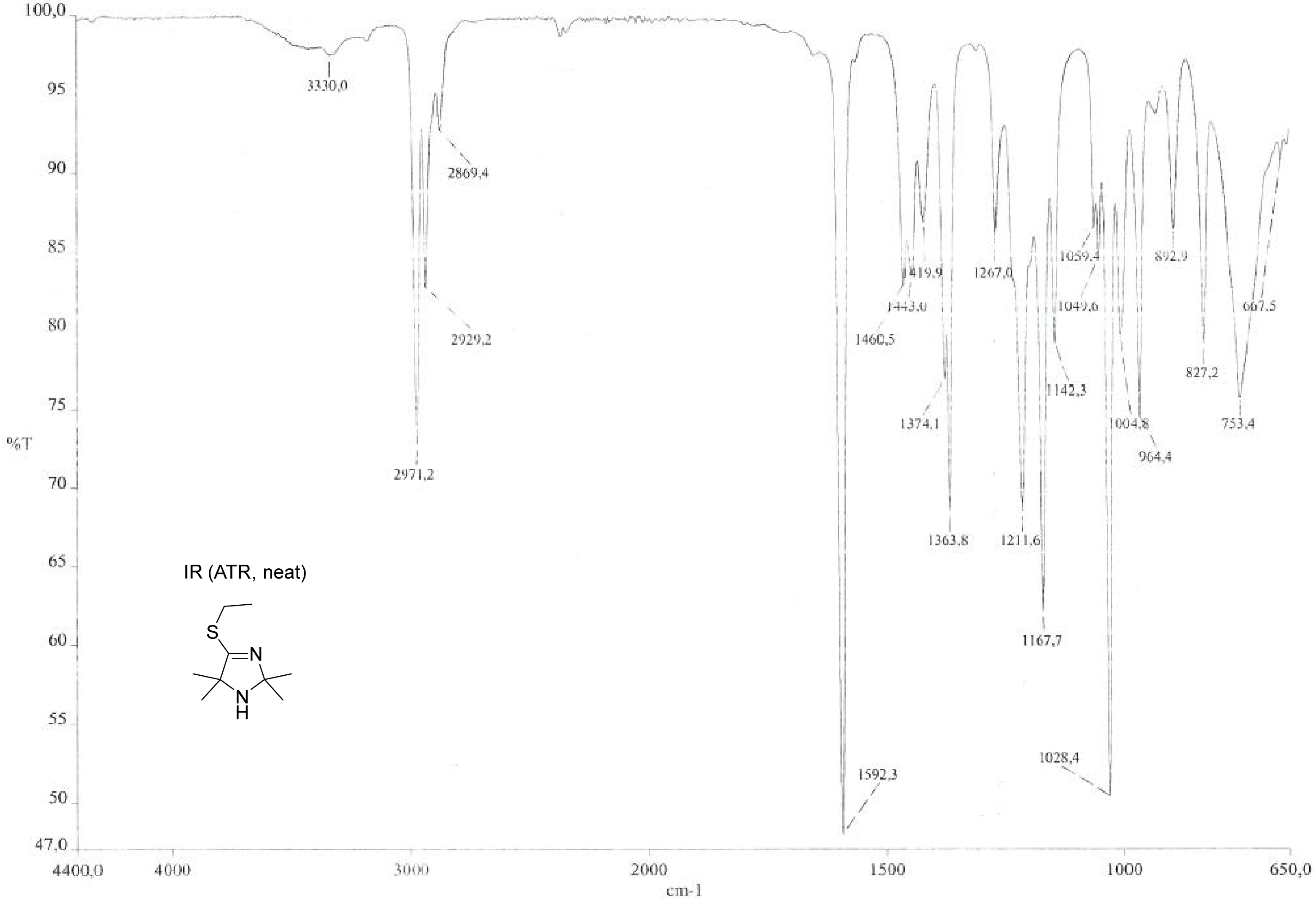

4-(Benzylthio)-2,2,5,5-tetramethyl-2,5-dihydro-1*H*-imidazole (3c) (MJH-I-76)

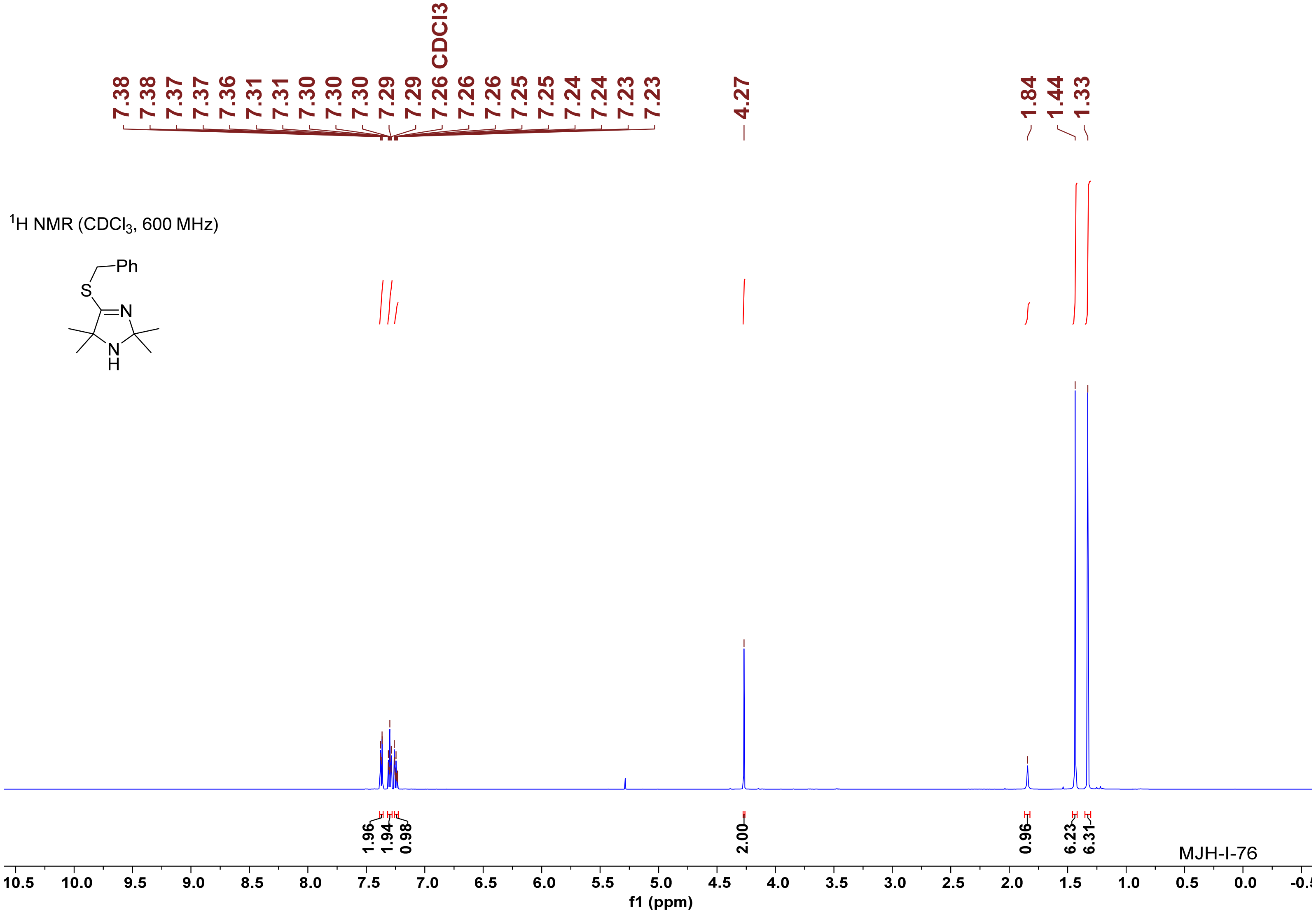

4-(Benzylthio)-2,2,5,5-tetramethyl-2,5-dihydro-1*H*-imidazole (3c) (MJH-I-76)

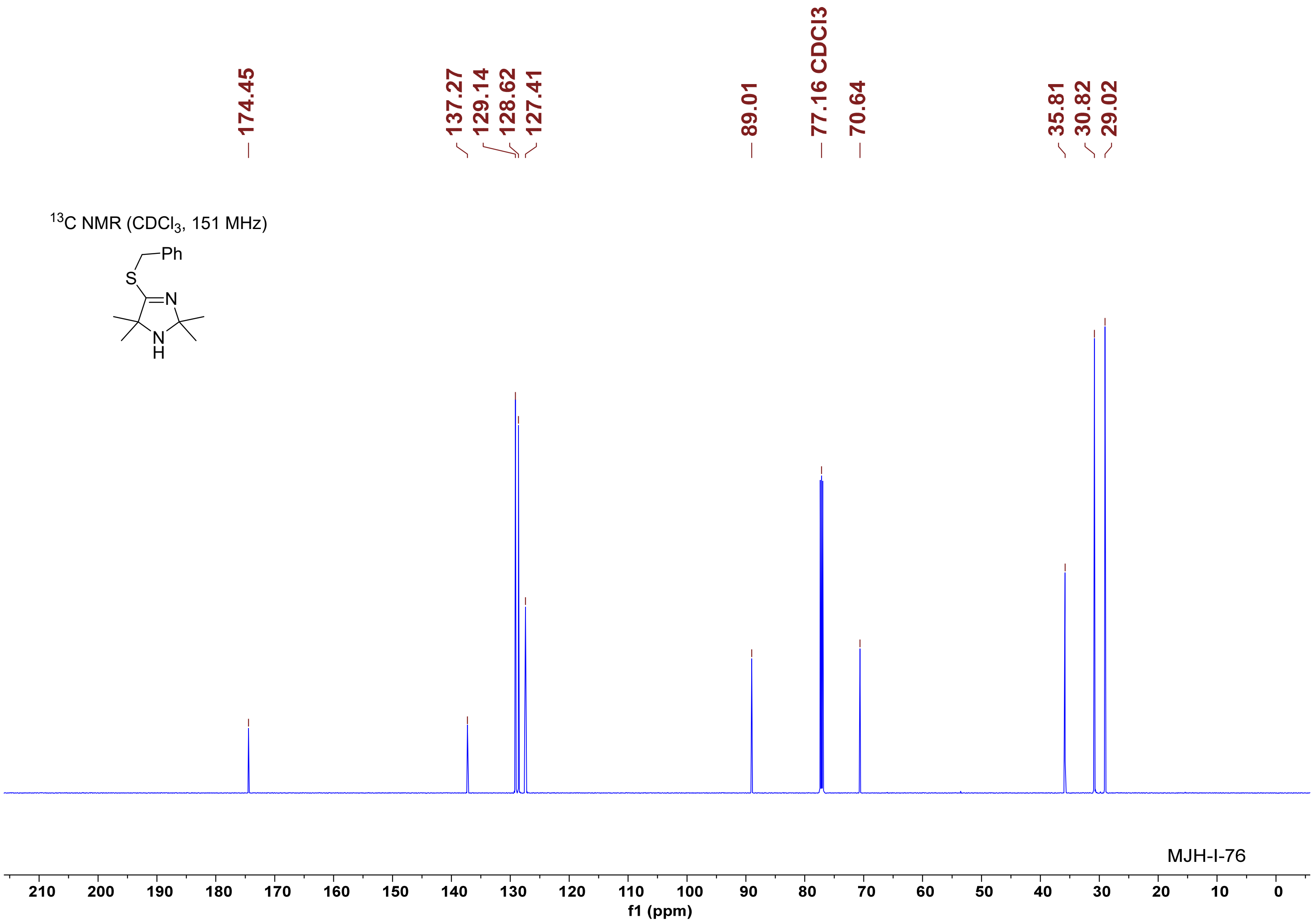

4-(Benzylthio)-2,2,5,5-tetramethyl-2,5-dihydro-1*H*-imidazole (3c) (MJH-I-76)

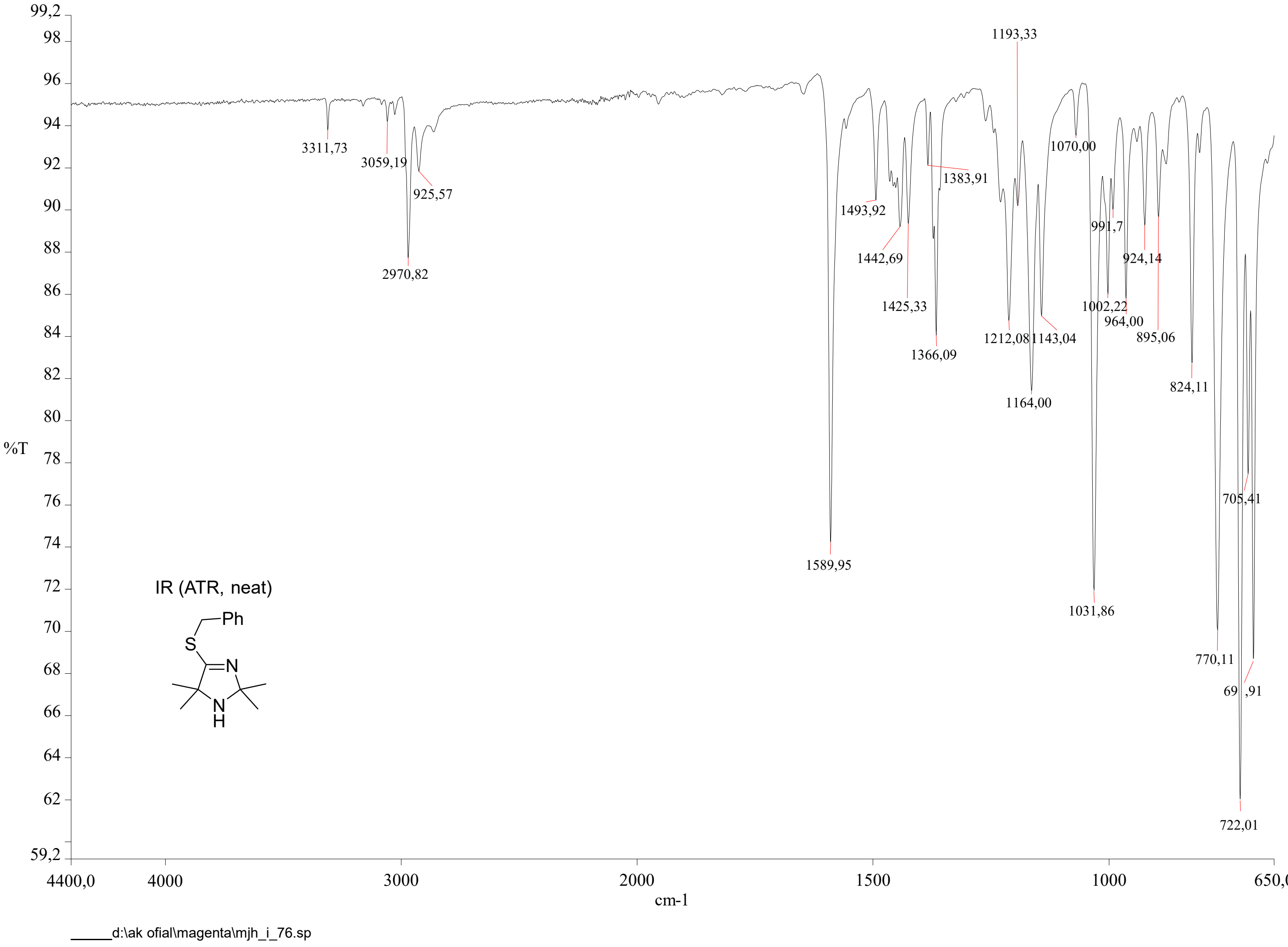

13-(Ethylthio)-6,12-diazadispiro[4.1.4^7.2^5]tridec-12-ene (3d) (MJH-I-61)

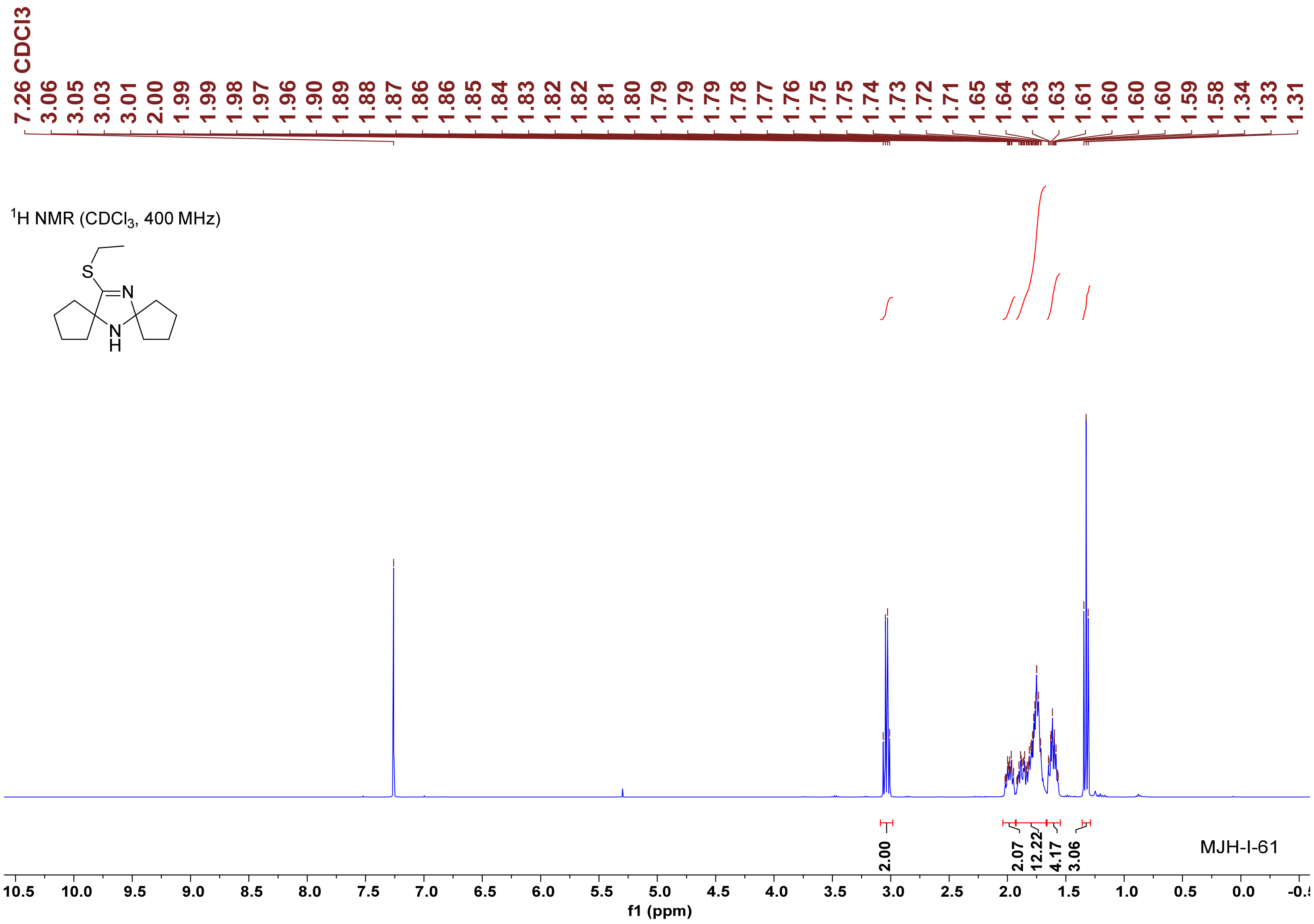

13-(Ethylthio)-6,12-diazadispiro[4.1.4^7.2^5]tridec-12-ene (3d) (MJH-I-61)

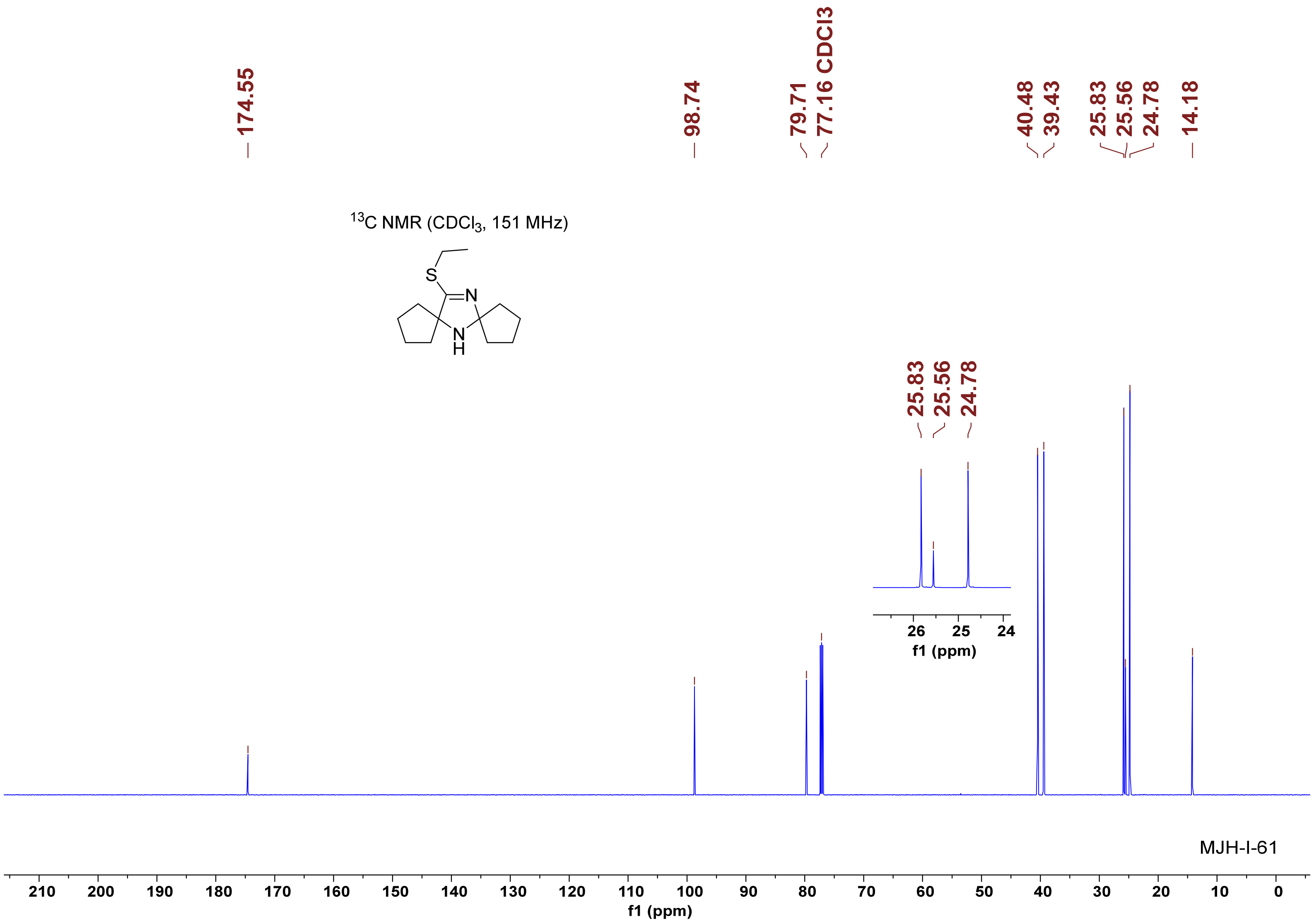

13-(Ethylthio)-6,12-diazadispiro[4.1.4^7.2^5]tridec-12-ene (3d) (MJH-I-61)

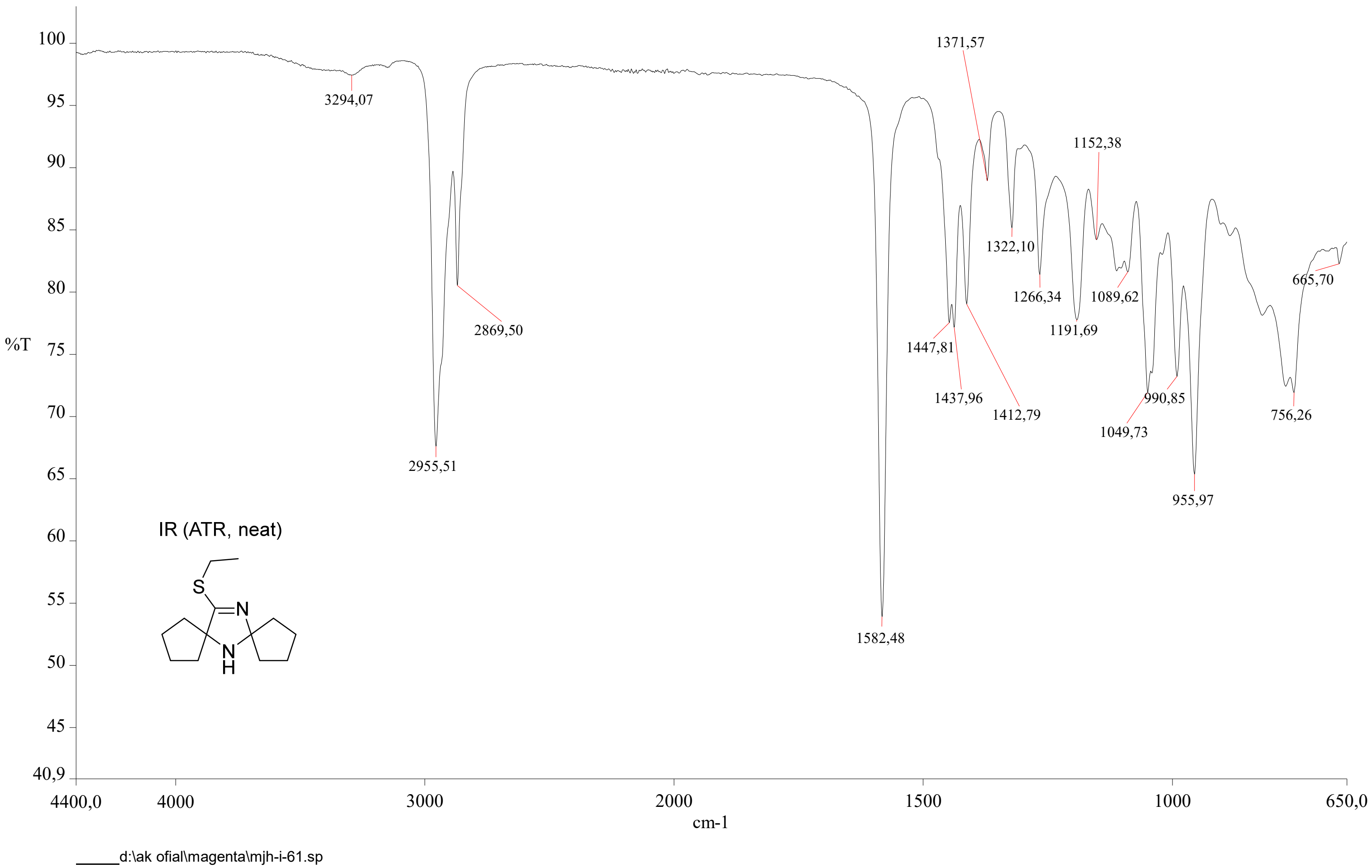

(*E*)-2-((1-(But-1-en-1-yl)-2,2,5,5-tetramethyl-2,5-dihydro-1*H*-imidazol-4-yl)thio)acetonitrile (4b) (MJH-I-127)

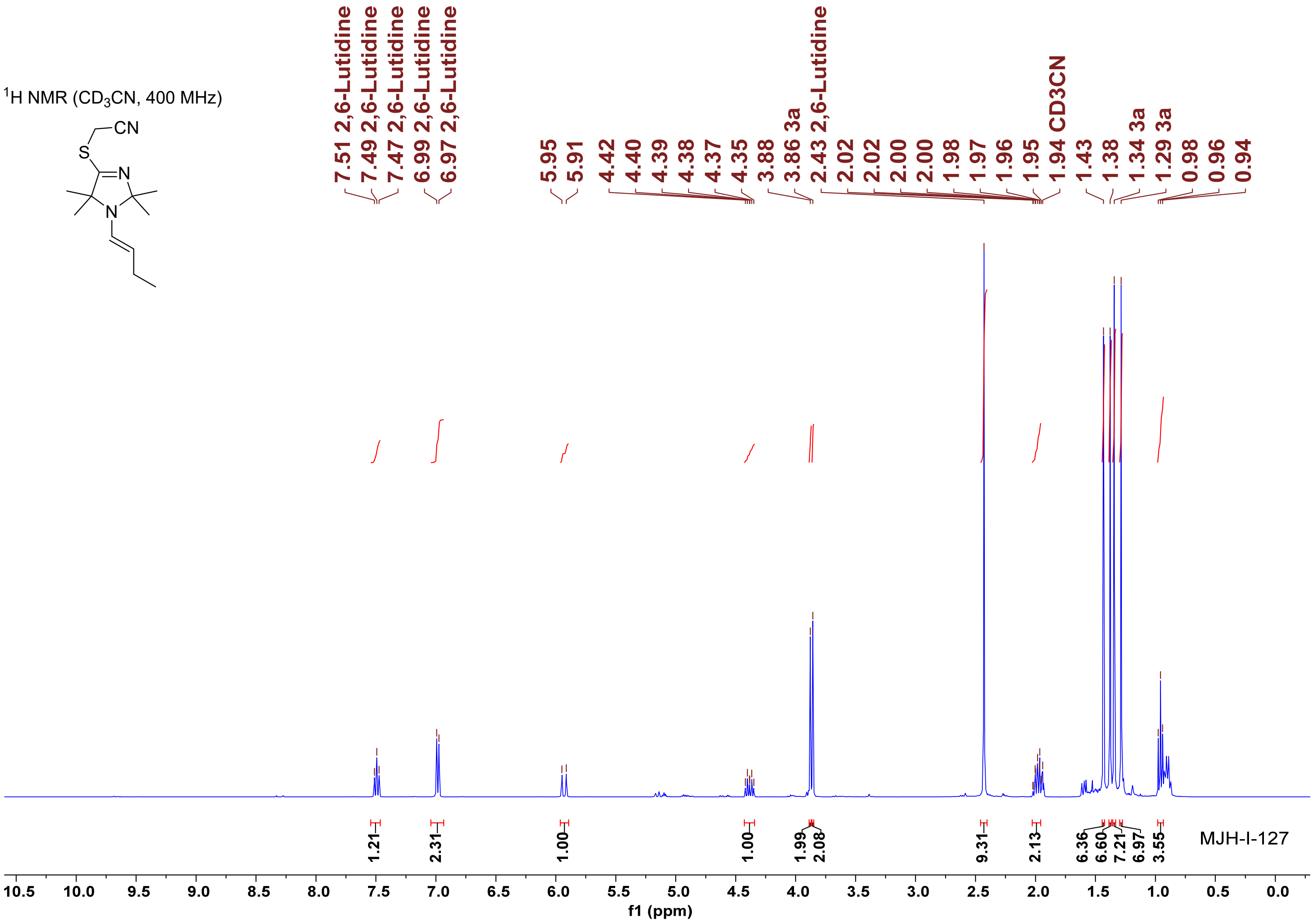

(*E*)-2-((1-(But-1-en-1-yl)-2,2,5,5-tetramethyl-2,5-dihydro-1*H*-imidazol-4-yl)thio)acetonitrile (4b) (MJH-I-127)

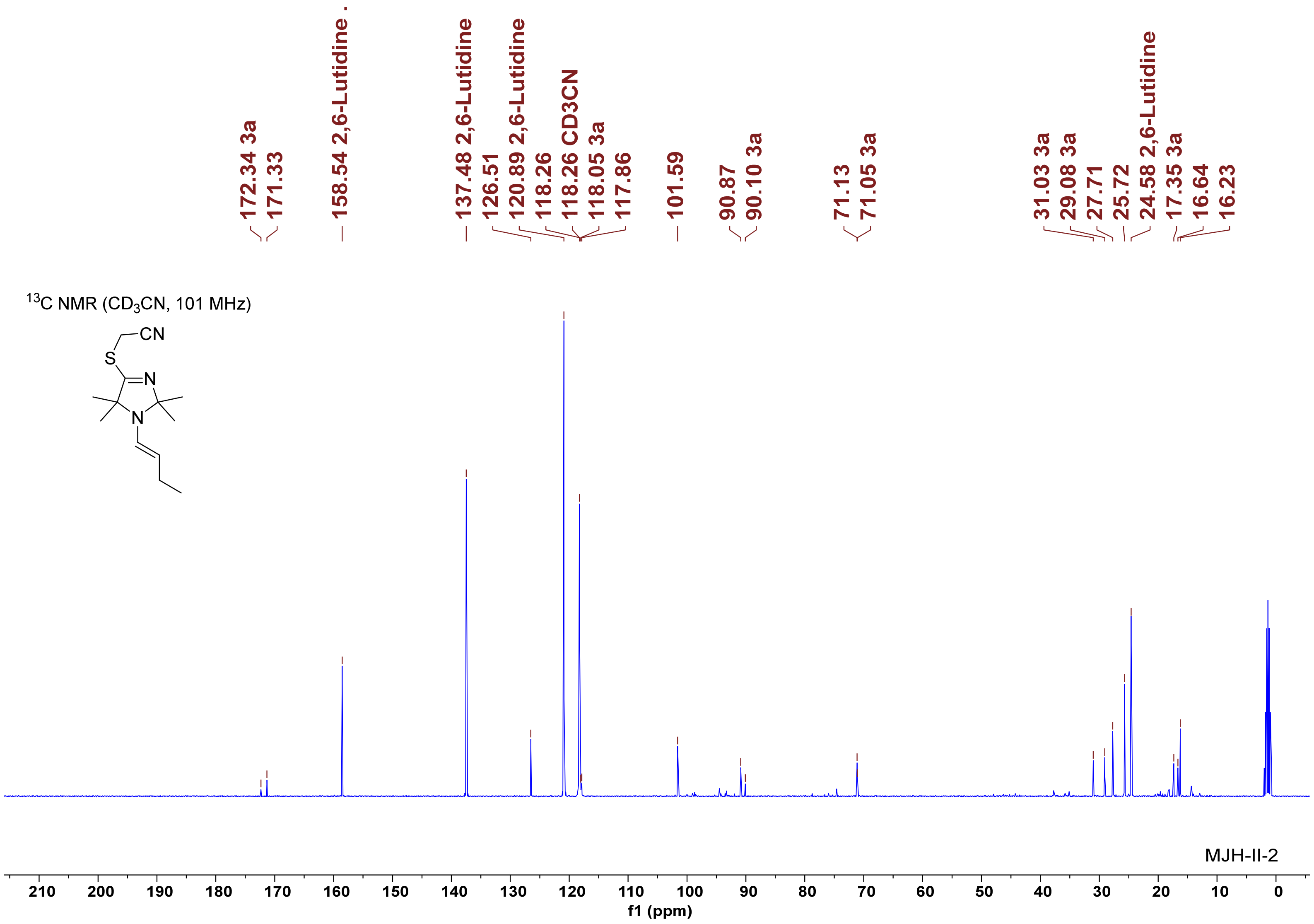

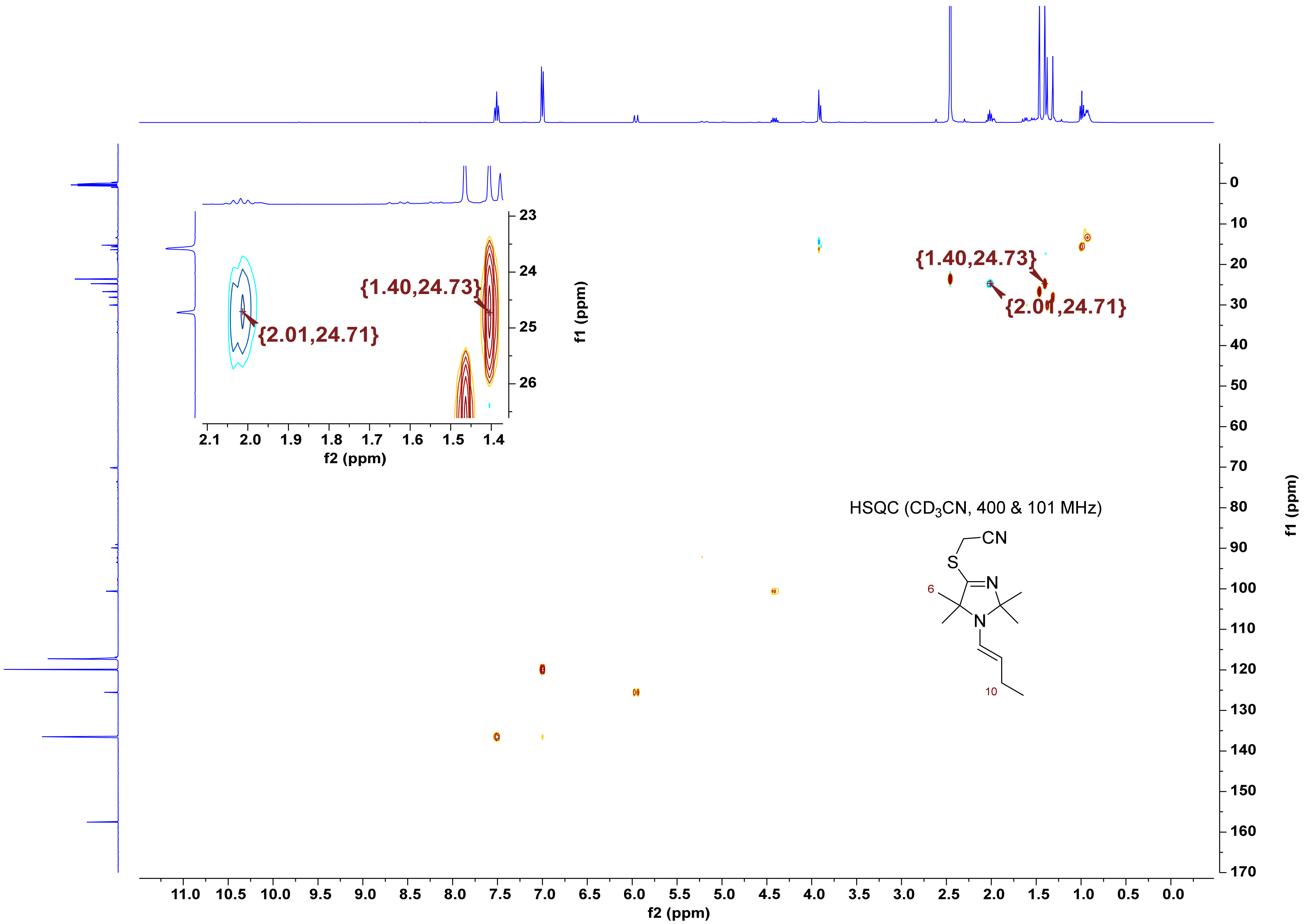
{1.40,24.73}
{2.01,24.71}
f2 (ppm)
f1 (ppm)
HSQC (CD3CN, 400 & 101 MHz)
CN
S
N
N
6
10

(*E*)-4-(Ethylthio)-2,2,5,5-tetramethyl-1-styryl-2,5-dihydro-1*H*-imidazole (4d) (MJH-I-39//MJH-I-22)

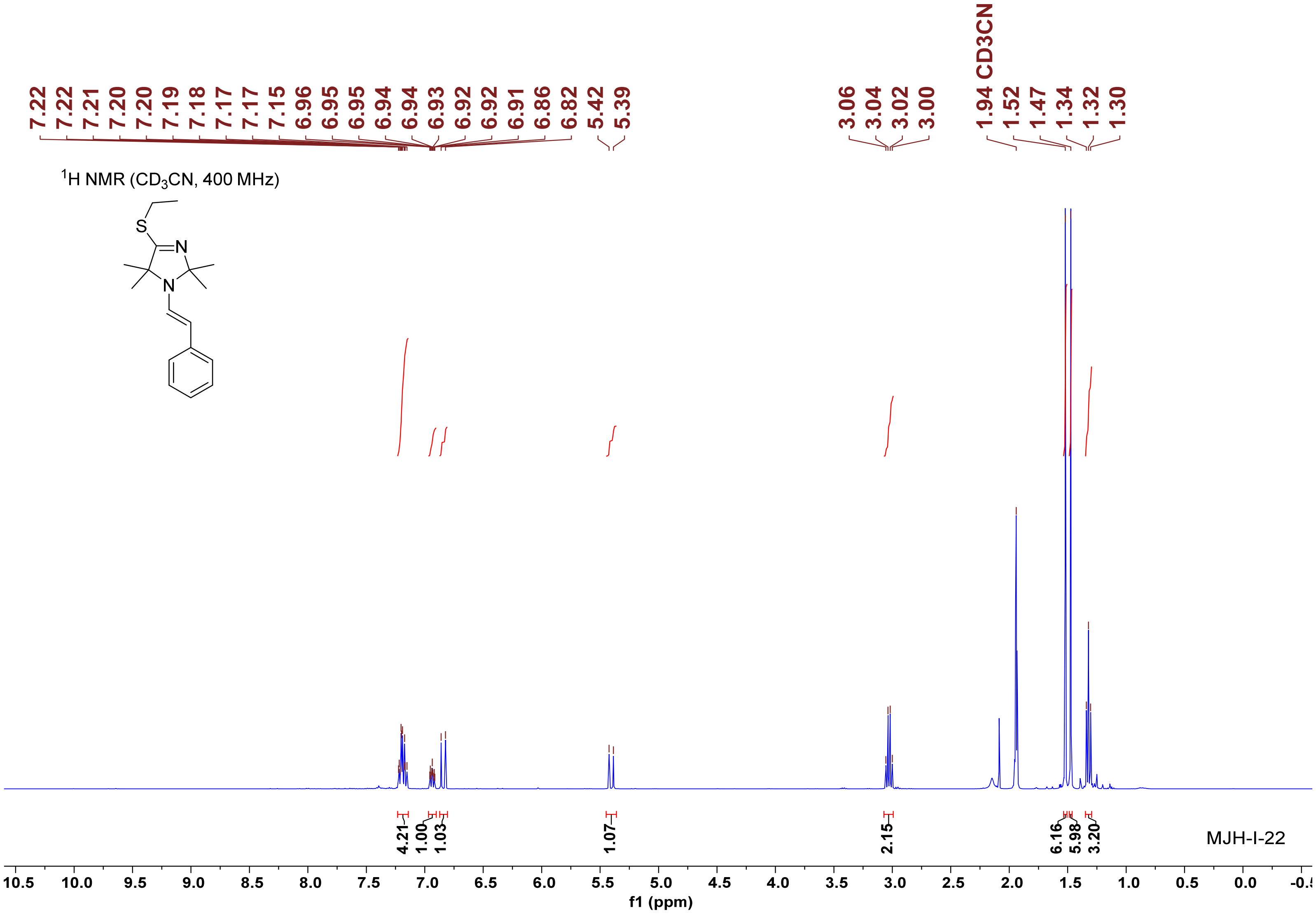

(*E*)-4-(Ethylthio)-2,2,5,5-tetramethyl-1-styryl-2,5-dihydro-1*H*-imidazole (4d) (MJH-I-39//MJH-I-22)

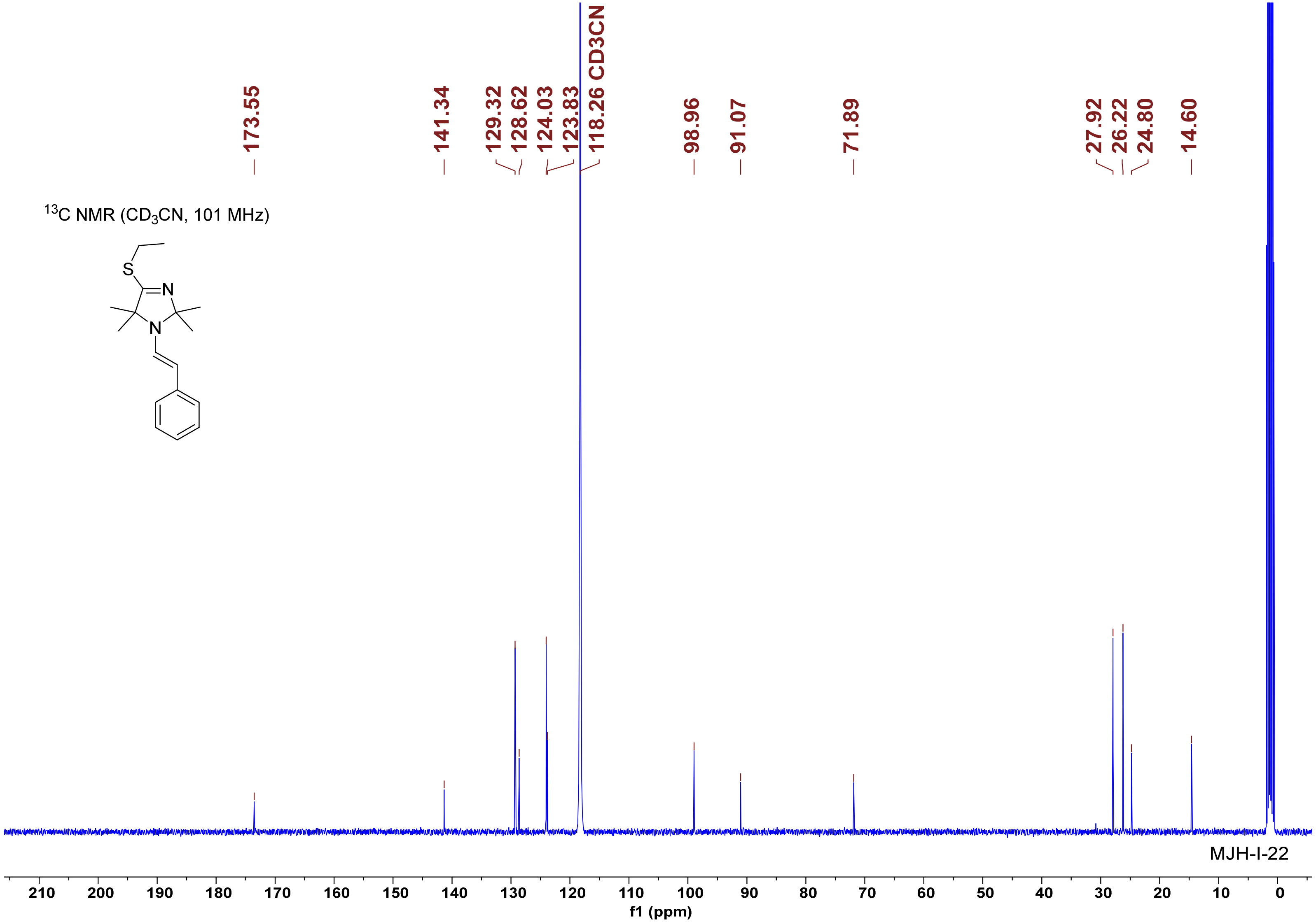

(*E*)-4-(Ethylthio)-2,2,5,5-tetramethyl-1-styryl-2,5-dihydro-1*H*-imidazole (4d) (MJH-I-39//MJH-I-22)

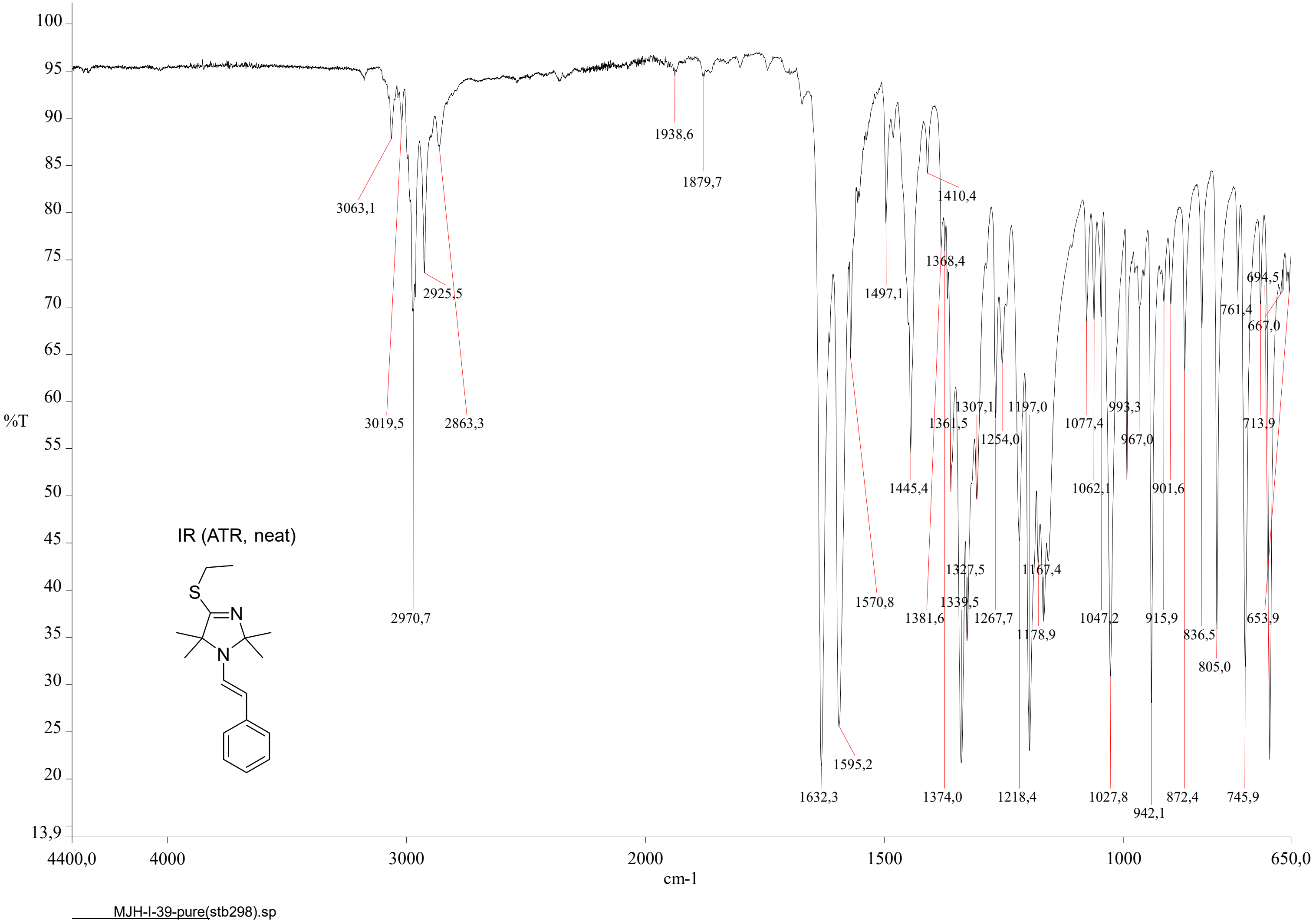

(*E*)-13-(Ethylthio)-6-styryl-6,12-diazadispiro[4.1.4[7].2[5]]tridec-12-ene (**4e**) (MJH-I-63, in $CDCl_3$)

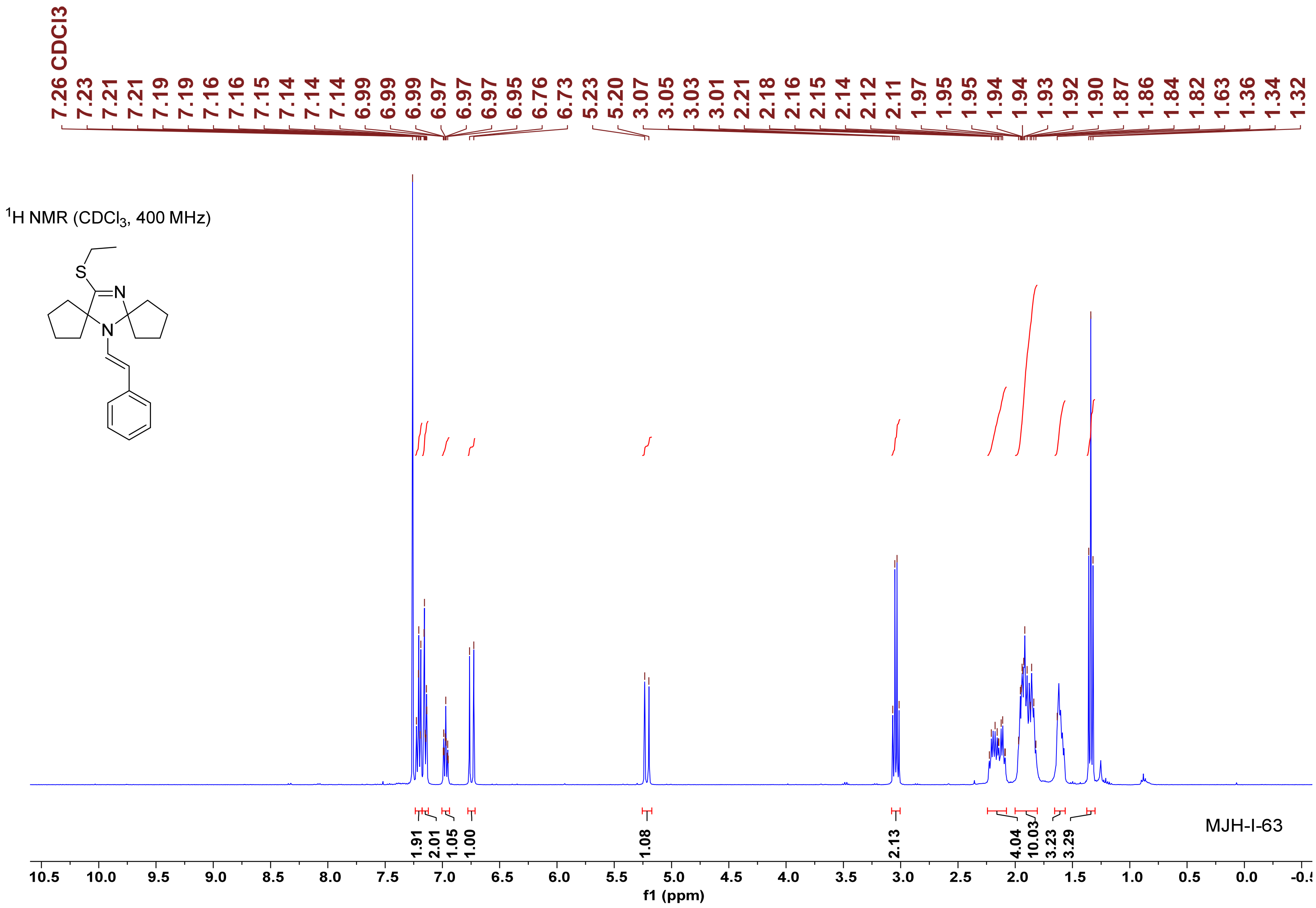

(*E*)-13-(Ethylthio)-6-styryl-6,12-diazadispiro[4.1.4⁷.2⁵]tridec-12-ene (4e) (MJH-I-168, in CD_3CN)

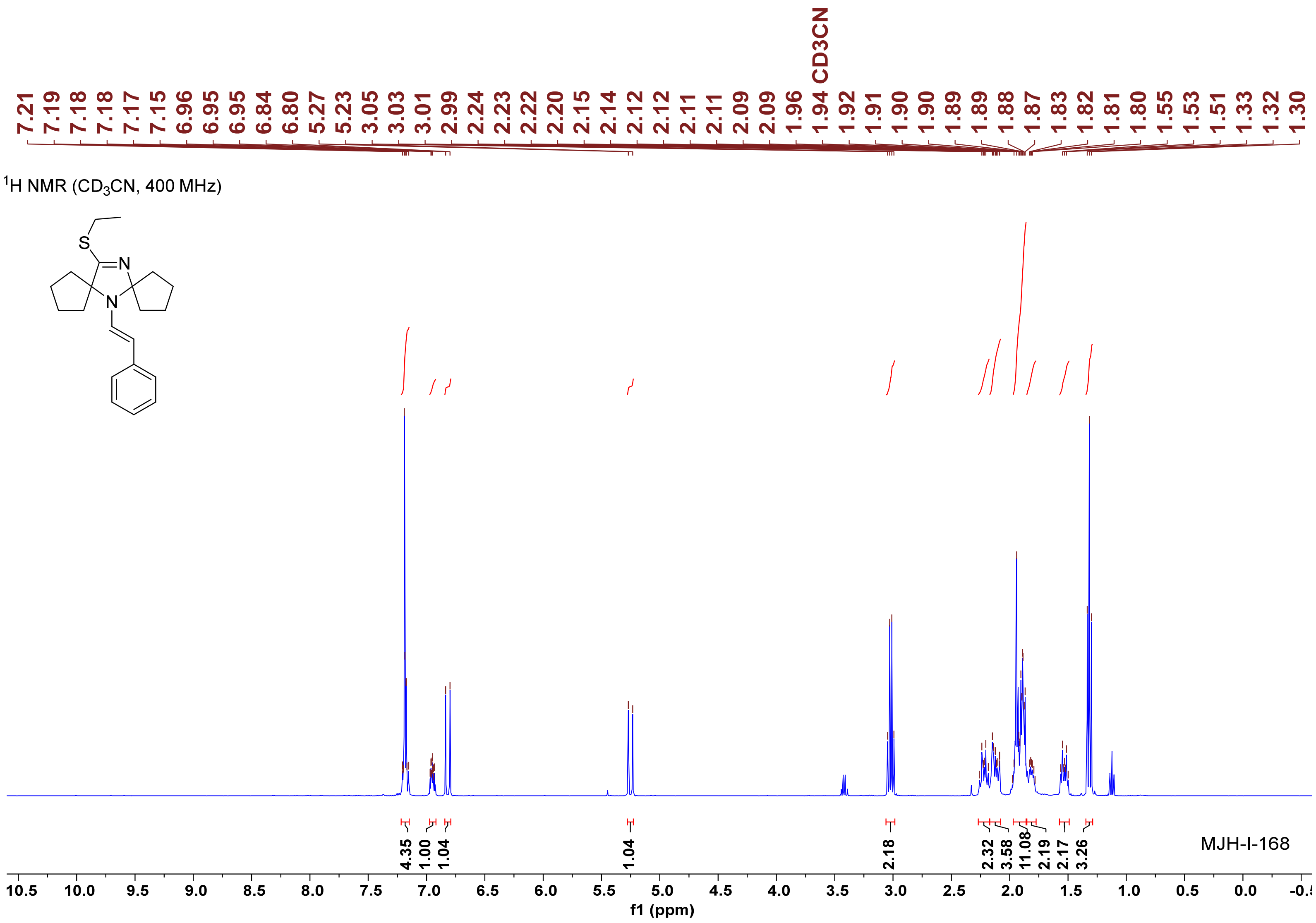

(*E*)-13-(Ethylthio)-6-styryl-6,12-diazadispiro[4.1.4^7.2^5]tridec-12-ene (4e) (MJH-I-63, in CD_3CN)

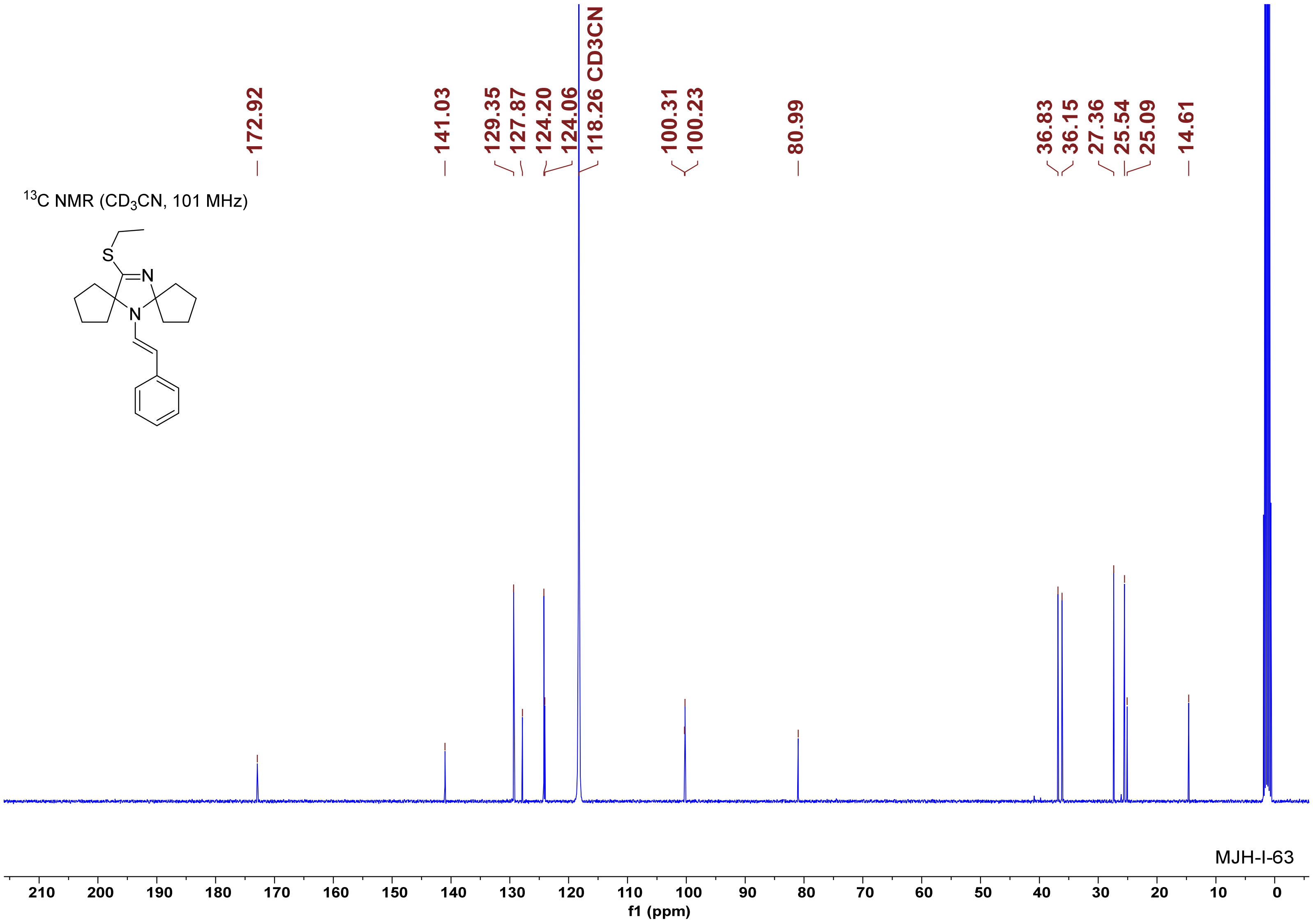

(*E*)-13-(Ethylthio)-6-styryl-6,12-diazadispiro[4.1.4^{7}.2^{5}]tridec-12-ene (4e) (MJH-I-63)

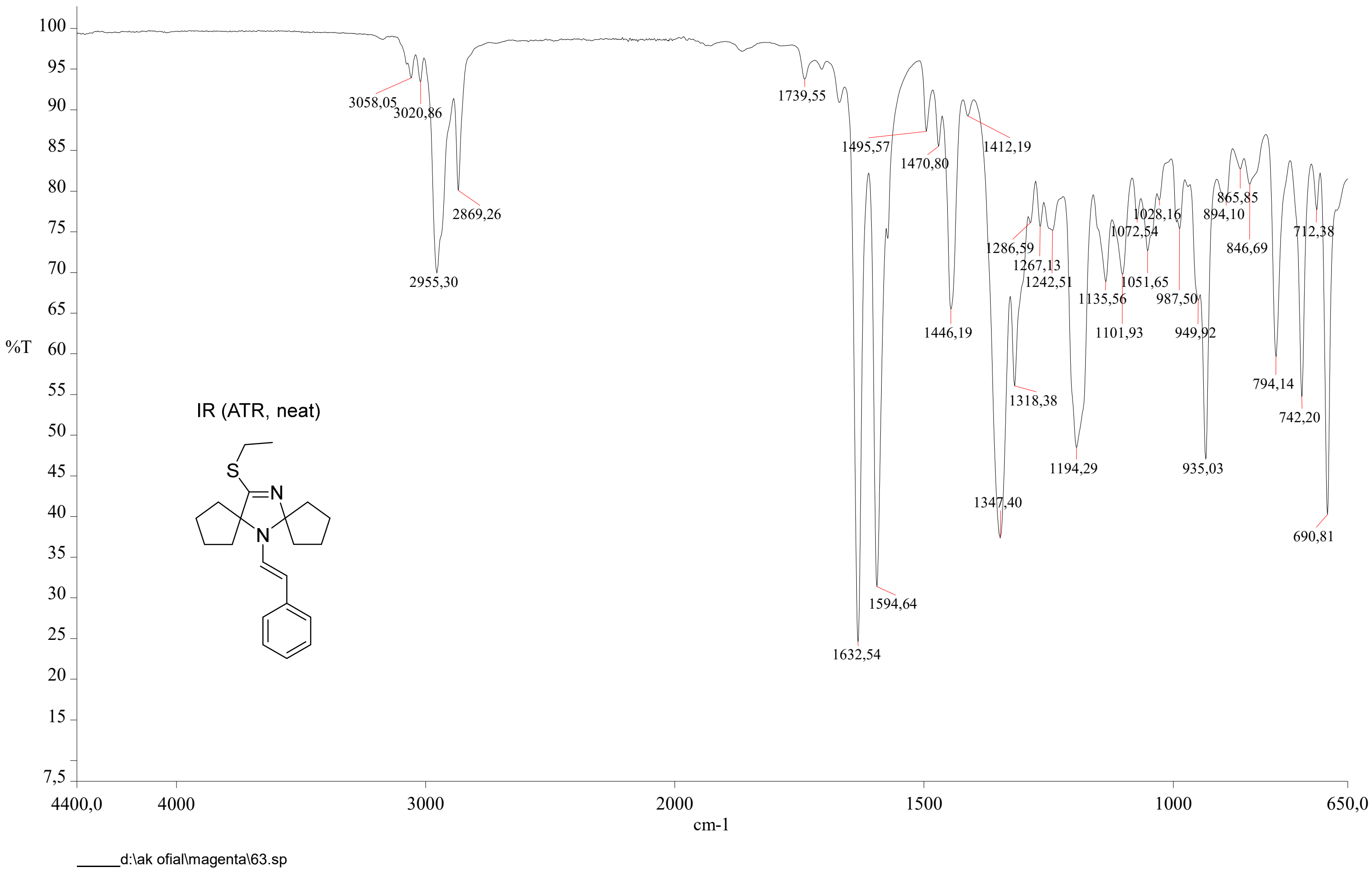

(*E*)-4-(Ethylthio)-2,2,5,5-tetramethyl-1-(3-phenylprop-1-en-1-yl)-2,5-dihydro-1H-imidazole (4f) (MJH-II-41)

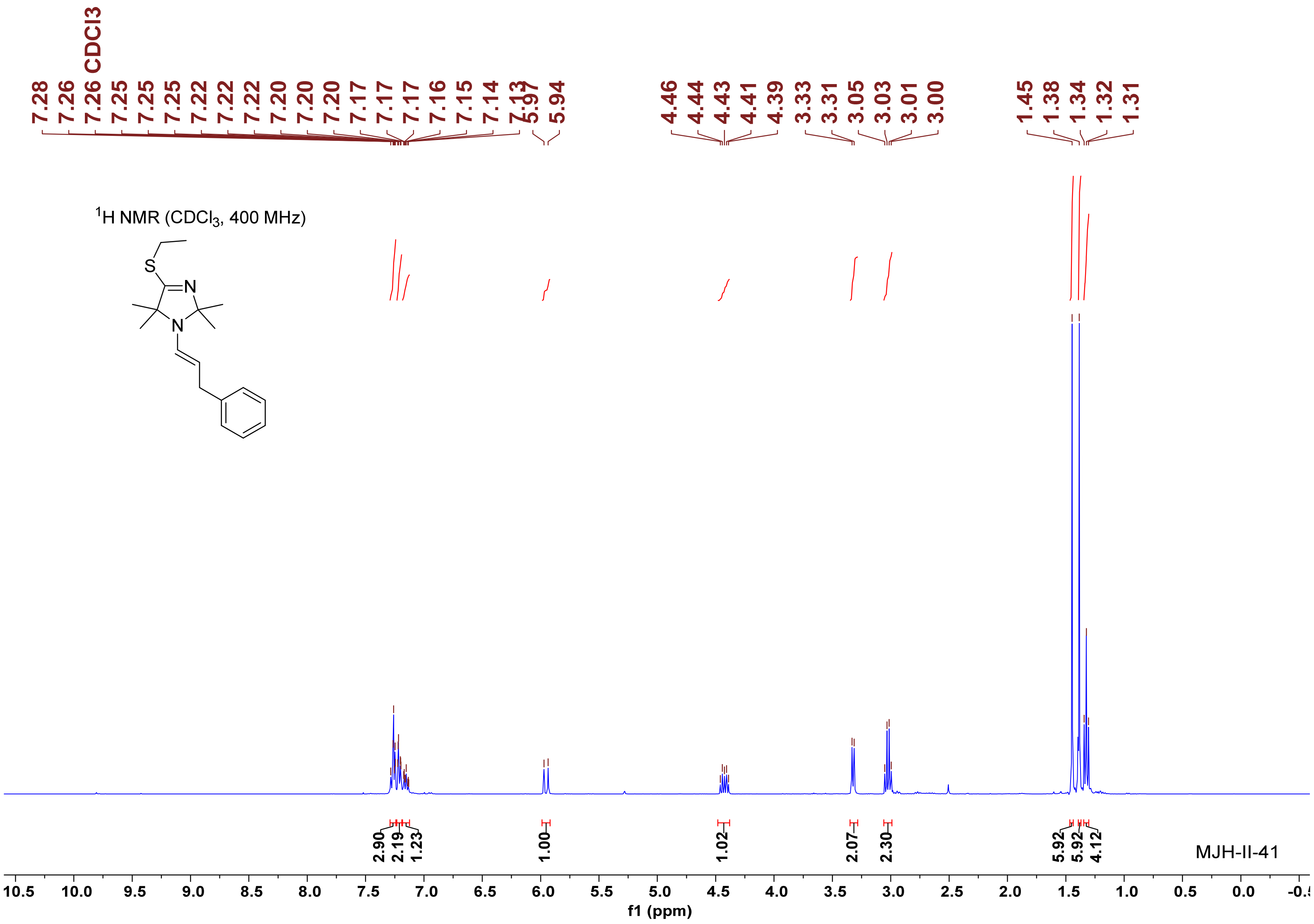

(*E*)-4-(Ethylthio)-2,2,5,5-tetramethyl-1-(3-phenylprop-1-en-1-yl)-2,5-dihydro-1H-imidazole (4f) (MJH-II-41)

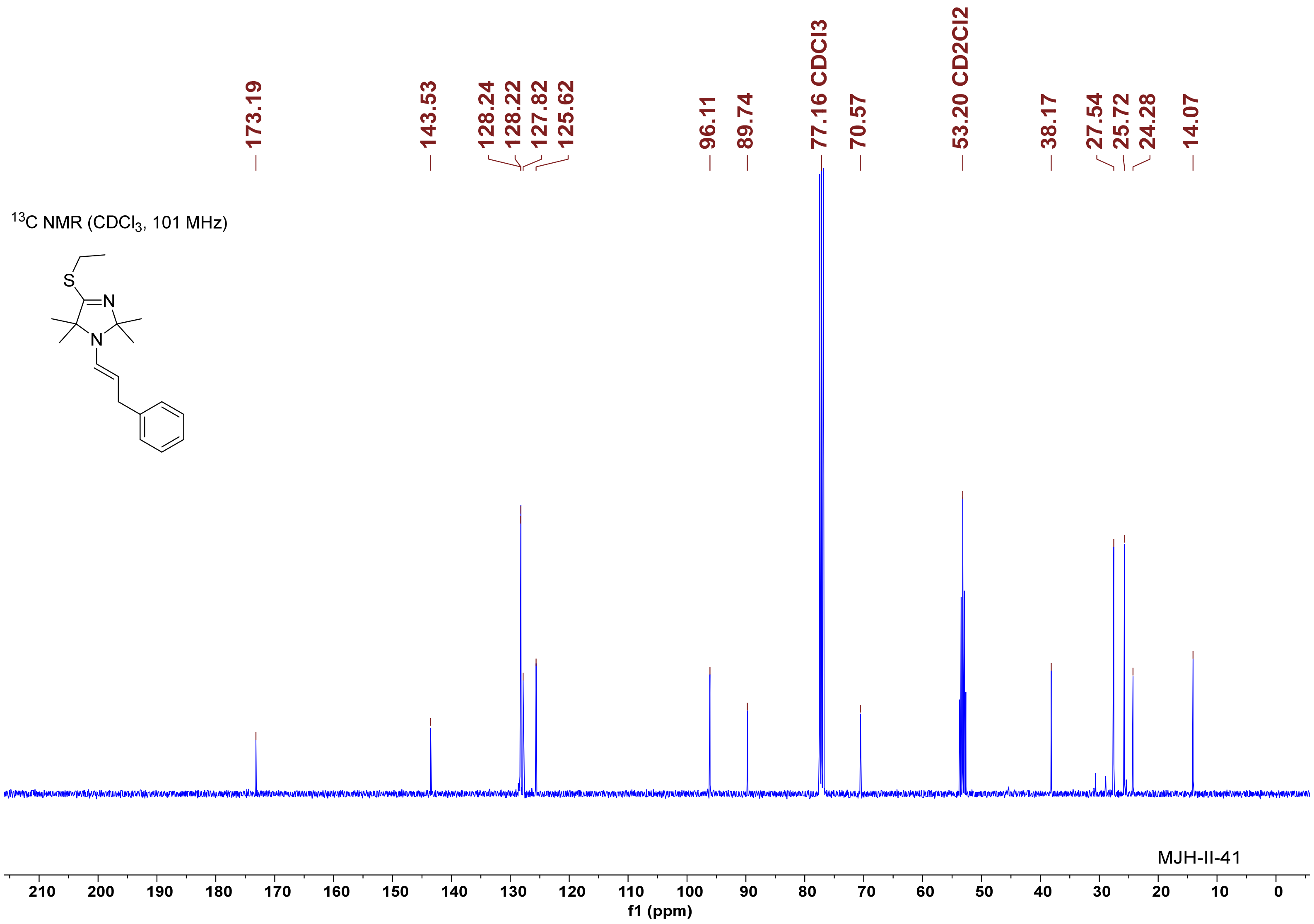

(*E*)-4-(Ethylthio)-2,2,5,5-tetramethyl-1-(3-phenylprop-1-en-1-yl)-2,5-dihydro-1H-imidazole (4f) (MJH-II-41)

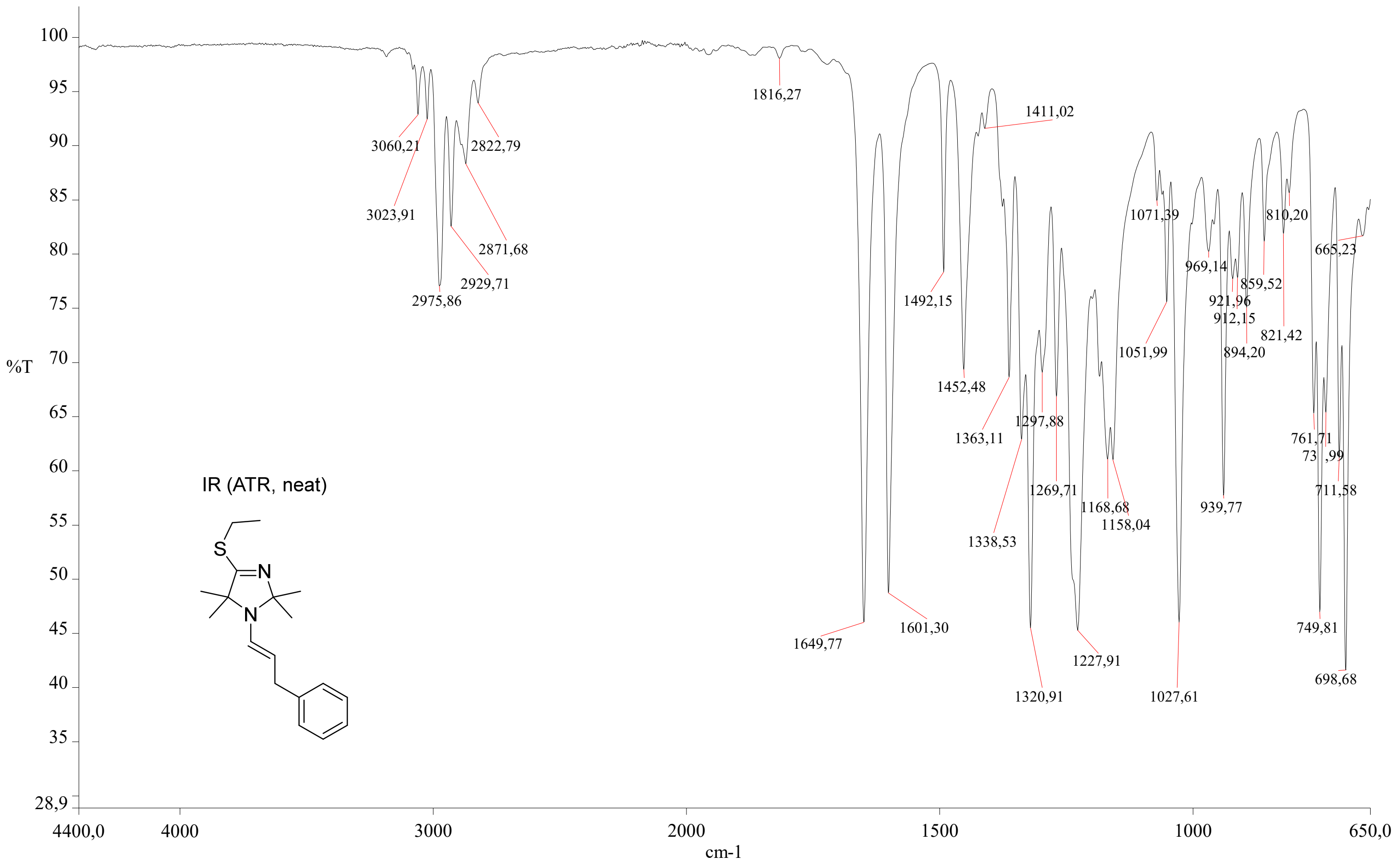

d:\ak ofial\magenta\mjh-ii-41-redo.sp

(*E*)-4-(Ethylthio)-2,2,5,5-tetramethyl-1-(3-phenylbut-1-en-1-yl)-2,5-dihydro-1H-imidazole (**4g**) (MJH-II-50)

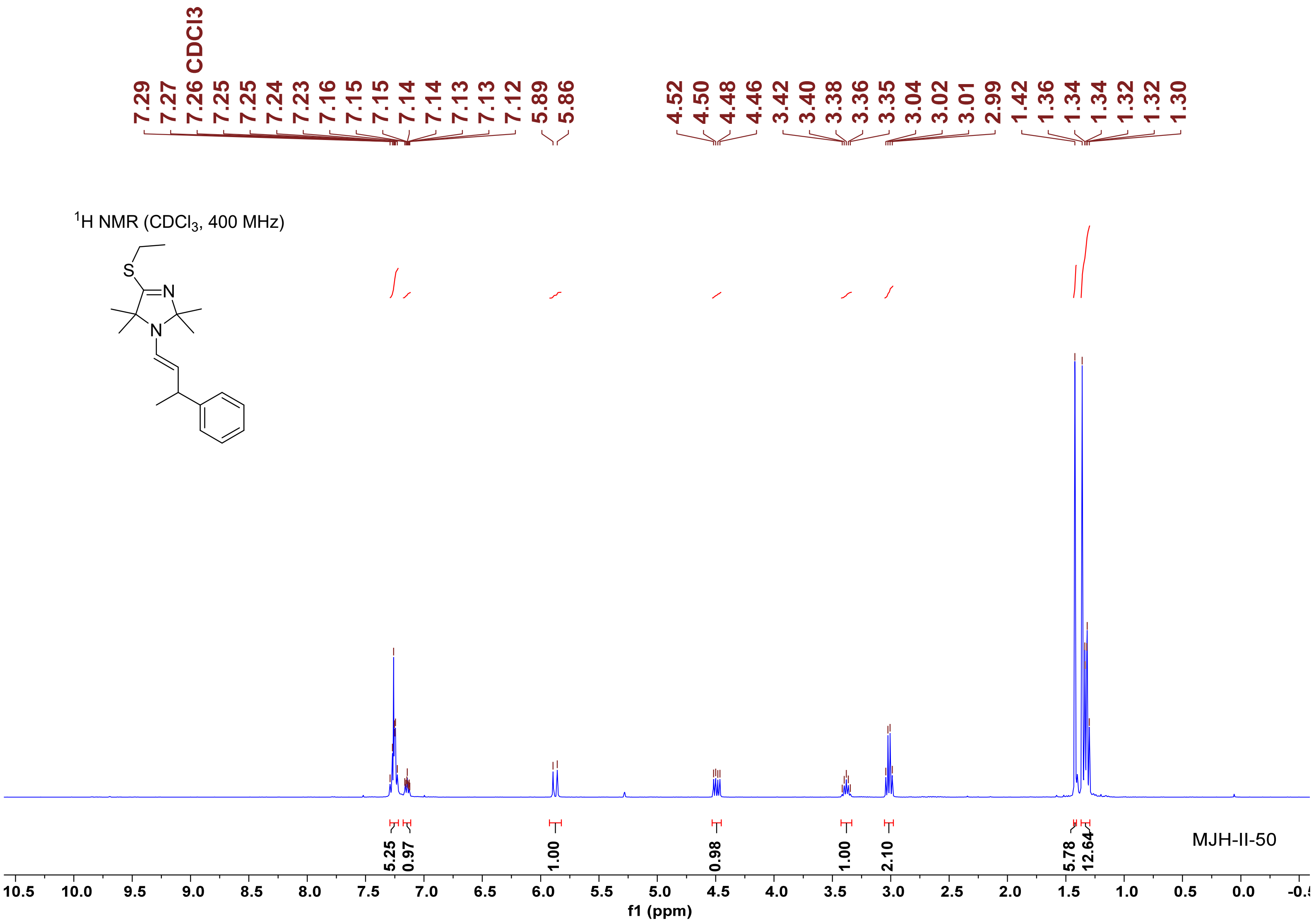

(*E*)-4-(Ethylthio)-2,2,5,5-tetramethyl-1-(3-phenylbut-1-en-1-yl)-2,5-dihydro-1H-imidazole (**4g**) (MJH-II-50)

^{13}C NMR ($CDCl_3$, 101 MHz)

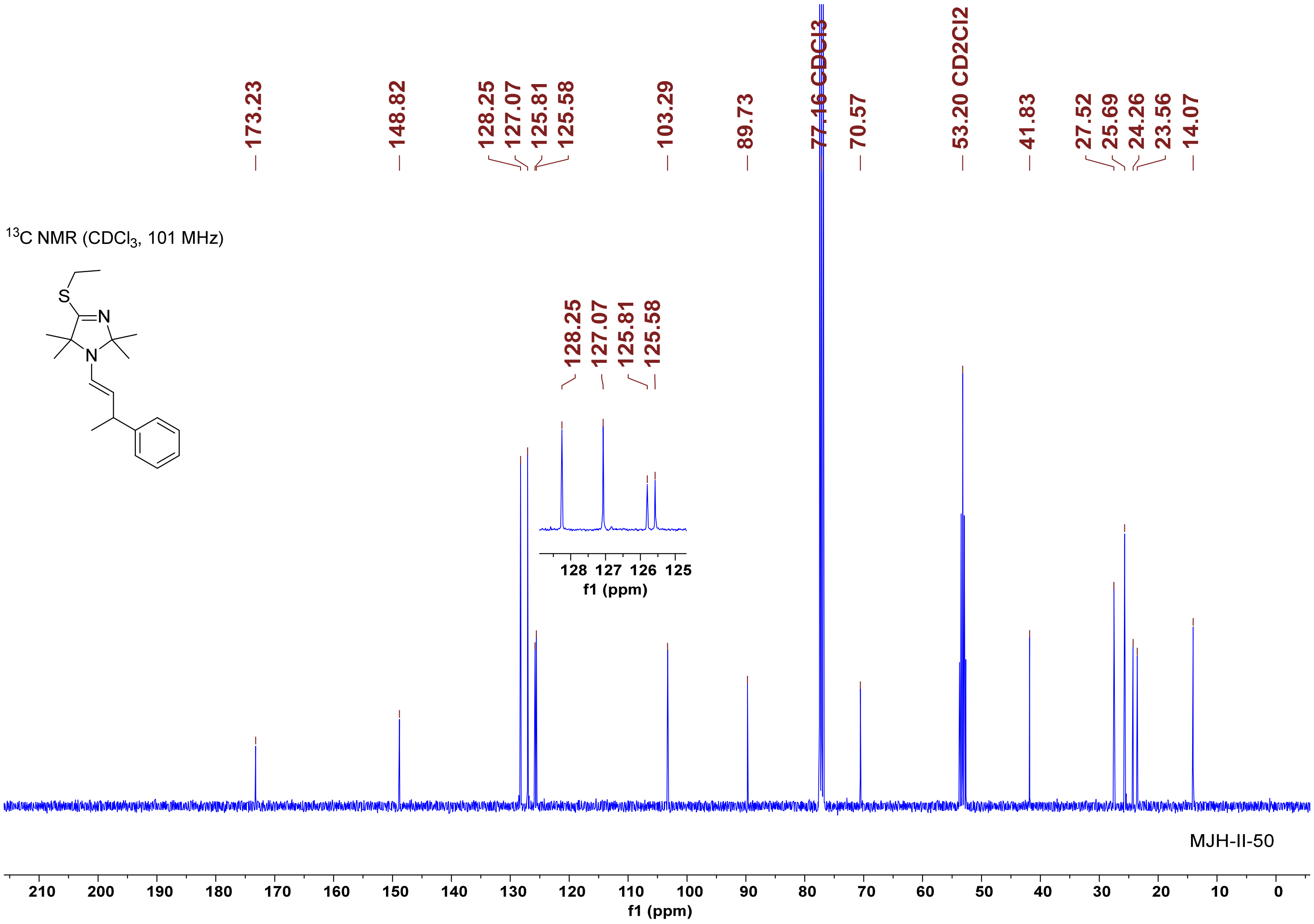

(*E*)-4-(Ethylthio)-2,2,5,5-tetramethyl-1-(3-phenylbut-1-en-1-yl)-2,5-dihydro-1H-imidazole (**4g**) (MJH-II-50)

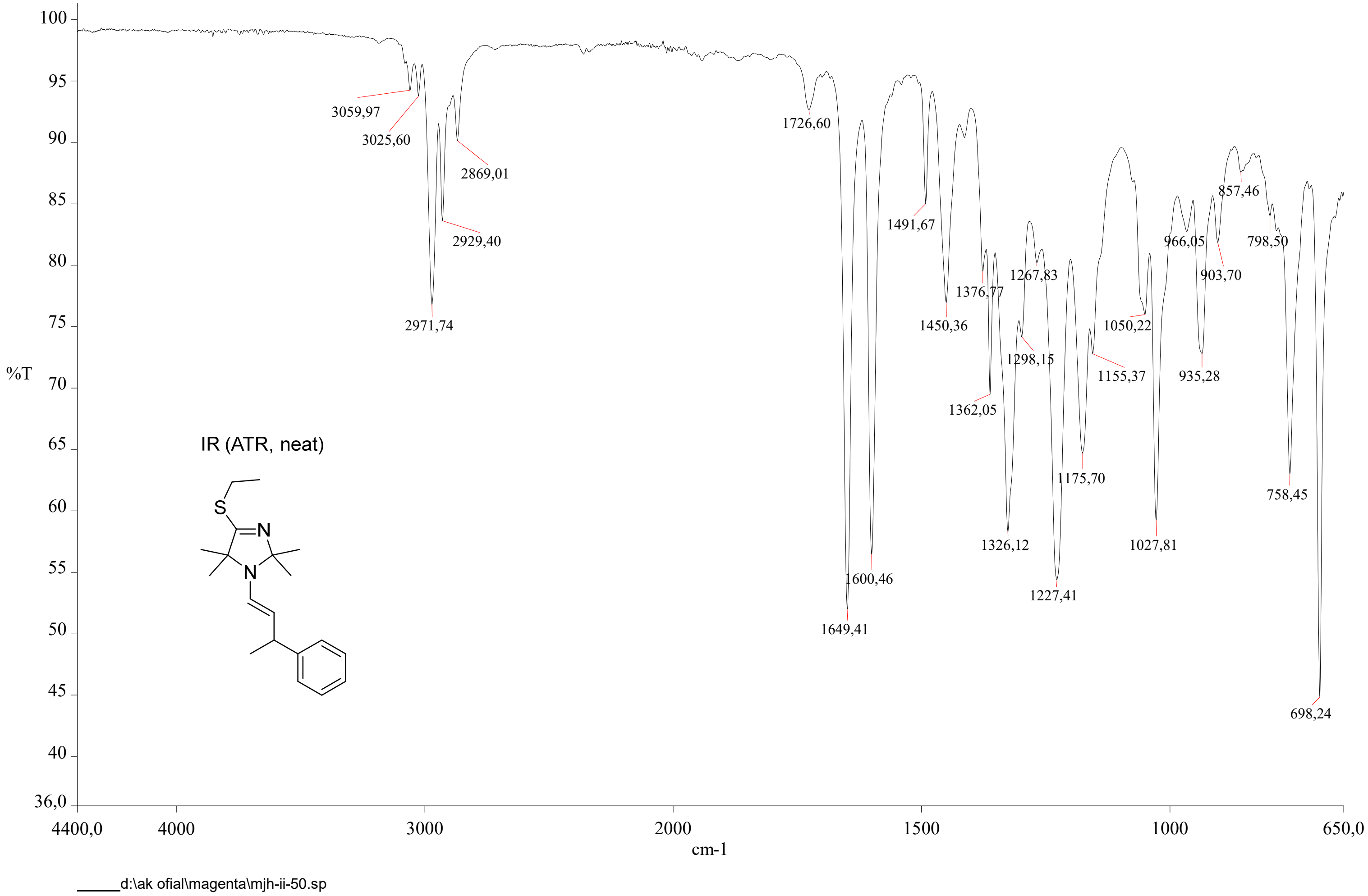

(*E*)-4-(Benzylthio)-2,2,5,5-tetramethyl-1-styryl-2,5-dihydro-1*H*-imidazole (4h) (MJH-I-78)

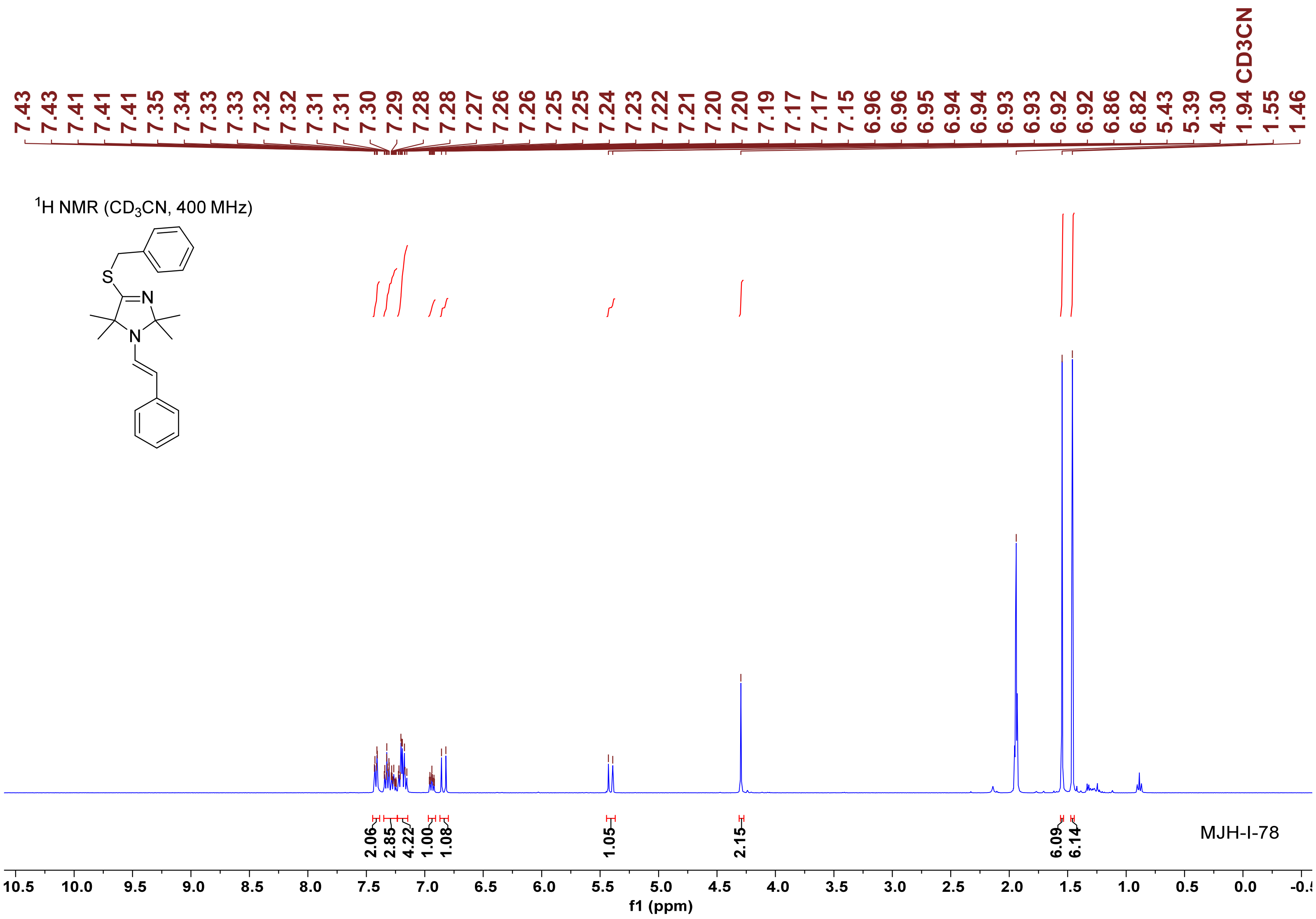

(*E*)-4-(Benzylthio)-2,2,5,5-tetramethyl-1-styryl-2,5-dihydro-1*H*-imidazole (4h) (MJH-I-78)

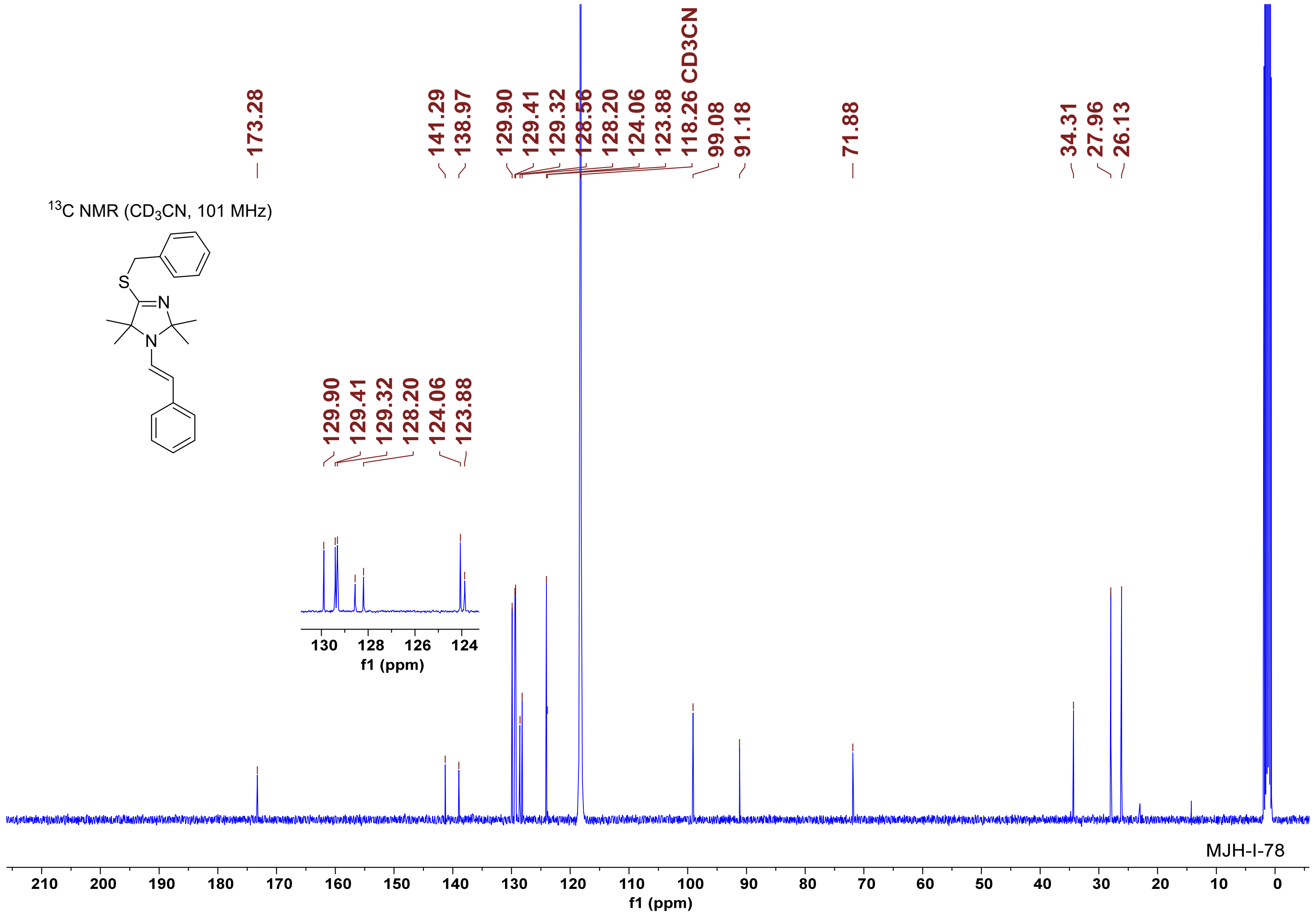

(*E*)-4-(Benzylthio)-2,2,5,5-tetramethyl-1-styryl-2,5-dihydro-1*H*-imidazole (4h) (MJH-I-78)

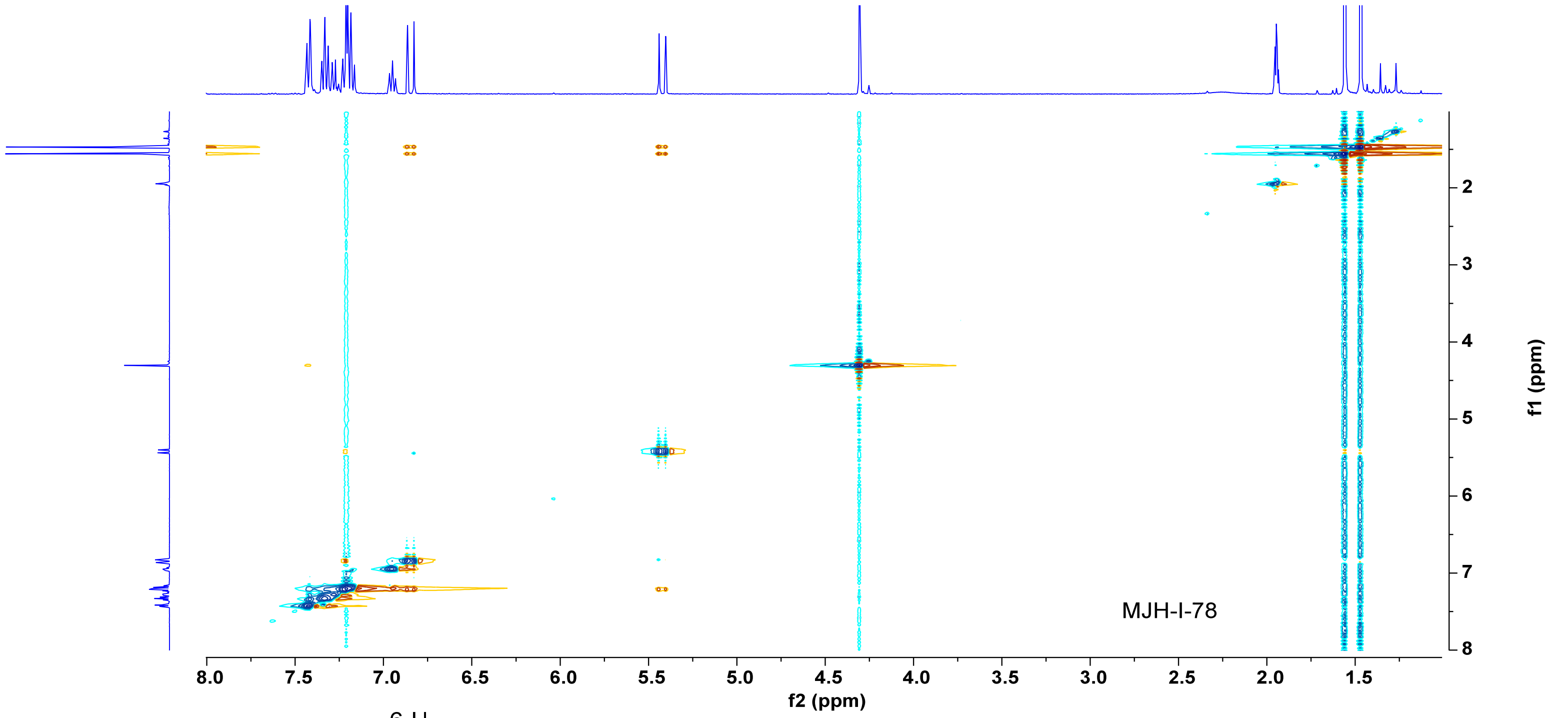

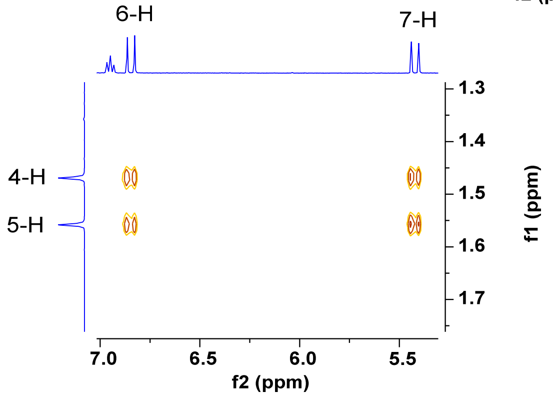

NOESY (CD_3CN, 400 MHz)

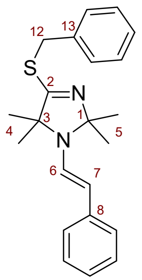

(*E*)-4-(Benzylthio)-2,2,5,5-tetramethyl-1-styryl-2,5-dihydro-1*H*-imidazole (4h) (MJH-I-78)

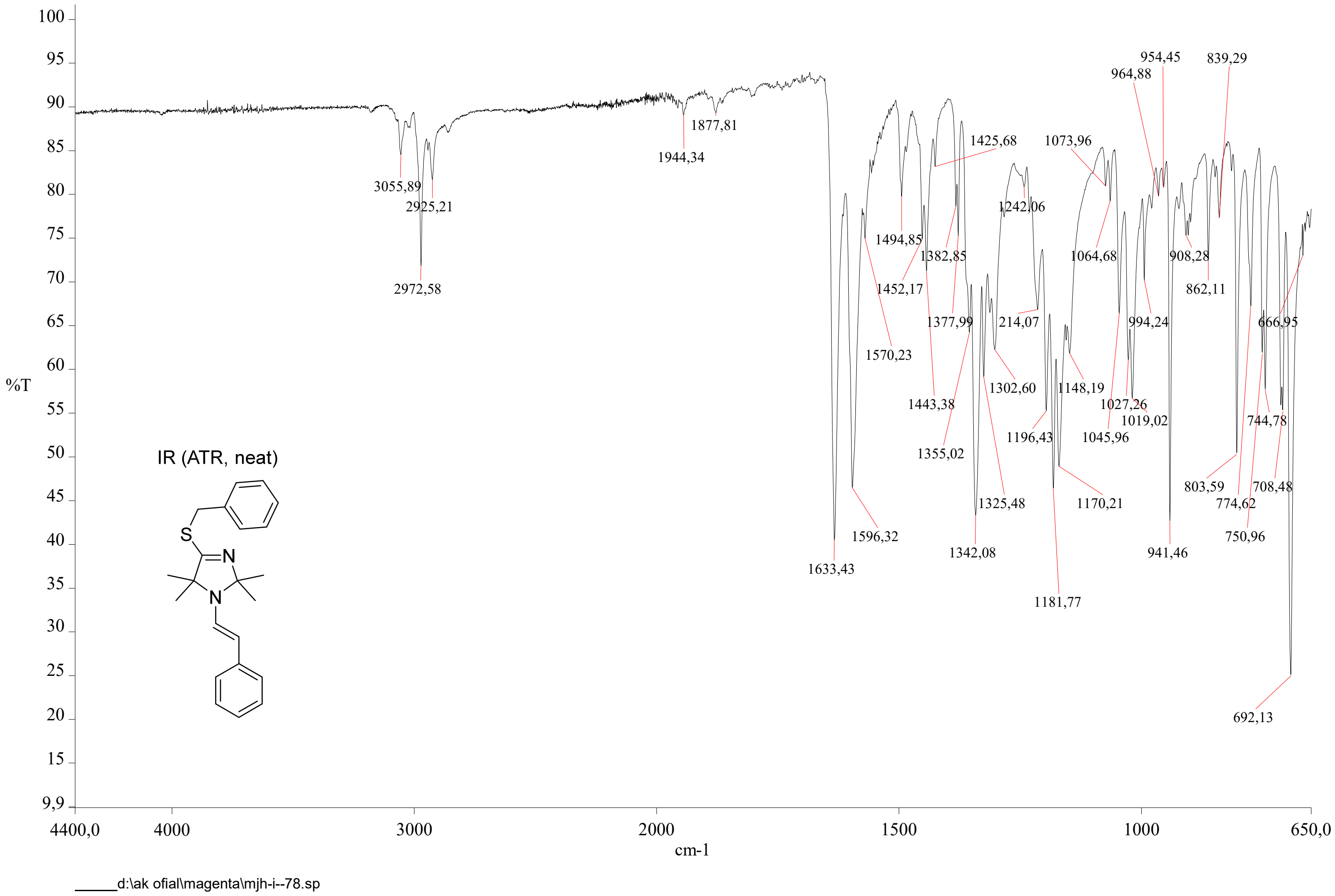

(*E*)-2,2,3,5,5-Pentamethyl-1-styrylimidazolidin-4-one (4i) (MJH-II-44)

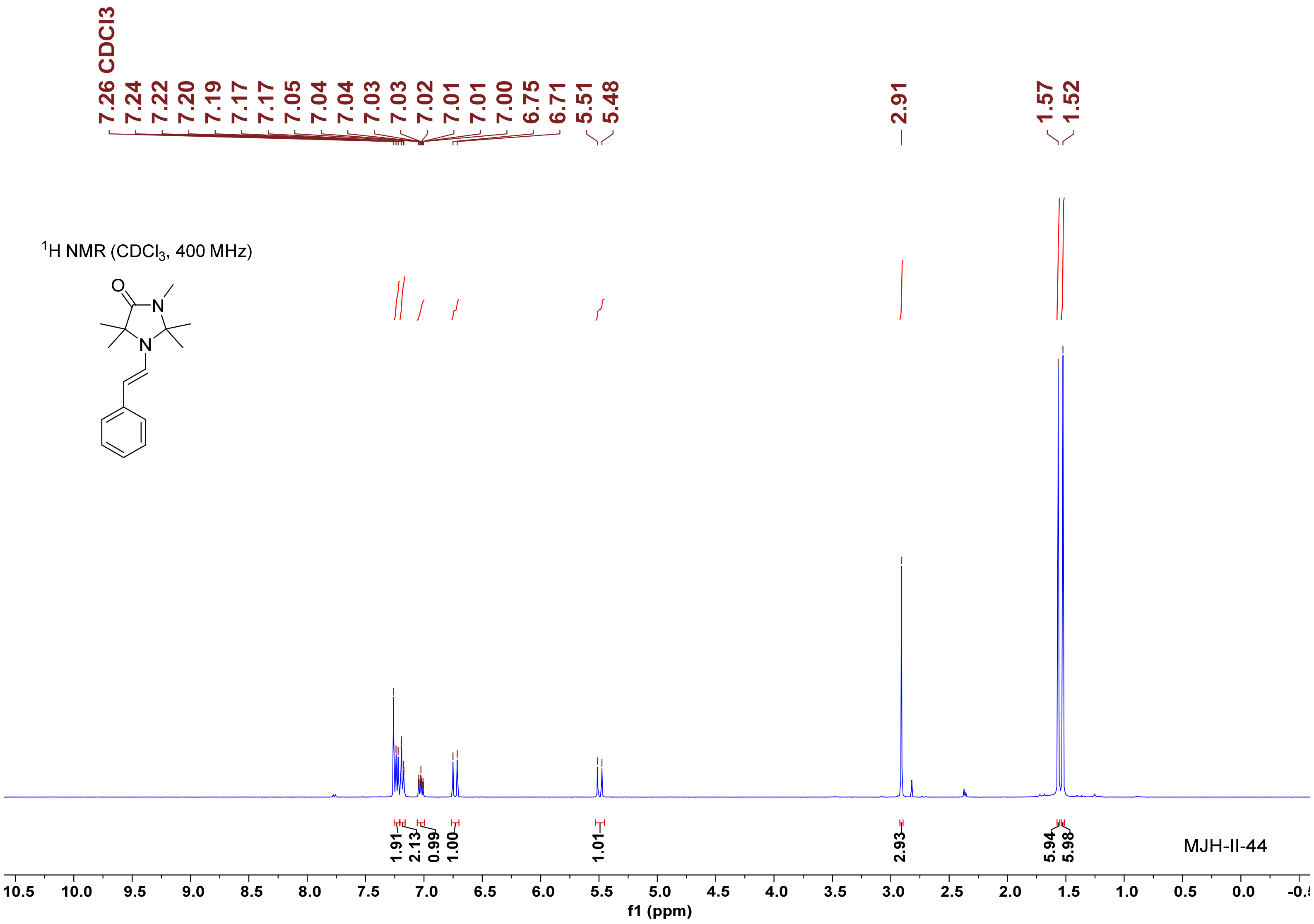

(*E*)-2,2,3,5,5-Pentamethyl-1-styrylimidazolidin-4-one (4i) (MJH-II-44)

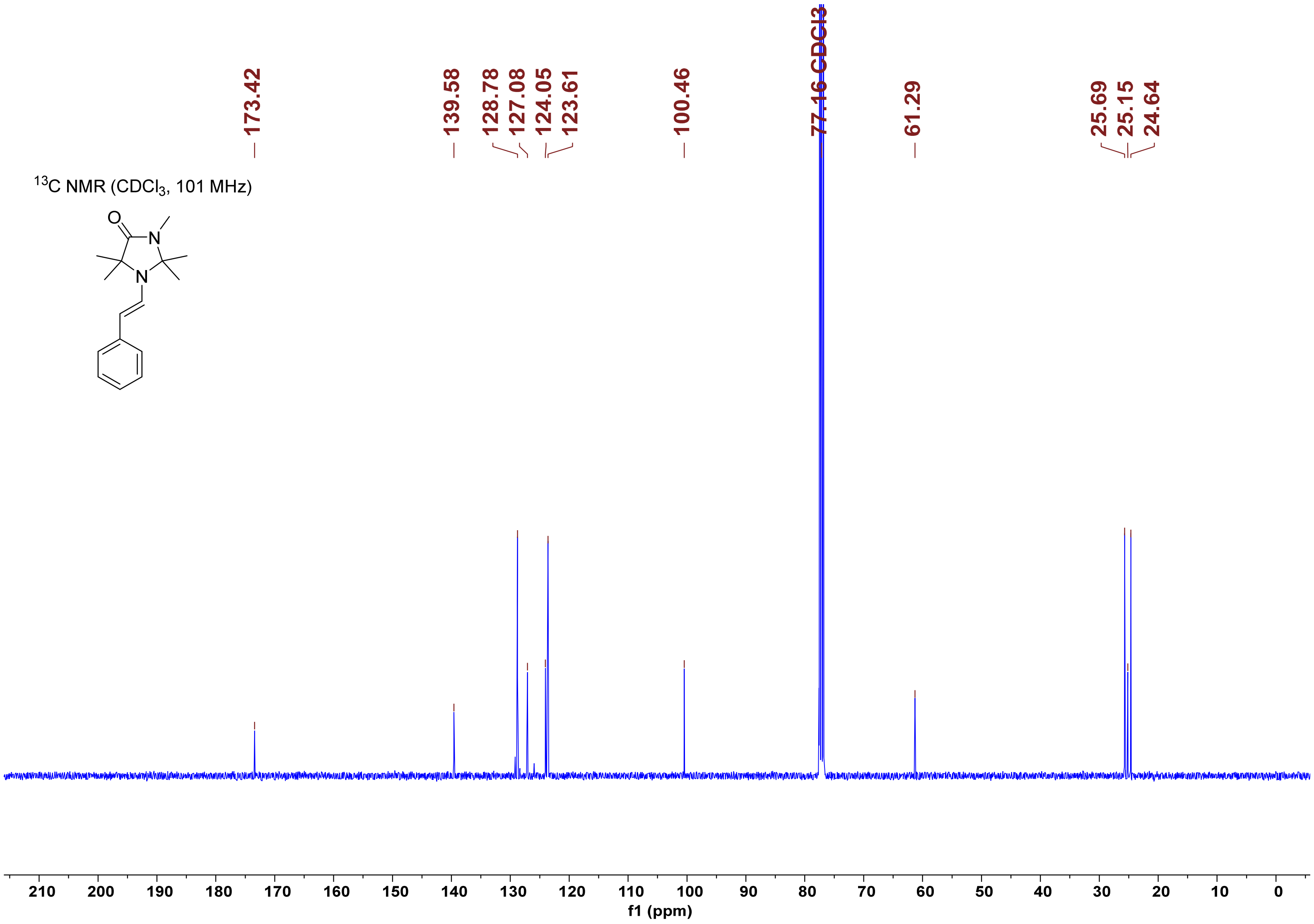

(*E*)-2,2,3,5,5-Pentamethyl-1-styrylimidazolidin-4-one (4i) (MJH-II-44)

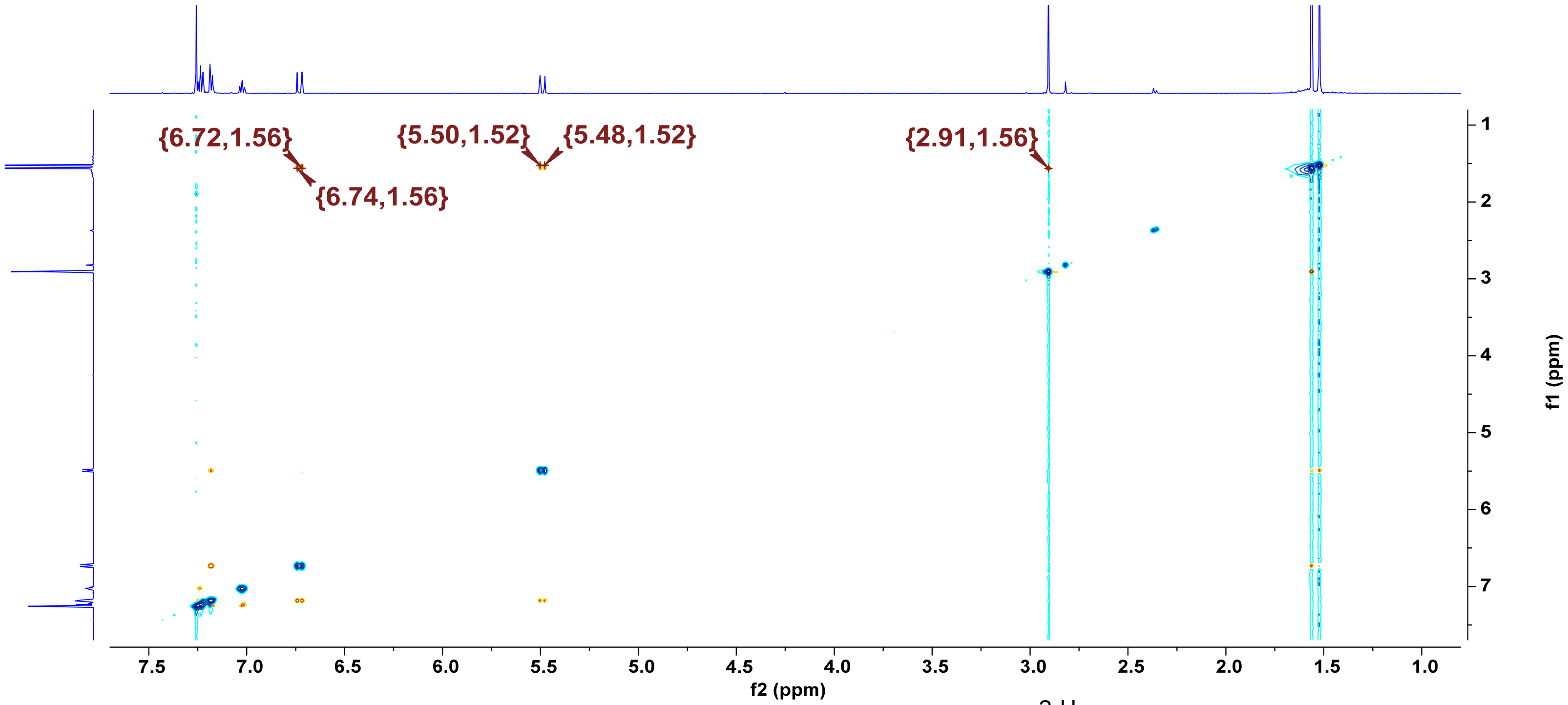

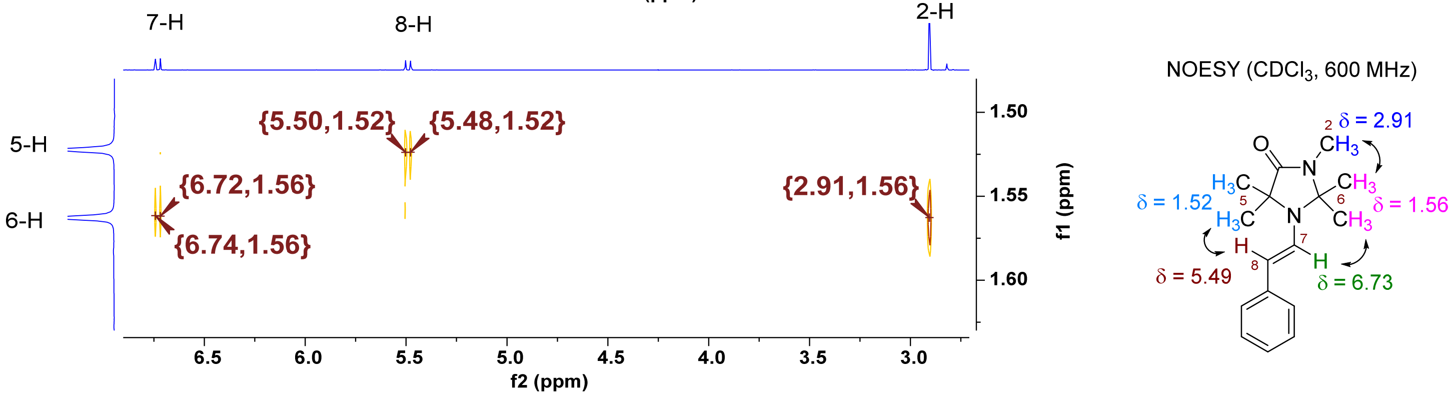

(*E*)-2,2,3,5,5-Pentamethyl-1-styrylimidazolidin-4-one (4i) (MJH-II-44)

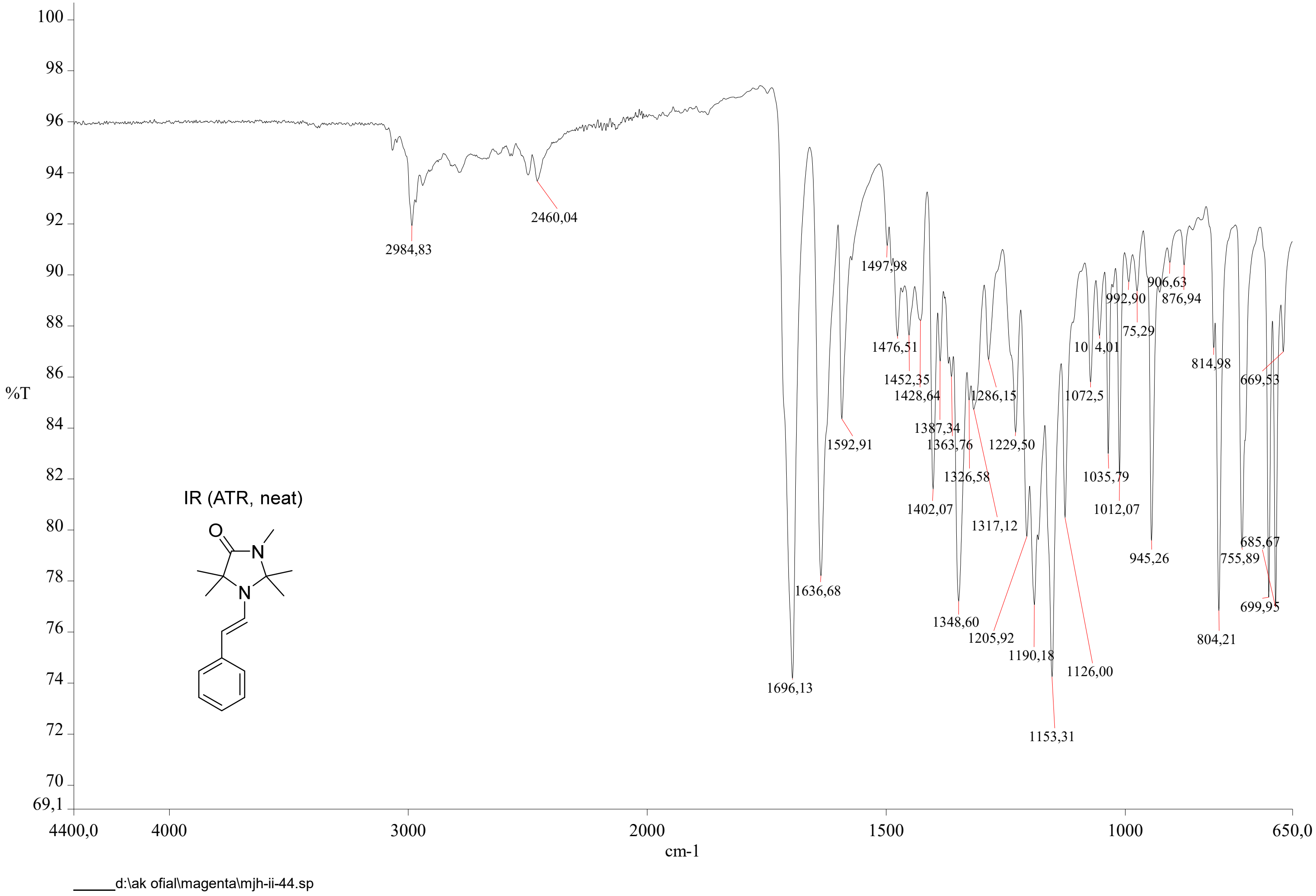

(*E*)-2,2,3,5,5-Pentamethyl-1-styrylimidazolidine-4-thione (**4j**) (MJH-II-8//MJH-I-172)

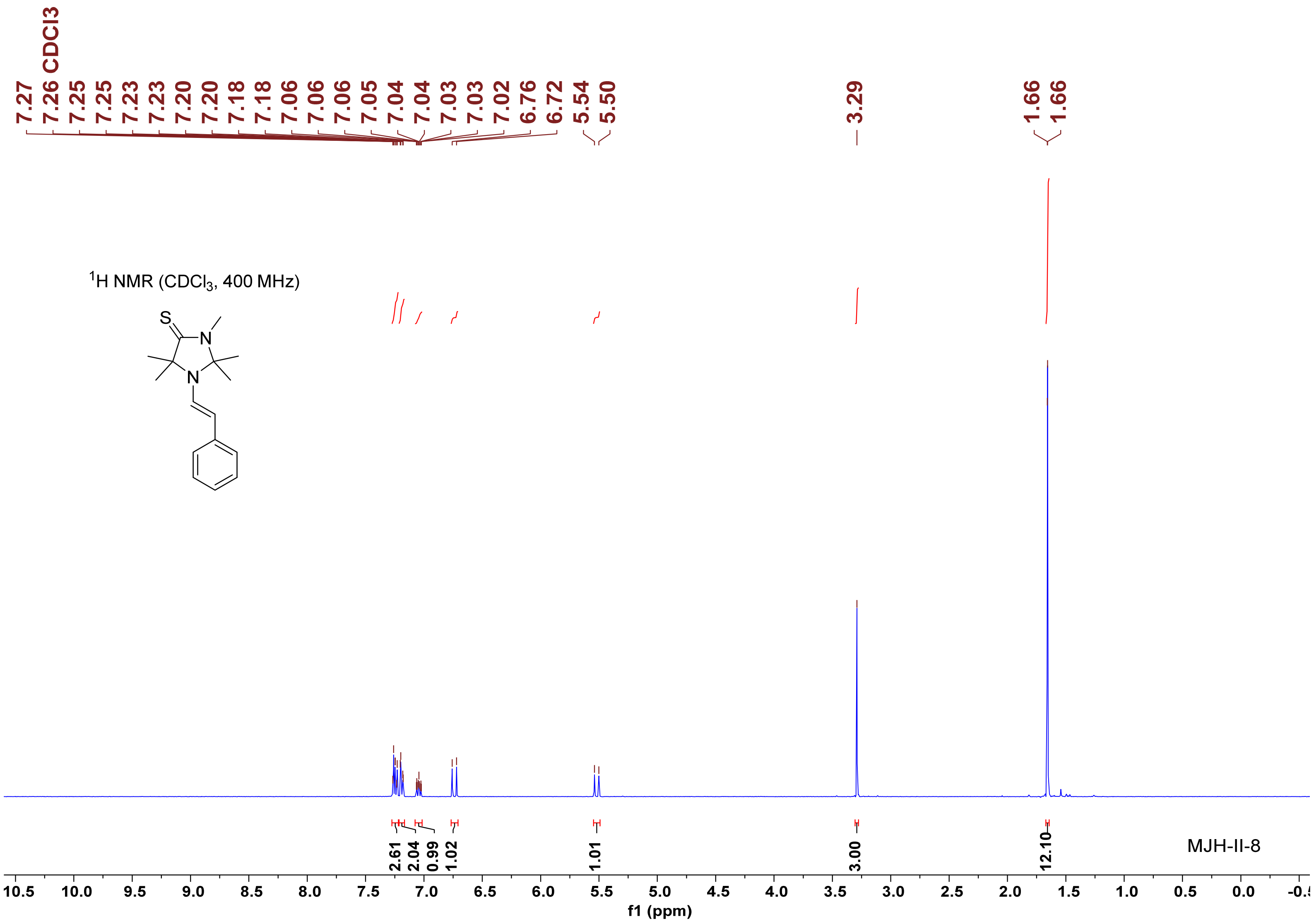

(*E*)-2,2,3,5,5-Pentamethyl-1-styrylimidazolidine-4-thione (4j) (MJH-II-8//MJH-I-172)

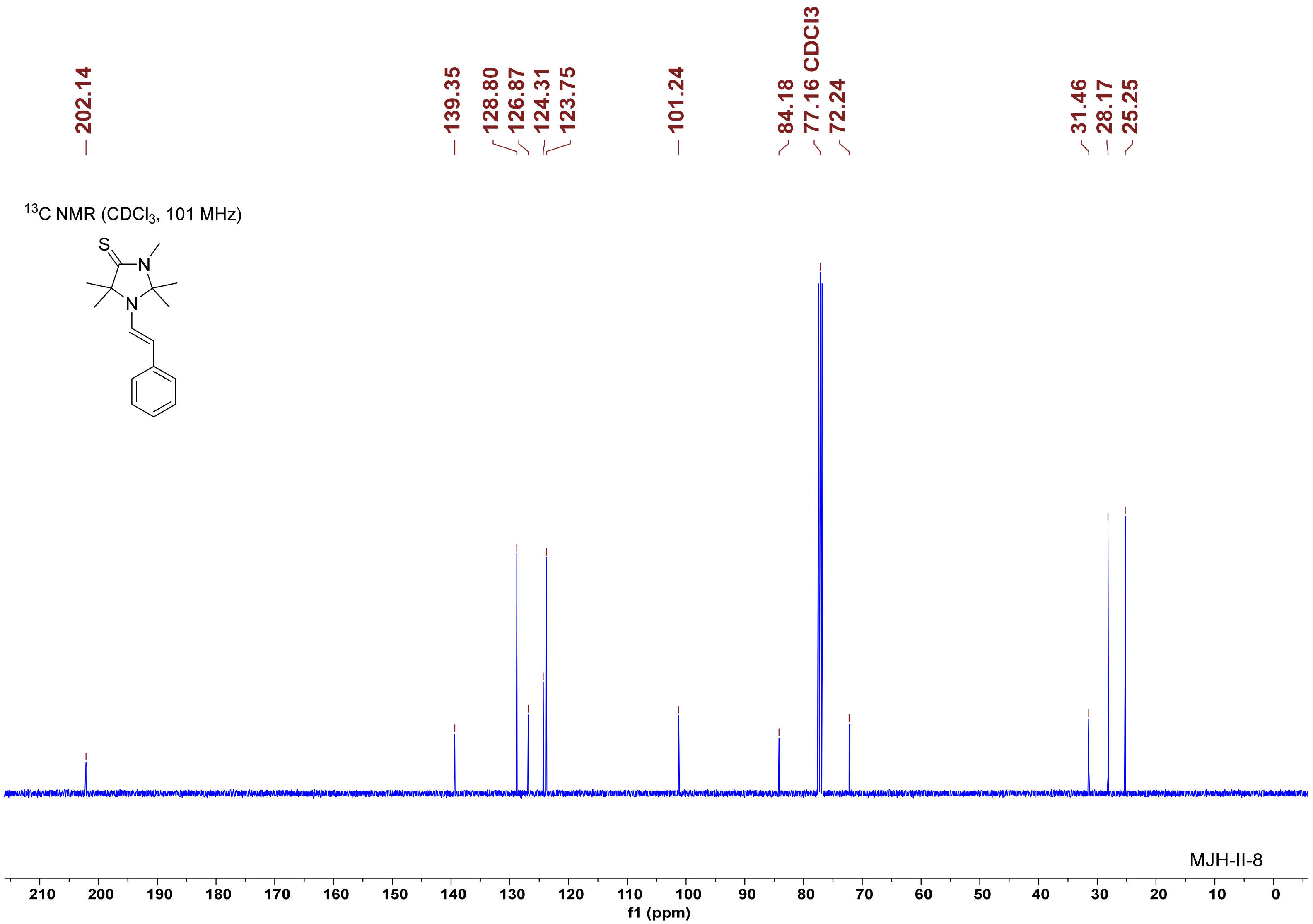

(*E*)-2,2,3,5,5-Pentamethyl-1-styrylimidazolidine-4-thione (4j) (MJH-II-8//MJH-I-172)

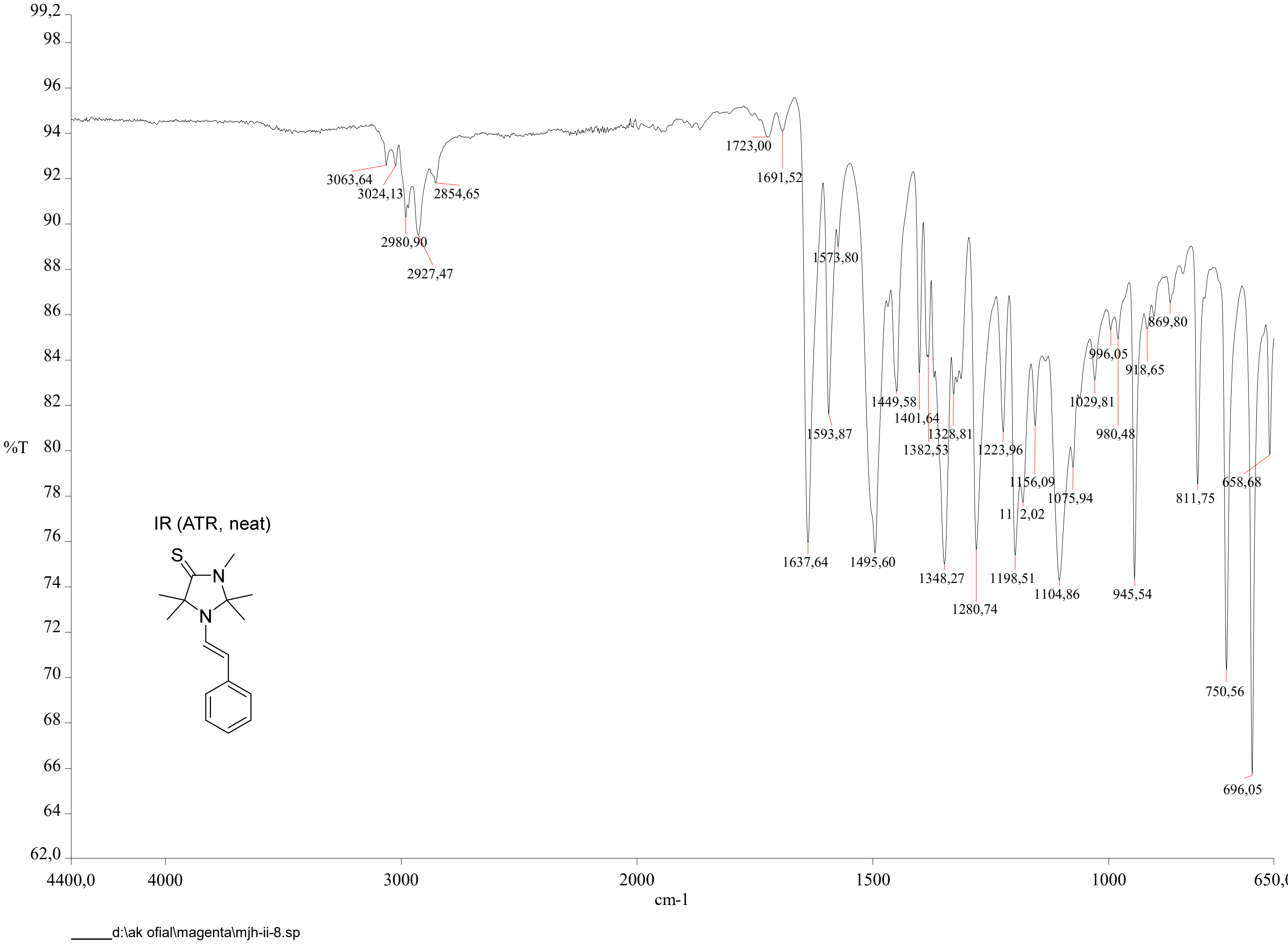

2-Phenyl-3,3-bis(4-(phenyl(2,2,2-trifluoroethyl)amino)phenyl)propanal (**7a**) (MJH-II-56)

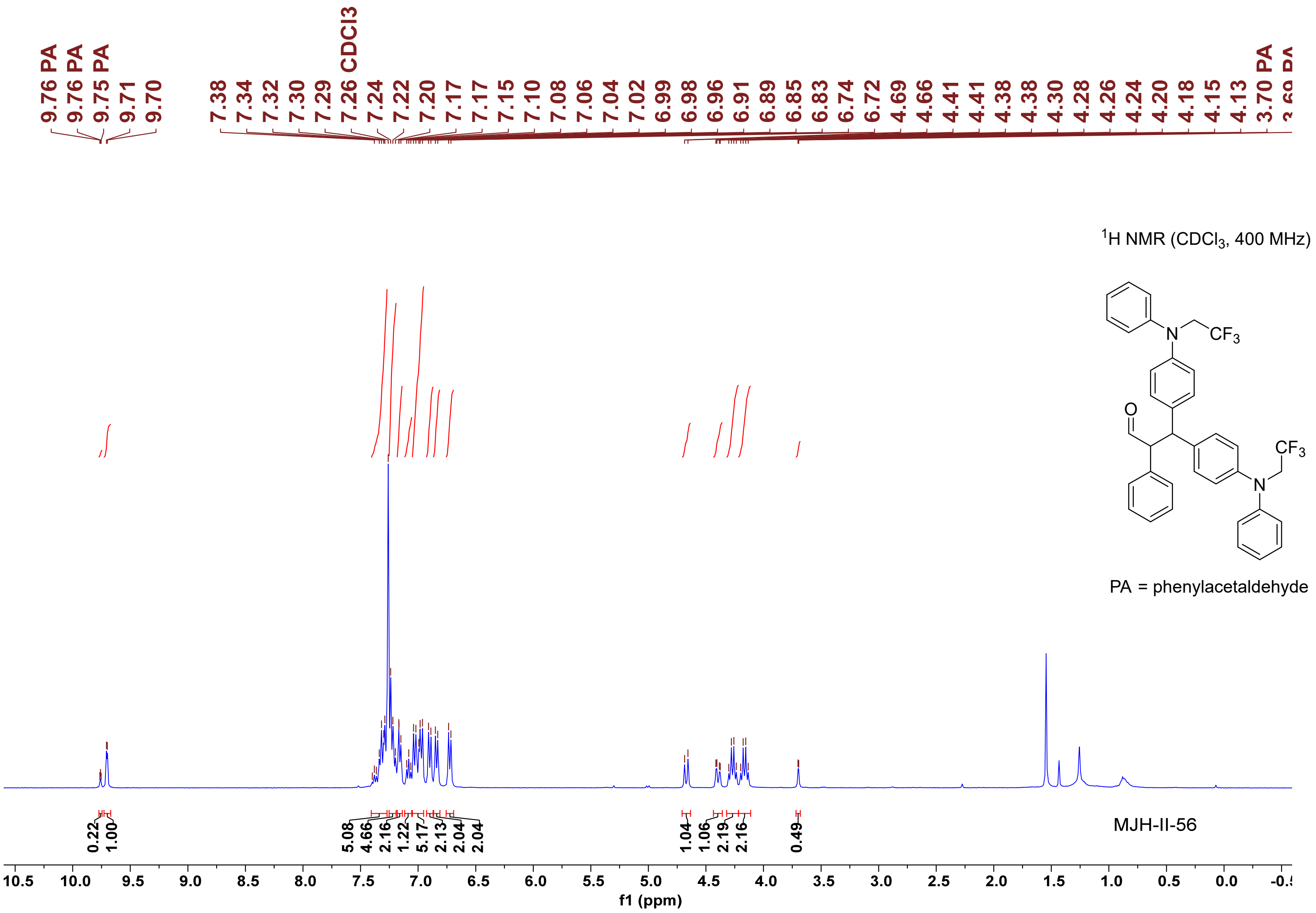

2-Phenyl-3,3-bis(4-(phenyl(2,2,2-trifluoroethyl)amino)phenyl)propanal (**7a**) (MJH-II-56)

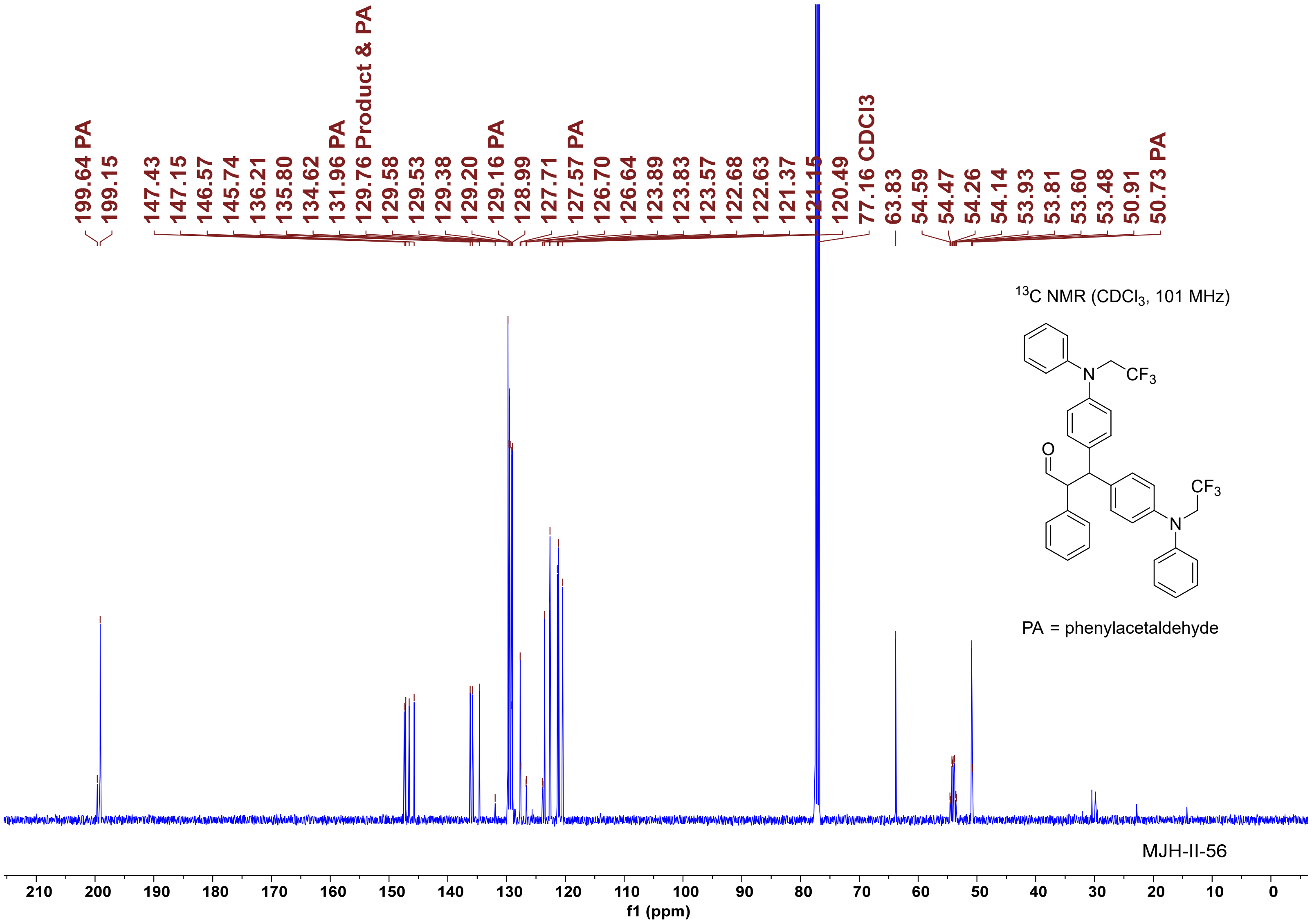

2-Phenyl-3,3-bis(4-(phenyl(2,2,2-trifluoroethyl)amino)phenyl)propanal (**7a**) (MJH-II-21)

^{19}F NMR ($CDCl_3$, 377 MHz)

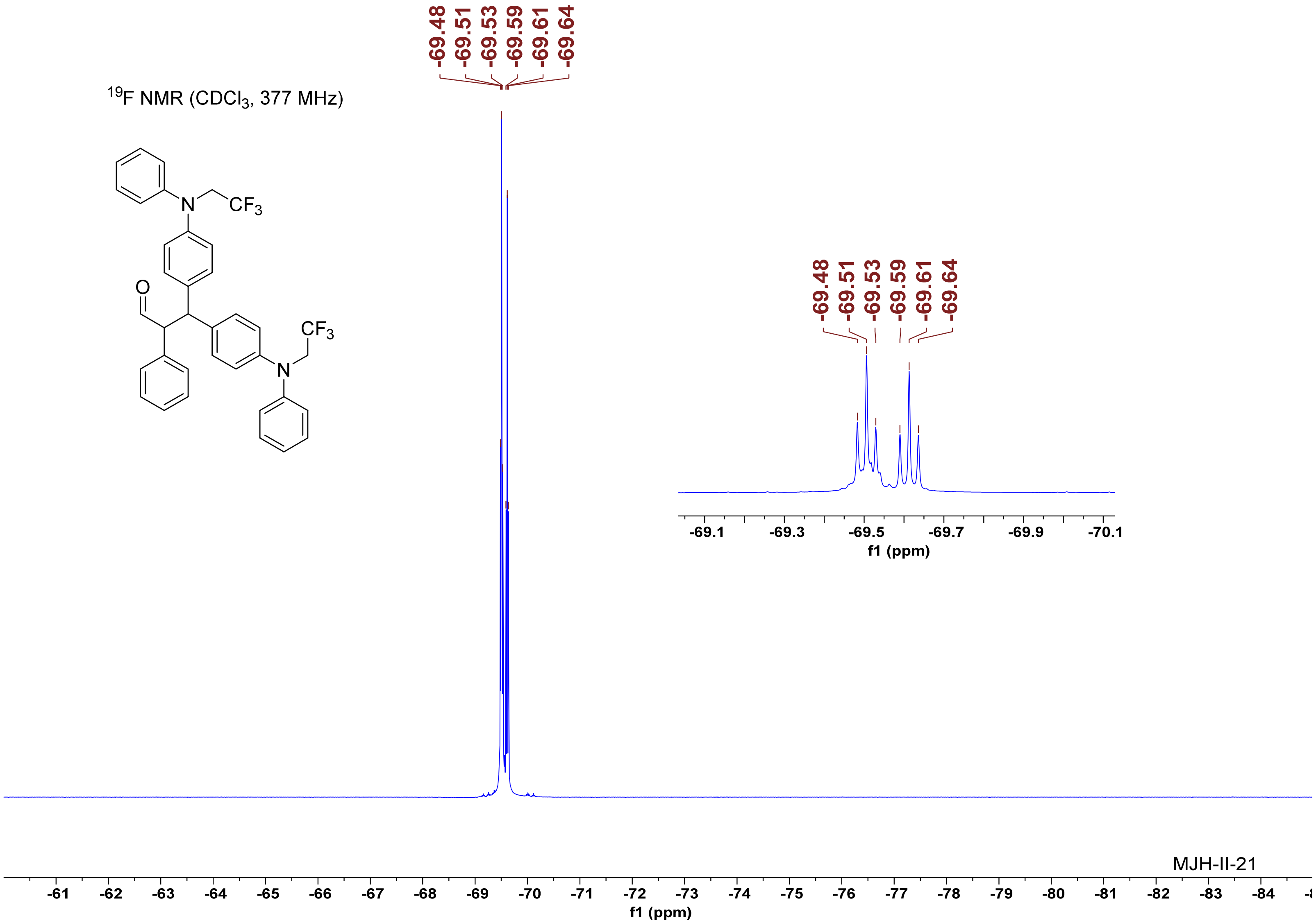

3,3-Bis(4-(methyl(2,2,2-trifluoroethyl)amino)phenyl)-2-phenylpropanal (**7b**) (MJH-I-43)

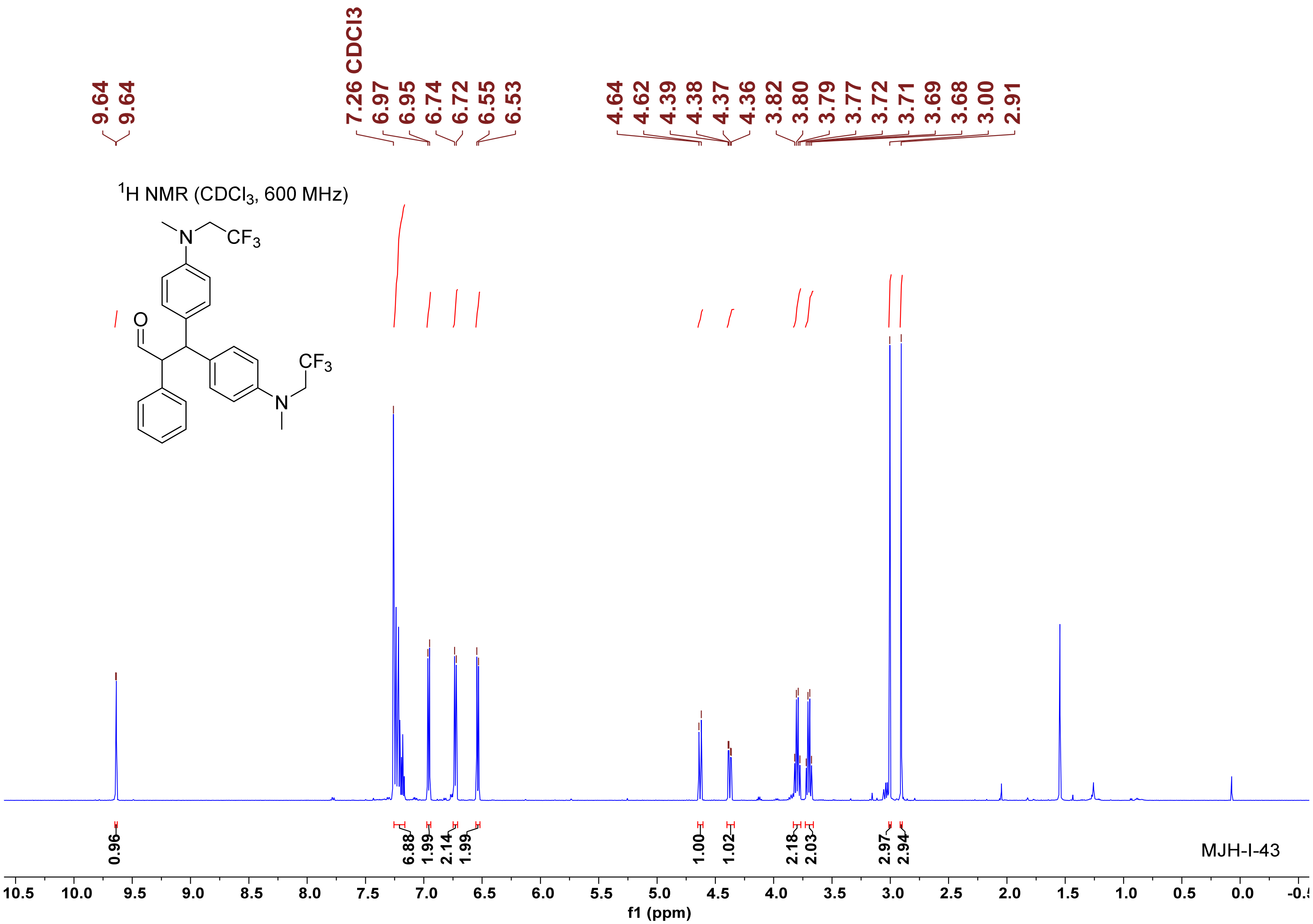

3,3-Bis(4-(methyl(2,2,2-trifluoroethyl)amino)phenyl)-2-phenylpropanal (**7b**) (MJH-I-43)

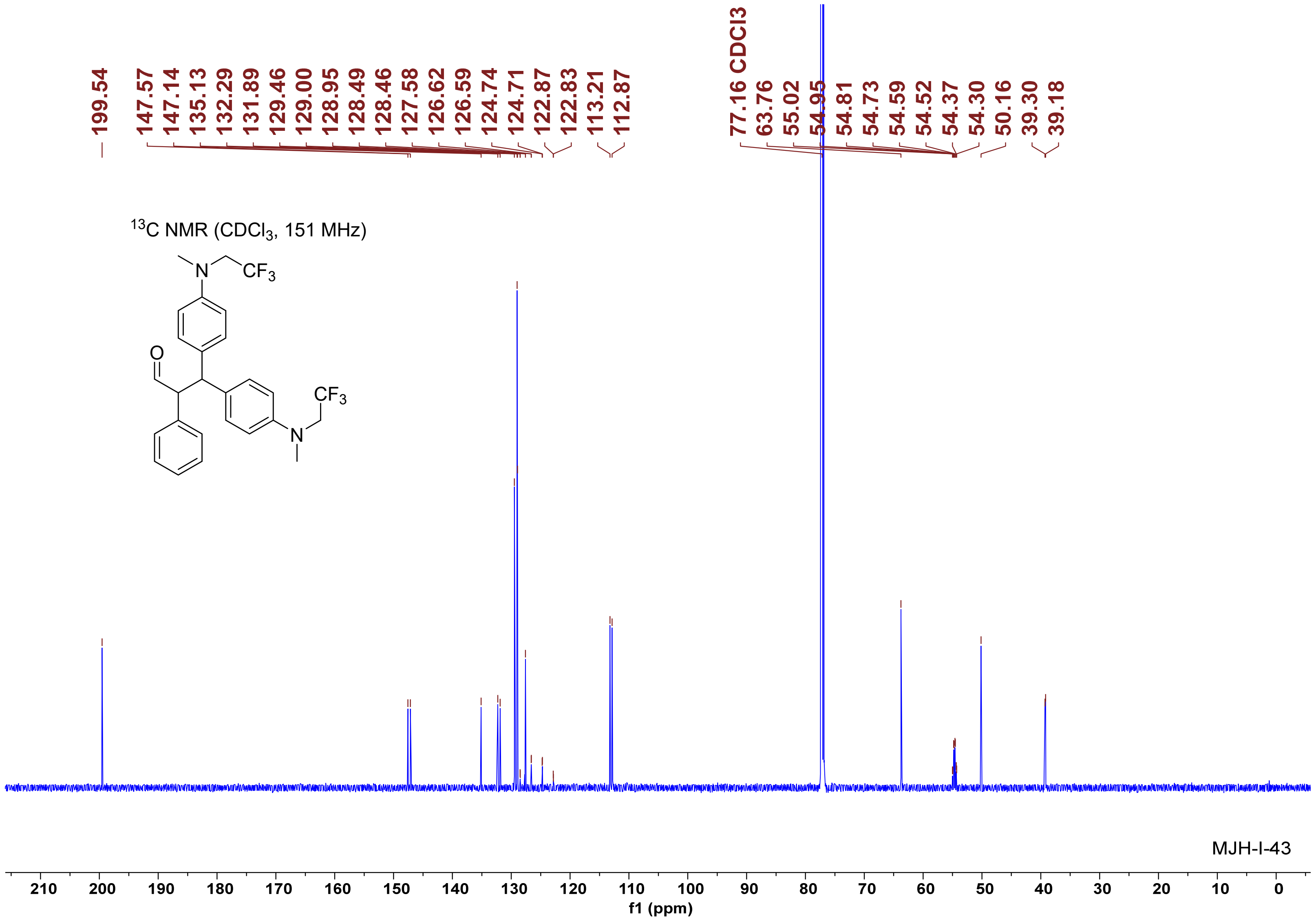

3,3-Bis(4-(methyl(2,2,2-trifluoroethyl)amino)phenyl)-2-phenylpropanal (**7b**) (MJH-I-43)

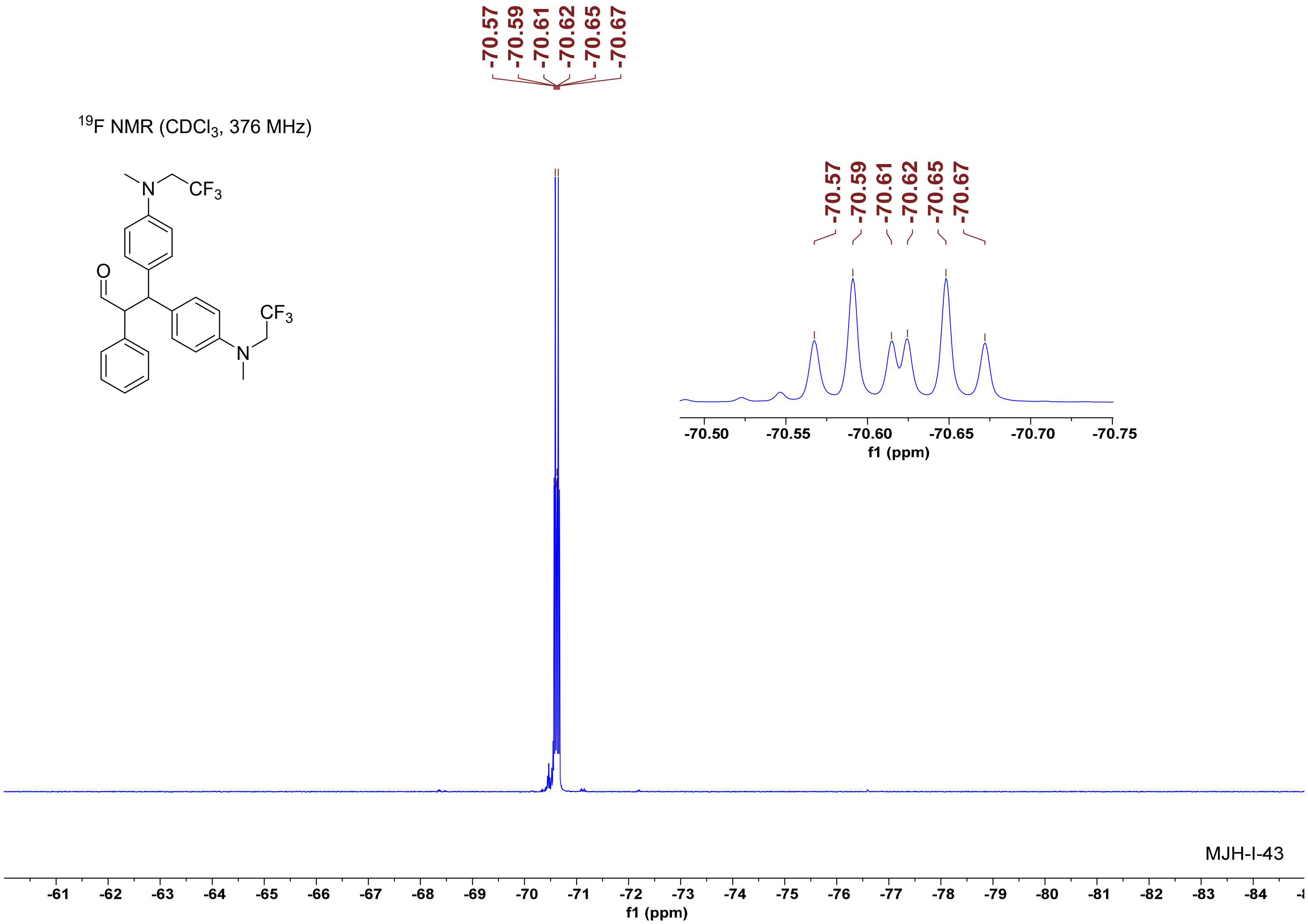

3,3-Bis(4-(methyl(2,2,2-trifluoroethyl)amino)phenyl)-2-phenylpropanal (**7b**) (MJH-I-43)

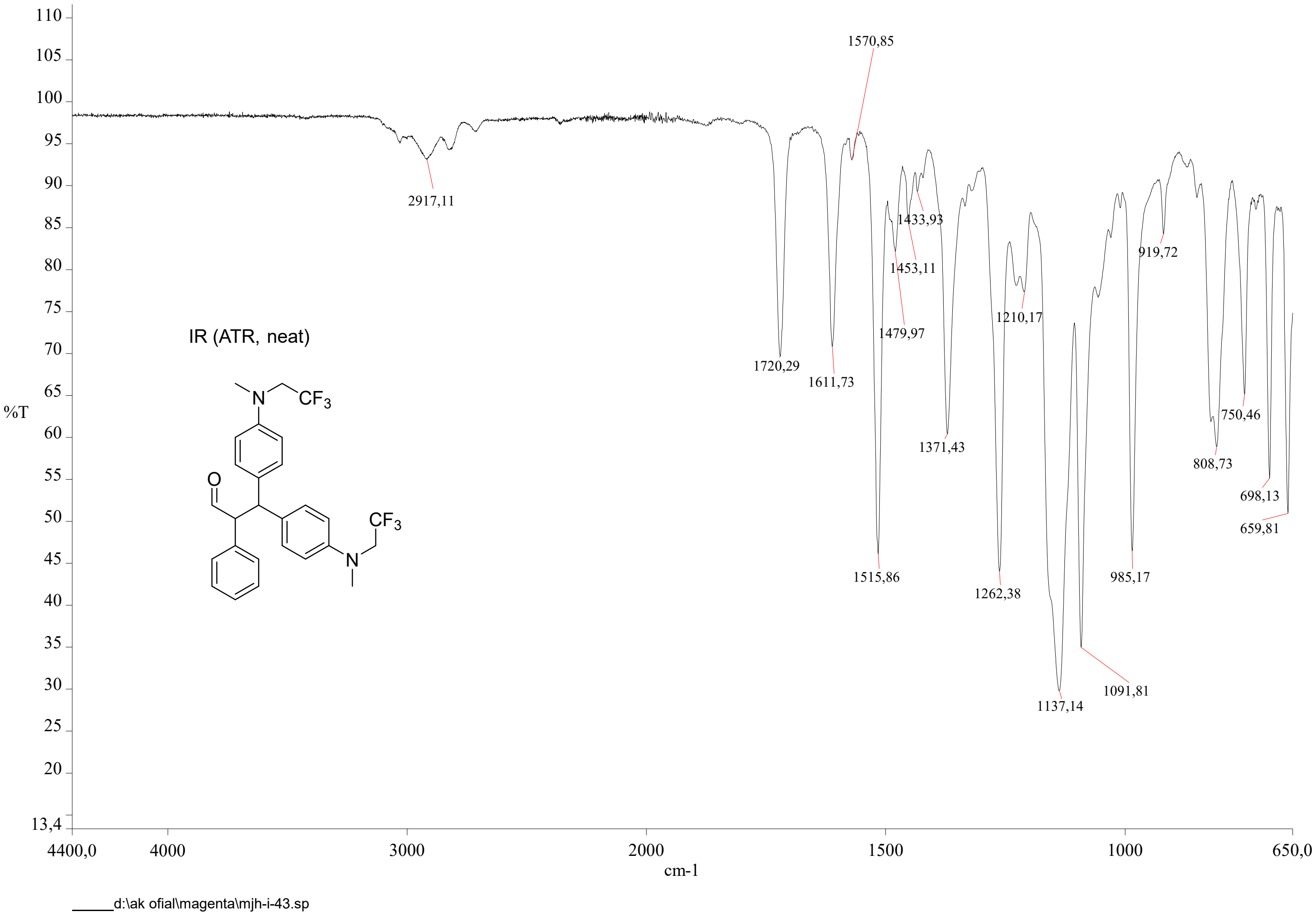

3,3-Bis(4-(methyl(2,2,2-trifluoroethyl)amino)phenyl)-2-phenylpropanal (7b) (MJH-I-189)

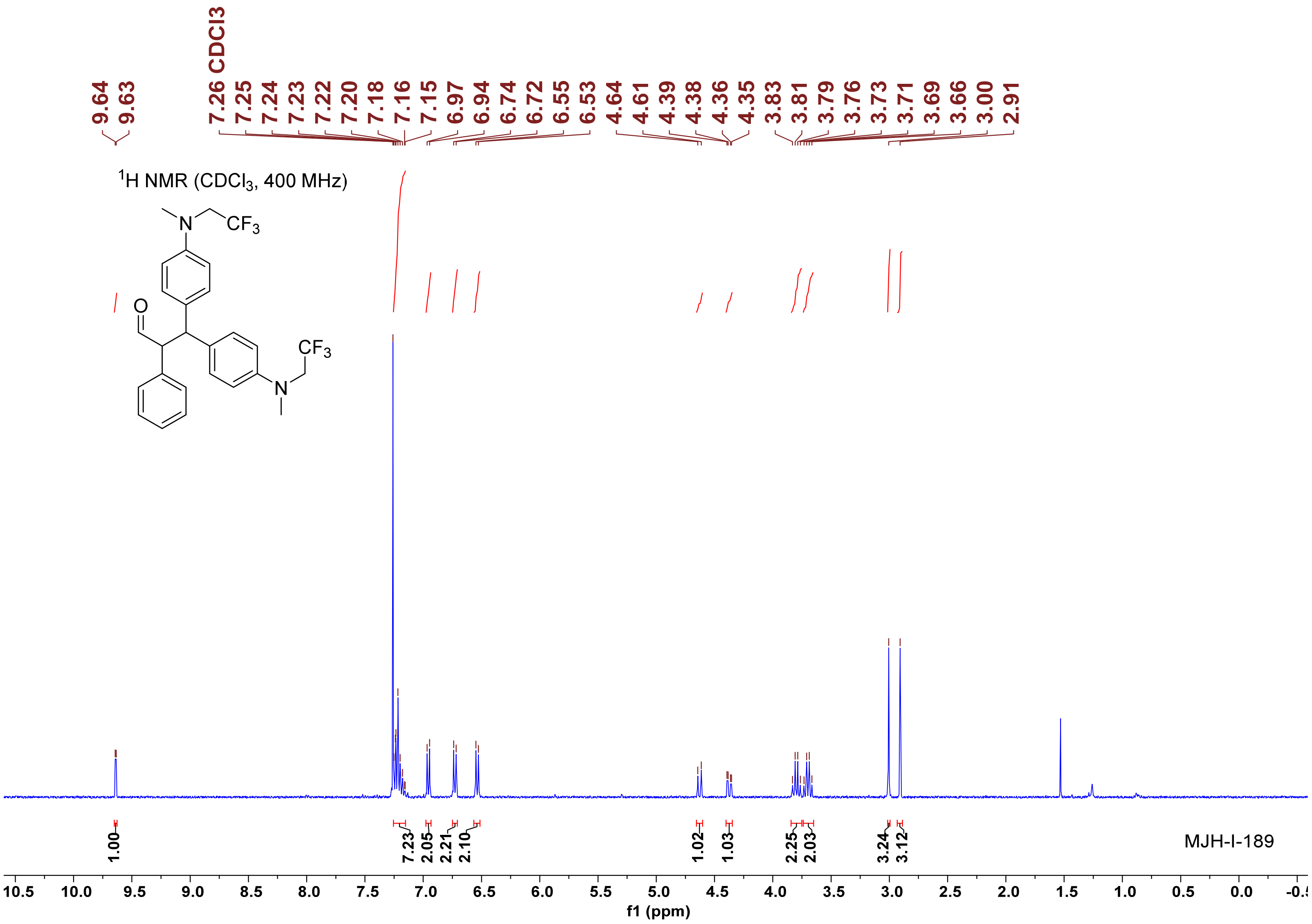

3,3-Bis(4-diphenylaminophenyl)-2-phenylpropanal (7c) (MJH-I-88)

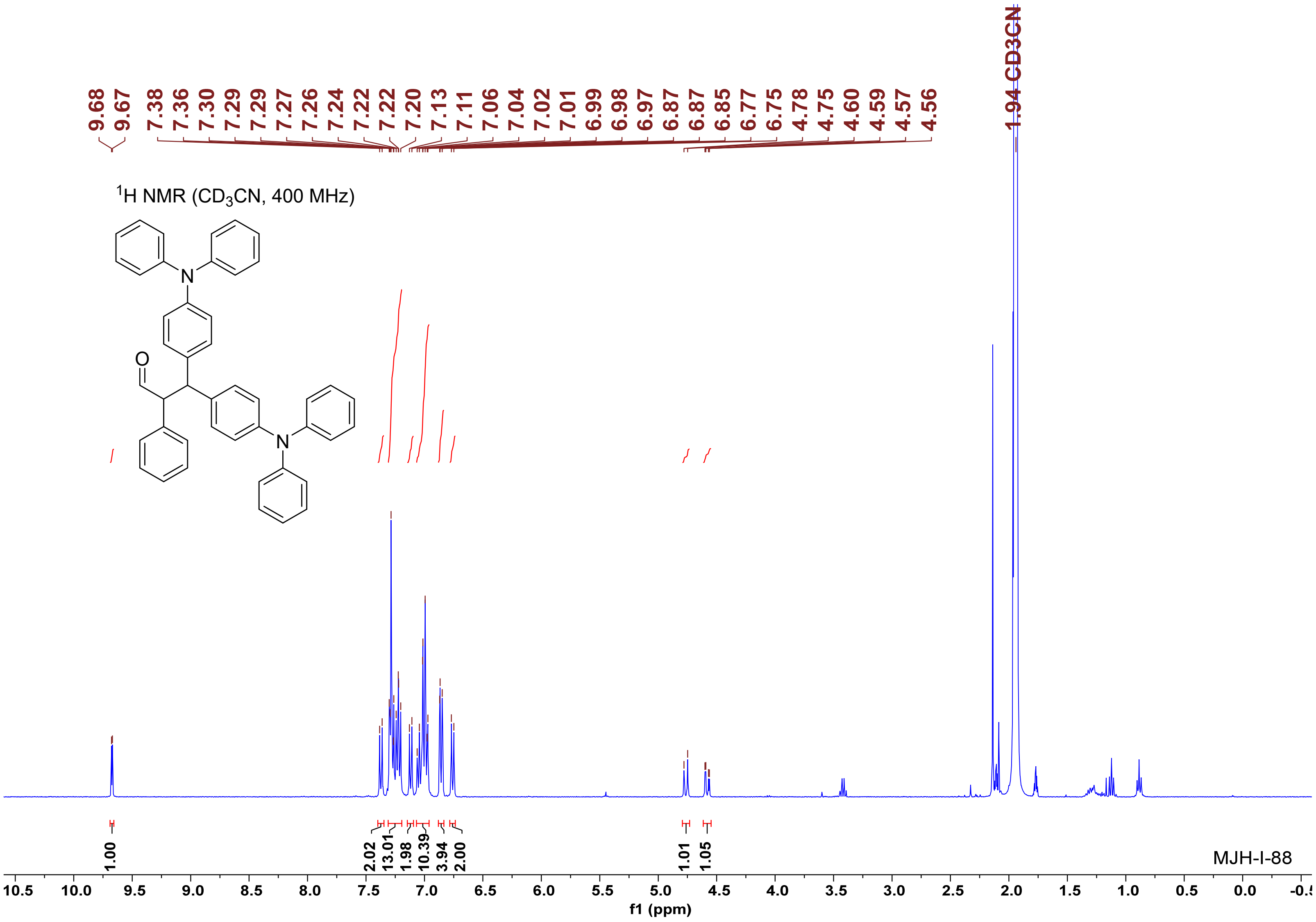

3,3-Bis(4-diphenylaminophenyl)-2-phenylpropanal (**7c**) (MJH-I-88)

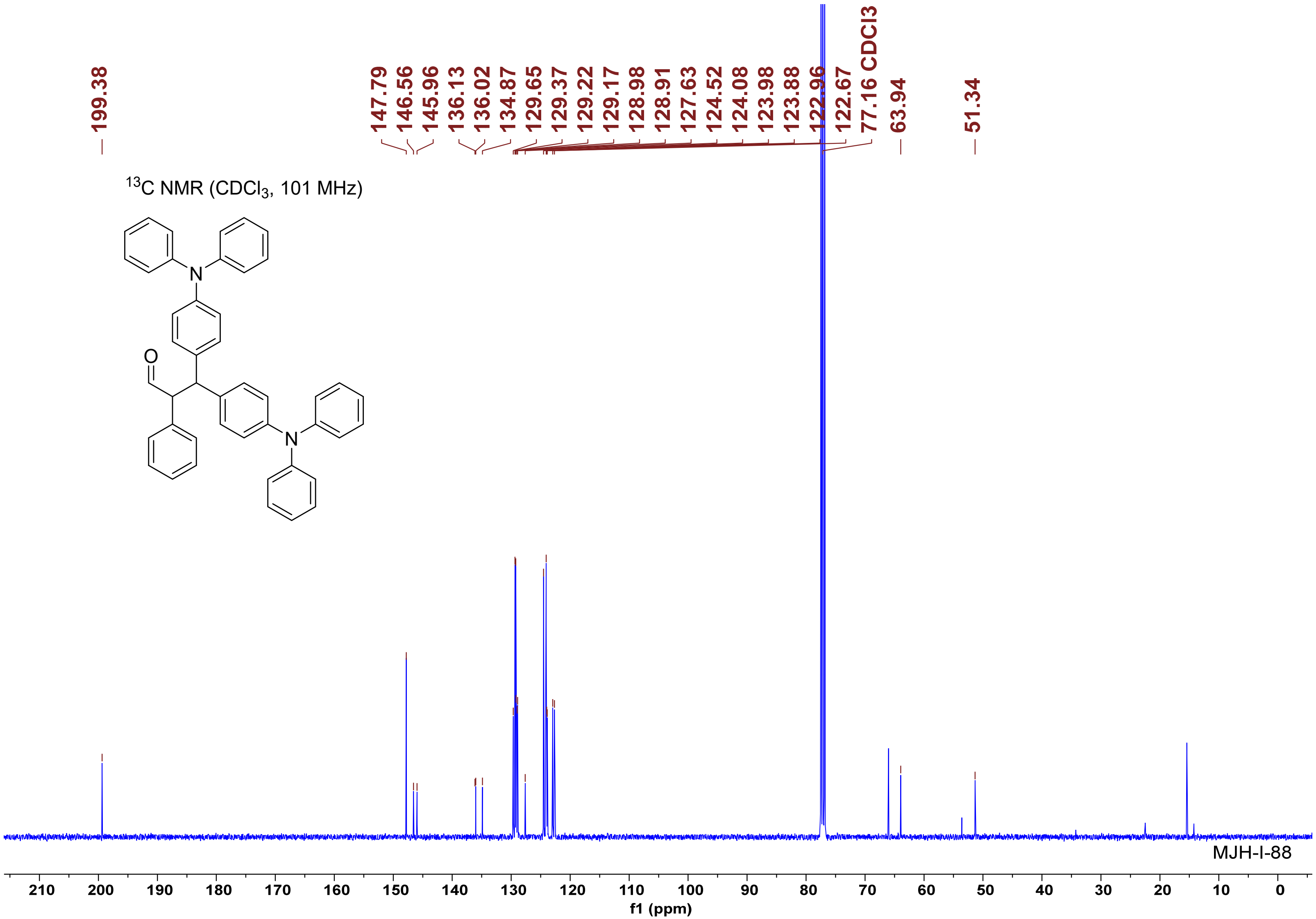

3,3-Bis(4-diphenylaminophenyl)-2-phenylpropanal (7c) (MJH-I-88)

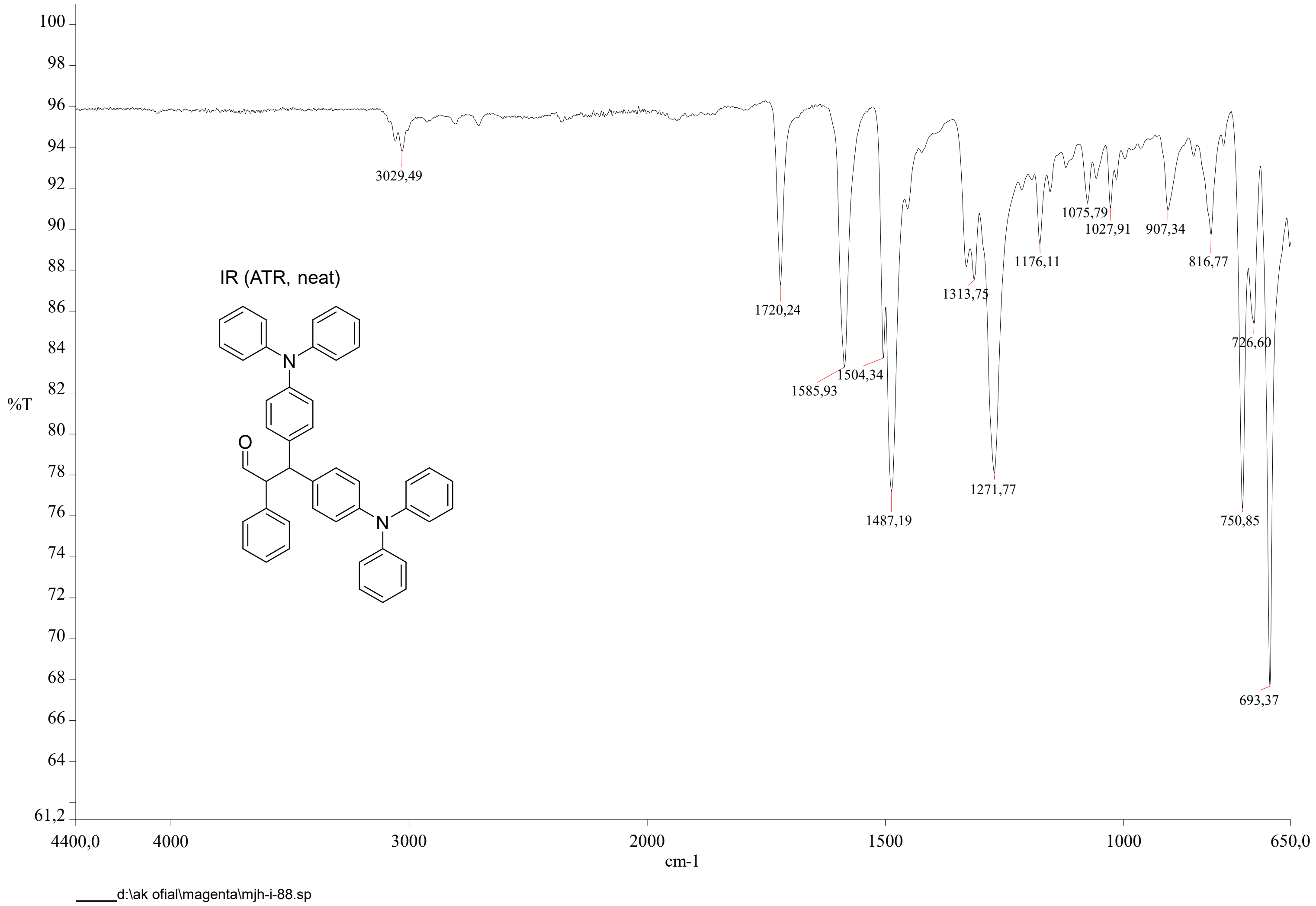

3,3-Bis(4-morpholinophenyl)-2-phenylpropanal (7d) (MJH-I-181)

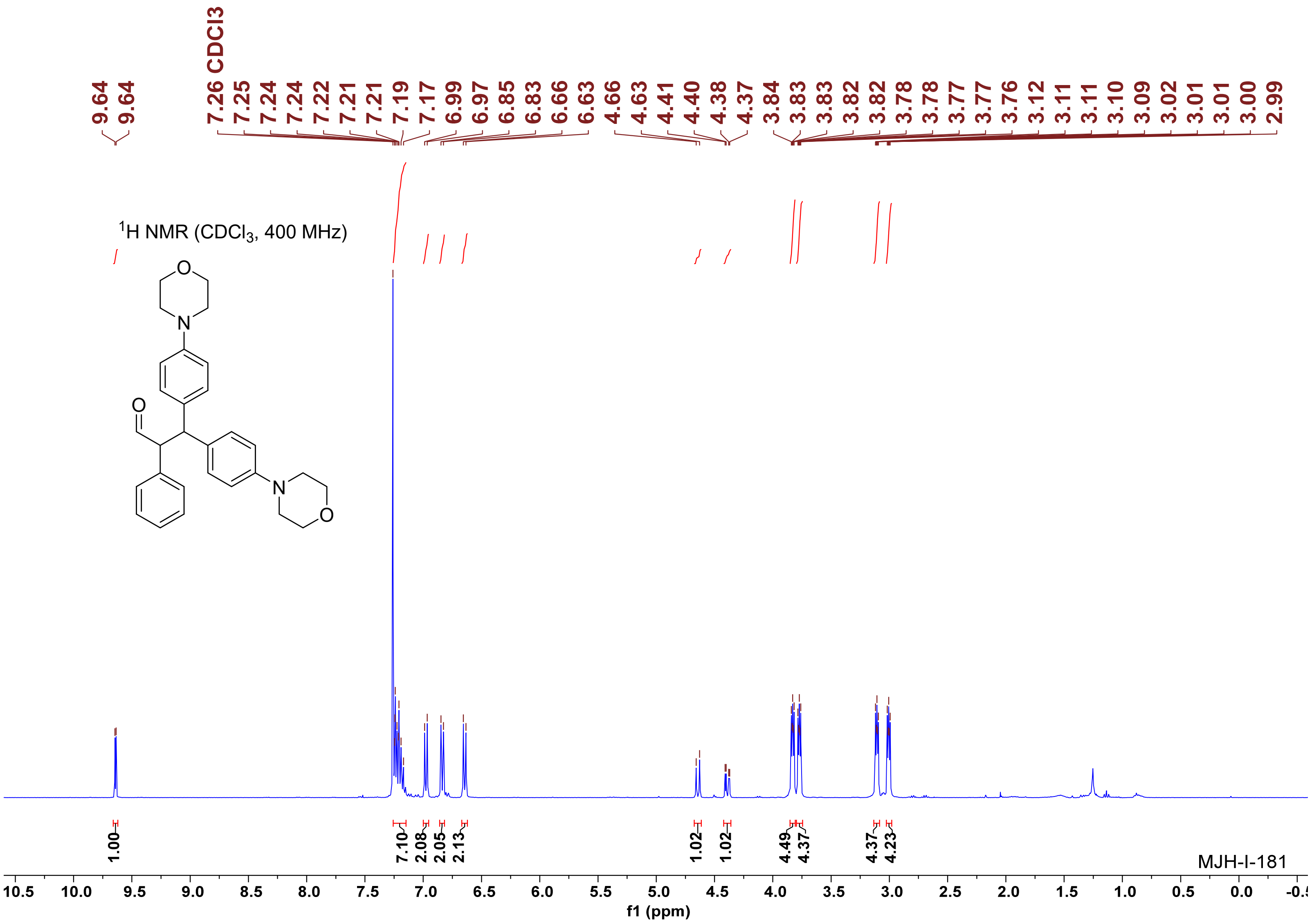

3,3-Bis(4-morpholinophenyl)-2-phenylpropanal (**7d**) (MJH-I-45)

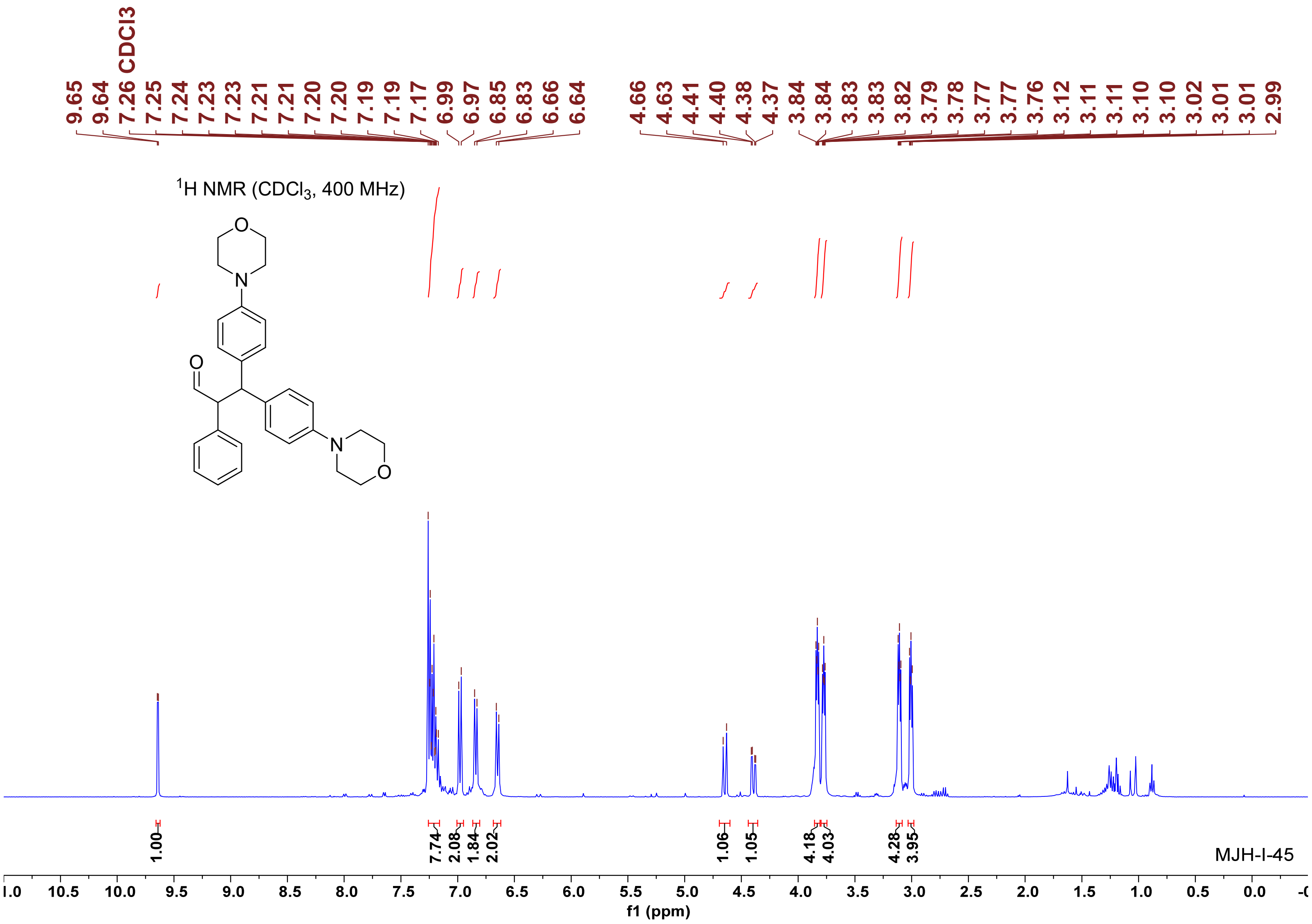

3,3-Bis(4-morpholinophenyl)-2-phenylpropanal (**7d**) (MJH-I-182)

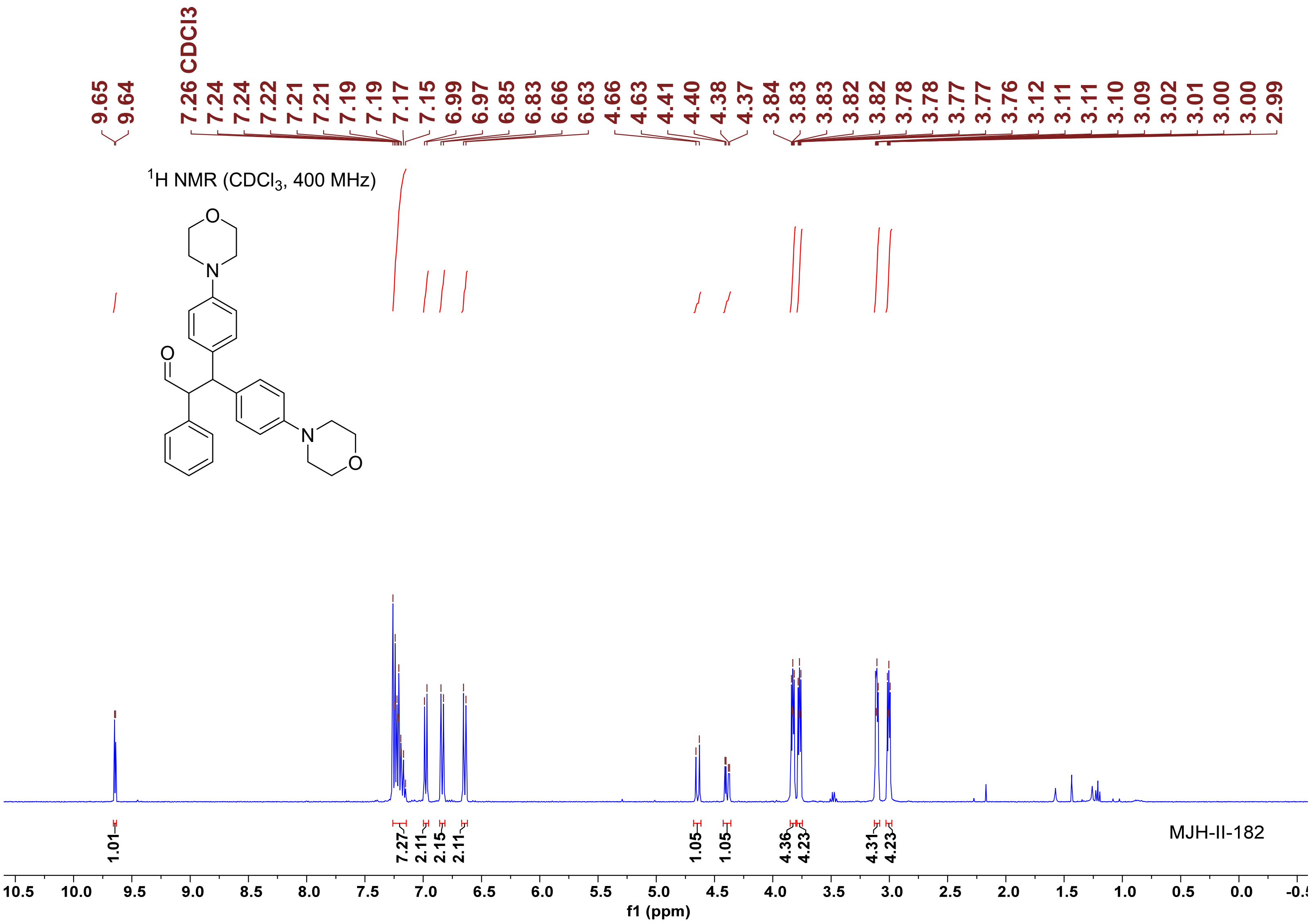

3,3-Bis(4-morpholinophenyl)-2-phenylpropanal (**7d**) (MJH-II-49)

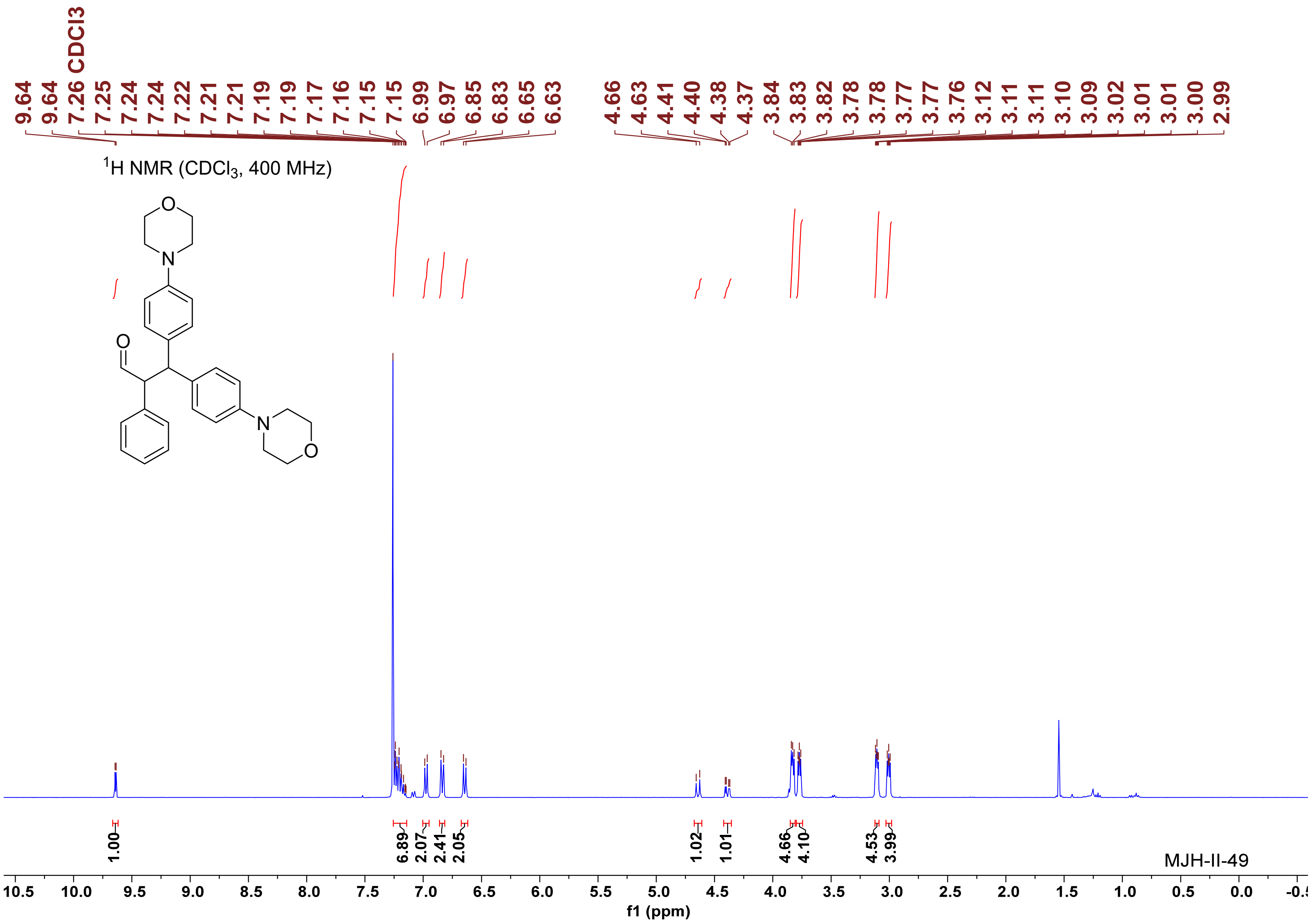

3,3-Bis(4-(dimethylamino)phenyl)-2-phenylpropanal (7e) (MJH-I-153)

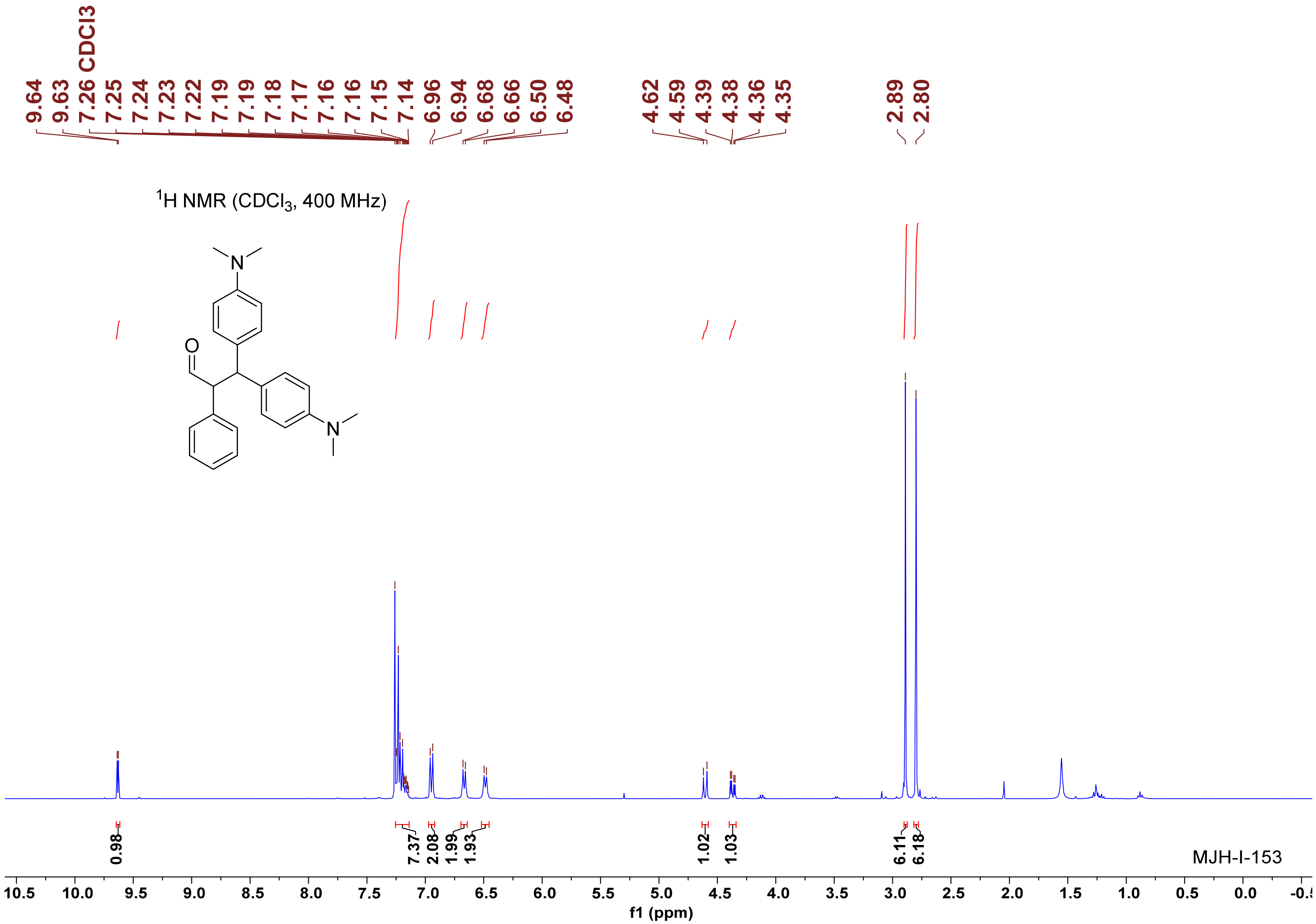

3,3-Bis(4-(dimethylamino)phenyl)-2-phenylpropanal (7e) (MJH-I-153)

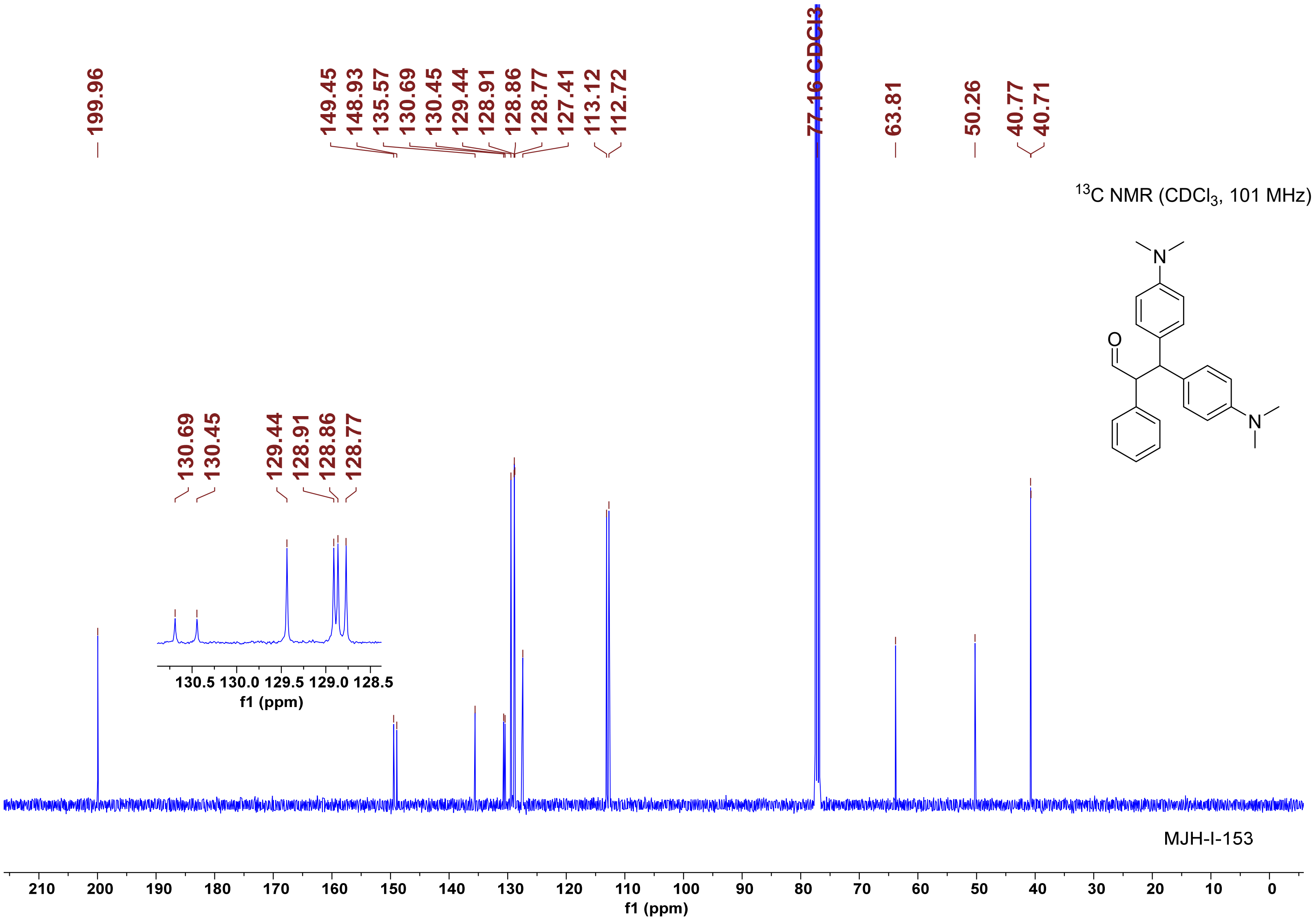

2-(Bis(1-methyl-1,2,3,4-tetrahydroquinolin-6-yl)methyl)butanal (7f) (MJH-II-22)

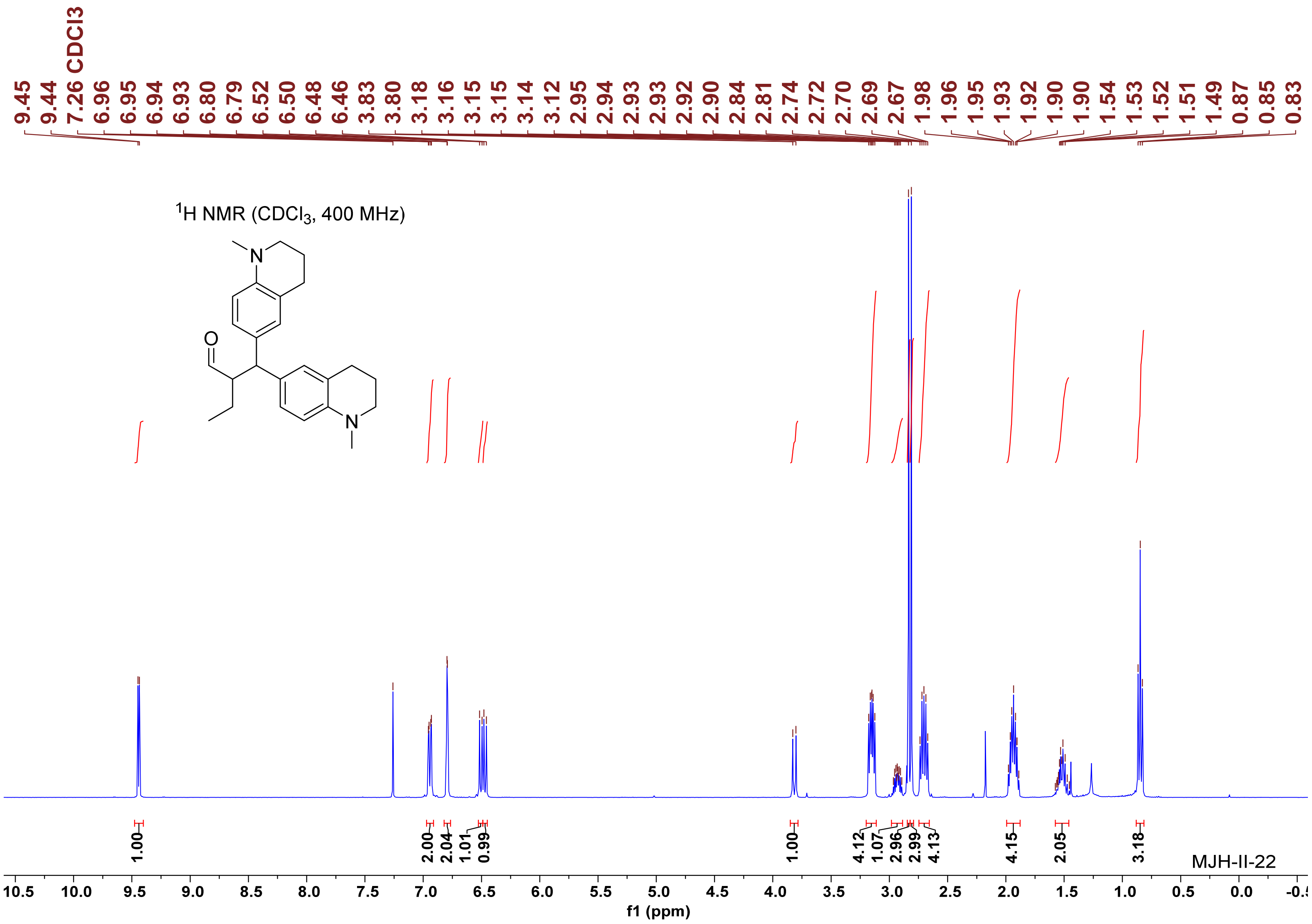

2-(Bis(1-methyl-1,2,3,4-tetrahydroquinolin-6-yl)methyl)butanal (**7f**) (MJH-II-22)

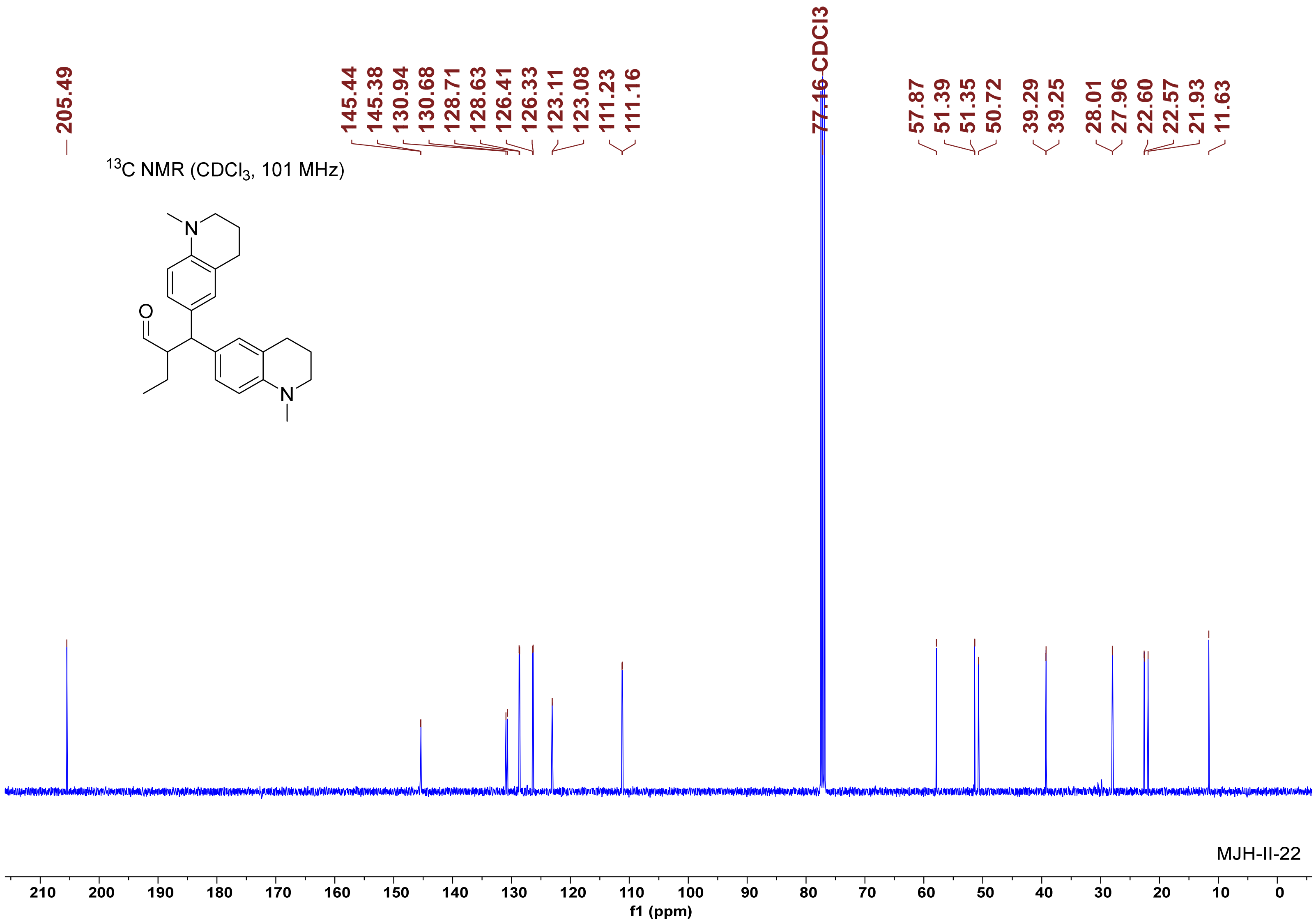

2-(Bis(1-methyl-1,2,3,4-tetrahydroquinolin-6-yl)methyl)butanal (**7f**) (MJH-II-22)

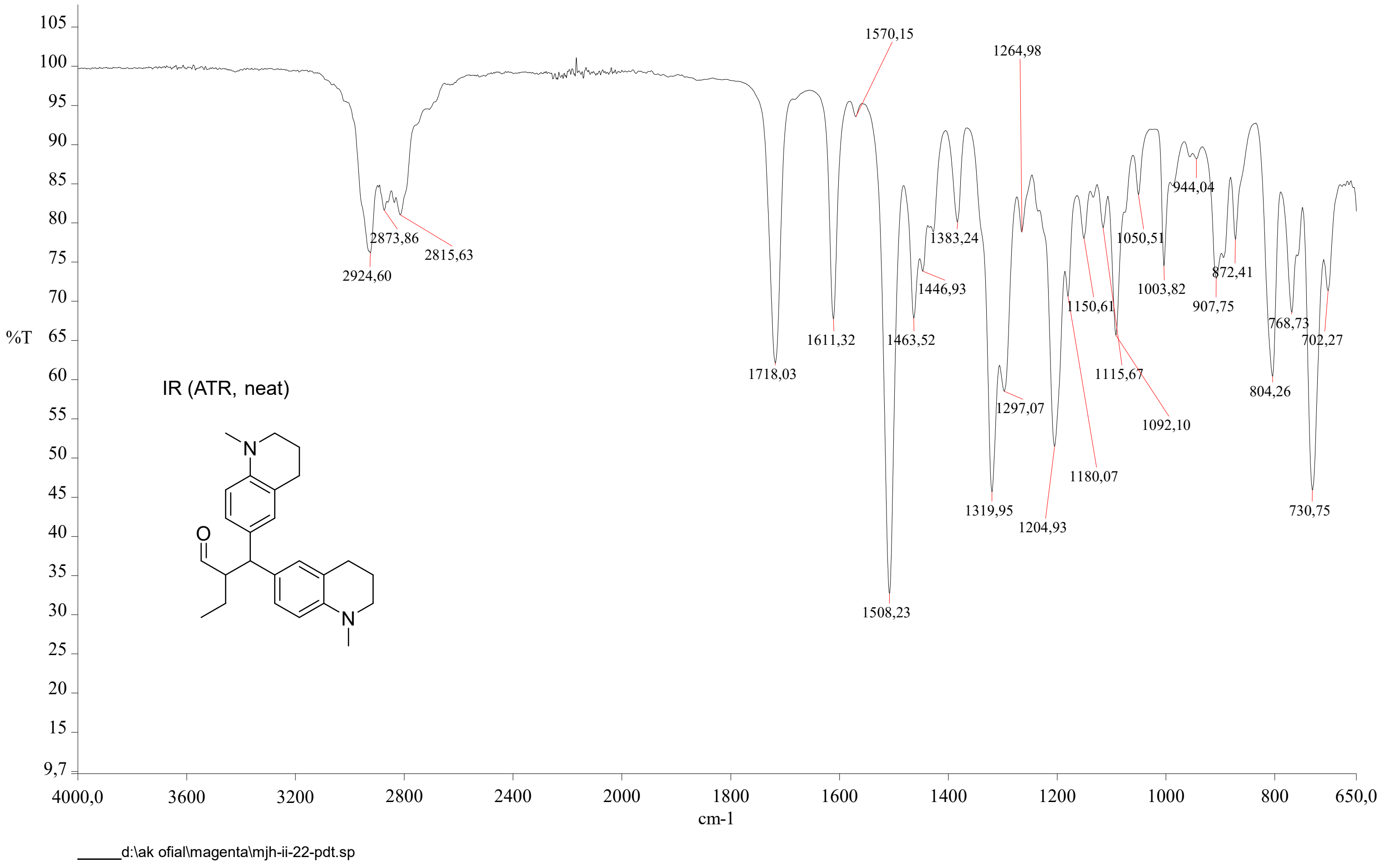

2-Benzyl-3,3-bis(4-(dimethylamino)phenyl)propanal (7g) (MJH-II-48)

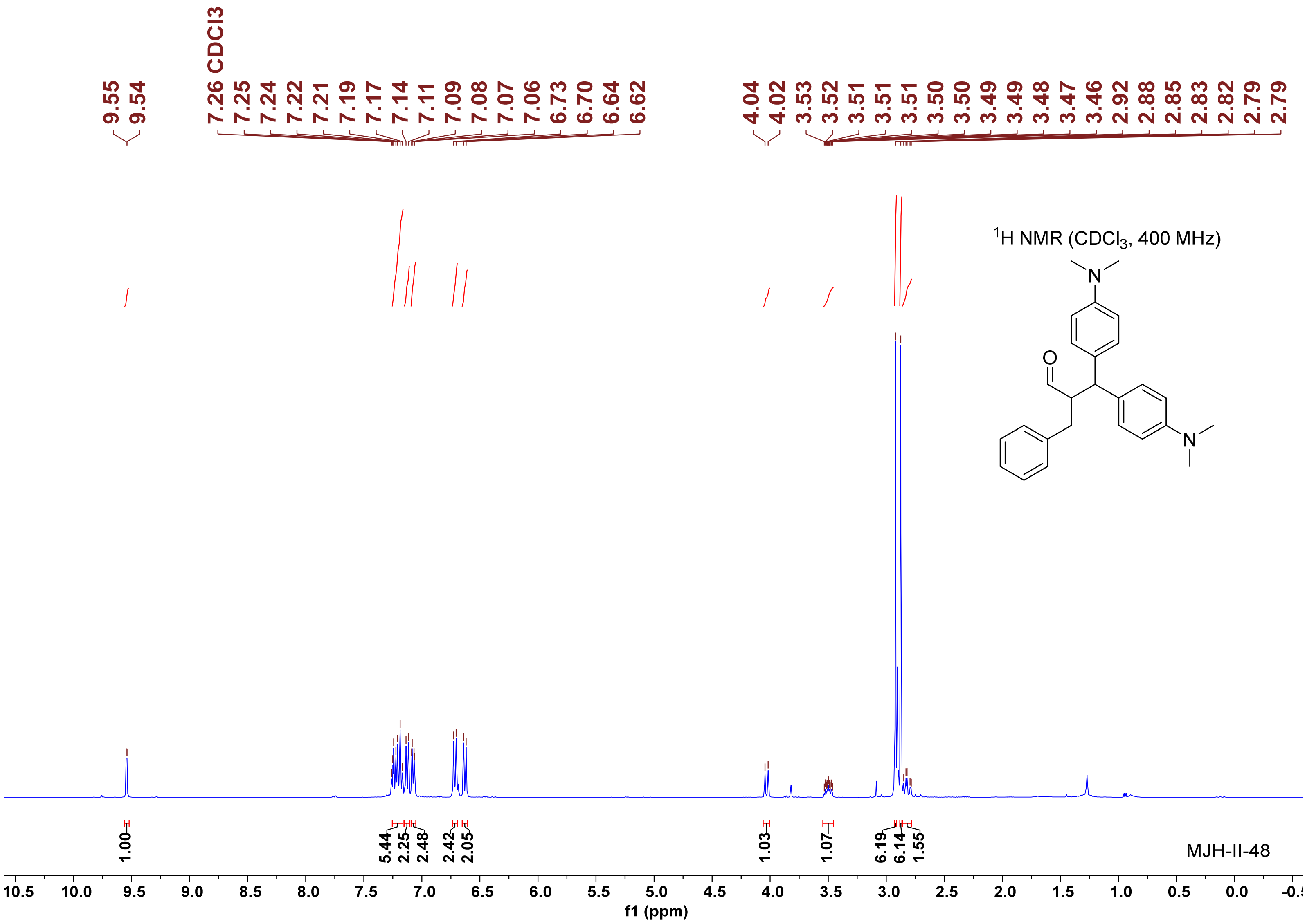

2-Benzyl-3,3-bis(4-(dimethylamino)phenyl)propanal (**7g**) (MJH-II-48)

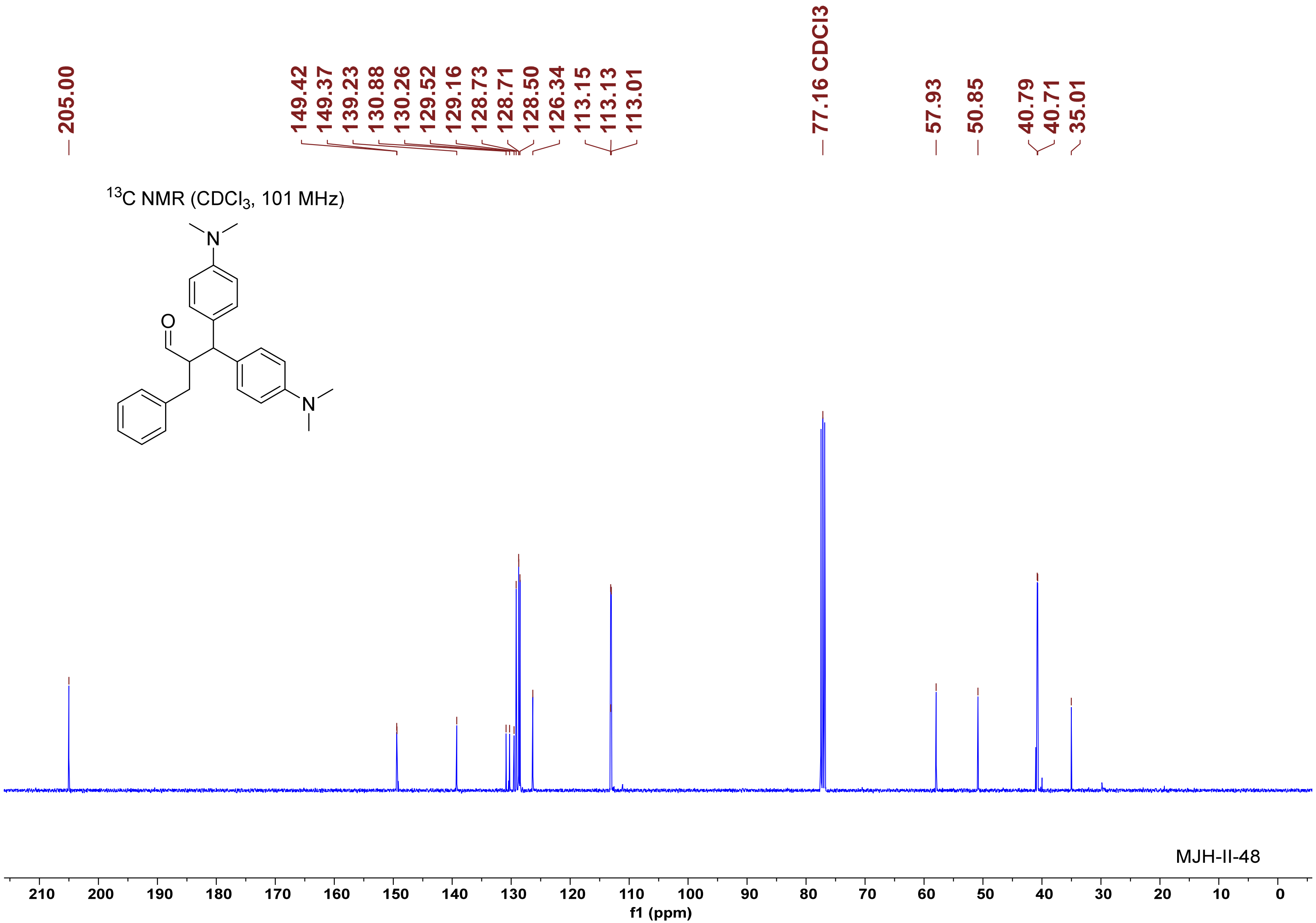

9. Computational Details

Methyl Cation Affinities (MCAs) are defined as the negative Gibbs energies ($-\Delta G_R$) of the reaction of a nucleophile with the methyl cation ($^+CH_3$).[S15,S16] In this work, MCAs were calculated by using the same DFT method employed earlier for enamines:[S7]

$$\text{Nu} + {}^{\oplus}\text{CH}_3 \xrightarrow[\text{MeCN, 25 °C}]{\text{MCA} = -\Delta G_\text{R}} \overset{\oplus}{\text{Nu}}-\text{CH}_3$$

An initial conformational search was carried out using the OPLS4 force field as implemented in MacroModel (release 2021-4).[S17] DFT calculations were performed using Gaussian09 (Rev. D.01).[S18] Geometry optimizations and frequency calculations were carried out in the gas phase using the B3LYP hybrid functional in combination with the 6-31G(d,p) basis set.[S19–S21] Thermochemical corrections to Gibbs free energies (ΔG_{corr}) were obtained from frequency calculations on the same level using the rigid rotor/harmonic oscillator model without scaling. The absence of imaginary vibrational frequencies (NImag=0) confirms that all optimized structures are true minima. Single point energies were obtained on the same level of theory with consideration of implicit solvation using the continuum solvation model based on density (SMD) for acetonitrile.[S22] Single point solvation energies (ΔG_{sol}) were obtained from single point calculations using the (SMD=acetonitrile)/B3LYP/6-31G(d,p) and B3LYP/6-311++G(3df,2pd) functional/basis set combinations. Finally, Gibbs energies (ΔG_R) were calculated by adding ΔG_{sol} and ΔG_{corr} to the total electronic energies calculated at the B3LYP/6-311++G(3df,2pd) level. All MCA values in Figure 4 (main text) are given as the Boltzmann-weighted averages of conformers with at least 1 % contribution to ΔG_{298}.

HOMO energies were obtained from single point calculations on the SMD(acetonitrile)/PBE0-D3/def2-TZVP//PBE0-D3/def2-TZVP level.[S23–S26] For visualization of the HOMOs (Figure 3, main text) based on intrinsic atomic orbitals (IAOs), the program IboView (v20211019-RevA) was used.[S27]

Table S40. Summary of final energies and thermochemical corrections for gas phase optimized structures of relevant conformers of enamines **4** as well as methylated products. $E_{SMD(MeCN)}$, ΔG and thermochemical corrections were calculated at the SMD(acetonitrile)/B3LYP/6-311++G(3df,2pd)//B3LYP/6-31G(d,p) level. ε_{HOMO} and ε_{LUMO} were obtained from a single point calculation at the SMD(acetonitrile)/PBE0-D3/def2-TZVP//PBE0-D3/def2-TZVP level. Values are given in hartrees.

label	filename	$E_{SMD(MeCN)}$	ZPE_{corr}	ΔE_{corr}	ΔH_{corr}	ΔG_{corr}	ΔG_{solv}	ε_{HOMO}	ε_{LUMO}
	methylcation	-39.4943410	0.031392	0.034245	0.035189	0.013991	-0.131697		
**4a**	SCN_Me_1	-1031.3721900	0.293875	0.312861	0.313805	0.246310	-0.018454	-0.19442	-0.01573
	SCN_Me_2	-1031.3721732	0.293828	0.312800	0.313744	0.246171	-0.018613	-0.19449	-0.01572
	SCN_Me_3	-1031.3719958	0.293653	0.312743	0.313687	0.245456	-0.017657	-0.19394	-0.00826
	SCN_Me_4	-1031.3719065	0.293622	0.312740	0.313684	0.245432	-0.017498	-0.19397	-0.00833
	SCN_Me_ME_1	-1071.0900077	0.335529	0.355824	0.356768	0.287100	-0.095155		
	SCN_Me_ME_2	-1071.0864820	0.335278	0.355742	0.356686	0.285928	-0.097625		
	SCN_Me_ME_3	-1071.0896921	0.335620	0.355916	0.356860	0.287063	-0.095155		
	SCN_Me_ME_4	-1071.0861525	0.335434	0.355830	0.356774	0.286312	-0.097402		
	SCN_Me_MS_1	-1071.0169258	0.331907	0.353168	0.354112	0.281787	-0.096334		
	SCN_Me_MS_2	-1071.0157956	0.332278	0.353344	0.354288	0.282785	-0.096334		
	SCN_Me_MS_3	-1071.0160798	0.332287	0.353367	0.354311	0.282796	-0.095888		
	SCN_Me_MS_4	-1071.0172657	0.332051	0.353261	0.354205	0.282241	-0.096414		
	SCN_Me_MS_5	-1071.0169988	0.332159	0.353267	0.354211	0.282850	-0.096780		
	SCN_Me_MS_6	-1071.0176168	0.332127	0.353341	0.354285	0.281949	-0.095203		
	SCN_Me_MS_7	-1071.0172181	0.332014	0.353264	0.354208	0.282091	-0.096127		
	SCN_Me_MS_8	-1071.0174150	0.332035	0.353238	0.354182	0.282081	-0.095346		
	SCN_Me_MN2_1	-1071.0480943	0.336791	0.356359	0.357304	0.290529	-0.093561		
	SCN_Me_MN2_2	-1071.0476344	0.337545	0.356841	0.357785	0.291399	-0.092175		
	SCN_Me_MN2_3	-1071.0478089	0.337566	0.356887	0.357831	0.291508	-0.092796		
	SCN_Me_MN2_4	-1071.0413728	0.336564	0.356101	0.357045	0.290882	-0.090964		
**4c**	SCN_Ph_1	-1223.1752095	0.347267	0.369208	0.370152	0.294393	-0.026932	-0.19288	-0.01935
	SCN_Ph_2	-1223.1749279	0.347121	0.369164	0.370108	0.293426	-0.025912	-0.19270	-0.01952
	SCN_Ph_3	-1223.1752916	0.347503	0.369334	0.370278	0.295065	-0.026980	-0.19250	-0.01970
	SCN_Ph_4	-1223.1752788	0.347361	0.369307	0.370251	0.294425	-0.026741	-0.19253	-0.02076
	SCN_Ph_5	-1223.1749113	0.347196	0.369203	0.370148	0.294227	-0.025753	-0.19272	-0.02017
	SCN_Ph_6	-1223.1752173	0.347362	0.369289	0.370233	0.294477	-0.026741	-0.19273	-0.02045
	SCN_Ph_ME_1	-1262.8849662	0.388548	0.411963	0.412908	0.334760	-0.097386		
	SCN_Ph_ME_2	-1262.8850062	0.388586	0.411959	0.412903	0.334894	-0.097227		
	SCN_Ph_ME_3	-1262.8816398	0.388468	0.411971	0.412915	0.334108	-0.099378		
	SCN_Ph_ME_4	-1262.8845383	0.388458	0.411914	0.412859	0.334547	-0.097274		
	SCN_Ph_ME_5	-1262.8845101	0.388555	0.411999	0.412944	0.334456	-0.097354		
	SCN_Ph_ME_6	-1262.8812808	0.388459	0.411967	0.412912	0.334220	-0.099282		
	SCN_Ph_MS_1	-1262.8162392	0.385578	0.409656	0.410600	0.330378	-0.105099		
	SCN_Ph_MS_2	-1262.8172685	0.385194	0.409488	0.410432	0.329241	-0.105593		
	SCN_Ph_MS_3	-1262.8168909	0.385317	0.409543	0.410487	0.329589	-0.105880		
	SCN_Ph_MS_4	-1262.8172025	0.385382	0.409600	0.410544	0.329895	-0.105609		
	SCN_Ph_MS_5	-1262.8174132	0.385440	0.409595	0.410539	0.330261	-0.104844		
	SCN_Ph_MS_6	-1262.8176315	0.385336	0.409584	0.410529	0.329723	-0.104685		
	SCN_Ph_MN2_1	-1262.8477939	0.390213	0.412743	0.413687	0.339061	-0.094358		
	SCN_Ph_MN2_2	-1262.8475088	0.390740	0.413188	0.414132	0.338318	-0.095936		

	SCN_Ph_MN2_3	-1262.8476963	0.390783	0.413207	0.414151	0.338522	-0.096494		
**4d**	SEt_1	-1170.2409471	0.377175	0.398784	0.399729	0.325795	-0.021323		
	SEt_2	-1170.2411921	0.377286	0.398828	0.399772	0.326193	-0.021609		
	SEt_3	-1170.2410396	0.377037	0.398697	0.399641	0.325559	-0.021466		
	SEt_4	-1170.2410054	0.377230	0.398820	0.399764	0.326051	-0.021339		
	SEt_5	-1170.2407542	0.377010	0.398761	0.399705	0.325222	-0.021833		
	SEt_6	-1170.2408218	0.376879	0.398691	0.399635	0.324151	-0.022008		
	SEt_7	-1170.2410396	0.377037	0.398697	0.399641	0.325559	-0.021466		
	SEt_8	-1170.2408218	0.376879	0.398691	0.399635	0.324153	-0.022008		
	SEt_9	-1170.2411920	0.377286	0.398828	0.399772	0.326193	-0.021609		
	SEt_10	-1170.2409470	0.377175	0.398784	0.399728	0.325795	-0.021323		
	SEt_11	-1170.2407542	0.377010	0.398761	0.399705	0.325222	-0.021833		
	Set_ME_1	-1209.9574570	0.418264	0.441470	0.442414	0.365301	-0.087521		
	Set_ME_2	-1209.9573993	0.418181	0.441421	0.442365	0.365003	-0.087394		
	Set_ME_3	-1209.9577652	0.418201	0.441530	0.442474	0.364878	-0.087251		
	Set_ME_4	-1209.9570614	0.418344	0.441515	0.442459	0.365560	-0.087458		
	Set_ME_5	-1209.9571394	0.418415	0.441562	0.442506	0.365788	-0.087490		
	Set_ME_6	-1209.9573873	0.418156	0.441479	0.442423	0.364734	-0.087282		
	SEt_MS_1	-1209.9034362	0.415212	0.439272	0.440217	0.360050	-0.095011		
	SEt_MS_2	-1209.9037420	0.415282	0.439346	0.440290	0.360200	-0.094486		
	SEt_MS_3	-1209.9034197	0.415359	0.439328	0.440272	0.360831	-0.094948		
	SEt_MS_4	-1209.9038565	0.415456	0.439420	0.440365	0.360588	-0.094741		
	SEt_MS_5	-1209.9035311	0.415350	0.439315	0.440259	0.360295	-0.094900		
	SEt_MS_6	-1209.9036054	0.415398	0.439379	0.440323	0.360340	-0.095043		
	SEt_MS_7	-1209.9039207	0.415480	0.439435	0.440379	0.361018	-0.094661		
	SEt_MS_8	-1209.9036420	0.415342	0.439338	0.440283	0.360624	-0.094597		
	SEt_MN2_1	-1209.9206044	0.419746	0.442192	0.443136	0.368500	-0.087984		
	SEt_MN2_2	-1209.9203158	0.419651	0.442102	0.443046	0.368584	-0.088047		
	SEt_MN2_3	-1209.9208031	0.419614	0.442171	0.443116	0.368075	-0.087808		
	SEt_MN2_4	-1209.9203093	0.420389	0.442628	0.443572	0.369070	-0.086948		
	SEt_MN2_5	-1209.9200370	0.420389	0.442626	0.443570	0.369210	-0.087107		
	SEt_MN2_6	-1209.9205356	0.420239	0.442622	0.443566	0.368180	-0.086948		
**4i**	OIm_1	-807.9645583	0.352863	0.372490	0.373434	0.304286	-0.022964	-0.19289	-0.01963
	OIm_2	-807.9646219	0.352962	0.372573	0.373517	0.304437	-0.022486	-0.19343	-0.01946
	OIm_ME_1	-847.6705160	0.394312	0.415436	0.416380	0.344617	-0.094852		
	OIm_MO_1	-847.6280728	0.393707	0.415342	0.416286	0.342964	-0.094549		
	OIm_MO_2	-847.6282195	0.393837	0.415357	0.416301	0.343513	-0.094310		
	OIm_MN_1	-847.6330157	0.396638	0.416694	0.417639	0.348991	-0.094294		
	OIm_MN_2	-847.6338195	0.395846	0.416171	0.417115	0.348169	-0.095123		
**4j**	SIm_1	-1130.9203216	0.350719	0.370636	0.371580	0.301774	-0.026374	-0.19487	-0.02253
	SIm_2	-1130.9196583	0.350585	0.370577	0.371521	0.301212	-0.027012	-0.19455	-0.02356
	SIm_ME_1	-1170.6246514	0.391820	0.413256	0.414201	0.341495	-0.099091		
	SIm_MS_1	-1170.6082667	0.389580	0.411767	0.412711	0.338223	-0.092318		
	SIm_MS_2	-1170.6081227	0.389660	0.411800	0.412744	0.338240	-0.091872		
	SIm_MS_3	-1170.6081740	0.389744	0.411901	0.412845	0.338244	-0.092255		
	SIm_MN_1	-1170.5877238	0.393587	0.414187	0.415131	0.344870	-0.098533		
	SIm_MN_2	-1170.5867332	0.393914	0.414433	0.415377	0.344587	-0.097466		

Geometries of Optimized Structures

Filenames (cf. Table S40), electronic energies E (“*SCF done energy*”, in hartrees), enthalpies H (in hartrees), Gibbs free energies G (in hartrees), number of the imaginary vibrational frequencies (NImag), and Cartesian coordinates of atoms (in Å) for all stationary points found at the B3LYP/6-31G(d,p) level of theory are given below.

```
methylcation.log
E =     -39.450436
H =     -39.449492
G =     -39.470690
NImag=0
C        0.0000000        0.0000000        0.0000000
H        0.0000000        1.0948150        0.0000000
H        0.9481370       -0.5474070        0.0000000
H       -0.9481370       -0.5474070        0.0000000
```

```
SCN_Me_1.log
E =     -1031.148407
H =     -1030.834602
G =     -1030.902097
NImag=0
C        0.5030565       -0.9862862        0.0257329
N        1.2932576        0.2449109       -0.1032437
C        0.4315695        1.4289989       -0.3484254
N       -0.9206331        0.8713584       -0.4627337
C       -0.8708040       -0.3832990       -0.2699241
S       -2.3032579       -1.4355302       -0.3265315
C       -3.5964130       -0.1485020       -0.5719125
C        0.8755232       -2.0491220       -1.0320201
C        0.5346565       -1.5808963        1.4529544
C        0.4492361        2.4236881        0.8257588
C        0.7835794        2.1323746       -1.6699326
C        2.6581124        0.3715204        0.0896121
C        3.5953409       -0.5508298        0.3787881
C        5.0496308       -0.2025824        0.5370828
C       -4.0281419        0.4844938        0.6708122
N       -4.3834849        0.9850594        1.6558700
H       -3.1835041        0.6084389       -1.2433156
H       -4.4406706       -0.6496931       -1.0515506
H        1.9025861       -2.3910378       -0.8937893
H        0.7879804       -1.6340249       -2.0393472
H        0.2166252       -2.9195208       -0.9551340
H        1.5507619       -1.8698290        1.7267104
H       -0.1030110       -2.4672478        1.5184072
H        0.1813027       -0.8475558        2.1823325
H        0.1672610        1.9217496        1.7544680
H       -0.2592935        3.2354308        0.6411901
H        1.4455647        2.8571425        0.9530821
H        0.7376445        1.4233464       -2.5003443
H        1.7916550        2.5552663       -1.6307991
H        0.0779971        2.9453652       -1.8607134
H        2.9926378        1.4003098       -0.0290950
H        3.3340181       -1.5963595        0.5085677
H        5.2207771        0.8697548        0.3926388
H        5.6825018       -0.7355202       -0.1856656
H        5.4291025       -0.4631100        1.5344974
```

```
SCN_Me_2.log
E =     -1031.148408
H =     -1030.834664
G =     -1030.902237
NImag=0
C       -0.3042798        1.2216442       -0.1424273
N       -1.2926343        0.1450287        0.0300271
C       -0.6498848       -1.1399319        0.3918192
N        0.7767826       -0.8037889        0.5082144
C        0.9427630        0.4244332        0.2319713
S        2.5293298        1.2282271        0.2620835
C        3.5859438       -0.2353386        0.6231042
C       -0.2238804        1.7329763       -1.5982755
C       -0.5384363        2.3978390        0.8295224
C       -1.1232121       -1.6626477        1.7591968
C       -0.7910650       -2.2072127       -0.7081253
C       -2.6374230        0.3580414       -0.2215890
C       -3.6819451       -0.4897474       -0.1733321
C       -5.0898404       -0.0567035       -0.4751832
C        3.9264147       -1.0110830       -0.5658577
N        4.2099473       -1.6264126       -1.5082668
H        4.4938718        0.1501051        1.0933074
H        3.0413200       -0.8673690        1.3289202
H       -1.1690385        2.2036624       -1.8852156
H       -0.0273948        0.9086549       -2.2883444
H        0.5692915        2.4778134       -1.7119648
H       -1.4908429        2.8886200        0.6079557
H        0.2509507        3.1503096        0.7364712
H       -0.5668017        2.0457814        1.8637733
H       -0.9471684       -0.9069755        2.5291447
H       -0.5673249       -2.5664293        2.0223406
H       -2.1889712       -1.8981280        1.7468442
H       -0.3847741       -1.8306440       -1.6501748
H       -1.8365269       -2.4780193       -0.8668972
H       -0.2338619       -3.1041215       -0.4256917
H       -2.8482656        1.3912587       -0.4919930
H       -3.5446929       -1.5337596        0.0895166
H       -5.7616942       -0.2140000        0.3796629
H       -5.5206801       -0.6166192       -1.3163216
H       -5.1341250        1.0068910       -0.7340588
```

```
SCN_Me_3.log
E =     -1031.148002
H =     -1030.834315
G =     -1030.902546
NImag=0
C       -0.3830613       -1.1412785       -0.0000076
N       -1.4469987       -0.1246156        0.0000034
C       -0.8973226        1.2500641       -0.0000033
N        0.5602424        1.0536265        0.0000010
C        0.8145874       -0.1926120       -0.0000028
S        2.4613166       -0.8488021        0.0000024
C        3.3702065        0.7577699        0.0000077
C       -0.4044540       -2.0226766        1.2677536
C       -0.4044572       -2.0226570       -1.2677829
C       -1.2670461        2.0307166       -1.2737559
C       -1.2670544        2.0307271        1.2737400
C       -2.7842634       -0.4844214       -0.0000002
C       -3.8950768        0.2761657        0.0000017
C       -5.2784322       -0.3130907        0.0000028
C        4.8061603        0.5095840        0.0000192
N        5.9504492        0.3160552        0.0000270
H        3.0760758        1.3307929       -0.8824884
H        3.0760631        1.3307948        0.8824983
H        0.4377784       -2.7211133        1.2774674
H       -0.3575778       -1.4064680        2.1690854
H       -1.3258660       -2.6114967        1.3025323
H       -0.3575823       -1.4064347       -2.1691054
H       -1.3258699       -2.6114757       -1.3025687
H        0.4377747       -2.7210940       -1.2775094
H       -2.3455599        2.1813278       -1.3481667
H       -0.7758086        3.0073005       -1.2649320
H       -0.9349800        1.4813651       -2.1584924
H       -0.9349921        1.4813837        2.1584829
H       -2.3455692        2.1813363        1.3481430
H       -0.7758192        3.0073122        1.2649102
H       -2.9211260       -1.5644717       -0.0000002
H       -3.8329576        1.3597994        0.0000051
H       -5.8583219       -0.0024964       -0.8797629
H       -5.2463598       -1.4081545       -0.0000005
H       -5.8583184       -0.0025019        0.8797728
```

```
SCN_Me_4.log
E =     -1031.147899
H =     -1030.834215
G =     -1030.902467
NImag=0
C       -0.5399236       -0.9076508        0.0000013
N       -1.4672562        0.2304798       -0.0000153
```

C	-0.7490576	1.5288192	-0.0000008
N	0.6706557	1.1573243	0.0000017
C	0.7669331	-0.1115011	0.0000064
S	2.3199625	-0.9658618	0.0000002
C	3.4217609	0.5147906	0.0000049
C	-0.6493690	-1.7742346	1.2754530
C	-0.6493509	-1.7742546	-1.2754378
C	-1.0378534	2.3513224	-1.2676790
C	-1.0378610	2.3513033	1.2676883
C	-2.8502541	0.1671941	-0.0000068
C	-3.6817013	-0.8917598	0.0000095
C	-5.1782047	-0.7453287	0.0000133
C	4.8155160	0.0890909	0.0000039
N	5.9267448	-0.2455924	0.0000004
H	3.2023998	1.1203392	-0.8825678
H	3.2023997	1.1203329	0.8825820
H	-0.4973751	-1.1625308	2.1685001
H	0.1012263	-2.5701202	1.2688174
H	-1.6348443	-2.2376208	1.3454148
H	-0.4973459	-1.1625641	-2.1684921
H	-1.6348241	-2.2376438	-1.3454063
H	0.1012454	-2.5701391	-1.2687794
H	-0.4308131	3.2606578	-1.2731867
H	-2.0916519	2.6412069	-1.3130007
H	-0.8016859	1.7661047	-2.1600164
H	-0.8017012	1.7660723	2.1600191
H	-2.0916595	2.6411883	1.3130062
H	-0.4308193	3.2606378	1.2732146
H	-3.3043584	1.1564929	-0.0000155
H	-3.2982218	-1.9072503	0.0000216
H	-5.4766111	0.3088054	0.0000029
H	-5.6380347	-1.2155729	0.8798708
H	-5.6380411	-1.2155922	-0.8798306

SCN_Me_ME_1.log
E = -1070.863585
H = -1070.506816
G = -1070.576485
NImag=0

C	-0.0785160	1.2900829	-0.2436578
N	1.0027290	0.2510217	0.0015548
C	0.3582483	-1.0388380	0.5843964
N	-1.0407573	-0.6913207	0.6793725
C	-1.2789557	0.4824821	0.2510108
S	-2.8876503	1.1951367	0.1686513
C	-3.8969949	-0.3009233	0.5614338
C	0.1704120	2.5442386	0.6087488
C	-0.1747920	1.6177175	-1.7423859
C	0.5171926	-2.2095731	-0.3930938
C	0.9022762	-1.3284252	1.9879629
C	2.2367882	0.4854867	-0.2739270
C	3.4354766	-0.3912065	-0.1285686
C	4.0688414	-0.5823134	-1.5275536
C	4.4249356	0.2910477	0.8458164
C	-3.7983985	-1.3215966	-0.4757951
N	-3.6927720	-2.1256544	-1.3055202
H	-3.5731315	-0.7048742	1.5227689
H	-4.9230915	0.0654834	0.6510319
H	0.2618938	2.2985633	1.6694497
H	1.0744337	3.0676027	0.2833267
H	-0.6664624	3.2369494	0.4901152
H	-1.0079906	2.3027855	-1.9131294
H	-0.3471312	0.7192633	-2.3397056
H	0.7363813	2.1120332	-2.0926861
H	-0.0814936	-3.0416337	-0.0165798
H	0.1342491	-1.9474704	-1.3819122
H	1.5528925	-2.5430050	-0.4792411
H	0.8053887	-0.4521844	2.6335722
H	1.9436676	-1.6542057	1.9742013
H	0.3008344	-2.1324768	2.4173856
H	2.4430263	1.4779807	-0.6800220
H	3.1558909	-1.3659720	0.2707465
H	4.3671505	0.3754625	-1.9656020
H	4.9663723	-1.1977709	-1.4269394
H	3.3876966	-1.0845572	-2.2195960
H	4.7332859	1.2749948	0.4785240
H	5.3206635	-0.3293610	0.9311004
H	3.9977298	0.4110397	1.8449713

SCN_Me_ME_2.log
E = -1070.859920
H = -1070.503234
G = -1070.573993
NImag=0

C	-0.0504103	1.1756468	-0.0000084
N	-1.1999331	0.1820670	-0.0000012
C	-0.6338954	-1.2643232	-0.0000021
N	0.7980637	-1.0600688	-0.0000035
C	1.1089613	0.1762071	-0.0000078
S	2.7517132	0.7929751	-0.0000085
C	3.6643390	-0.8183099	-0.0000040
C	-0.0803446	2.0330903	-1.2750376
C	-0.0803374	2.0330999	1.2750144
C	-1.0382142	-1.9997864	1.2831453
C	-1.0382152	-1.9997867	-1.2831491
C	-2.4260027	0.5711296	0.0000064
C	-3.6874291	-0.2261067	0.0000182
C	-4.4871340	0.1277553	1.2768340
C	-4.4871530	0.1277475	-1.2767877
C	5.0940006	-0.5403457	-0.0000132
N	6.2307867	-0.3099494	-0.0000095
H	3.3774227	-1.3864430	0.8878068
H	3.3774167	-1.3864516	-0.8878074
H	-0.0755160	1.4142026	-2.1753833
H	-0.9619516	2.6808171	-1.2909636
H	0.8006486	2.6784261	-1.3009977
H	0.8006562	2.6784357	1.3009648
H	-0.0755040	1.4142191	2.1753649
H	-0.9619441	2.6808272	1.2909405
H	-0.4903815	-2.9440549	1.3126442
H	-2.1055230	-2.2247596	1.3179532
H	-0.7618748	-1.4210382	2.1677401
H	-2.1055237	-2.2247607	-1.3179561
H	-0.7618768	-1.4210385	-2.1677441
H	-0.4903820	-2.9440549	-1.3126484
H	-2.5699790	1.6536269	0.0000055
H	-3.4714377	-1.2944928	0.0000200
H	-3.9450483	-0.1415946	2.1872319
H	-4.7254572	1.1953620	1.3162484
H	-5.4300828	-0.4247445	1.2666033
H	-4.7254765	1.1953541	-1.3162052
H	-5.4301018	-0.4247519	-1.2665395
H	-3.9450810	-0.1416082	-2.1871921

SCN_Me_ME_3.log
E = -1070.863281
H = -1070.506421
G = -1070.576218
NImag=0

C	0.2326543	-0.9829426	0.0032332
N	1.0037186	0.3103046	-0.1381859
C	0.0209368	1.4654669	-0.5065698
N	-1.2525231	0.8007594	-0.6023289
C	-1.1646077	-0.4390115	-0.3323591
S	-2.5453315	-1.5340056	-0.2903539
C	-3.9087990	-0.2941190	-0.4146085
C	0.6797864	-2.0266322	-1.0359291
C	0.2896155	-1.5092333	1.4486327
C	-0.0066866	2.4987706	0.6240904
C	0.4125813	2.0699045	-1.8575705
C	2.2631040	0.5171022	0.0183291
C	3.3655191	-0.4237906	0.3748160
C	4.3956034	-0.4291232	-0.7800082
C	4.0095784	0.0605719	1.6961211
C	-3.9718112	0.5749656	0.7550369
N	-3.9919397	1.2638692	1.6883849
H	-3.7674808	0.2987948	-1.3204872
H	-4.8223585	-0.8870458	-0.5094369
H	0.6622531	-1.6133073	-2.0472559
H	1.6793029	-2.4108856	-0.8247799
H	-0.0088518	-2.8749518	-1.0070927
H	-0.3831820	-2.3643159	1.5437655
H	-0.0351495	-0.7473008	2.1612022
H	1.2929975	-1.8449145	1.7165307
H	-0.7705395	3.2429970	0.3898707
H	-0.2731621	2.0343324	1.5760571
H	0.9526768	3.0145544	0.7234699
H	0.4549232	1.3042614	-2.6359098
H	1.3735594	2.5893622	-1.8065240
H	-0.3490321	2.8003100	-2.1382884
H	2.5761331	1.5515103	-0.1331508
H	2.9837884	-1.4352841	0.5154208
H	4.8092070	0.5698058	-0.9506952
H	5.2226806	-1.0917878	-0.5128720
H	3.9598591	-0.7888456	-1.7159661
H	4.4137512	1.0727653	1.5953900
H	4.8374578	-0.6060174	1.9508181
H	3.2979215	0.0521942	2.5259666

SCN_Me_ME_4.log
E = -1070.859610
H = -1070.502836
G = -1070.573298
NImag=0

C	-0.2862815	-0.8354841	-0.0000044
N	-1.2283782	0.3459606	0.0000048

C	-0.4036864	1.6703092	-0.0000016
N	0.9637110	1.2152144	-0.0000114
C	1.0400489	-0.0571115	-0.0000144
S	2.5447408	-0.9620302	-0.0000173
C	3.7337449	0.4573490	-0.0000189
C	-0.4353463	-1.6732110	1.2832223
C	-0.4353671	-1.6732084	-1.2832304
C	-0.7031163	2.4625185	-1.2759065
C	-0.7030982	2.4625185	1.2759076
C	-2.5145300	0.3495553	0.0000179
C	-3.4898256	-0.7807178	0.0000281
C	-4.3579812	-0.6751169	1.2767438
C	-4.3580074	-0.6751176	-1.2766698
C	5.0891194	-0.0758514	-0.0000235
N	6.1649674	-0.5093494	-0.0000274
H	3.5548040	1.0681960	-0.8878211
H	3.5548098	1.0681939	0.8877858
H	-0.3441631	-1.0506853	2.1765783
H	-1.3891865	-2.2027797	1.3137449
H	0.3588212	-2.4228302	1.3139946
H	0.3587996	-2.4228278	-1.3140171
H	-0.3441980	-1.0506809	-2.1765866
H	-1.3892079	-2.2027768	-1.3137389
H	-0.0420058	3.3311757	-1.3043314
H	-1.7351904	2.8235633	-1.2962372
H	-0.5130546	1.8598068	-2.1671665
H	-1.7351721	2.8235631	1.2962530
H	-0.5130237	1.8598069	2.1671649
H	-0.0419874	3.3311758	1.3043230
H	-2.9671564	1.3428991	0.0000231
H	-2.9731030	-1.7405534	0.0000232
H	-3.7609925	-0.7723645	2.1874582
H	-4.8983821	0.2759346	1.3148046
H	-5.0976105	-1.4796786	1.2674308
H	-4.8984093	0.2759337	-1.3147200
H	-5.0976363	-1.4796794	-1.2673412
H	-3.7610375	-0.7723655	-2.1873965

SCN_Me_MS_1.log
E = -1070.784951
H = -1070.430839
G = -1070.503164
NImag=0

C	-0.3025335	-0.8765419	-0.7328621
N	-1.4068288	-0.2193034	-0.0149330
C	-0.9422623	0.6669211	1.0735287
N	0.5286105	0.4386491	1.1016417
C	0.7995567	-0.3514348	0.1662135
S	2.5767453	-0.8141956	-0.0944773
C	3.3528120	0.8656519	0.0985929
C	-0.3856509	-2.4181030	-0.6831993
C	-0.1147693	-0.3886511	-2.1880285
C	-1.1466172	2.1642668	0.7805108
C	-1.4928439	0.2554494	2.4479950
C	-2.7214418	-0.3259134	-0.4847186
C	-3.8422679	0.2647718	-0.0434437
C	-5.1905207	0.0123226	-0.6577321
C	2.9827944	-1.5601860	1.5250359
C	3.0396563	1.6722020	-1.0699516
N	2.7506992	2.2951636	-2.0048009
H	2.9315442	1.3053336	1.0079458
H	4.4297842	0.7023154	0.2004015
H	-0.5105688	-2.7726889	0.3431808
H	0.5024229	-2.8824628	-1.1279581
H	-1.2450122	-2.7625999	-1.2639960
H	0.7586652	-0.8542096	-2.6575777
H	-0.0066434	0.6973136	-2.2360922
H	-0.9871581	-0.6700475	-2.7832366
H	-0.6555168	2.4435456	-0.1550272
H	-0.7374282	2.7696417	1.5926451
H	-2.2100166	2.3854076	0.6818515
H	-1.2437577	-0.7870752	2.6610695
H	-2.5792144	0.3507905	2.4608438
H	-1.0763783	0.8928192	3.2314012
H	-2.8039826	-1.0136746	-1.3227139
H	-3.8211770	0.9570527	0.7929362
H	-5.8933640	-0.4038881	0.0742469
H	-5.1280297	-0.6898843	-1.4948709
H	-5.6398055	0.9395343	-1.0333455
H	2.5195505	-2.5481721	1.5397222
H	2.5833452	-0.9266980	2.3181047
H	4.0683078	-1.6599232	1.5812772

SCN_Me_MS_2.log
E = -1070.783673
H = -1070.429385
G = -1070.500888
NImag=0

C	0.5088322	-1.2272614	0.0636313
N	1.4785680	-0.1327910	-0.1090800
C	0.8924096	1.1983216	0.1646342
N	-0.5562237	0.9127816	0.3166920
C	-0.7123476	-0.3295139	0.2261746
S	-2.4077012	-1.0107125	0.4842576
C	-3.3271096	0.5259181	0.9997255
C	0.4310068	-2.1381229	-1.1792910
C	0.7317593	-2.0747589	1.3405943
C	1.3592597	1.8238935	1.4917183
C	1.0418891	2.1634526	-1.0216672
C	2.8474421	-0.4172024	-0.2057872
C	3.8973283	0.4169138	-0.2416075
C	5.3142890	-0.0644986	-0.3799354
C	-3.0361767	-1.3212038	-1.2091554
C	-3.5703330	1.4422384	-0.0997883
N	-3.7876898	2.1318174	-1.0071513
H	-4.2622872	0.1673348	1.4397476
H	-2.7014732	0.9818652	1.7710503
H	0.1949873	-1.5638285	-2.0786725
H	-0.3011601	-2.9427417	-1.0462515
H	1.3973183	-2.6222004	-1.3390607
H	-0.0614275	-2.8177947	1.4768692
H	0.7845840	-1.4435557	2.2311145
H	1.6749338	-2.6207306	1.2581921
H	1.1476175	1.1553843	2.3303622
H	0.8526244	2.7769585	1.6597802
H	2.4351432	1.9995091	1.4655837
H	0.5079167	3.0948312	-0.8207240
H	0.6413494	1.7100470	-1.9312261
H	2.0943550	2.3933098	-1.1920586
H	3.0439443	-1.4835014	-0.2848202
H	3.7633418	1.4926416	-0.1800802
H	5.7872542	0.3374672	-1.2841426
H	5.3672134	-1.1563413	-0.4338436
H	5.9316068	0.2576244	0.4673540
H	-4.0815792	-1.6185896	-1.1058416
H	-2.9344029	-0.4192568	-1.8137284
H	-2.4520804	-2.1472502	-1.6141371

SCN_Me_MS_3.log
E = -1070.783943
H = -1070.429631
G = -1070.501146
NImag=0

C	0.7020512	-0.9503299	0.1298125
N	1.4770966	0.2768727	-0.0878299
C	0.6779409	1.5074771	0.1253456
N	-0.7033161	0.9967711	0.2945728
C	-0.6525543	-0.2568837	0.2531429
S	-2.2154019	-1.1953478	0.5412545
C	-3.3865372	0.1917914	0.9576155
C	0.7714333	-1.9083604	-1.0793203
C	1.0224432	-1.6908711	1.4536748
C	1.0535054	2.2679268	1.4093014
C	0.6874044	2.4334142	-1.1002710
C	2.8677115	0.3480008	-0.2308429
C	3.7922841	-0.6241407	-0.2105613
C	5.2552252	-0.3595968	-0.4329562
C	-2.7588979	-1.7065476	-1.1328641
C	-3.7501667	0.9978667	-0.1939130
N	-4.0547942	1.5947095	-1.1410381
H	-4.2604659	-0.2936004	1.4019864
H	-2.8640684	0.7840905	1.7129037
H	0.2129188	-2.8320463	-0.8921599
H	1.8075730	-2.1926789	-1.2647946
H	0.3981153	-1.4247452	-1.9857803
H	0.9396023	-1.0170339	2.3101458
H	2.0410777	-2.0789856	1.4328378
H	0.3493754	-2.5414986	1.6052392
H	0.9867783	1.6172983	2.2850394
H	0.3956648	3.1284867	1.5521255
H	2.0816075	2.6298098	1.3325584
H	0.3874527	1.8868738	-1.9971537
H	1.6914446	2.8336596	-1.2599679
H	0.0060889	3.2733242	-0.9467298
H	3.2035706	1.3659313	-0.4113736
H	3.5180357	-1.6605131	-0.0361696
H	5.4531967	0.7058124	-0.5851097
H	5.6327553	-0.8962064	-1.3119189
H	5.8564721	-0.6925417	0.4216168
H	-2.0378035	-2.4434989	-1.4852033
H	-2.8035337	-0.8377666	-1.7906476
H	-3.7401705	-2.1704732	-1.0147732

SCN_Me_MS_4.log
E = -1070.785357
H = -1070.431151

G = -1070.503116
NImag=0

C	0.5409599	-0.9138824	-0.2628081
N	1.4056798	0.2297656	0.0487193
C	0.7299490	1.5332633	-0.1696884
N	-0.6792551	1.1538851	-0.4305890
C	-0.7342695	-0.0998616	-0.4405574
S	-2.3612174	-0.9043219	-0.7736802
C	-3.0709934	-1.1105381	0.9433300
C	0.8807854	-1.6429115	-1.5874722
C	0.4297517	-1.9102080	0.9122656
C	0.7494713	2.4203060	1.0842294
C	1.2431762	2.2906278	-1.4072118
C	2.7883698	0.1704994	0.2572406
C	3.6169233	-0.8846716	0.2733961
C	5.0869128	-0.7600150	0.5620329
C	-3.3341150	0.4949307	-1.4238692
C	-3.0571813	0.1204540	1.7117623
N	-3.0291418	1.1207756	2.2991911
H	-4.0872643	-1.4882650	0.7927274
H	-2.4711596	-1.8918781	1.4170292
H	0.9193268	-0.9394292	-2.4229253
H	0.1432144	-2.4209316	-1.8116223
H	1.8553699	-2.1261062	-1.5142243
H	0.0944290	-1.4043289	1.8220060
H	1.4049574	-2.3491356	1.1250316
H	-0.2472987	-2.7384074	0.6707183
H	0.3230846	1.8898244	1.9382609
H	0.1786555	3.3354150	0.9113762
H	1.7775128	2.6994508	1.3273628
H	1.1831873	1.6655714	-2.3017365
H	2.2883668	2.5718770	-1.2577480
H	0.6619082	3.2015259	-1.5685601
H	3.2094528	1.1516501	0.4605708
H	3.2538807	-1.8903820	0.0820809
H	5.6921594	-1.1454296	-0.2673456
H	5.3725444	-1.3323610	1.4530525
H	5.3771295	0.2815942	0.7301195
H	-3.1492638	1.3766394	-0.8098594
H	-4.3808369	0.1860553	-1.4176605
H	-2.9936787	0.6562606	-2.4475270

SCN_Me_MS_5.log
E = -1070.785126
H = -1070.430916
G = -1070.502276
NImag=0

C	-0.3336106	-1.1853807	0.0754564
N	-1.3859064	-0.1660361	-0.0609275
C	-0.9371599	1.1774444	0.3714445
N	0.5201397	1.0016948	0.5933626
C	0.7863415	-0.2108641	0.4081897
S	2.5278183	-0.7739781	0.6424916
C	3.2540980	-0.5725261	-1.0677253
C	-0.5577736	-2.1786878	1.2409360
C	-0.0804737	-1.9486881	-1.2420707
C	-1.0997351	2.2356942	-0.7311192
C	-1.5368973	1.6304858	1.7151023
C	-2.7140717	-0.5530925	-0.2837413
C	-3.8312707	0.1886643	-0.3138093
C	-5.1861411	-0.3926261	-0.6054310
C	3.2523649	0.6410815	1.5366473
C	3.0364116	0.7507635	-1.6222508
N	2.8447480	1.8174810	-2.0368117
H	4.3191904	-0.8003713	-0.9586479
H	2.7888382	-1.3507999	-1.6781512
H	-0.7253598	-1.6515210	2.1832814
H	0.2920029	-2.8596550	1.3587986
H	-1.4386175	-2.7927444	1.0370855
H	0.7393244	-2.6703353	-1.1392225
H	0.1273428	-1.2588870	-2.0645299
H	-0.9678290	-2.5263803	-1.5118857
H	-2.1543483	2.3608398	-0.9797228
H	-0.7014867	3.1956156	-0.3952350
H	-0.5690718	1.9273035	-1.6345188
H	-1.3240958	0.8994655	2.4993767
H	-2.6197713	1.7264850	1.6290945
H	-1.1212266	2.5971552	2.0080111
H	-2.8059913	-1.6180782	-0.4827989
H	-3.7998741	1.2591208	-0.1349746
H	-5.8845891	-0.2169190	0.2216495
H	-5.1350447	-1.4728302	-0.7740931
H	-5.6336516	0.0631000	-1.4970296
H	2.8890061	0.5738981	2.5629478
H	2.9226151	1.5685383	1.0679990
H	4.3360300	0.5145760	1.5100623

SCN_Me_MS_6.log
E = -1070.785722
H = -1070.431436
G = -1070.503772
NImag=0

C	-0.7505273	-0.8932765	-0.1256832
N	-1.6675915	0.2399241	0.0315428
C	-0.9685186	1.5305954	0.2335979
N	0.4678109	1.1505688	0.2320069
C	0.5254336	-0.0902021	0.0542123
S	2.1884475	-0.9002232	0.0027429
C	3.1126412	0.3973079	-0.9633438
C	-0.8807512	-1.9615074	0.9857785
C	-0.7831225	-1.5396487	-1.5318714
C	-1.1915293	2.5231390	-0.9194831
C	-1.2784167	2.1710188	1.5963444
C	-3.0639848	0.1790094	-0.0324495
C	-3.8707657	-0.8724484	-0.2395402
C	-5.3690462	-0.7562052	-0.2686572
C	2.7942192	-0.5903943	1.7024663
C	4.5419323	0.2169253	-0.7784179
N	5.6740973	0.0517879	-0.5849319
H	2.8196376	0.2543496	-2.0074237
H	2.7571506	1.3687239	-0.6049340
H	-0.1131153	-2.7369673	0.8801371
H	-1.8499253	-2.4574783	0.9233656
H	-0.8045651	-1.5052544	1.9763014
H	-0.6340725	-0.7875621	-2.3110609
H	-1.7511392	-2.0101448	-1.7073780
H	-0.0183545	-2.3179024	-1.6285275
H	-0.9150987	2.0746640	-1.8769232
H	-0.6044277	3.4315652	-0.7646572
H	-2.2468670	2.8020309	-0.9671126
H	-1.0753806	1.4687333	2.4085116
H	-2.3337718	2.4499044	1.6428428
H	-0.6797864	3.0733887	1.7414133
H	-3.5217886	1.1531439	0.1192980
H	-3.4667639	-1.8684363	-0.3963746
H	-5.8356298	-1.3760874	0.5065727
H	-5.7792388	-1.0914196	-1.2289322
H	-5.6959063	0.2760233	-0.1098899
H	2.2738984	-1.2950992	2.3528644
H	2.5745222	0.4424041	1.9755843
H	3.8662773	-0.7964412	1.7034586

SCN_Me_MS_7.log
E = -1070.785096
H = -1070.430888
G = -1070.503005
NImag=0

C	0.5107775	-0.8957090	0.1473989
N	1.4365432	0.1779895	-0.2358313
C	0.7398648	1.4019500	-0.7000281
N	-0.6861254	0.9853179	-0.7611725
C	-0.7464150	-0.1882338	-0.3237568
S	-2.4098999	-1.0049305	-0.2410883
C	-3.4145664	0.4249715	0.3953081
C	0.7316527	-2.2039567	-0.6458615
C	0.4314498	-1.1657972	1.6699784
C	0.8320007	2.5751865	0.2915240
C	1.1709975	1.8203396	-2.1133475
C	2.8147436	0.1889492	0.0078022
C	3.6051948	-0.7633266	0.5249905
C	5.0887711	-0.5884910	0.6911916
C	-2.8896772	-0.9937699	-2.0059908
C	-3.0967868	0.6440953	1.7970783
N	-2.8036192	0.8065376	2.9075409
H	-3.1459565	1.2942440	-0.2127297
H	-4.4653597	0.1540422	0.2562136
H	0.7545004	-2.0079102	-1.7211109
H	-0.0512235	-2.9381639	-0.4221516
H	1.6841528	-2.6588262	-0.3716589
H	1.3961219	-1.5183410	2.0364132
H	-0.3099330	-1.9394557	1.8959637
H	0.1746179	-0.2581730	2.2212596
H	1.8736286	2.8893197	0.3926135
H	0.2507747	3.4285044	-0.0663386
H	0.4658910	2.2826212	1.2787041
H	1.0605615	0.9873711	-2.8117167
H	2.2205602	2.1239818	-2.1080464
H	0.5735913	2.6665595	-2.4610094
H	3.2757300	1.1228863	-0.3026848
H	3.1987201	-1.7181034	0.8455350
H	5.4195278	0.3954265	0.3447005
H	5.6479861	-1.3450506	0.1273572
H	5.3903372	-0.6909796	1.7406262
H	-3.9494915	-1.2489070	-2.0624652
H	-2.6785152	-0.0098967	-2.4269435
H	-2.2965460	-1.7671226	-2.4968688

SCN_Me_MS_8.log
E = -1070.785577
H = -1070.431395
G = -1070.503497
NImag=0

C	-0.5969486	-1.1383048	-0.1909744
N	-1.6484687	-0.1406718	0.0600743
C	-1.1139827	1.2275840	0.2307734
N	0.3601097	1.0245421	0.2334472
C	0.5715217	-0.1947955	0.0245811
S	2.3211651	-0.7919436	-0.0428701
C	3.1021811	0.6819522	-0.8718698
C	-0.6003608	-2.2888081	0.8392187
C	-0.5921275	-1.7020614	-1.6319362
C	-1.4279676	2.1695820	-0.9456119
C	-1.4899549	1.8426322	1.5879890
C	-2.9988786	-0.4840733	-0.0832728
C	-4.0943025	0.2869880	-0.0134530
C	-5.4854383	-0.2616675	-0.1643036
C	2.8504744	-0.5577393	1.6947210
C	4.5360563	0.6680946	-0.6412109
N	5.6733920	0.6316545	-0.4139566
H	2.6120669	1.5694396	-0.4585211
H	2.8636705	0.5853881	-1.9349408
H	-0.5818179	-1.9021885	1.8614592
H	0.2468674	-2.9678356	0.6861152
H	-1.5073541	-2.8875476	0.7243458
H	0.2623045	-2.3664028	-1.8016574
H	-0.5758372	-0.8978239	-2.3718096
H	-1.4964715	-2.2927851	-1.7983766
H	-1.0752518	1.7413031	-1.8873659
H	-0.9486792	3.1393627	-0.7932063
H	-2.5045855	2.3220446	-1.0282706
H	-1.1692846	1.1894869	2.4032006
H	-2.5717747	1.9622289	1.6581189
H	-1.0212624	2.8223718	1.7051680
H	-3.1358618	-1.5487417	-0.2552110
H	-4.0197336	1.3555822	0.1647513
H	-6.0887837	-0.0762828	0.7325794
H	-5.4768275	-1.3415348	-0.3418219
H	-6.0136006	0.2099894	-1.0017080
H	2.3966812	-1.3648686	2.2716647
H	2.5124331	0.4196730	2.0409584
H	3.9384605	-0.6453024	1.7129866

SCN_Me_MN2_1.log
E = -1070.821514
H = -1070.464210
G = -1070.530984
NImag=0

C	-0.2108006	-0.8230548	0.9133999
N	-1.2583294	0.3027061	0.5210636
C	-0.2930935	1.4292667	-0.2541965
N	0.7917790	0.6197097	-0.7237775
C	0.8595392	-0.5093188	-0.1378278
S	2.1559823	-1.6716159	-0.4061610
C	3.3394167	-0.5922336	-1.3277536
C	0.3651916	-0.6374111	2.3304576
C	-0.8069046	-2.2341863	0.8128781
C	-1.0538165	2.0879645	-1.4026889
C	0.2171130	2.5020730	0.7136029
C	-2.1915295	-0.2502464	-0.4826522
C	-3.5201056	-0.2802279	-0.4018622
C	-4.3870732	-0.8550958	-1.4816419
C	-1.9876425	0.8708977	1.6941141
C	3.8152827	0.5207758	-0.5141035
N	4.1599653	1.4080587	0.1493949
H	4.1646673	-1.2516071	-1.6095387
H	2.8515034	-0.2192087	-2.2305413
H	0.8035834	0.3441994	2.5046662
H	1.1662185	-1.3686795	2.4579544
H	-0.3885793	-0.8420406	3.0944767
H	-0.0544166	-2.9474714	1.1597010
H	-1.0953484	-2.5195164	-0.1978513
H	-1.6760289	-2.3358992	1.4682047
H	-1.2840305	1.3981277	-2.2130856
H	-1.9758037	2.5596865	-1.0545514
H	-0.4075223	2.8702744	-1.8077174
H	0.7334557	2.0970321	1.5825974
H	-0.5806308	3.1756147	1.0338383
H	0.9531010	3.0912123	0.1617745
H	-1.6693913	-0.6469519	-1.3439046
H	-4.0422452	0.1183555	0.4630173
H	-3.8043272	-1.2410980	-2.3214019
H	-5.0780952	-0.0938680	-1.8604665
H	-5.0032997	-1.6687937	-1.0835219
H	-1.2831492	1.2893381	2.4037512
H	-2.5671565	0.0827405	2.1753667
H	-2.6570819	1.6553179	1.3427241

SCN_Me_MN2_2.log
E = -1070.820965
H = -1070.463180
G = -1070.529566
NImag=0

C	0.2303445	-1.1971720	-0.0608490
N	1.1258346	0.0978096	-0.2801008
C	0.3468030	1.2238840	0.6938426
N	-0.9874213	0.7059039	0.7401170
C	-1.0735480	-0.4990658	0.3382836
S	-2.5982932	-1.3759758	0.2191490
C	-3.7662906	0.0295830	0.4917005
C	0.1356967	-2.0698509	-1.3189520
C	0.7344057	-2.0826732	1.0960289
C	0.9721415	1.2852545	2.0906118
C	0.3737361	2.6157378	0.0625680
C	2.5302378	-0.1079887	0.0977460
C	3.5930101	0.0633755	-0.6904630
C	4.9983896	-0.1546056	-0.2184615
C	0.9979301	0.5510779	-1.7087482
C	-3.6597445	1.0246988	-0.5703061
N	-3.5279685	1.8010610	-1.4226790
H	-4.7607377	-0.4242752	0.5050089
H	-3.5637271	0.4859915	1.4625541
H	1.1314897	-2.3838336	-1.6451944
H	-0.3912859	-1.5974764	-2.1471726
H	-0.4157218	-2.9775864	-1.0624530
H	1.6724057	-2.5759798	0.8319119
H	-0.0126003	-2.8621241	1.2645794
H	0.8578031	-1.5511023	2.0383495
H	0.3406291	1.9480628	2.6864498
H	0.9976580	0.3237480	2.6015367
H	1.9760748	1.7135918	2.0606994
H	-0.2203003	2.6834096	-0.8480715
H	1.3963180	2.9569355	-0.1187802
H	-0.0777638	3.2974938	0.7868254
H	2.6491297	-0.4171922	1.1251406
H	3.4855579	0.3754558	-1.7249976
H	5.0428214	-0.4707803	0.8263077
H	5.5831004	0.7655534	-0.3285378
H	5.4925438	-0.9148404	-0.8335767
H	-0.0536366	0.7027674	-1.9433808
H	1.4203698	-0.2027483	-2.3673157
H	1.5387641	1.4840635	-1.8341523

SCN_Me_MN2_3.log
E = -1070.820937
H = -1070.463106
G = -1070.529429
NImag=0

C	-0.2021822	-1.1375594	-0.2231749
N	-1.2602947	-0.1119783	0.3715576
C	-0.3286400	1.2831903	0.5364681
N	0.9907322	0.7471614	0.6644726
C	1.0801284	-0.4605540	0.2715550
S	2.6005578	-1.3473850	0.1834619
C	3.7813476	0.0581507	0.3949077
C	-0.3969550	-2.5682822	0.2983676
C	-0.2220049	-1.1862772	-1.7636834
C	-0.4337202	2.1748458	-0.7048489
C	-0.7409897	2.0785804	1.7724813
C	-2.4222761	0.0892881	-0.5056935
C	-3.7009777	-0.1054680	-0.1786518
C	-4.8297325	0.1403467	-1.1332101
C	-1.6793257	-0.5617939	1.7418321
C	3.6909289	1.0205233	-0.6972828
N	3.5823821	1.7799749	-1.5677947
H	3.5835201	0.5496840	1.3496258
H	4.7709212	-0.4055441	0.4262617
H	-1.3909416	-2.9442152	0.0396349
H	-0.2330962	-2.6773175	1.3702545
H	0.3264563	-3.2124651	-0.2078786
H	-1.1433328	-1.6474947	-2.1261544
H	0.6137436	-1.8118317	-2.0838251
H	-0.0909242	-0.2151031	-2.2385093
H	0.2934139	2.9791088	-0.5722524
H	-0.1672689	1.6658485	-1.6298844
H	-1.4255628	2.6219073	-0.7965033
H	-0.5339052	1.5597955	2.7079232
H	-1.7930828	2.3719251	1.7278486
H	-0.1435622	2.9931499	1.7722655
H	-2.1507190	0.4390003	-1.4898394
H	-3.9865938	-0.4559281	0.8087323
H	-4.4818498	0.4863580	-2.1092661
H	-5.5179963	0.8884783	-0.7242060
H	-5.4126792	-0.7762974	-1.2763782

H	-0.7954918	-0.6807372	2.3644997
H	-2.2083436	-1.5081536	1.6675186
H	-2.3363175	0.1852854	2.1771155

SCN_Me_MN2_4.log
E = -1070.815818
H = -1070.458773
G = -1070.524936
NImag=0

C	-0.6456156	-0.9969637	-0.9402340
N	-1.5822961	-0.0385982	-0.0865296
C	-0.8976404	-0.1337225	1.4293605
N	0.4613264	-0.4865260	1.1259063
C	0.6108925	-0.9033690	-0.0681298
S	2.1728521	-1.3622840	-0.7479199
C	3.2704939	-0.7082073	0.5864741
C	-1.1521895	-2.4543695	-0.9716728
C	-0.4758422	-0.5264676	-2.3908860
C	-0.9860025	1.1993458	2.1726401
C	-1.5604917	-1.2243483	2.2825037
C	-1.5774369	1.3621728	-0.6071142
C	-0.5156851	2.1570299	-0.7278220
C	-0.6045876	3.5559952	-1.2548996
C	-3.0191886	-0.4743242	-0.0909669
C	3.1627518	0.7441586	0.6818870
N	3.0190822	1.8951410	0.7259149
H	3.0045632	-1.1733773	1.5376227
H	4.2822617	-1.0097262	0.3031218
H	-2.0517977	-2.5494188	-1.5844560
H	-1.3357157	-2.8824122	0.0120949
H	-0.3759999	-3.0624320	-1.4427313
H	0.0826319	0.4001320	-2.4906084
H	-1.4506130	-0.4035522	-2.8717451
H	0.0603841	-1.3040378	-2.9406503
H	-0.3166007	1.9562313	1.7728970
H	-2.0083586	1.5863133	2.1823828
H	-0.6895528	1.0088421	3.2067748
H	-1.5846485	-2.2041352	1.8082554
H	-2.5687435	-0.9398883	2.5915837
H	-0.9534217	-1.3215391	3.1851824
H	-2.5673973	1.6968031	-0.8926992
H	0.4775343	1.8231587	-0.4351090
H	0.0318144	3.6691041	-2.1398378
H	-0.2247377	4.2633860	-0.5092794
H	-1.6260023	3.8393176	-1.5196809
H	-3.1212588	-1.4820552	0.2940983
H	-3.3985366	-0.4283471	-1.1130010
H	-3.5865615	0.2162451	0.5334250

SCN_Ph_1.log
E = -1222.895941
H = -1222.525790
G = -1222.601548
NImag=0

C	-1.0424688	1.2036137	-0.0857134
N	-0.0869572	0.1089002	0.1697700
C	-0.7842035	-1.1608671	0.5021468
N	-2.2053609	-0.7893216	0.5220737
C	-2.3275952	0.4371106	0.2164383
S	-3.8947586	1.2710855	0.1269779
C	-5.0028897	-0.1649062	0.4421791
C	-0.8487322	2.3890703	0.8829195
C	-1.0090739	1.6884673	-1.5511755
C	-0.6012815	-2.2407048	-0.5791680
C	-0.4078775	-1.6777487	1.9004959
C	1.2673727	0.2992446	0.0279367
C	2.2855129	-0.5872538	0.1419596
C	-5.2540686	-0.9740354	-0.7466580
N	-5.4659279	-1.6156226	-1.6903268
C	3.7044000	-0.2542867	-0.0096866
C	4.2099643	1.0623289	-0.0035196
C	5.5682210	1.3151717	-0.1721332
C	6.4739602	0.2638546	-0.3352283
C	5.9967255	-1.0474723	-0.3237644
C	4.6364883	-1.3001267	-0.1635338
H	-4.5343151	-0.7815846	1.2128893
H	-5.9402433	0.2511726	0.8194630
H	-0.8884418	2.0523105	1.9218302
H	-1.6210962	3.1491964	0.7308965
H	0.1207628	2.8665502	0.7127565
H	-0.0337376	2.1279752	-1.7806097
H	-1.7718605	2.4517935	-1.7301399
H	-1.1816144	0.8574222	-2.2394017
H	-0.9151855	-1.8557074	-1.5525288
H	-1.2201283	-3.1080898	-0.3360767
H	0.4389743	-2.5623028	-0.6538100
H	-0.6147238	-0.9107190	2.6511905
H	0.6505005	-1.9380828	1.9575101
H	-1.0008530	-2.5654149	2.1352174
H	1.5152422	1.3295281	-0.2125442
H	2.0770214	-1.6340243	0.3324902
H	3.5374815	1.9005983	0.1550277
H	5.9247511	2.3417706	-0.1627325
H	7.5337454	0.4644725	-0.4597843
H	6.6858656	-1.8792366	-0.4424597
H	4.2784865	-2.3268864	-0.1650174

SCN_Ph_2.log
E = -1222.895448
H = -1222.525341
G = -1222.602022
NImag=0

C	-0.9560427	-1.0451589	0.1007719
N	0.0439535	0.0363829	0.0219572
C	-0.5976395	1.3712606	-0.0886946
N	-2.0372080	1.0769876	-0.0803737
C	-2.2116450	-0.1788370	0.0217542
S	-3.8107962	-0.9391704	0.0742589
C	-4.8250492	0.5975532	-0.0591175
C	-0.8687539	-2.0185323	-1.0942714
C	-0.8822479	-1.8177558	1.4347624
C	-0.2984892	2.2698190	1.1243384
C	-0.2627553	2.0703994	-1.4171360
C	1.3912051	-0.2387832	0.0262168
C	2.4511584	0.6042462	-0.0115432
C	-6.2406094	0.2553327	-0.0085662
N	-7.3687800	-0.0136578	0.0314803
C	3.8538179	0.1791563	-0.0211567
C	4.2754554	-1.1344965	-0.3133046
C	5.6228462	-1.4827315	-0.2878578
C	6.5994552	-0.5294473	0.0114420
C	6.2046500	0.7814182	0.2822957
C	4.8557858	1.1278242	0.2653368
H	-4.5588427	1.2687780	0.7608259
H	-4.5825824	1.1030499	-0.9968386
H	-0.9406036	-1.4783609	-2.0416001
H	-1.6717987	-2.7603000	-1.0539920
H	0.0825079	-2.5586586	-1.0771277
H	0.0763724	-2.3386289	1.5170297
H	-1.6762251	-2.5676842	1.4991286
H	-0.9759968	-1.1367930	2.2843497
H	-0.5915192	1.7631503	2.0474019
H	-0.8692065	3.1983638	1.0408213
H	0.7624069	2.5172273	1.1898042
H	-0.5467129	1.4321184	-2.2577430
H	0.8038663	2.2877856	-1.4957905
H	-0.8190453	3.0085414	-1.4879863
H	1.5938585	-1.3052150	0.0763342
H	2.2962707	1.6775211	-0.0083610
H	3.5443223	-1.8903217	-0.5857400
H	5.9142475	-2.5040480	-0.5181680
H	7.6501891	-0.8028158	0.0228371
H	6.9497564	1.5388466	0.5101283
H	4.5626870	2.1513580	0.4863693

SCN_Ph_3.log
E = -1222.896001
H = -1222.525723
G = -1222.600936
NImag=0

C	1.0433100	1.2045343	0.1207088
N	0.0871383	0.0829017	0.1814188
C	0.7803177	-1.2215261	0.3447123
N	2.2037497	-0.8624147	0.3938228
C	2.3284614	0.3960446	0.2789659
S	3.8980123	1.2301249	0.2974903
C	5.0041952	-0.2388727	0.3861431
C	1.0057758	1.9406361	-1.2357055
C	0.8541536	2.2012953	1.2839484
C	0.4278044	-1.9119153	1.6739912
C	0.5675540	-2.1518580	-0.8612525
C	-1.2653924	0.2936863	0.0524039
C	-2.2899955	-0.5916815	0.0962331
C	5.2518875	-0.8561476	-0.9134103
N	5.4614293	-1.3449986	-1.9449863
C	-3.7039846	-0.2446936	-0.0688938
C	-4.1596662	0.9952217	-0.5631647
C	-5.5189125	1.2696008	-0.6822210
C	-6.4731605	0.3123344	-0.3280910
C	-6.0429949	-0.9277731	0.1456313
C	-4.6828954	-1.1995756	0.2720422
H	5.9431547	0.1123934	0.8209080
H	4.5352011	-0.9656202	1.0538334
H	1.1669935	1.2432936	-2.0613808
H	1.7749382	2.7169837	-1.2808291
H	0.0350090	2.4247051	-1.3777393

```
H       -0.1237540       2.6864838       1.2110537
H        1.6172032       2.9851808       1.2572570
H        0.9123220       1.6897664       2.2480121
H        0.6248954      -1.2374120       2.5112655
H        1.0443362      -2.8064794       1.7937166
H       -0.6227196      -2.2053169       1.7088863
H        0.9040008      -1.6605683      -1.7774480
H       -0.4846155      -2.4176031      -0.9777921
H        1.1501980      -3.0665152      -0.7263256
H       -1.5060370       1.3427915      -0.0965027
H       -2.0948408      -1.6408369       0.2882136
H       -3.4446861       1.7498341      -0.8785517
H       -5.8362069       2.2350711      -1.0673140
H       -7.5326081       0.5276004      -0.4287788
H       -6.7691723      -1.6881717       0.4200256
H       -4.3630921      -2.1682674       0.6486023
```

```
SCN_Ph_4.log
E =     -1222.895976
H =     -1222.525726
G =     -1222.601551
NImag=0
C        0.8635048      -0.9929323       0.0622429
N        0.0938096       0.2472742       0.2541492
C        0.9850468       1.4150615       0.4990600
N        2.3346985       0.8446246       0.4856218
C        2.2611109      -0.4028964       0.2568611
S        3.6862182      -1.4602116       0.1576090
C        5.0030731      -0.1859151       0.3331333
C        0.5703122      -2.0565490       1.1442330
C        0.7114932      -1.5769816      -1.3599704
C        0.8896440       2.4642822      -0.6213928
C        0.7315541       2.0494251       1.8763173
C       -1.2696627       0.3923958       0.1600536
C       -2.2337590      -0.5363440      -0.0504842
C        5.2952461       0.5207251      -0.9105159
N        5.5396669       1.0793049      -1.8980782
C       -3.6679047      -0.2492605      -0.1399263
C       -4.2051424       1.0461746      -0.2903280
C       -5.5797130       1.2548103      -0.3532375
C       -6.4681323       0.1785080      -0.2846332
C       -5.9563518      -1.1131396      -0.1542334
C       -4.5810514      -1.3212830      -0.0836040
H        4.6759628       0.5286860       1.0920647
H        5.8926493      -0.7157282       0.6826120
H        0.7228210      -1.6399782       2.1431525
H        1.2324184      -2.9192231       1.0228397
H       -0.4589983      -2.4119244       1.0770690
H        1.0160777      -0.8437916      -2.1110426
H       -0.3257796      -1.8536003      -1.5552766
H        1.3324380      -2.4694969      -1.4778786
H        1.0948953       2.0050608      -1.5913025
H        1.6218962       3.2575094      -0.4509641
H       -0.1061492       2.9160020      -0.6525871
H        0.8274854       1.2983549       2.6644547
H       -0.2724368       2.4804813       1.9289850
H        1.4574221       2.8460292       2.0593513
H       -1.5782089       1.4256078       0.2908941
H       -1.9714851      -1.5847737      -0.1401578
H       -3.5421530       1.9020417      -0.3791388
H       -5.9606141       2.2659583      -0.4696547
H       -7.5396413       0.3446910      -0.3408024
H       -6.6304348      -1.9640782      -0.1055032
H       -4.1973034      -2.3330580       0.0244480
```

```
SCN_Ph_5.log
E =     -1222.895387
H =     -1222.525239
G =     -1222.601160
NImag=0
C        0.8002204      -0.8031615      -0.0343575
N       -0.0454294       0.3991685       0.0416425
C        0.7698619       1.6404030       0.1371374
N        2.1561430       1.1662182       0.0896415
C        2.1609873      -0.1028743      -0.0036811
S        3.6469477      -1.0632087      -0.0906053
C        4.8540610       0.3308910      -0.0069884
C        0.6459632      -1.7304236       1.1929321
C        0.6109694      -1.5803080      -1.3554464
C        0.5354280       2.5795261      -1.0583157
C        0.5448162       2.3747464       1.4693415
C       -1.4195108       0.4426428       0.0280637
C       -2.3239567      -0.5658390      -0.0015260
C        6.2123417      -0.1943018      -0.0607977
N        7.2954227      -0.6088826      -0.1031708
C       -3.7775945      -0.3822265      -0.0336360
C       -4.4089234       0.8352804      -0.3618226
C       -5.7957981       0.9535952      -0.3562764
C       -6.6030211      -0.1425072      -0.0416593
C       -5.9982456      -1.3622572       0.2650210
C       -4.6103554      -1.4783572       0.2679939
H        4.6685972       1.0106311      -0.8419971
H        4.6906621       0.8869289       0.9191843
H        0.8252561      -1.1763782       2.1179052
H        1.3607261      -2.5568623       1.1409079
H       -0.3578262      -2.1553373       1.2405613
H        0.8048093      -0.9311426      -2.2131666
H       -0.4089010      -1.9590416      -1.4397460
H        1.2958983      -2.4318497      -1.4034855
H        0.7133694       2.0503135      -1.9977945
H        1.2183886       3.4309943      -1.0002815
H       -0.4889804       2.9629679      -1.0642781
H        0.7409811       1.7046735       2.3101042
H       -0.4852105       2.7348390       1.5465875
H        1.2164708       3.2343108       1.5416342
H       -1.7947142       1.4619116       0.0526555
H       -1.9928132      -1.5980316       0.0278178
H       -3.8109778       1.6960317      -0.6472179
H       -6.2509281       1.9061208      -0.6143153
H       -7.6847208      -0.0486404      -0.0458837
H       -6.6091438      -2.2281960       0.5053292
H       -4.1530478      -2.4331247       0.5165830
```

```
SCN_Ph_6.log
E =     -1222.895928
H =     -1222.525696
G =     -1222.601452
NImag=0
C       -0.8653802      -0.9699309       0.1757831
N       -0.0926033       0.2829271       0.1825601
C       -0.9815670       1.4759140       0.2437048
N       -2.3310253       0.9127715       0.3399088
C       -2.2599785      -0.3552101       0.3066056
S       -3.6850533      -1.4130560       0.3991325
C       -5.0000265      -0.1249084       0.4031492
C       -0.7448695      -1.7478256      -1.1545830
C       -0.5453453      -1.8708333       1.3887272
C       -0.7163945       2.3252493       1.4978049
C       -0.8960461       2.3290826      -1.0329059
C        1.2732747       0.4088649       0.0968403
C        2.2310804      -0.5412536      -0.0307313
C       -5.3193977       0.3816600      -0.9282964
N       -5.5855336       0.7813156      -1.9849301
C        3.6707423      -0.2747206      -0.0897937
C        4.2596758       0.9551299       0.2704524
C        5.6340920       1.1527003       0.1748823
C        6.4729295       0.1261490      -0.2664691
C        5.9121924      -1.1050046      -0.6089761
C        4.5359956      -1.3001143      -0.5212120
H       -5.8812543      -0.5927314       0.8489599
H       -4.6577917       0.6976179       1.0355782
H       -1.0257658      -1.1118441      -1.9978337
H       -1.4016142      -2.6224324      -1.1485801
H        0.2779172      -2.0925395      -1.3141459
H       -0.7081226      -1.3277258       2.3232291
H        0.4945737      -2.2003333       1.3642244
H       -1.1828392      -2.7601078       1.3878139
H       -0.8010189       1.7106929       2.3976540
H       -1.4451024       3.1378686       1.5569273
H        0.2845892       2.7654025       1.4704780
H       -1.1111474       1.7187203      -1.9132746
H        0.1016470       2.7636488      -1.1451043
H       -1.6247815       3.1424131      -0.9879701
H        1.5886772       1.4474556       0.1400262
H        1.9549672      -1.5855587      -0.1266550
H        3.6401626       1.7627845       0.6496676
H        6.0558329       2.1128056       0.4602302
H        7.5454031       0.2819444      -0.3330745
H        6.5482649      -1.9180099      -0.9484834
H        4.1125909      -2.2625151      -0.7989356
```

```
SCN_Ph_ME_1.log
E =     -1262.603287
H =     -1262.190380
G =     -1262.268527
NImag=0
C       -1.0504196       1.1322122       0.5648064
N       -0.1075092      -0.0357756       0.3304474
C       -0.8092143      -1.0969937      -0.5582855
N       -2.1207233      -0.5288558      -0.7756552
C       -2.2599193       0.5976935      -0.2019443
S       -3.7500006       1.5377293      -0.2254266
C       -4.8728433       0.2961591      -1.0051412
C       -1.3411673       1.3018135       2.0642024
C       -0.4841431       2.4152328      -0.0645961
C       -0.0941436      -1.2341492      -1.9068178
```

C	-0.9574661	-2.4219722	0.1981036
C	1.0686362	-0.0609504	0.8538929
C	2.1560713	-1.0838636	0.7447322
C	2.4382507	-1.6845288	2.1433917
C	-5.0963154	-0.8638435	-0.1495512
N	-5.2498320	-1.7841578	0.5400737
C	3.3659915	-0.3642091	0.1327449
C	4.0423225	0.6490667	0.8287927
C	5.1430114	1.2846997	0.2554589
C	5.5804496	0.9165580	-1.0181154
C	4.9137960	-0.0909336	-1.7165822
C	3.8077610	-0.7219908	-1.1474982
H	-5.8081019	0.8331164	-1.1841643
H	-4.4467900	-0.0149735	-1.9611099
H	-0.4419838	1.6087731	2.6067482
H	-2.0899809	2.0844242	2.2039102
H	-1.7282379	0.3795975	2.5040486
H	-1.2100371	3.2252223	0.0417443
H	0.4339881	2.7263412	0.4425127
H	-0.2731889	2.2820735	-1.1282266
H	-0.7146229	-1.8607605	-2.5510208
H	0.0232711	-0.2607522	-2.3887698
H	0.8846070	-1.7051786	-1.8111837
H	-1.4465801	-2.2676614	1.1625649
H	0.0001832	-2.9233921	0.3500444
H	-1.5974803	-3.0765350	-0.3971820
H	1.3201560	0.8049316	1.4661878
H	1.8593431	-1.8891523	0.0733394
H	3.2773122	-2.3793265	2.0625725
H	1.5695661	-2.2290052	2.5242986
H	2.7081741	-0.9150349	2.8710720
H	3.7324147	0.9371071	1.8302033
H	5.6629625	2.0615640	0.8068158
H	6.4399910	1.4089812	-1.4612030
H	5.2553242	-0.3896116	-2.7024776
H	3.3036533	-1.5140240	-1.6947461

SCN_Ph_ME_2.log
E = -1262.603380
H = -1262.190477
G = -1262.268486
NImag=0

C	1.0986398	-0.9338082	-0.9577485
N	0.1101668	-0.6799076	0.1673379
C	0.8448611	-0.0105525	1.3589294
N	2.2235040	0.0123796	0.9238960
C	2.3636689	-0.4383820	-0.2571962
S	3.9001563	-0.4835554	-1.1185257
C	4.9265241	0.5080404	0.0534377
C	1.1725707	-2.4299448	-1.2986237
C	0.7388141	-0.0767676	-2.1823238
C	0.3637652	1.4322421	1.5528464
C	0.7268609	-0.8720269	2.6206283
C	-1.1307318	-1.0059339	0.0552877
C	-2.2759039	-0.7946147	0.9965455
C	-2.8238882	-2.1636461	1.4655792
C	4.4940664	1.8990571	0.1286893
N	4.1246938	2.9974889	0.1849337
C	-3.3025697	0.0475980	0.2254810
C	-4.0137769	-0.4837849	-0.8612590
C	-4.9440730	0.2978429	-1.5450123
C	-5.1728925	1.6183471	-1.1538966
C	-4.4686172	2.1548018	-0.0751712
C	-3.5329841	1.3761437	0.6054924
H	4.8832930	0.0445402	1.0411163
H	5.9464116	0.4381717	-0.3339182
H	1.3999581	-3.0325993	-0.4160145
H	1.9603776	-2.5956665	-2.0377017
H	0.2358950	-2.7819491	-1.7409380
H	0.6921884	0.9848016	-1.9286776
H	-0.2238061	-0.3841471	-2.6020364
H	1.4977679	-0.2082308	-2.9565981
H	-0.6606784	1.4796679	1.9244886
H	0.4367057	1.9981616	0.6216750
H	1.0202669	1.9081088	2.2842976
H	-0.2869782	-0.8830640	3.0242215
H	1.3891926	-0.4467707	3.3776306
H	1.0501230	-1.8973500	2.4251702
H	-1.3985571	-1.4946535	-0.8814885
H	-1.9538280	-0.2286868	1.8701686
H	-3.7073284	-1.9940447	2.0853199
H	-2.0813325	-2.7066053	2.0571323
H	-3.1197436	-2.7953504	0.6242733
H	-3.8648114	-1.5151196	-1.1706388
H	-5.4946368	-0.1269913	-2.3782253
H	-5.8998900	2.2242913	-1.6848507
H	-4.6484601	3.1775931	0.2399347
H	-2.9994002	1.8006737	1.4513818

SCN_Ph_ME_3.log
E = -1262.599846
H = -1262.186931
G = -1262.265738
NImag=0

C	0.9688306	-0.7769366	0.8200676
N	-0.0703994	0.2749117	0.4744486
C	0.5845198	1.3860228	-0.3854946
N	1.9684591	0.9701459	-0.4692900
C	2.1762365	-0.1311393	0.1378777
S	3.7319403	-0.9387551	0.2333950
C	4.7384793	0.2633388	-0.7512966
C	1.1509792	-0.8840077	2.3421803
C	0.5937145	-2.1258766	0.1853375
C	-0.0146193	1.4100421	-1.7962837
C	0.5046979	2.7372163	0.3340234
C	-1.2850599	0.1762289	0.8918910
C	-2.4722103	1.0525751	0.6408043
C	-2.9583847	1.6670164	1.9755008
C	6.1102876	-0.2218410	-0.8146881
N	7.2006445	-0.6147707	-0.8608314
C	-3.5172279	0.1566614	-0.0398250
C	-3.8399953	0.3686103	-1.3864291
C	-4.7964359	-0.4257363	-2.0184286
C	-5.4289676	-1.4519008	-1.3152903
C	-5.1072322	-1.6764393	0.0244711
C	-4.1562245	-0.8786593	0.6594343
H	4.3080158	0.3473788	-1.7518065
H	4.6931986	1.2418109	-0.2676612
H	1.3901762	0.0851356	2.7865367
H	1.9707862	-1.5711491	2.5639024
H	0.2517735	-1.2843843	2.8197357
H	0.4622654	-2.0376434	-0.8956328
H	-0.3285606	-2.5195238	0.6229761
H	1.3868371	-2.8529268	0.3743232
H	0.6030347	2.0706617	-2.4084769
H	-1.0363468	1.7914582	-1.8074020
H	0.0021258	0.4148014	-2.2463631
H	0.9215713	2.6673580	1.3415704
H	1.1039968	3.4543911	-0.2309597
H	-0.5164618	3.1182586	0.3915407
H	-1.4902580	-0.6957679	1.5129170
H	-2.2154070	1.8624608	-0.0416095
H	-2.2067994	2.3382502	2.4005762
H	-3.1924552	0.9006216	2.7185694
H	-3.8691024	2.2395402	1.7853833
H	-3.3634096	1.1726823	-1.9403973
H	-5.0491424	-0.2395190	-3.0573005
H	-6.1724312	-2.0708601	-1.8069689
H	-5.6016065	-2.4679183	0.5785209
H	-3.9352071	-1.0587773	1.7083520

SCN_Ph_ME_4.log
E = -1262.602936
H = -1262.190077
G = -1262.268389
NImag=0

C	-0.9270562	-0.8246595	-0.7321353
N	-0.1087565	-0.5452509	0.5060093
C	-1.0250355	0.0692494	1.6075356
N	-2.3343141	0.0403880	1.0067795
C	-2.2992200	-0.4041317	-0.1841689
S	-3.7141331	-0.5187616	-1.2304759
C	-4.9202120	0.4259067	-0.1996040
C	-0.5052532	0.0851709	-1.8999920
C	-0.9091863	-2.3186238	-1.0995527
C	-0.9984879	-0.8079507	2.8620432
C	-0.5983466	1.5147575	1.8816285
C	1.1468630	-0.7446480	0.7099203
C	2.2178645	-1.2682066	-0.1969420
C	2.7721924	-2.5979224	0.3685972
C	-4.5622824	1.8350662	-0.0828093
N	-4.2509680	2.9490612	0.0079208
C	3.2721834	-0.1561939	-0.2926992
C	4.0603780	0.1981327	0.8127230
C	5.0145130	1.2089667	0.7034853
C	5.1910200	1.8784076	-0.5087892
C	4.4108905	1.5321414	-1.6128323
C	3.4514846	0.5258427	-1.5029405
H	-5.8806724	0.3091897	-0.7085436
H	-4.9771258	-0.0338203	0.7891141
H	0.4905876	-0.1681828	-2.2659614
H	-1.2087105	-0.0448610	-2.7252597
H	-0.5182688	1.1379192	-1.6085149
H	-1.6373703	-2.4997801	-1.8944183
H	0.0662177	-2.6341168	-1.4743005
H	-1.1819947	-2.9413873	-0.2441039
H	-1.7353335	-0.4171179	3.5667102

H	-1.2642428	-1.8413541	2.6268306
H	-0.0217633	-0.7872130	3.3532997
H	-0.6246487	2.1131691	0.9684022
H	0.4035555	1.5613003	2.3174916
H	-1.3028275	1.9538109	2.5910241
H	1.4916274	-0.4694641	1.7060527
H	1.8217440	-1.4521744	-1.1953090
H	3.6031923	-2.9259764	-0.2599842
H	2.0065753	-3.3790145	0.3713469
H	3.1475019	-2.4832865	1.3885984
H	3.9521573	-0.3225274	1.7607889
H	5.6236984	1.4685644	1.5632961
H	5.9360761	2.6628205	-0.5933823
H	4.5500654	2.0415975	-2.5609220
H	2.8593451	0.2555302	-2.3731578

SCN_Ph_ME_5.log
E = -1262.602886
H = -1262.189943
G = -1262.268430
NImag=0

C	-0.8664027	-0.8852656	0.4238786
N	-0.1096013	0.4000085	0.1846940
C	-1.0004054	1.3863667	-0.6296575
N	-2.2219387	0.6482946	-0.8263945
C	-2.1751261	-0.5001741	-0.2823417
S	-3.5209113	-1.6398016	-0.2799974
C	-4.8407456	-0.5391735	-0.9557271
C	-0.1994974	-2.0793523	-0.2819490
C	-1.0918397	-1.1259952	1.9267035
C	-1.2767245	2.6449142	0.1988728
C	-0.3355292	1.6912627	-1.9746878
C	1.0742835	0.7270716	0.5708914
C	2.1121037	-0.0499872	1.3206630
C	2.4205029	0.6569569	2.6624621
C	-5.1770209	0.5467768	-0.0418963
N	-5.4191873	1.4106269	0.6937913
C	3.3274708	-0.1467917	0.3871181
C	4.0693430	0.9909674	0.0352107
C	5.1729501	0.8801103	-0.8096873
C	5.5482023	-0.3667399	-1.3129311
C	4.8161145	-1.5033966	-0.9670379
C	3.7073620	-1.3926109	-0.1283111
H	-4.5095067	-0.1377690	-1.9155728
H	-5.7014624	-1.1940834	-1.1150899
H	-0.0139313	-1.8651657	-1.3370231
H	-0.8638011	-2.9452531	-0.2229292
H	0.7426030	-2.3545720	0.1933869
H	-1.5681376	-0.2633741	2.3988173
H	-0.1558375	-1.3452156	2.4438570
H	-1.7521616	-1.9863001	2.0564679
H	-0.3674447	3.2295578	0.3646703
H	-1.7297234	2.3921346	1.1601011
H	-1.9844343	3.2682555	-0.3513286
H	0.5948054	2.2514384	-1.8469595
H	-1.0203550	2.3023836	-2.5662177
H	-0.1284567	0.7728838	-2.5289478
H	1.3782970	1.7344591	0.2882529
H	1.7603451	-1.0594752	1.5320438
H	3.2302338	0.1208135	3.1626686
H	1.5464036	0.6615000	3.3199817
H	2.7432795	1.6907181	2.5148519
H	3.8073152	1.9684608	0.4317457
H	5.7432912	1.7665155	-1.0681627
H	6.4098971	-0.4523235	-1.9669431
H	5.1090253	-2.4773748	-1.3457423
H	3.1529772	-2.2865633	0.1445847

SCN_Ph_ME_6.log
E = -1262.599510
H = -1262.186599
G = -1262.265291
NImag=0

C	-0.8162026	-0.5552379	0.6567952
N	0.0817985	0.6053349	0.2999718
C	-0.7352268	1.6749363	-0.4833034
N	-2.0696320	1.1286510	-0.5095744
C	-2.1269233	-0.0046007	0.0706112
S	-3.5888583	-0.9654252	0.2292742
C	-4.7661104	0.1585269	-0.6528040
C	-0.3933675	-1.8434307	-0.0719026
C	-0.9181472	-0.7371562	2.1811857
C	-0.7231988	3.0030584	0.2794868
C	-0.1803121	1.8026710	-1.9052766
C	1.3299758	0.7764321	0.5676489
C	2.3172263	-0.1119293	1.2595006
C	2.8049581	0.5694224	2.5611765
C	-6.0801213	-0.4695754	-0.6704100
N	-7.1230908	-0.9770491	-0.6799722
C	3.4396223	-0.3679377	0.2433832
C	3.6037966	-1.6492592	-0.2983425
C	4.6240770	-1.9049575	-1.2142760
C	5.4812083	-0.8776750	-1.6111254
C	5.3200966	0.4043845	-1.0826130
C	4.3049542	0.6596739	-0.1615543
H	-4.7992730	1.1172525	-0.1302148
H	-4.3997952	0.3260136	-1.6682298
H	-0.3034947	-1.6805068	-1.1483372
H	-1.1529552	-2.6114695	0.0914290
H	0.5541685	-2.2283897	0.3071282
H	-1.2052402	0.1952401	2.6733964
H	0.0210471	-1.0919109	2.6093512
H	-1.6840045	-1.4842359	2.4027395
H	-1.3876866	3.6998347	-0.2355442
H	0.2748618	3.4488876	0.3066014
H	-1.0896156	2.8733921	1.3006303
H	-0.1834600	0.8372496	-2.4165760
H	-0.8190027	2.4889495	-2.4652598
H	0.8362159	2.2059340	-1.9064080
H	1.7418777	1.7217192	0.2157019
H	1.8627263	-1.0683859	1.5166297
H	1.9893034	0.6811204	3.2811198
H	3.2311420	1.5574985	2.3706388
H	3.5837414	-0.0514415	3.0098448
H	2.9504438	-2.4597774	0.0135151
H	4.7505717	-2.9062168	-1.6133439
H	6.2743065	-1.0756955	-2.3248230
H	5.9889329	1.2051172	-1.3814038
H	4.2095749	1.6605663	0.2513490

SCN_Ph_MS_1.log
E = -1262.528840
H = -1262.118241
G = -1262.198462
NImag=0

C	-0.6210135	-0.9456148	0.1515546
N	0.1370666	0.3126412	0.1544834
C	-0.7122450	1.4967149	0.4339985
N	-2.0868553	0.9437764	0.4275215
C	-1.9993135	-0.2943117	0.2392056
S	-3.5555013	-1.2856247	0.2717317
C	-4.7922167	0.0182231	0.7629617
C	-0.4187868	-1.7497297	-1.1505984
C	-0.3989095	-1.8357674	1.4014937
C	-0.4556883	2.1099041	1.8208976
C	-0.6348400	2.5579361	-0.6746240
C	1.5228340	0.4349499	0.0670642
C	2.4711426	-0.5236382	0.0790431
C	-3.9418945	-1.5794265	-1.4955442
C	-5.0825037	0.9610287	-0.3023244
N	-5.3247667	1.6711146	-1.1872950
C	3.9098609	-0.2735863	-0.0892111
C	4.4240196	0.8869830	-0.6970240
C	5.7974367	1.0900758	-0.8021736
C	6.6928900	0.1315838	-0.3220062
C	6.1993982	-1.0363028	0.2609629
C	4.8254432	-1.2361111	0.3733689
H	-5.6861886	-0.5340268	1.0673793
H	-4.3475942	0.5154344	1.6286537
H	-0.9587980	-2.7022345	-1.1199990
H	0.6388879	-1.9838255	-1.2764712
H	-0.7316960	-1.1721689	-2.0243858
H	0.6157413	-2.2347338	1.4090473
H	-1.0849475	-2.6898545	1.4064517
H	-0.5406828	-1.2627736	2.3211119
H	-0.5739374	1.3616803	2.6087676
H	-1.1420470	2.9390392	2.0084989
H	0.5675568	2.4907734	1.8658543
H	0.3603347	3.0081204	-0.6987518
H	-1.3612353	3.3517971	-0.4869352
H	-0.8369120	2.1116722	-1.6510009
H	1.8340716	1.4696641	-0.0395642
H	2.2041705	-1.5618662	0.2492326
H	-4.9183571	-2.0669401	-1.5255731
H	-3.1758733	-2.2548615	-1.8755315
H	-3.9502204	-0.6324102	-2.0363268
H	3.7473751	1.6253496	-1.1177484
H	6.1710278	1.9925259	-1.2768380
H	7.7629559	0.2884755	-0.4135387
H	6.8850350	-1.7932447	0.6297237
H	4.4517058	-2.1458202	0.8370181

SCN_Ph_MS_2.log
E = -1262.529829
H = -1262.119397
G = -1262.200589

NImag=0

C	-0.7400110	-0.7171233	-0.3938086
N	0.0821608	0.4731342	-0.1364308
C	-0.7275598	1.6791902	0.1672492
N	-2.1231888	1.2265033	-0.0702892
C	-2.0730545	0.0075021	-0.3596242
S	-3.6670228	-0.8792710	-0.6931827
C	-4.7197735	-0.1144998	0.6360239
C	-0.4866095	-1.3435406	-1.7836513
C	-0.6948770	-1.7865351	0.7255840
C	-0.6259343	2.1236326	1.6361175
C	-0.4420447	2.8400647	-0.7973791
C	1.4713520	0.4988446	-0.0253263
C	2.3556732	-0.5141786	-0.1203889
C	-4.2687213	0.0277314	-2.1633162
C	-4.3155935	-0.6461299	1.9274795
N	-3.9527539	-1.0728734	2.9432098
C	3.8152797	-0.3635007	-0.0323026
C	4.4800128	0.8619534	-0.2262870
C	5.8638236	0.9518667	-0.1046918
C	6.6232238	-0.1815908	0.1957209
C	5.9823761	-1.4087681	0.3688260
C	4.5968516	-1.4973315	0.2541020
H	-4.5578908	0.9665192	0.5841795
H	-5.7562629	-0.3717206	0.3984945
H	0.5210105	-1.7574330	-1.8346498
H	-0.5760077	-0.5919821	-2.5725826
H	-1.1845021	-2.1661409	-1.9779549
H	0.3064037	-2.2114526	0.8003127
H	-1.3848044	-2.6095740	0.5105194
H	-0.9506120	-1.3553882	1.6962907
H	-0.8937424	1.3069994	2.3109060
H	-1.2825602	2.9757776	1.8275831
H	0.4013109	2.4249172	1.8556903
H	-0.5518699	2.5162696	-1.8351276
H	0.5792893	3.2016527	-0.6576955
H	-1.1247365	3.6706769	-0.6035825
H	1.8471851	1.5014906	0.1518824
H	2.0106527	-1.5355250	-0.2466188
H	-5.3102316	-0.2568784	-2.3239379
H	-3.6607356	-0.3036020	-3.0067891
H	-4.1549519	1.0991486	-1.9933025
H	3.9172799	1.7498484	-0.5006424
H	6.3543665	1.9081120	-0.2607124
H	7.7027646	-0.1096406	0.2819048
H	6.5613061	-2.2992860	0.5943061
H	4.1072317	-2.4573515	0.3986811

SCN_Ph_MS_3.log
E = -1262.529665
H = -1262.119178
G = -1262.200076
NImag=0

C	-0.9255563	-0.9138891	-0.5262410
N	0.0780621	0.1218665	-0.2262011
C	-0.5211681	1.4429983	0.0680201
N	-1.9819755	1.2001469	-0.0776405
C	-2.1317283	-0.0070757	-0.3828884
S	-3.8594585	-0.6339258	-0.6308979
C	-4.6697130	0.1229887	0.8638526
C	-0.8090247	-1.4742571	-1.9614960
C	-0.9624116	-2.0628568	0.5063907
C	-0.2803878	1.9038963	1.5149775
C	-0.1489246	2.5215203	-0.9639482
C	1.4329512	-0.2025178	-0.1730962
C	2.4953101	0.5979900	0.0463735
C	-4.4531930	0.4956294	-1.9406806
C	-4.2272328	-0.5822040	2.0557490
N	-3.8359381	-1.1516391	2.9874944
C	3.8903491	0.1372827	0.0999928
C	4.2542614	-1.2028950	0.3295258
C	5.5917944	-1.5884326	0.3456579
C	6.6026219	-0.6441061	0.1505415
C	6.2601142	0.6925399	-0.0561911
C	4.9213450	1.0772156	-0.0789102
H	-4.3666257	1.1742611	0.8883189
H	-5.7493560	0.0270059	0.7150473
H	0.1255179	-2.0315872	-2.0649579
H	-0.8017551	-0.6693561	-2.7010934
H	-1.6235437	-2.1724657	-2.1875635
H	-0.0310523	-2.6325139	0.4590951
H	-1.7760961	-2.7642016	0.2925336
H	-1.0782441	-1.6819363	1.5235015
H	-0.6635073	1.1639790	2.2220479
H	-0.7744127	2.8612514	1.6958148
H	0.7888971	2.0217077	1.6962204
H	-0.6886183	3.4477435	-0.7531783
H	-0.3932126	2.1850134	-1.9746652
H	0.9211322	2.7275020	-0.9276823
H	1.6099409	-1.2591368	-0.3479377
H	2.3618953	1.6676593	0.1706184
H	-5.5370317	0.3846360	-2.0071836
H	-3.9899329	0.1663548	-2.8722712
H	-4.1552050	1.5149997	-1.6917362
H	3.4891890	-1.9489736	0.5257550
H	5.8478359	-2.6279960	0.5277359
H	7.6449160	-0.9461038	0.1719389
H	7.0362137	1.4381760	-0.2002301
H	4.6658063	2.1204908	-0.2471399

SCN_Ph_MS_4.log
E = -1262.529850
H = -1262.119306
G = -1262.199955
NImag=0

C	-0.7442117	-0.8385803	-0.0509265
N	0.0758208	0.3288471	-0.4073630
C	-0.7355245	1.5253746	-0.7427249
N	-2.1329737	1.0249841	-0.6815628
C	-2.0817373	-0.1757213	-0.3238018
S	-3.6811097	-1.0930315	-0.1190315
C	-4.6734060	0.2307452	0.7314422
C	-0.5456350	-2.0484245	-0.9944686
C	-0.6348479	-1.2572551	1.4342627
C	-0.6116044	2.6563451	0.2930292
C	-0.4717775	2.0305270	-2.1688563
C	1.4607280	0.4223645	-0.2773430
C	2.3569107	-0.5403630	0.0177909
C	-4.3645341	-0.9890221	-1.8122547
C	-4.1876732	0.3902053	2.0924424
N	-3.7600560	0.5088243	3.1641576
C	3.8058457	-0.3272802	0.1428075
C	4.3871474	0.9389051	0.3433428
C	5.7686552	1.0860764	0.4287953
C	6.6063455	-0.0280229	0.3343311
C	6.0455256	-1.2932261	0.1579132
C	4.6631738	-1.4396065	0.0653813
H	-4.5361681	1.1489568	0.1521921
H	-5.7162397	-0.0992494	0.7092946
H	-1.2865846	-2.8310342	-0.7907210
H	0.4365694	-2.4971034	-0.8433442
H	-0.6162254	-1.7422089	-2.0414194
H	0.3913471	-1.5488329	1.6614190
H	-0.9043601	-0.4338876	2.1001068
H	-1.2792988	-2.1155495	1.6505296
H	-0.8466586	2.2935095	1.2965427
H	-1.2861915	3.4784776	0.0424056
H	0.4094625	3.0447032	0.2988986
H	0.5595470	2.3823235	-2.2507025
H	-1.1378790	2.8619436	-2.4114511
H	-0.6157413	1.2275772	-2.8958057
H	1.8223402	1.4279845	-0.4660247
H	2.0343177	-1.5677876	0.1539830
H	-4.2531170	0.0318371	-2.1799931
H	-5.4098584	-1.3001769	-1.7666941
H	-3.7959568	-1.6917907	-2.4235040
H	3.7563790	1.8158475	0.4598161
H	6.1937295	2.0730277	0.5855710
H	7.6827583	0.0890738	0.4094182
H	6.6846312	-2.1685599	0.0916806
H	4.2372550	-2.4295891	-0.0788399

SCN_Ph_MS_5.log
E = -1262.530297
H = -1262.119757
G = -1262.200036
NImag=0

C	0.7076173	-1.0288255	-0.2370174
N	-0.2799386	0.0079980	0.1067398
C	0.3393484	1.3273441	0.3673350
N	1.7979255	1.0367362	0.3284933
C	1.9330858	-0.1730913	0.0232656
S	3.6424915	-0.8632406	-0.1283990
C	4.4876192	0.6130551	-0.8878795
C	0.6547297	-2.2490511	0.7080594
C	0.6508876	-1.4835330	-1.7141152
C	0.0722941	2.3686322	-0.7350863
C	0.0151648	1.8634817	1.7700905
C	-1.6454330	-0.2557880	-0.0075145
C	-2.6917050	0.5839826	0.1225374
C	4.2274697	-0.7709516	1.6042370
C	5.9238565	0.4999754	-0.7040672
N	7.0626977	0.3818619	-0.5153884
C	-4.1030430	0.1851077	0.0218936
C	-5.0702336	1.1780859	-0.2167648
C	-6.4197887	0.8557847	-0.3418929
C	-6.8373992	-0.4704242	-0.2270782

C	-5.8920689	-1.4675264	0.0257614
C	-4.5444555	-1.1445495	0.1571819
H	4.2141407	0.5992576	-1.9468418
H	4.0644599	1.4994526	-0.4043812
H	1.4679455	-2.9533406	0.4964416
H	-0.2790556	-2.7969227	0.5599311
H	0.6999665	-1.9385670	1.7549826
H	-0.2947546	-1.9979757	-1.9029802
H	1.4554000	-2.1891438	-1.9487918
H	0.7096487	-0.6300284	-2.3941976
H	0.3734818	1.9822942	-1.7121786
H	0.6262613	3.2872657	-0.5282894
H	-0.9905370	2.6072491	-0.7813797
H	-1.0571009	2.0392105	1.8662125
H	0.5416015	2.8039655	1.9478646
H	0.3076200	1.1374058	2.5324276
H	-1.8447069	-1.3014962	-0.2199182
H	-2.5284119	1.6432458	0.2921291
H	5.3089035	-0.9190954	1.5854306
H	3.7439455	-1.5881585	2.1414995
H	3.9544581	0.1986349	2.0226492
H	-4.7548929	2.2140005	-0.3143281
H	-7.1451565	1.6417080	-0.5292643
H	-7.8883817	-0.7245251	-0.3211721
H	-6.2091559	-2.5002511	0.1367317
H	-3.8355612	-1.9331788	0.3939203

SCN_Ph_MS_6.log
E = -1262.530479
H = -1262.119951
G = -1262.200756
NImag=0

C	0.5499866	-0.7747507	-0.0705462
N	-0.2811096	0.4090284	0.1869794
C	0.5161628	1.6456583	0.3776626
N	1.9201393	1.1684578	0.3004067
C	1.8848195	-0.0654475	0.0748354
S	3.4850318	-0.9817617	-0.0826691
C	4.4578016	0.2962745	-1.0267265
C	0.4108866	-1.8813333	1.0014115
C	0.3943692	-1.3504329	-1.4980719
C	0.3179264	2.6780427	-0.7455707
C	0.3042177	2.2732145	1.7638056
C	-1.6726818	0.4620264	0.1134999
C	-2.5578121	-0.5416856	-0.0484815
C	4.1759902	-0.7957117	1.6025052
C	5.8774924	0.0137177	-0.9075678
N	7.0023750	-0.2342978	-0.7674934
C	-4.0155720	-0.3694862	-0.1275886
C	-4.6341147	0.8576502	-0.4318867
C	-6.0211168	0.9697936	-0.4679538
C	-6.8277953	-0.1433565	-0.2191785
C	-6.2307018	-1.3726026	0.0616904
C	-4.8426154	-1.4831968	0.1045869
H	4.1172892	0.2220612	-2.0635159
H	4.1819941	1.2707687	-0.6110491
H	0.5258793	-1.4667059	2.0061314
H	1.1518129	-2.6748261	0.8459492
H	-0.5717016	-2.3503326	0.9448932
H	0.5895476	-0.5842363	-2.2530934
H	-0.6253966	-1.7075425	-1.6465252
H	1.0690295	-2.1977118	-1.6594142
H	0.4980644	2.2281241	-1.7250491
H	0.9954678	3.5240390	-0.6082803
H	-0.7062365	3.0575998	-0.7276192
H	0.5026912	1.5441360	2.5530099
H	-0.7318640	2.6064512	1.8616479
H	0.9576691	3.1384271	1.8986464
H	-2.0491433	1.4740946	0.2218678
H	-2.2181684	-1.5714348	-0.1006803
H	3.6289338	-1.4891722	2.2429251
H	4.0458921	0.2368463	1.9287708
H	5.2287712	-1.0799392	1.5506800
H	-4.0297349	1.7287405	-0.6690139
H	-6.4753463	1.9268270	-0.7068294
H	-7.9088772	-0.0545616	-0.2559039
H	-6.8459243	-2.2476225	0.2483596
H	-4.3881647	-2.4445986	0.3313957

SCN_Ph_MN2_1.log
E = -1262.567328
H = -1262.153641
G = -1262.228267
NImag=0

C	1.6139362	-0.8434709	-1.1186612
N	0.6652723	-1.4205441	0.0177319
C	1.4646313	-0.9801931	1.4053524
N	2.1906243	0.1823957	0.9711389
C	2.2518943	0.2827040	-0.2969491
S	2.9765751	1.6730861	-1.1149802
C	2.8929589	2.8815552	0.2832196
C	2.6878343	-1.8446345	-1.5845369
C	0.8145626	-0.3823209	-2.3436763
C	0.4671947	-0.6319327	2.5108847
C	2.4183735	-2.0730645	1.9003753
C	-0.5996574	-0.6565484	-0.0111072
C	-1.8317848	-1.1680907	-0.1182264
C	0.4397140	-2.8916814	-0.0879117
C	1.5073395	3.0749095	0.7020298
N	0.3888850	3.1569636	1.0018287
C	-3.0759830	-0.3910161	-0.1120532
C	-4.2781663	-1.0729800	-0.3713986
C	-5.4944908	-0.3948028	-0.3870723
C	-5.5277772	0.9772395	-0.1379520
C	-4.3402856	1.6668997	0.1289767
C	-3.1240374	0.9938302	0.1439674
H	3.4970857	2.5233144	1.1186138
H	3.3209924	3.8086796	-0.1066374
H	3.2893254	-2.2588625	-0.7772634
H	3.3711768	-1.3095349	-2.2492643
H	2.2471208	-2.6586467	-2.1652477
H	0.1532844	0.4589923	-2.1443706
H	0.2224333	-1.2072179	-2.7491811
H	1.5208217	-0.0745415	-3.1187659
H	-0.1017365	0.2719792	2.3002653
H	1.0460360	-0.4527901	3.4201949
H	-0.2197471	-1.4591336	2.7056277
H	3.1370782	-2.4044538	1.1524651
H	1.8741750	-2.9338816	2.2948269
H	2.9904381	-1.6380821	2.7230265
H	-0.4226417	0.4058939	0.0828212
H	-1.9729044	-2.2394368	-0.2258947
H	1.3881638	-3.4158006	-0.0436967
H	-0.0584290	-3.1135585	-1.0319520
H	-0.1908848	-3.2100497	0.7415323
H	-4.2563635	-2.1423739	-0.5650366
H	-6.4126013	-0.9362851	-0.5906042
H	-6.4738838	1.5090447	-0.1457863
H	-4.3648530	2.7327660	0.3316085
H	-2.2205721	1.5533520	0.3685046

SCN_Ph_MN2_2.log
E = -1262.565934
H = -1262.151802
G = -1262.227616
NImag=0

C	1.0153463	-1.1494088	0.2458476
N	0.1587895	0.1926824	0.2471370
C	0.9778355	1.1201112	-0.8910462
N	2.2956136	0.5618090	-0.8405275
C	2.3414456	-0.5642163	-0.2480562
S	3.8391184	-1.4542359	0.0260944
C	5.0484198	-0.1453700	-0.4617404
C	1.0755918	-1.8102631	1.6290823
C	0.4880447	-2.1944775	-0.7564769
C	0.3566846	0.9703334	-2.2829003
C	0.9909193	2.5972118	-0.4984732
C	-1.2485387	-0.0267877	-0.1004937
C	-2.3047874	0.3200334	0.6517282
C	0.3086083	0.8669635	1.5832153
C	4.9710368	1.0101666	0.4261458
N	4.8634649	1.9170179	1.1422013
C	-3.7145136	0.1162469	0.3071198
C	-4.1475081	-0.4638258	-0.9011367
C	-5.5030380	-0.6289840	-1.1577547
C	-6.4544155	-0.2194059	-0.2169327
C	-6.0413841	0.3579026	0.9835214
C	-4.6829410	0.5244024	1.2420577
H	6.0292950	-0.6236538	-0.3974170
H	4.8588685	0.1552925	-1.4940088
H	1.6156238	-1.2284132	2.3750931
H	1.5953436	-2.7661093	1.5283418
H	0.0674642	-2.0313447	1.9915776
H	1.2081934	-3.0158843	-0.7858922
H	0.3903335	-1.8209069	-1.7745788
H	-0.4687237	-2.6050898	-0.4262869
H	1.0053535	1.5106651	-2.9759616
H	-0.6357312	1.4232932	-2.3257208
H	0.3051865	-0.0602823	-2.6313475
H	1.5839469	2.7968622	0.3932045
H	-0.0222292	2.9916428	-0.3840718
H	1.4642337	3.1374560	-1.3215337
H	-1.3686749	-0.5028909	-1.0595412
H	-2.1520874	0.7975458	1.6142497
H	1.3661348	1.0158145	1.7913439
H	-0.1384220	0.2464001	2.3546850
H	-0.1968187	1.8272633	1.5547167

H	-3.4294022	-0.7877105	-1.6485345
H	-5.8231826	-1.0769930	-2.0929504
H	-7.5118761	-0.3505251	-0.4228314
H	-6.7740654	0.6784441	1.7168724
H	-4.3656409	0.9749574	2.1788618

SCN_Ph_MN2_3.log
E = -1262.565870
H = -1262.151719
G = -1262.227347
NImag=0

C	1.0537665	-0.9465666	-0.6774735
N	0.0977769	-0.5439798	0.5301375
C	1.0013543	0.6600164	1.2894257
N	2.3372447	0.3155916	0.9101294
C	2.3850775	-0.4772536	-0.0850906
S	3.8876108	-0.9844532	-0.8545132
C	5.0521304	0.2023601	-0.0493573
C	1.0039098	-2.4456387	-1.0046963
C	0.7368529	-0.1680082	-1.9692155
C	0.6272079	2.0505585	0.7677433
C	0.8135653	0.6187179	2.8042891
C	-1.2171283	-0.0683035	0.0882849
C	-2.4011699	-0.5909089	0.4439120
C	-0.0187459	-1.7025077	1.4776899
C	4.7650105	1.5867789	-0.4072982
N	4.5047821	2.6810159	-0.6922600
C	-3.7181978	-0.1082753	0.0185718
C	-3.9083154	1.0239073	-0.7974595
C	-5.1880602	1.4208428	-1.1654134
C	-6.3039717	0.6989921	-0.7277998
C	-6.1320671	-0.4229188	0.0826442
C	-4.8499175	-0.8216376	0.4529490
H	4.9977917	0.0730649	1.0335480
H	6.0450074	-0.0931741	-0.3987677
H	1.3786579	-3.0874760	-0.2076807
H	1.6299969	-2.6199340	-1.8834776
H	-0.0115013	-2.7503403	-1.2739401
H	0.7485132	0.9138827	-1.8463305
H	-0.2258712	-0.4769220	-2.3824790
H	1.5100280	-0.4130554	-2.7003510
H	1.3439248	2.7504161	1.2033000
H	-0.3750887	2.3423150	1.0878935
H	0.7149892	2.1482960	-0.3132053
H	1.2220877	-0.2801767	3.2648724
H	-0.2370219	0.7408331	3.0803211
H	1.3632501	1.4692348	3.2139316
H	-1.1491143	0.7827170	-0.5685042
H	-2.4398687	-1.4530270	1.1024681
H	0.9770723	-2.0082579	1.7904766
H	-0.5201471	-2.5292885	0.9820175
H	-0.5977561	-1.3968871	2.3438637
H	-3.0594098	1.6043365	-1.1460139
H	-5.3202080	2.2959456	-1.7935217
H	-7.3012265	1.0138710	-1.0178681
H	-6.9933749	-0.9861179	0.4266493
H	-4.7210308	-1.6966902	1.0846233

SEt_1.log
E = -1169.983877
H = -1169.584149
G = -1169.658082
NImag=0

C	1.0076380	-0.9178810	0.1290570
N	0.2164920	0.3217570	0.0554840
C	1.0913820	1.5262000	0.0435450
N	2.4501300	0.9894130	0.1226380
C	2.4020530	-0.2829740	0.1736270
S	3.8270590	-1.3234690	0.2976080
C	5.1761630	-0.0703170	0.2898020
C	5.5247950	0.4595950	-1.0976440
C	0.9485390	2.3238700	-1.2638920
C	0.8482750	2.4263840	1.2674600
C	0.7285510	-1.7224340	1.4173120
C	0.8586430	-1.8014970	-1.1298640
C	-1.1513690	0.4270860	0.0176600
C	-2.1015660	-0.5398080	-0.0153530
C	-3.5451520	-0.2941610	-0.0363760
C	-4.1384150	0.9516270	0.2575040
C	-5.5183310	1.1251830	0.2048130
C	-6.3593350	0.0595240	-0.1260770
C	-5.7940310	-1.1863050	-0.4017920
C	-4.4127500	-1.3580690	-0.3570240
H	4.8593880	0.7360990	0.9542620
H	6.0222910	-0.5892110	0.7492300
H	4.6703540	0.9819430	-1.5325390
H	5.8249620	-0.3481720	-1.7708890
H	6.3563340	1.1698120	-1.0231480
H	1.1401950	1.6774920	-2.1241500
H	1.6667100	3.1480850	-1.2783440
H	-0.0577680	2.7421400	-1.3619340
H	0.9663370	1.8517610	2.1896210
H	-0.1582680	2.8549660	1.2509140
H	1.5699600	3.2474640	1.2725150
H	-0.3082070	-2.0620950	1.4486300
H	1.3785280	-2.6003340	1.4690300
H	0.9114840	-1.1052300	2.3007480
H	1.0983450	-1.2295140	-2.0299910
H	-0.1604490	-2.1791950	-1.2283770
H	1.5358090	-2.6584390	-1.0753090
H	-1.4793010	1.4628240	0.0073850
H	-1.8141250	-1.5844790	-0.0559730
H	-3.5165750	1.7924420	0.5511640
H	-5.9422970	2.0985600	0.4373460
H	-7.4358890	0.1971200	-0.1591350
H	-6.4309500	-2.0296470	-0.6550710
H	-3.9867080	-2.3332120	-0.5811120

SEt_2.log
E = -1169.984072
H = -1169.584300
G = -1169.657879
NImag=0

C	1.1902608	-1.1349890	0.0261485
N	0.2127470	-0.0339041	-0.0699134
C	0.8870165	1.2778139	-0.2521312
N	2.3159955	0.9475699	-0.2675520
C	2.4684449	-0.3088938	-0.1248064
S	4.0378324	-1.1250142	-0.1069027
C	5.1771964	0.3081732	-0.3027344
C	5.4316647	1.0792877	0.9888091
C	0.5350969	1.9315127	-1.5998809
C	0.6362051	2.2325838	0.9286542
C	1.1356583	-1.8490309	1.3929825
C	1.0387770	-2.1546416	-1.1222879
C	-1.1374504	-0.2720931	-0.0019434
C	-2.1782138	0.5951348	-0.0586404
C	-3.5912650	0.2174453	0.0135482
C	-4.0620866	-1.1103426	-0.0642268
C	-5.4199925	-1.4021485	0.0224927
C	-6.3613177	-0.3809597	0.1765995
C	-5.9183200	0.9407259	0.2405598
C	-4.5588339	1.2323181	0.1602790
H	6.0959310	-0.1342439	-0.6981342
H	4.7414401	0.9517243	-1.0695759
H	5.8544683	0.4338062	1.7636968
H	4.5033034	1.5162192	1.3621226
H	6.1393703	1.8944015	0.7987553
H	0.7759604	1.2500180	-2.4197481
H	1.1207185	2.8459499	-1.7251673
H	-0.5254676	2.1825868	-1.6608082
H	0.9371578	1.7558949	1.8651399
H	-0.4162185	2.5113113	1.0070959
H	1.2299485	3.1405635	0.7945590
H	1.2673004	-1.1347286	2.2096563
H	1.9175825	-2.6101656	1.4666593
H	0.1694838	-2.3460234	1.5241376
H	0.0725399	-2.6641501	-1.0560313
H	1.8222856	-2.9160156	-1.0722998
H	1.0993415	-1.6566641	-2.0932939
H	-1.3622188	-1.3279823	0.1222020
H	-1.9937880	1.6600322	-0.1453390
H	-3.3618654	-1.9277956	-0.2104104
H	-5.7477005	-2.4364744	-0.0413753
H	-7.4203572	-0.6124718	0.2382676
H	-6.6339832	1.7505323	0.3549119
H	-4.2288662	2.2669733	0.2171302

SEt_3.log
E = -1169.983987
H = -1169.584346
G = -1169.658428
NImag=0

C	1.1879130	-1.1240580	-0.1670310
N	0.2120090	-0.0185420	-0.1181820
C	0.8884220	1.3046080	-0.1100910
N	2.3176570	0.9769860	-0.1418950
C	2.4682000	-0.2873880	-0.1780300
S	4.0381600	-1.0997480	-0.2468480
C	5.1792120	0.3454830	-0.2023020
C	5.3952080	0.9213510	1.1936490
C	0.5694500	2.1339280	-1.3669660
C	0.6058010	2.0909060	1.1814450
C	1.1174630	-2.0268180	1.0826870
C	1.0494500	-1.9691150	-1.4507390
C	-1.1376840	-0.2604190	-0.0556420

C	-2.1786370	0.6084990	-0.0419510
C	-3.5875990	0.2194430	0.0517250
C	-4.0266910	-1.0627780	0.4432770
C	-5.3813550	-1.3777640	0.4969820
C	-6.3490510	-0.4209660	0.1801400
C	-5.9364530	0.8600030	-0.1896760
C	-4.5808080	1.1726960	-0.2519760
H	4.7637350	1.0936870	-0.8803180
H	6.1096460	-0.0371210	-0.6313620
H	6.1072230	1.7531360	1.1437040
H	5.7960570	0.1694430	1.8791000
H	4.4556580	1.3025460	1.5985680
H	-0.4849850	2.4125570	-1.4120980
H	0.8133830	1.5603850	-2.2648410
H	1.1722320	3.0458840	-1.3636690
H	0.9060190	1.5001540	2.0507750
H	-0.4537980	2.3335670	1.2803010
H	1.1810610	3.0203550	1.1772370
H	1.2323840	-1.4363260	1.9951660
H	1.9047300	-2.7854230	1.0579560
H	0.1549990	-2.5456690	1.1266800
H	1.1247380	-1.3385300	-2.3401850
H	0.0792910	-2.4753750	-1.4696730
H	1.8287200	-2.7351060	-1.5001660
H	-1.3654950	-1.3225460	-0.0211050
H	-2.0006040	1.6743820	-0.1307920
H	-3.3017070	-1.8189140	0.7308870
H	-5.6848920	-2.3755220	0.8029720
H	-7.4052050	-0.6679610	0.2304700
H	-6.6734500	1.6206240	-0.4332660
H	-4.2750020	2.1729920	-0.5492140

SEt_4.log
E = -1169.983929
H = -1169.584165
G = -1169.657878
NImag=0

C	1.0095410	-0.9334560	-0.0449590
N	0.2171110	0.3026090	-0.1558500
C	1.0914000	1.4986880	-0.3050300
N	2.4514660	0.9602140	-0.2756090
C	2.4047040	-0.3062520	-0.1416440
S	3.8332830	-1.3474120	-0.0770010
C	5.1806310	-0.0975930	-0.1949980
C	5.4817130	0.6141430	1.1202260
C	0.8671000	2.2055080	-1.6524990
C	0.9277150	2.4825890	0.8664440
C	0.8205950	-1.6396070	1.3153290
C	0.7704090	-1.9057280	-1.2217490
C	-1.1495020	0.4149300	-0.0983510
C	-2.1025330	-0.5436210	0.0099610
C	-3.5425190	-0.2857230	0.0806920
C	-4.1056730	0.9850240	0.3228400
C	-5.4846740	1.1683320	0.3637880
C	-6.3535360	0.0892920	0.1813880
C	-5.8168840	-1.1796390	-0.0409830
C	-4.4370540	-1.3618500	-0.0898980
H	6.0419680	-0.6687830	-0.5528330
H	4.8851590	0.6110800	-0.9712380
H	5.7644390	-0.0950340	1.9031570
H	4.6104620	1.1802860	1.4555670
H	6.3110720	1.3166880	0.9789650
H	1.0119170	1.5029970	-2.4770140
H	1.5780900	3.0283820	-1.7650100
H	-0.1459190	2.6137980	-1.7180560
H	1.0991100	1.9721240	1.8175230
H	-0.0755780	2.9185670	0.8820690
H	1.6522860	3.2953330	0.7696880
H	1.4728700	-2.5144320	1.3852850
H	-0.2115480	-1.9701640	1.4434770
H	1.0652300	-0.9602770	2.1360340
H	-0.2561730	-2.2760260	-1.2243010
H	1.4417910	-2.7658660	-1.1479180
H	0.9547380	-1.4051600	-2.1758310
H	-1.4755430	1.4493770	-0.1606360
H	-1.8232710	-1.5912540	0.0247150
H	-3.4592010	1.8396010	0.5013520
H	-5.8849970	2.1609390	0.5528700
H	-7.4287910	0.2350990	0.2209660
H	-6.4753080	-2.0331150	-0.1791450
H	-4.0339830	-2.3555480	-0.2707890

SEt_5.log
E = -1169.983268
H = -1169.583563
G = -1169.658046
NImag=0

C	0.9816440	-0.8209000	0.0343820
N	0.1442520	0.3873530	-0.0454860
C	0.9744730	1.6203210	-0.1372800
N	2.3540280	1.1354750	-0.0899860
C	2.3522850	-0.1358110	0.0034180
S	3.8219170	-1.1126450	0.0910060
C	5.1010220	0.2092910	0.0150910
C	6.4915860	-0.4155830	0.0887600
C	0.7541010	2.3598610	-1.4677540
C	0.7444400	2.5593370	1.0595950
C	0.7820020	-1.5927020	1.3566940
C	0.8164060	-1.7496110	-1.1897620
C	-1.2267300	0.4440980	-0.0325290
C	-2.1430750	-0.5551390	0.0002700
C	-3.5937820	-0.3565620	0.0300660
C	-4.2149740	0.8732170	0.3333270
C	-5.6006810	1.0035590	0.3281080
C	-6.4189820	-0.0912770	0.0382380
C	-5.8250590	-1.3222360	-0.2442830
C	-4.4383550	-1.4507110	-0.2475070
H	4.9509300	0.7640890	-0.9133480
H	4.9166990	0.8959960	0.8437990
H	6.6714160	-1.0999400	-0.7457480
H	6.6362640	-0.9690770	1.0213670
H	7.2492090	0.3731400	0.0452820
H	0.9434660	1.6888970	-2.3094010
H	1.4364110	3.2111410	-1.5388750
H	-0.2717950	2.7324090	-1.5448090
H	0.9155200	2.0257910	1.9979430
H	-0.2759260	2.9542230	1.0647800
H	1.4376400	3.4027990	1.0055330
H	1.4640010	-2.4460350	1.4086760
H	-0.2402030	-1.9659880	1.4402820
H	0.9780760	-0.9416360	2.2125760
H	-0.1904590	-2.1681580	-1.2354240
H	0.9987190	-1.1985190	-2.1160080
H	1.5273220	-2.5789600	-1.1366000
H	-1.5920270	1.4670080	-0.0613690
H	-1.8221150	-1.5905760	-0.0237410
H	-3.6093400	1.7352150	0.5979470
H	-6.0464650	1.9657010	0.5665350
H	-7.4998370	0.0124230	0.0424330
H	-6.4439030	-2.1877420	-0.4656330
H	-3.9901660	-2.4145850	-0.4770020

SEt_6.log
E = -1169.983350
H = -1169.583715
G = -1169.659199
NImag=0

C	-1.1486480	-1.0361030	-0.0885080
N	-0.1435060	0.0415320	-0.0160020
C	-0.7855170	1.3779530	0.0884160
N	-2.2232980	1.0887240	0.0800650
C	-2.4060040	-0.1686660	-0.0151220
S	-3.9995260	-0.9305070	-0.0642650
C	-5.0788120	0.5562960	0.0556590
C	-6.5447950	0.1331060	0.0193780
C	-0.4478830	2.0820540	1.4139470
C	-0.4791960	2.2702400	-1.1277380
C	-1.0740070	-1.8170530	-1.4171130
C	-1.0607050	-2.0026120	1.1115100
C	1.2005560	-0.2361420	-0.0224160
C	2.2642940	0.6040570	0.0160310
C	3.6655480	0.1779150	0.0196580
C	4.0883160	-1.1456690	0.2645220
C	5.4363660	-1.4906720	0.2372290
C	6.4146510	-0.5262160	-0.0181400
C	6.0192820	0.7930910	-0.2435070
C	4.6698610	1.1368190	-0.2242330
H	-4.8259420	1.0752580	0.9824800
H	-4.8235920	1.2186230	-0.7740800
H	-6.7929630	-0.3828080	-0.9129590
H	-7.1825780	1.0195610	0.0927230
H	-6.7945620	-0.5297130	0.8532740
H	-0.7315270	1.4462960	2.2566140
H	-1.0070430	3.0187610	1.4817990
H	0.6183750	2.3023180	1.4922330
H	-0.7728240	1.7599930	-2.0486680
H	0.5824280	2.5155820	-1.1930360
H	-1.0493190	3.1996580	-1.0496220
H	-0.1180320	-2.3440240	-1.4954920
H	-1.8733420	-2.5612130	-1.4778020
H	-1.1634330	-1.1401710	-2.2705180
H	-1.8673640	-2.7403660	1.0762770
H	-1.1306420	-1.4561010	2.0554320
H	-0.1106570	-2.5456800	1.0973260
H	1.4002600	-1.3031580	-0.0749690
H	2.1112270	1.6775070	0.0202160
H	3.3569930	-1.9138570	0.4990930

H	5.7270230	-2.5200010	0.4303830
H	7.4658460	-0.7979130	-0.0316930
H	6.7647150	1.5597320	-0.4375300
H	4.3771380	2.1676530	-0.4091100

SEt_7.log
E = -1169.983987
H = -1169.584346
G = -1169.658428
NImag=0

C	1.1414168	-1.0320779	0.1080002
N	0.1353085	0.0439753	0.0255512
C	0.7755383	1.3798909	-0.0917349
N	2.2132138	1.0897997	-0.0925634
C	2.3985541	-0.1655945	0.0180908
S	3.9910385	-0.9334746	0.0796811
C	5.0908983	0.5331687	-0.0990404
C	5.2415287	1.0230344	-1.5358076
C	0.4261745	2.0742926	-1.4191231
C	0.4796637	2.2804381	1.1209982
C	1.0714064	-1.7958300	1.4470234
C	1.0504591	-2.0148816	-1.0784035
C	-1.2088396	-0.2341304	0.0289051
C	-2.2723799	0.6065688	-0.0010516
C	-3.6729312	0.1781923	-0.0171928
C	-4.0914312	-1.1368715	-0.3098893
C	-5.4382844	-1.4874609	-0.2928968
C	-6.4191816	-0.5360181	-0.0015465
C	-6.0278912	0.7757525	0.2705428
C	-4.6796899	1.1246323	0.2622305
H	4.6801171	1.3131677	0.5451615
H	6.0459073	0.2016915	0.3184094
H	5.6375304	0.2385078	-2.1866444
H	5.9324799	1.8733524	-1.5653529
H	4.2783114	1.3544406	-1.9287537
H	0.7107917	1.4362481	-2.2598042
H	0.9762749	3.0157757	-1.4953842
H	-0.6423612	2.2842855	-1.4935674
H	0.7706693	1.7720000	2.0437337
H	-0.5795312	2.5357075	1.1876757
H	1.0574540	3.2046572	1.0373345
H	0.1163605	-2.3232248	1.5342449
H	1.8720568	-2.5379013	1.5158422
H	1.1615844	-1.1076352	2.2912241
H	1.8580239	-2.7509766	-1.0349822
H	1.1169739	-1.4817576	-2.0301708
H	0.1011934	-2.5587094	-1.0534246
H	-1.4093835	-1.3014941	0.0703816
H	-2.1197249	1.6799693	0.0132452
H	-3.3573035	-1.8921387	-0.5757681
H	-5.7258827	-2.5099327	-0.5233821
H	-7.4695101	-0.8113125	0.0033556
H	-6.7757104	1.5323667	0.4926848
H	-4.3902055	2.1491269	0.4837430

SEt_8.log
E = -1169.983350
H = -1169.583715
G = -1169.659197
NImag=0

C	-1.1959602	-1.1178802	0.1410059
N	-0.2170234	-0.0146477	0.1032525
C	-0.8904921	1.3103203	0.1040970
N	-2.3206702	0.9876378	0.1415662
C	-2.4732433	-0.2771574	0.1636070
S	-4.0475926	-1.0775799	0.2172294
C	-5.1617075	0.3881911	0.2264211
C	-6.6162608	-0.0705482	0.2842768
C	-0.5616861	2.1323786	1.3631233
C	-0.6135431	2.1029610	-1.1850147
C	-1.1286954	-2.0057967	-1.1195478
C	-1.0592288	-1.9777603	1.4148022
C	1.1320645	-0.2607253	0.0480090
C	2.1757639	0.6048485	0.0274071
C	3.5848089	0.2125849	-0.0490570
C	4.0269605	-1.0830246	-0.3903259
C	5.3821791	-1.3974902	-0.4291011
C	6.3479310	-0.4281607	-0.1459835
C	5.9325786	0.8650301	0.1746657
C	4.5762921	1.1776946	0.2214046
H	-4.9518526	0.9693887	-0.6738187
H	-4.8923197	1.0035710	1.0872461
H	-7.2748769	0.8036149	0.2877311
H	-6.8212937	-0.6489369	1.1900558
H	-6.8803331	-0.6860331	-0.5809047
H	0.4960983	2.3979229	1.4087353
H	-0.8126551	1.5592831	2.2593702
H	-1.1537771	3.0512844	1.3622925
H	-0.9123399	1.5142367	-2.0561365
H	0.4444819	2.3524906	-1.2843519
H	-1.1944791	3.0287620	-1.1755084
H	-1.2425547	-1.4035855	-2.0244967
H	-1.9178120	-2.7629487	-1.1041722
H	-0.1673943	-2.5263673	-1.1707347
H	-1.1337059	-1.3573761	2.3114866
H	-0.0901450	-2.4861103	1.4284641
H	-1.8400172	-2.7427132	1.4549274
H	1.3560842	-1.3239924	0.0268853
H	1.9998679	1.6726251	0.0956521
H	3.3045119	-1.8520404	-0.6484976
H	5.6878480	-2.4057845	-0.6959668
H	7.4045077	-0.6755600	-0.1840985
H	6.6677678	1.6353502	0.3918260
H	4.2682431	2.1879563	0.4801763

SEt_9.log
E = -1169.984072
H = -1169.584300
G = -1169.657879
NImag=0

C	-1.1968530	-1.1141356	0.1249180
N	-0.2140093	-0.0141289	0.0909958
C	-0.8823421	1.3131330	0.1033758
N	-2.3133280	0.9938766	0.1438095
C	-2.4717954	-0.2698998	0.1525213
S	-4.0467482	-1.0749596	0.1799556
C	-5.1785542	0.3768290	0.2229808
C	-5.3399460	0.9967006	1.6075992
C	-0.5443968	2.1255900	1.3660922
C	-0.6097115	2.1146903	-1.1814501
C	-1.1320890	-1.9967359	-1.1393476
C	-1.0633502	-1.9798258	1.3952842
C	1.1347201	-0.2651928	0.0432086
C	2.1803212	0.5976805	0.0176135
C	3.5903164	0.2072351	-0.0459459
C	4.0387248	-1.1053014	-0.3046357
C	5.3956878	-1.4118143	-0.3404321
C	6.3575155	-0.4195686	-0.1330570
C	5.9359077	0.8883451	0.1094281
C	4.5777797	1.1939182	0.1516861
H	-6.1261952	-0.0130086	-0.1593151
H	-4.7850694	1.1015578	-0.4925791
H	-6.0482858	1.8316677	1.5579671
H	-4.3841864	1.3835661	1.9667454
H	-5.7200087	0.2690710	2.3300723
H	0.5142541	2.3883351	1.4068516
H	-0.7904089	1.5465871	2.2599847
H	-1.1342720	3.0458654	1.3750562
H	-0.9111630	1.5324613	-2.0559606
H	0.4477658	2.3658317	-1.2825293
H	-1.1908334	3.0403370	-1.1635509
H	-1.2487717	-1.3908578	-2.0414560
H	-1.9201106	-2.7549548	-1.1251653
H	-0.1699591	-2.5152493	-1.1945963
H	-1.1334077	-1.3627954	2.2946611
H	-0.0969556	-2.4933118	1.4058011
H	-1.8482636	-2.7405215	1.4328735
H	1.3539420	-1.3294500	0.0316505
H	2.0038730	1.6664119	0.0646860
H	3.3205092	-1.8972425	-0.4967405
H	5.7058092	-2.4336915	-0.5426532
H	7.4153878	-0.6618435	-0.1673348
H	6.6673914	1.6762479	0.2681005
H	4.2649562	2.2169348	0.3471356

SEt_10.log
E = -1169.983877
H = -1169.584149
G = -1169.658082
NImag=0

C	-1.0063835	-0.9257729	0.0099011
N	-0.2147548	0.3130629	0.0913763
C	-1.0890379	1.5096097	0.2333700
N	-2.4478866	0.9674573	0.2472744
C	-2.4003621	-0.3012181	0.1373473
S	-3.8256312	-1.3487040	0.1324362
C	-5.1740932	-0.1042453	0.2857536
C	-5.5255566	0.5964242	-1.0230210
C	-0.8427165	2.2482645	1.5604395
C	-0.9487075	2.4657012	-0.9634046
C	-0.8606750	-1.6436698	-1.3507059
C	-0.7248305	-1.8863760	1.1858114
C	1.1530737	0.4219943	0.0640014
C	2.1026876	-0.5332486	-0.0928168
C	3.5463508	-0.2872535	-0.0860072
C	4.1409232	0.9113977	0.3612452

C	5.5208083	1.0898869	0.3276942
C	6.3605099	0.0742567	-0.1368476
C	5.7939372	-1.1267324	-0.5661483
C	4.4126710	-1.3024449	-0.5402281
H	-6.0194609	-0.6767156	0.6780409
H	-4.8554076	0.6118995	1.0458435
H	-5.8276546	-0.1199586	-1.7920342
H	-4.6718202	1.1692263	-1.3905547
H	-6.3565556	1.2917800	-0.8576861
H	-0.9590119	1.5620036	2.4030738
H	-1.5639593	3.0623278	1.6706138
H	0.1640110	2.6752760	1.5957493
H	-1.1426299	1.9329720	-1.8978420
H	0.0575970	2.8927550	-1.0102401
H	-1.6664764	3.2853329	-0.8721944
H	-1.5381682	-2.5004901	-1.4030657
H	0.1579942	-2.0061805	-1.4983795
H	-1.1020918	-0.9627186	-2.1709953
H	0.3118176	-2.2275202	1.1716859
H	-1.3751494	-2.7636387	1.1279333
H	-0.9054594	-1.3854239	2.1404231
H	1.4815252	1.4506863	0.1836288
H	1.8146082	-1.5643952	-0.2641557
H	3.5201833	1.7086197	0.7600043
H	5.9458032	2.0260762	0.6801303
H	7.4370591	0.2146687	-0.1547602
H	6.4298454	-1.9315587	-0.9251992
H	3.9856169	-2.2414511	-0.8845031

SEt_11.log
E = -1169.983268
H = -1169.583563
G = -1169.658046
NImag=0

C	-0.9828509	-0.8184170	-0.0666948
N	-0.1441736	0.3898879	-0.0026950
C	-0.9730937	1.6258083	0.0547360
N	-2.3531437	1.1401507	0.0456417
C	-2.3527428	-0.1331848	-0.0136203
S	-3.8233840	-1.1120299	-0.0414062
C	-5.1010646	0.2107653	0.0430519
C	-6.4922516	-0.4170589	0.0434186
C	-0.7404711	2.4147619	1.3549727
C	-0.7535508	2.5188966	-1.1780808
C	-0.8202258	-1.5942678	-1.3933239
C	-0.7824259	-1.7429784	1.1535760
C	1.2268884	0.4433203	0.0150483
C	2.1421256	-0.5535761	-0.0731354
C	3.5930955	-0.3614193	-0.0218248
C	4.2160784	0.8226559	0.4254559
C	5.6019260	0.9513211	0.4339320
C	6.4186057	-0.1018009	0.0140798
C	5.8229163	-1.2895555	-0.4127871
C	4.4360635	-1.4153561	-0.4294211
H	-4.9149062	0.7932815	0.9476601
H	-4.9515090	0.8724393	-0.8126045
H	-6.6363865	-1.0779422	0.9033605
H	-6.6739211	-0.9965542	-0.8666737
H	-7.2490227	0.3719540	0.0956058
H	-0.9109786	1.7730234	2.2230366
H	-1.4327707	3.2593222	1.4031055
H	0.2803526	2.8051861	1.4059130
H	-0.9447498	1.9535614	-2.0936466
H	0.2726748	2.8969678	-1.2114685
H	-1.4349711	3.3732495	-1.1459653
H	-1.5320263	-2.4233206	-1.4387115
H	0.1860995	-2.0053490	-1.4901013
H	-1.0030763	-0.9362053	-2.2467827
H	0.2394547	-2.1245905	1.1905117
H	-0.9766714	-1.1987202	2.0814324
H	-1.4653380	-2.5957308	1.1041079
H	1.5933237	1.4619786	0.1082113
H	1.8199472	-1.5783953	-0.2203319
H	3.6117673	1.6474204	0.7921026
H	6.0491129	1.8776067	0.7850836
H	7.4995828	-0.0004128	0.0291414
H	6.4404883	-2.1229908	-0.7369091
H	3.9864811	-2.3444270	-0.7719076

SEt_ME_1.log
E = -1209.697682
H = -1209.255268
G = -1209.332381
NImag=0

C	-1.2840062	1.8208998	0.4056481
N	-1.4440496	0.3663077	0.0026664
C	-0.0758273	-0.1842448	-0.4807593
N	0.7987454	0.9606425	-0.3847117
C	0.1992011	1.9944286	0.0642638
S	0.9297757	3.5681611	0.3053199
C	2.6764217	3.2487130	-0.2059683
C	3.5225019	2.5730451	0.8669922
C	0.4292319	-1.2839562	0.4609804
C	-0.1684284	-0.6319627	-1.9442189
C	-2.1877079	2.7219880	-0.4494987
C	-1.5575903	1.9894638	1.9088445
C	-2.5838560	-0.2273386	0.0728370
C	-2.9583516	-1.6453853	-0.2300759
C	-3.9563157	-1.6764617	-1.4126618
C	-3.5321822	-2.2238734	1.0710468
C	-2.8200738	-3.2122894	1.7623746
C	-3.3297014	-3.7590511	2.9399849
C	-4.5500916	-3.3120780	3.4480768
C	-5.2630030	-2.3214505	2.7703795
C	-4.7577211	-1.7791241	1.5893564
H	3.0506404	4.2459028	-0.4520575
H	2.6314750	2.6607878	-1.1247603
H	3.1371801	1.5787780	1.1027875
H	3.5558359	3.1661726	1.7842353
H	4.5471405	2.4601230	0.4977235
H	1.4637248	-1.5039018	0.1887768
H	-0.1495678	-2.2046708	0.3767596
H	0.4158511	-0.9460730	1.4997804
H	-0.5625012	0.1710601	-2.5717419
H	-0.7833604	-1.5250125	-2.0706696
H	0.8424039	-0.8625707	-2.2872585
H	-3.2453317	2.5373713	-0.2373976
H	-2.0058324	2.5735803	-1.5167224
H	-1.9839880	3.7684622	-0.2127326
H	-0.9182202	1.3364816	2.5076213
H	-2.6042826	1.7725831	2.1429777
H	-1.3569073	3.0226743	2.2003241
H	-3.4086870	0.3930656	0.4240015
H	-2.0770469	-2.2244834	-0.5039462
H	-4.2802946	-2.7076477	-1.5709364
H	-3.4917096	-1.3113070	-2.3329775
H	-4.8451102	-1.0719570	-1.2146165
H	-1.8740589	-3.5753623	1.3700899
H	-2.7748427	-4.5355318	3.4567909
H	-4.9463174	-3.7363994	4.3649279
H	-6.2163125	-1.9750972	3.1565421
H	-5.3378521	-1.0223620	1.0677018

SEt_ME_2.log
E = -1209.697635
H = -1209.255270
G = -1209.332632
NImag=0

C	-1.2282687	1.7762581	0.5323429
N	-1.3641628	0.3184207	0.1317940
C	0.0346340	-0.2438044	-0.2364718
N	0.9041825	0.8995879	-0.0886099
C	0.2785982	1.9382634	0.3108070
S	1.0034299	3.5002360	0.6321058
C	2.7729508	3.1837633	0.2040175
C	3.0671554	3.2374735	-1.2906664
C	0.4619023	-1.3312524	0.7568621
C	0.0538961	-0.7130417	-1.6959399
C	-2.0509087	2.6746951	-0.4040181
C	-1.6253814	1.9618225	2.0054332
C	-2.5107074	-0.2661282	0.1135355
C	-2.8713204	-1.6841156	-0.2057874
C	-3.7530221	-1.7196117	-1.4774304
C	-3.5723354	-2.2388737	1.0419361
C	-4.8418996	-1.7841202	1.4285113
C	-5.4619488	-2.3040205	2.5640320
C	-4.8210039	-3.2815905	3.3272657
C	-3.5572558	-3.7379568	2.9505972
C	-2.9332881	-3.2137307	1.8186159
H	3.0329183	2.2171633	0.6397206
H	3.3060263	3.9646885	0.7526185
H	2.7969460	4.2062276	-1.7181505
H	2.5286032	2.4523576	-1.8256117
H	4.1391184	3.0821658	-1.4512440
H	0.3690281	-0.9787853	1.7867131
H	-0.1107288	-2.2523924	0.6399643
H	1.5139752	-1.5562183	0.5692521
H	-0.2912132	0.0798423	-2.3639534
H	-0.5484774	-1.6093707	-1.8560714
H	1.0881796	-0.9471743	-1.9569126
H	-1.7788681	2.5170825	-1.4505253
H	-1.8620085	3.7217957	-0.1581096
H	-3.1236571	2.4963666	-0.2821812
H	-1.0406702	1.3136728	2.6624569
H	-2.6888677	1.7505416	2.1538746
H	-1.4465071	2.9975309	2.3022801
H	-3.3559985	0.3642090	0.3901977

H	-1.9746954	-2.2756922	-0.3873264
H	-4.6484553	-1.1017469	-1.3720571
H	-4.0738578	-2.7489201	-1.6533841
H	-3.1985100	-1.3720127	-2.3536151
H	-5.3660652	-1.0368146	0.8384821
H	-6.4480363	-1.9505477	2.8478677
H	-5.3064499	-3.6884890	4.2084178
H	-3.0571845	-4.5041277	3.5343664
H	-1.9533900	-3.5828261	1.5281983

SEt_ME_3.log
E = -1209.697725
H = -1209.255251
G = -1209.332847
NImag=0

C	-1.2054614	1.7259218	0.4503155
N	-1.4199860	0.2816379	0.0350878
C	-0.0679206	-0.3228415	-0.4312122
N	0.8525792	0.7831844	-0.3135040
C	0.2900041	1.8365937	0.1388546
S	1.0968203	3.3632142	0.4237234
C	2.8255693	2.9485090	-0.0796195
C	3.7072452	4.1831110	0.0859968
C	0.3761692	-1.4472808	0.5119186
C	-0.1564024	-0.7581314	-1.8985477
C	-2.0506533	2.6701773	-0.4183953
C	-1.5041352	1.8979001	1.9480422
C	-2.5847663	-0.2630911	0.0877014
C	-3.0165189	-1.6617440	-0.2291756
C	-4.0129130	-1.6396551	-1.4133753
C	-3.6185634	-2.2296327	1.0635687
C	-4.8150673	-1.7249452	1.5946025
C	-5.3481849	-2.2603052	2.7665322
C	-4.6926301	-3.3040660	3.4219825
C	-3.5018548	-3.8114561	2.9005247
C	-2.9639703	-3.2718010	1.7322560
H	2.7898258	2.6038847	-1.1148498
H	3.1572649	2.1186843	0.5474919
H	3.7364791	4.5253283	1.1240996
H	3.3688344	5.0107779	-0.5432720
H	4.7289093	3.9313126	-0.2124260
H	0.3640967	-1.1125610	1.5517291
H	-0.2408737	-2.3418116	0.4167095
H	1.4038156	-1.7103575	0.2523975
H	-0.5052094	0.0645752	-2.5273189
H	-0.8068701	-1.6232655	-2.0405977
H	0.8490641	-1.0297846	-2.2269345
H	-1.8554857	2.5163789	-1.4824388
H	-1.8031616	3.7054714	-0.1741845
H	-3.1194414	2.5335197	-0.2271260
H	-1.2701801	2.9210388	2.2501198
H	-2.5632289	1.7207140	2.1588706
H	-0.9036378	1.2174869	2.5562950
H	-3.3856784	0.3905189	0.4334782
H	-2.1590472	-2.2739282	-0.5066030
H	-4.8777839	-1.0036299	-1.2076181
H	-4.3765896	-2.6553786	-1.5847761
H	-3.5336815	-1.2814598	-2.3289338
H	-5.3517781	-0.9256742	1.0899420
H	-6.2788151	-1.8669467	3.1628628
H	-5.1107404	-3.7228232	4.3316353
H	-2.9921542	-4.6294372	3.3995204
H	-2.0420919	-3.6816330	1.3287497

SEt_ME_4.log
E = -1209.697324
H = -1209.254865
G = -1209.331765
NImag=0

C	-1.3259532	1.3303057	0.0116985
N	-0.2622919	0.3818048	0.5086791
C	-0.8895714	-1.0298200	0.7236716
N	-2.2575342	-0.8600110	0.3119907
C	-2.5011051	0.3375187	-0.0561903
S	-4.0567012	0.9310482	-0.6038685
C	-5.0954828	-0.5931630	-0.4992464
C	-4.9351002	-1.5363980	-1.6861016
C	-0.1855449	-2.0505201	-0.1758967
C	-0.8149261	-1.3998419	2.2084675
C	-1.6104972	2.4426867	1.0364988
C	-0.9779229	1.8786099	-1.3828270
C	0.9833786	0.6068357	0.7401877
C	1.7878011	1.8675810	0.6443736
C	2.8961142	1.6939265	-0.4214778
C	2.3332189	2.1365360	2.0539601
C	1.8766092	3.2484329	2.7730125
C	2.3658459	3.5145910	4.0516813
C	3.3064305	2.6628355	4.6322765
C	3.7633090	1.5490254	3.9255832
C	3.2801461	1.2860512	2.6443277
H	-6.1117639	-0.1965860	-0.4290855
H	-4.8478905	-1.0777129	0.4471842
H	-3.9188283	-1.9325453	-1.7401043
H	-5.1709082	-1.0374714	-2.6293467
H	-5.6212940	-2.3815146	-1.5678218
H	-0.7104080	-3.0038479	-0.0863944
H	0.8549030	-2.2098502	0.1212423
H	-0.2195501	-1.7396966	-1.2228257
H	-1.3004130	-0.6410307	2.8265020
H	0.2183658	-1.5291938	2.5427595
H	-1.3405986	-2.3458861	2.3539248
H	-0.7748366	3.1387104	1.1214989
H	-1.8255985	2.0268531	2.0236385
H	-2.4859191	3.0087637	0.7108958
H	-0.7655959	1.0685791	-2.0849192
H	-0.1226884	2.5565099	-1.3512909
H	-1.8328435	2.4396123	-1.7667593
H	1.5446146	-0.2636397	1.0781541
H	1.1549471	2.7060696	0.3550757
H	3.5161696	2.5932471	-0.4327321
H	2.4699884	1.5516522	-1.4187567
H	3.5443509	0.8418944	-0.2011989
H	1.1540614	3.9259524	2.3261184
H	2.0145734	4.3885242	4.5908873
H	3.6858972	2.8685563	5.6279169
H	4.5012468	0.8876742	4.3682023
H	3.6636500	0.4239383	2.1045422

SEt_ME_5.log
E = -1209.697394
H = -1209.254888
G = -1209.331606
NImag=0

C	-1.2953994	1.1823434	0.4976138
N	-0.2625130	0.0818244	0.4785154
C	-0.8895861	-1.2036053	-0.1436714
N	-2.2241381	-0.7956742	-0.4930392
C	-2.4490409	0.4217530	-0.1827842
S	-3.9500328	1.2788016	-0.4740351
C	-4.9894135	-0.0440322	-1.2377310
C	-5.6114908	-1.0022237	-0.2284377
C	-0.1099985	-1.6033605	-1.4003031
C	-0.9225433	-2.3154450	0.9099229
C	-1.6758591	1.5704844	1.9374994
C	-0.8449419	2.3874545	-0.3458695
C	0.9598542	0.1072954	0.8797572
C	1.7554148	1.1968915	1.5314728
C	2.9346528	1.5957314	0.6116399
C	2.2045869	0.6506139	2.8936416
C	3.1089245	-0.4176932	2.9894963
C	3.5085632	-0.8941624	4.2375239
C	3.0100674	-0.3102217	5.4034116
C	2.1109313	0.7533946	5.3167293
C	1.7047443	1.2257405	4.0688081
H	-4.3565763	-0.5621088	-1.9608420
H	-5.7486842	0.5207749	-1.7849671
H	-6.2377827	-0.4728495	0.4939229
H	-4.8424660	-1.5588541	0.3114365
H	-6.2410092	-1.7238911	-0.7594083
H	-0.0663021	-0.7770097	-2.1137020
H	0.9057482	-1.9338039	-1.1648706
H	-0.6327386	-2.4360184	-1.8753381
H	-1.4667777	-1.9955658	1.8015765
H	0.0839735	-2.6318789	1.1979814
H	-1.4410401	-3.1770790	0.4842531
H	-1.9633680	0.6932917	2.5218479
H	-2.5303833	2.2499186	1.9060753
H	-0.8589793	2.0821678	2.4484499
H	-0.5619612	2.0803517	-1.3556841
H	-0.0079903	2.9163967	0.1141393
H	-1.6754595	3.0918677	-0.4302712
H	1.5026998	-0.8258524	0.7335440
H	1.1342223	2.0755266	1.7025539
H	3.5769276	0.7423040	0.3795602
H	3.5430096	2.3435571	1.1255029
H	2.5777973	2.0230072	-0.3298285
H	3.5248891	-0.8740037	2.0947788
H	4.2144584	-1.7162667	4.2983981
H	3.3251076	-0.6791414	6.3741101
H	1.7274850	1.2189793	6.2189898
H	1.0136466	2.0626081	4.0133838

SEt_ME_6.log
E = -1209.697373
H = -1209.254949
G = -1209.332639

NImag=0

C	1.2929513	-1.0379988	0.6696031
N	0.2084287	0.0087744	0.5885188
C	0.7923837	1.3085435	-0.0464257
N	2.1545687	0.9581316	-0.3478955
C	2.4290455	-0.2372944	0.0066758
S	3.9881939	-1.0077346	-0.1996026
C	4.9528157	0.3561684	-0.9890353
C	6.3760873	-0.1252958	-1.2563861
C	0.0314739	1.6434881	-1.3330043
C	0.7431206	2.4429104	0.9822084
C	1.6431901	-1.3737196	2.1299293
C	0.9305285	-2.2828574	-0.1581741
C	-1.0244665	-0.0687150	0.9485662
C	-1.7888176	-1.1804736	1.6004771
C	-2.9036160	-1.6700391	0.6447092
C	-2.3237124	-0.6176626	2.9246228
C	-3.2992425	0.3903919	2.9477244
C	-3.7727800	0.8863039	4.1617766
C	-3.2773637	0.3828249	5.3658783
C	-2.3072061	-0.6200797	5.3516425
C	-1.8277443	-1.1118030	4.1376736
H	4.9273842	1.2091541	-0.3081761
H	4.4324481	0.6341923	-1.9075588
H	6.3942274	-0.9806089	-1.9373126
H	6.8903027	-0.4053094	-0.3329388
H	6.9425410	0.6860177	-1.7223870
H	0.0486770	0.8017214	-2.0294039
H	-1.0054477	1.9285477	-1.1337521
H	0.5271374	2.4906987	-1.8113048
H	1.2734886	2.1658990	1.8962678
H	-0.2849559	2.7192646	1.2332515
H	1.2361426	3.3178088	0.5533251
H	1.8647578	-0.4701195	2.7026529
H	2.5323233	-2.0079258	2.1433542
H	0.8371733	-1.9170223	2.6253540
H	1.7980364	-2.9451185	-0.2017429
H	0.1074439	-2.8433751	0.2892232
H	0.6632255	-2.0120795	-1.1825658
H	-1.6079487	0.8318888	0.7601742
H	-1.1308783	-2.0193959	1.8255344
H	-3.5741826	-0.8588662	0.3499239
H	-3.4990907	-2.4275732	1.1593967
H	-2.4825828	-2.1135340	-0.2620534
H	-3.7118282	0.7837397	2.0222102
H	-4.5331619	1.6606946	4.1664561
H	-3.6496559	0.7670014	6.3100413
H	-1.9250514	-1.0231111	6.2840540
H	-1.0807001	-1.9010703	4.1385480

SEt_MS_1.log
E = -1209.637371
H = -1209.197155
G = -1209.277321
NImag=0

C	0.9300310	-0.8719740	0.4406860
N	-0.0883220	0.1150640	0.0465480
C	0.4852840	1.4559690	-0.2098020
N	1.9484440	1.2359440	-0.0864840
C	2.1290290	0.0372450	0.2446110
S	3.8514320	-0.5692960	0.4885280
C	4.4969610	-0.4531920	-1.2510730
C	5.9776640	-0.8030520	-1.3355750
C	0.2126140	1.9504260	-1.6389250
C	0.1065530	2.5033660	0.8534160
C	0.8121250	-1.3308800	1.9123210
C	0.9821390	-2.0938150	-0.5029840
C	-1.4405180	-0.2184160	0.0641430
C	-2.5185220	0.5659250	-0.1385280
C	4.6089280	0.8804810	1.2982610
C	-3.9097950	0.0917620	-0.1370180
C	-4.2690910	-1.2592930	-0.3023380
C	-5.6031890	-1.6550290	-0.2657740
C	-6.6167040	-0.7113890	-0.0815050
C	-6.2799150	0.6350500	0.0609120
C	-4.9442960	1.0299700	0.0316300
H	4.2610150	0.5584900	-1.5902240
H	3.8777660	-1.1670950	-1.8007000
H	6.1855350	-1.8060690	-0.9546900
H	6.6096100	-0.0819590	-0.8117180
H	6.2659100	-0.7805750	-2.3907160
H	0.5845490	1.2274170	-2.3690640
H	0.7021930	2.9125710	-1.8060760
H	-0.8596000	2.0703490	-1.7993720
H	-0.9656280	2.7004910	0.8308070
H	0.6365000	3.4397590	0.6637620
H	0.3628910	2.1458360	1.8539090
H	-0.1196200	-1.8855540	2.0504500
H	0.7980470	-0.4760080	2.5930230
H	1.6334100	-2.0000800	2.1906700
H	1.0645620	-1.7813190	-1.5472270
H	0.0693250	-2.6857780	-0.4006980
H	1.8174360	-2.7569570	-0.2501730
H	-1.6008640	-1.2730870	0.2659350
H	-2.3994120	1.6330460	-0.2944710
H	4.2700400	0.8796570	2.3350300
H	4.2824750	1.7818900	0.7788690
H	5.6907000	0.7489600	1.2622710
H	-3.5035110	-2.0074540	-0.4882680
H	-5.8550910	-2.7031880	-0.3979230
H	-7.6565090	-1.0220130	-0.0614850
H	-7.0580380	1.3803640	0.1956450
H	-4.6932480	2.0810690	0.1507910

SEt_MS_2.log
E = -1209.637608
H = -1209.197319
G = -1209.277409
NImag=0

C	0.7663000	-0.6922470	0.2639640
N	-0.0851720	0.4898390	0.0801740
C	0.6819590	1.7592140	0.1260120
N	2.0926390	1.3106150	0.2006100
C	2.0895710	0.0553560	0.2566490
S	3.6984240	-0.8383550	0.3450490
C	4.3566520	-0.5017610	-1.3612580
C	5.7606100	-1.0617440	-1.5540350
C	0.5239700	2.5919800	-1.1559260
C	0.3840090	2.5967580	1.3810210
C	0.5816800	-1.4096280	1.6243730
C	0.6797020	-1.6904020	-0.9122400
C	-1.4742980	0.4937230	-0.0076370
C	-2.3416340	-0.5332630	0.1009370
C	4.6676060	0.3037880	1.3875130
C	-3.7976340	-0.4189190	-0.0634510
C	-4.4186860	0.6551350	-0.7284890
C	-5.8050860	0.7270430	-0.8324440
C	-6.6087030	-0.2798800	-0.2922820
C	-6.0085030	-1.3632160	0.3503320
C	-4.6215070	-1.4313540	0.4609830
H	4.2895380	0.5800640	-1.4998440
H	3.6298370	-0.9913040	-2.0147780
H	6.0491770	-0.8868280	-2.5947560
H	5.8021590	-2.1387250	-1.3733210
H	6.5013340	-0.5631780	-0.9244440
H	0.7561960	1.9899540	-2.0376630
H	1.1876760	3.4593090	-1.1270870
H	-0.5025580	2.9544090	-1.2483440
H	0.5503000	2.0109680	2.2884730
H	-0.6609750	2.9164330	1.3695090
H	1.0168560	3.4871150	1.4099720
H	1.3254140	-2.2034290	1.7531720
H	-0.4031700	-1.8738710	1.6820110
H	0.6666860	-0.7025140	2.4533970
H	0.8783380	-1.1892440	-1.8635210
H	-0.3202050	-2.1219140	-0.9710530
H	1.3848890	-2.5184980	-0.7790620
H	-1.8689470	1.4860540	-0.2020390
H	-1.9871150	-1.5280690	0.3512430
H	5.7179430	0.0286360	1.2875440
H	4.3408600	0.1495620	2.4166760
H	4.4730570	1.3262220	1.0624970
H	-3.8148170	1.4297720	-1.1929990
H	-6.2605910	1.5650670	-1.3517750
H	-7.6889710	-0.2252550	-0.3822370
H	-6.6207890	-2.1571090	0.7674860
H	-4.1652250	-2.2766190	0.9705370

SEt_MS_3.log
E = -1209.637379
H = -1209.197107
G = -1209.276549
NImag=0

C	0.9314410	-0.9588900	0.1369490
N	-0.0891140	0.0991560	0.0661600
C	0.4860140	1.4584670	0.1862570
N	1.9463510	1.2110720	0.2803380
C	2.1282240	-0.0317820	0.2431950
S	3.8494110	-0.6832700	0.3232960
C	4.5086980	-0.0866570	-1.3098450
C	5.9848490	-0.4211790	-1.4858220
C	0.2610880	2.3192840	-1.0687620
C	0.0614460	2.1822490	1.4750770
C	0.8108520	-1.8523910	1.3932010
C	0.9906250	-1.8264150	-1.1396100
C	-1.4425490	-0.2255130	0.0155050
C	-2.5156640	0.5885380	-0.0521990

C	4.5983900	0.4839660	1.5099400
C	-3.9115790	0.1290330	-0.0850220
C	-4.3154830	-1.1522880	0.3353000
C	-5.6493170	-1.5435210	0.2601440
C	-6.6198760	-0.6606160	-0.2195030
C	-6.2404830	0.6211740	-0.6190790
C	-4.9044710	1.0100730	-0.5505000
H	4.2895940	0.9832810	-1.3472320
H	3.8834330	-0.6045420	-2.0419020
H	6.1749910	-1.4946190	-1.4094960
H	6.2826950	-0.1002010	-2.4884340
H	6.6227970	0.1076290	-0.7736760
H	0.6263430	1.7993610	-1.9579470
H	0.7864270	3.2721690	-0.9702850
H	-0.8012840	2.5212480	-1.2088680
H	0.5465870	3.1589130	1.5377200
H	0.3339440	1.5919400	2.3536160
H	-1.0197530	2.3256610	1.4873610
H	1.6457080	-2.5579280	1.4620320
H	-0.1063600	-2.4444640	1.3430370
H	0.7733660	-1.2488480	2.3034420
H	0.0658000	-2.4000860	-1.2407050
H	1.8120430	-2.5509700	-1.0948540
H	1.0973010	-1.2062140	-2.0335830
H	-1.6091250	-1.2983470	0.0208640
H	-2.3866370	1.6640790	-0.1162080
H	4.2657990	0.1851790	2.5048190
H	5.6811160	0.3796520	1.4342230
H	4.2601370	1.4920820	1.2682690
H	-3.5874120	-1.8432730	0.7512820
H	-5.9359980	-2.5367700	0.5930250
H	-7.6604220	-0.9653300	-0.2691520
H	-6.9856630	1.3208070	-0.9856560
H	-4.6198950	2.0091250	-0.8712780

SEt_MS_4.log
E =   -1209.637766
H =   -1209.197401
G =   -1209.277178
NImag=0

C	-0.8210620	-0.7948440	-0.0135670
N	0.0059540	0.3976290	-0.2401510
C	-0.8011960	1.6223670	-0.4634400
N	-2.1991470	1.1335350	-0.4107460
C	-2.1625610	-0.1007300	-0.1794060
S	-3.7529550	-1.0299940	-0.0673720
C	-4.8168920	0.1835440	0.8442780
C	-4.5132690	0.1761230	2.3370760
C	-0.6312610	2.6685170	0.6512160
C	-0.5657020	2.2379220	-1.8517330
C	-0.6480610	-1.8887750	-1.0941550
C	-0.6816270	-1.3854220	1.4087060
C	1.3941280	0.4606010	-0.1568170
C	2.2873530	-0.5350260	0.0155870
C	-4.3648570	-0.8434390	-1.7809620
C	3.7423880	-0.3487700	0.1073560
C	4.5823060	-1.4569260	-0.1054800
C	5.9688620	-1.3337950	-0.0493390
C	6.5526590	-0.0967850	0.2253040
C	5.7333800	1.0113700	0.4544640
C	4.3478400	0.8866420	0.4054480
H	-5.8366600	-0.1464150	0.6267930
H	-4.6258160	1.1506160	0.3733040
H	-4.6777880	-0.8058150	2.7870840
H	-5.1899520	0.8854830	2.8219910
H	-3.4910300	0.5009770	2.5447830
H	-0.8275090	2.2266860	1.6313390
H	-1.3136690	3.5069080	0.4927810
H	0.3900700	3.0563370	0.6497350
H	-0.7390510	1.4980360	-2.6369340
H	0.4674060	2.5847500	-1.9329280
H	-1.2284370	3.0921030	-2.0099780
H	-0.7492220	-1.4646500	-2.0965440
H	-1.3836190	-2.6907720	-0.9613010
H	0.3389150	-2.3467880	-1.0247490
H	-0.8930840	-0.6285530	2.1686940
H	0.3376620	-1.7387530	1.5690780
H	-1.3554590	-2.2364760	1.5508440
H	1.7642060	1.4754290	-0.2624180
H	1.9565770	-1.5675440	0.0671960
H	-3.7349980	-1.4615910	-2.4218540
H	-4.3068330	0.2076340	-2.0660200
H	-5.3913360	-1.2135380	-1.8016810
H	4.1385950	-2.4244760	-0.3274760
H	6.5934220	-2.2053240	-0.2211600
H	7.6325230	0.0020000	0.2719600
H	6.1765820	1.9749750	0.6879040
H	3.7335660	1.7550710	0.6265790

SEt_MS_5.log
E =   -1209.637525
H =   -1209.197266
G =   -1209.277230
NImag=0

C	-0.9973180	-0.9580650	-0.3297730
N	0.0086430	0.0861280	-0.0785210
C	-0.5907700	1.4266240	0.1097560
N	-2.0502720	1.1693150	0.0213830
C	-2.2107710	-0.0553420	-0.2081210
S	-3.9264170	-0.7004460	-0.4184490
C	-4.8238130	0.2145950	0.9214260
C	-4.5773530	-0.4169100	2.2856100
C	-0.3076190	2.0157440	1.5008450
C	-0.2502040	2.4136710	-1.0216130
C	-0.8978080	-1.5779110	-1.7424440
C	-1.0076050	-2.0663170	0.7465930
C	1.3660730	-0.2247450	-0.0675540
C	2.4304590	0.5933920	0.0601760
C	-4.4419850	0.2255010	-1.9090920
C	3.8295000	0.1431870	0.0897180
C	4.2115430	-1.1765460	0.3957830
C	5.5510390	-1.5556170	0.3826440
C	6.5472460	-0.6236490	0.0818810
C	6.1878870	0.6946390	-0.2006230
C	4.8469400	1.0721260	-0.1944840
H	-5.8737100	0.1565310	0.6207480
H	-4.4708950	1.2466010	0.8570220
H	-4.9018050	-1.4595970	2.3224240
H	-5.1578440	0.1417820	3.0252910
H	-3.5262190	-0.3557330	2.5772580
H	-0.6494470	1.3317400	2.2816420
H	-0.8180180	2.9746240	1.6163970
H	0.7638150	2.1707700	1.6346240
H	-0.7939510	3.3505810	-0.8796020
H	-0.5165830	1.9903490	-1.9934790
H	0.8184060	2.6301810	-1.0303090
H	-0.9050190	-0.8040460	-2.5144870
H	0.0380440	-2.1347000	-1.8366120
H	-1.7131160	-2.2866230	-1.9281890
H	-1.0798740	-1.6392200	1.7499880
H	-0.0831370	-2.6468530	0.6936580
H	-1.8350070	-2.7666180	0.5903380
H	1.5452080	-1.2895700	-0.1816470
H	2.2937100	1.6680590	0.1223220
H	-3.8971440	-0.1918610	-2.7568290
H	-4.2056290	1.2809590	-1.7691460
H	-5.5130480	0.0622780	-2.0400890
H	3.4595470	-1.9095470	0.6744910
H	5.8207320	-2.5794290	0.6247330
H	7.5914250	-0.9198870	0.0804950
H	6.9526750	1.4315500	-0.4269100
H	4.5783010	2.1004460	-0.4233640

SEt_MS_6.log
E =   -1209.637574
H =   -1209.197251
G =   -1209.277234
NImag=0

C	-0.9964020	-0.9994830	0.1686460
N	0.0114600	0.0286730	-0.1356290
C	-0.5906190	1.3268840	-0.5156040
N	-2.0486380	1.0463450	-0.5142850
C	-2.2091200	-0.1475930	-0.1579170
S	-3.9230510	-0.8217400	-0.0562590
C	-4.8351550	0.6168230	0.6753720
C	-4.6181690	0.7025600	2.1807720
C	-0.3550300	2.4405330	0.5207920
C	-0.2071350	1.7647490	-1.9380860
C	-0.9104760	-2.2304480	-0.7616300
C	-0.9981620	-1.4407100	1.6495970
C	1.3698880	-0.2481250	-0.0023280
C	2.4279830	0.5772710	-0.1352710
C	-4.4226910	-0.7232650	-1.8130800
C	3.8324150	0.1637880	-0.0052600
C	4.2606080	-1.1723780	-0.1182780
C	5.6019860	-1.5099950	0.0389060
C	6.5554050	-0.5216680	0.2952670
C	6.1516290	0.8106320	0.3879690
C	4.8082400	1.1474980	0.2377010
H	-5.8802210	0.4244020	0.4167520
H	-4.4709460	1.4961880	0.1389910
H	-3.5709080	0.8890880	2.4303370
H	-5.2024260	1.5467110	2.5579630
H	-4.9570550	-0.1981030	2.6982390
H	0.7048740	2.6887700	0.5814800
H	-0.6828980	2.1177980	1.5122400
H	-0.9071520	3.3397770	0.2376490

H	-0.7162790	2.6957150	-2.1975510
H	-0.4813590	0.9931090	-2.6617230
H	0.8699630	1.9231250	-2.0062270
H	-1.7407640	-2.9231330	-0.5823480
H	0.0102390	-2.7860170	-0.5667700
H	-0.9056130	-1.9296050	-1.8124270
H	-1.8147530	-2.1400120	1.8590050
H	-1.0811590	-0.5797120	2.3176520
H	-0.0623860	-1.9557450	1.8816500
H	1.5556370	-1.2898690	0.2406150
H	2.2788410	1.6347890	-0.3267380
H	-4.1959800	0.2739200	-2.1922270
H	-3.8638210	-1.4873060	-2.3548740
H	-5.4908100	-0.9423420	-1.8597150
H	3.5459540	-1.9547260	-0.3583940
H	5.9080720	-2.5477810	-0.0552360
H	7.6016640	-0.7870800	0.4091040
H	6.8832820	1.5901750	0.5779950
H	4.5038980	2.1881290	0.3186990

SEt_MS_7.log
E = -1209.637805
H = -1209.197426
G = -1209.276787
NImag=0

C	-0.8164790	-0.7651070	-0.1755820
N	0.0097330	0.4411000	-0.0395860
C	-0.7983200	1.6758640	0.1125980
N	-2.1941390	1.1983680	-0.0293540
C	-2.1562290	-0.0475700	-0.1856590
S	-3.7403770	-0.9636690	-0.4197260
C	-4.8744400	-0.0244040	0.7057040
C	-4.6788680	-0.4382540	2.1588210
C	-0.6532870	2.3178240	1.5020850
C	-0.5410170	2.6974260	-1.0070570
C	-0.5799830	-1.5191480	-1.5039330
C	-0.7434880	-1.7272490	1.0353200
C	1.4003590	0.4839020	0.0036050
C	2.2878260	-0.5315700	0.0115820
C	-4.2417770	-0.3350260	-2.0627860
C	3.7482810	-0.3673210	0.0152650
C	4.3947230	0.8202570	-0.3763140
C	5.7816930	0.9296100	-0.3278090
C	6.5626170	-0.1489710	0.0944650
C	5.9394630	-1.3413100	0.4642240
C	4.5510950	-1.4481350	0.4229710
H	-5.8738790	-0.2689340	0.3350510
H	-4.6542900	1.0308820	0.5283510
H	-4.8686160	-1.5030210	2.3139050
H	-5.3934070	0.1225870	2.7680050
H	-3.6764400	-0.1944210	2.5183160
H	-0.8890340	1.5974230	2.2894230
H	-1.3146310	3.1824090	1.5977670
H	0.3764610	2.6538960	1.6467080
H	-0.6712280	2.2355650	-1.9886970
H	0.4797030	3.0812430	-0.9411740
H	-1.2275040	3.5420690	-0.9129830
H	-0.7094150	-0.8515530	-2.3601900
H	-1.2597520	-2.3732680	-1.5989360
H	0.4383900	-1.9077330	-1.5412950
H	-0.9166330	-1.1900230	1.9712730
H	0.2401670	-2.1937260	1.0959540
H	-1.4811050	-2.5314870	0.9415080
H	1.7770940	1.5016340	0.0285190
H	1.9450080	-1.5608400	0.0440880
H	-3.5575700	-0.7604470	-2.7979330
H	-4.1886930	0.7542390	-2.0583010
H	-5.2562430	-0.6905240	-2.2514320
H	3.8133520	1.6594190	-0.7479360
H	6.2571430	1.8558610	-0.6367900
H	7.6442480	-0.0634780	0.1229860
H	6.5347400	-2.1904830	0.7862840
H	4.0759770	-2.3797610	0.7207230

SEt_MS_8.log
E = -1209.637586
H = -1209.197304
G = -1209.276963
NImag=0

C	-0.7582740	-0.6761700	-0.2861530
N	0.0936440	0.4636910	0.0764140
C	-0.6795280	1.7109270	0.2974590
N	-2.0856570	1.2915270	0.0914310
C	-2.0786390	0.0705140	-0.2049420
S	-3.6845760	-0.7746140	-0.5270760
C	-4.4934360	-0.6040640	1.1383340
C	-5.9264730	-1.1220260	1.1331230
C	-0.5459260	2.2381850	1.7346560
C	-0.3674590	2.8071320	-0.7355410
C	-0.5167660	-1.2011560	-1.7208100
C	-0.7310890	-1.8295470	0.7449960
C	1.4851590	0.4691080	0.1075700
C	2.3487420	-0.5564540	-0.0386350
C	-4.5362840	0.4977460	-1.5213140
C	3.8125630	-0.4252930	-0.0104740
C	4.4835450	0.7912850	-0.2365900
C	5.8721380	0.8658570	-0.1716460
C	6.6302250	-0.2752580	0.1014370
C	5.9825660	-1.4943120	0.3044020
C	4.5925380	-1.5672430	0.2470810
H	-4.4045460	0.4517370	1.4054590
H	-3.8490710	-1.1942720	1.7953660
H	-5.9848680	-2.1694330	0.8267330
H	-6.3097220	-1.0523680	2.1554820
H	-6.5859860	-0.5251290	0.4987170
H	-0.8259170	1.4670870	2.4565670
H	-1.1797400	3.1158630	1.8825620
H	0.4905470	2.5252540	1.9283940
H	-0.4873930	2.4266770	-1.7528630
H	0.6619030	3.1539440	-0.6182340
H	-1.0319730	3.6624950	-0.5924560
H	-0.6205880	-0.3965940	-2.4535740
H	-1.2157320	-2.0068170	-1.9683210
H	0.4939900	-1.6012890	-1.8093990
H	-1.4841710	-2.5888700	0.5036360
H	-0.9057900	-1.4521380	1.7560490
H	0.2383610	-2.3285630	0.7394030
H	1.8870100	1.4611820	0.2879270
H	1.9827720	-1.5712590	-0.1590910
H	-4.1073030	0.4592590	-2.5234530
H	-5.5928920	0.2316890	-1.5613710
H	-4.3712490	1.4715160	-1.0590930
H	3.9198510	1.6839790	-0.4925850
H	6.3666510	1.8159030	-0.3515210
H	7.7131850	-0.2158090	0.1429320
H	6.5600200	-2.3909920	0.5085680
H	4.0983590	-2.5211250	0.4147590

SEt_MN2_1.log
E = -1209.660786
H = -1209.217650
G = -1209.292286
NImag=0

C	-1.7577728	1.3628370	1.4250717
N	-1.2224277	1.0901276	-0.0422973
C	-0.3052524	-0.2954078	0.1760040
N	0.0571953	-0.2032063	1.5573502
C	-0.6544342	0.6360716	2.2091743
S	-0.4866311	0.9879094	3.9161023
C	0.8046435	-0.2311394	4.4245712
C	0.2716502	-1.6408976	4.6544008
C	-1.1211849	-1.5631772	-0.1020601
C	0.9197705	-0.2682030	-0.7372190
C	-1.8823646	2.8641148	1.7174280
C	-3.1215368	0.7016438	1.7005962
C	-0.2964640	2.1774848	-0.4091434
C	-0.3721021	2.9483587	-1.5007684
C	-2.3096905	0.9201620	-1.0493714
C	0.5608755	4.0222273	-1.8612667
C	1.6551044	4.4040956	-1.0615596
C	2.5014879	5.4293034	-1.4669937
C	2.2748468	6.0948020	-2.6764120
C	1.1940499	5.7289587	-3.4782797
C	0.3444397	4.7021763	-3.0727680
H	1.2119738	0.2043518	5.3407808
H	1.5794468	-0.2057700	3.6557939
H	-0.1312563	-2.0652990	3.7320973
H	-0.5077874	-1.6555741	5.4201918
H	1.0911893	-2.2840162	4.9914528
H	-2.0301452	-1.6381349	0.4933551
H	-0.4862682	-2.4071986	0.1769715
H	-1.3649928	-1.6691864	-1.1614674
H	1.6242215	0.5214634	-0.4811088
H	0.6373601	-0.1720510	-1.7884228
H	1.4349293	-1.2236094	-0.6116388
H	-0.9306264	3.3930846	1.7164962
H	-2.5510931	3.3439394	0.9975053
H	-2.3272896	2.9838791	2.7082737
H	-3.1443089	-0.3668474	1.4918374
H	-3.9248982	1.1970433	1.1497881
H	-3.3322783	0.8219805	2.7655847
H	0.4904847	2.2888010	0.3231008
H	-1.1765309	2.8024671	-2.2150130
H	-2.9621962	0.1051757	-0.7573053
H	-1.8609015	0.6956953	-2.0163677
H	-2.8867142	1.8427870	-1.1187802
H	1.8511925	3.9054198	-0.1170608

```
H       3.3407530      5.7134629     -0.8402649
H       2.9387129      6.8946975     -2.9882925
H       1.0116295      6.2416162     -4.4171242
H      -0.4968088      4.4211806     -3.7010423
```

SEt_MN2_2.log

```
E =     -1209.660560
H =     -1209.217514
G =     -1209.291977
NImag=0
C      -1.8901508      1.2957756      1.3752554
N      -1.3132580      1.0367082     -0.0798426
C      -0.4590200     -0.3862405      0.1350113
N      -0.1367545     -0.3383596      1.5290874
C      -0.8502359      0.5003936      2.1796739
S      -0.7687026      0.7640673      3.9086822
C       0.4886828     -0.4883460      4.4233248
C       1.9301816     -0.0416056      4.2068035
C      -1.3158266     -1.6150396     -0.1902112
C       0.7916373     -0.3893595     -0.7428106
C      -1.9535060      2.7925707      1.7080667
C      -3.2958435      0.6977320      1.5790491
C      -0.3370088      2.0974684     -0.3905120
C      -0.3617230      2.9078854     -1.4555925
C      -2.3703160      0.9298798     -1.1279518
C       0.6126302      3.9619835     -1.7596740
C       1.7247266      4.2540773     -0.9469149
C       2.6054971      5.2712306     -1.2954345
C       2.3968869      6.0173058     -2.4602573
C       1.3002973      5.7390413     -3.2757585
C       0.4166847      4.7200550     -2.9275818
H       0.2578645     -1.4080773      3.8822631
H       0.2662780     -0.6451855      5.4820902
H       2.1406740      0.1063779      3.1454468
H       2.6063408     -0.8175000      4.5805380
H       2.1477163      0.8847032      4.7443886
H      -0.7279895     -2.4880231      0.1028521
H      -1.5240103     -1.7000687     -1.2590685
H      -2.2480819     -1.6570678      0.3713826
H       1.5200118      0.3646049     -0.4489985
H       0.5434051     -0.2577735     -1.7989053
H       1.2639258     -1.3677569     -0.6261008
H      -2.5742448      3.3246750      0.9819202
H      -2.4249240      2.9038998      2.6875457
H      -0.9782480      3.2743448      1.7538009
H      -3.3661276     -0.3628509      1.3413570
H      -4.0487683      1.2477902      1.0093265
H      -3.5448857      0.8033159      2.6371303
H       0.4353636      2.1559423      0.3630264
H      -1.1575211      2.8173825     -2.1882898
H      -3.0600956      0.1311928     -0.8805800
H      -1.8948793      0.7155391     -2.0844791
H      -2.9135215      1.8730954     -1.1922049
H       1.9097859      3.6895159     -0.0378851
H       3.4582633      5.4856564     -0.6592618
H       3.0875747      6.8105783     -2.7276157
H       1.1322252      6.3135048     -4.1808960
H      -0.4365104      4.5073605     -3.5664956
```

SEt_MN2_3.log

```
E =     -1209.660746
H =     -1209.217630
G =     -1209.292671
NImag=0
C      -1.7965034      1.2788571      1.3438927
N      -1.2727449      1.0673209     -0.1378326
C      -0.3932002     -0.3543991      0.0059448
N      -0.0271018     -0.3457384      1.3887229
C      -0.7190746      0.4737116      2.0859459
S      -0.5520993      0.6972382      3.8131890
C       0.7063816     -0.5926215      4.2204934
C       0.9941295     -0.5580529      5.7189723
C      -1.2462388     -1.5822927     -0.3328701
C       0.8300986     -0.3130618     -0.9086720
C      -1.8640878      2.7643799      1.7231435
C      -3.1844189      0.6550602      1.5833522
C      -0.3192338      2.1463379     -0.4543710
C      -0.3824421      2.9764026     -1.5026278
C      -2.3667424      0.9773220     -1.1480687
C       0.5738946      4.0453381     -1.8128697
C       1.6698660      4.3726914     -0.9917551
C       2.5384123      5.3971229     -1.3496231
C       2.3328184      6.1161106     -2.5318519
C       1.2503021      5.8049045     -3.3542072
C       0.3783397      4.7792657     -2.9961257
H       1.5960693     -0.3794735      3.6247954
H       0.3024516     -1.5552933      3.9008080
H       0.0985549     -0.7698994      6.3093414
H       1.3963338      0.4090444      6.0328708
H       1.7393926     -1.3231555      5.9543281
H      -2.1556973     -1.6593908      0.2617479
H      -0.6360657     -2.4570859     -0.0965930
H      -1.4943793     -1.6293129     -1.3954916
H       1.5582559      0.4397461     -0.6109979
H       0.5495913     -0.1501582     -1.9521776
H       1.3166519     -1.2888806     -0.8379143
H      -0.8930814      3.2566914      1.7470609
H      -2.5175146      3.3117970      1.0383799
H      -2.2992873      2.8407451      2.7226295
H      -3.2464602     -0.4000814      1.3203382
H      -3.9665117      1.2071601      1.0562153
H      -3.3952131      0.7290187      2.6524379
H       0.4760397      2.1969926      0.2754559
H      -1.1945443      2.8883064     -2.2176386
H      -3.0382928      0.1643300     -0.8966372
H      -1.9261218      0.7933015     -2.1273490
H      -2.9212378      1.9159976     -1.1666912
H       1.8492564      3.8330648     -0.0665792
H       3.3783057      5.6393903     -0.7063479
H       3.0138277      6.9152966     -2.8063404
H       1.0836327      6.3594193     -4.2719587
H      -0.4640109      4.5410322     -3.6403746
```

SEt_MN2_4.log

```
E =     -1209.660241
H =     -1209.216670
G =     -1209.291171
NImag=0
C      -1.1515776      1.6681375      1.7576097
N      -0.7507539      1.3275968      0.2581187
C       0.1760968     -0.0685143      0.4715162
N      -0.3359142     -0.5884896      1.6985191
C      -1.0141353      0.2651189      2.3658983
S      -1.7617089     -0.0381803      3.9205207
C      -1.1702493     -1.7469809      4.2956163
C       0.2504321     -1.8053540      4.8458497
C      -0.0286424     -1.0426758     -0.6875705
C       1.6661935      0.2721957      0.5827983
C      -0.1629198      2.6400089      2.4300676
C      -2.5558435      2.2777409      1.8676751
C      -0.0108298      2.4067054     -0.4013445
C      -0.3691757      3.0206218     -1.5397343
C      -1.9853418      0.9489465     -0.5082030
C       0.3699879      4.0914655     -2.2150857
C       1.5933449      4.6106068     -1.7492419
C       2.2364881      5.6292995     -2.4418781
C       1.6747012      6.1511590     -3.6122316
C       0.4642039      5.6467541     -4.0867379
C      -0.1814113      4.6256106     -3.3935681
H      -1.2788951     -2.3260789      3.3764374
H      -1.9025817     -2.1103897      5.0213004
H       0.9731986     -1.4410627      4.1123657
H       0.5026547     -2.8448423      5.0797433
H       0.3505105     -1.2179310      5.7618965
H       0.6569833     -1.8773625     -0.5244569
H       0.2293337     -0.5857593     -1.6467436
H      -1.0348272     -1.4583844     -0.7257818
H       1.8927032      0.9748546      1.3835349
H       2.0658514      0.6470013     -0.3617316
H       2.1815734     -0.6609819      0.8210649
H       0.8744057      2.3110564      2.3907312
H      -0.2427763      3.6404495      1.9988093
H      -0.4365773      2.7056367      3.4852022
H      -2.6258313      3.1938050      1.2736682
H      -2.7217526      2.5580429      2.9103689
H      -3.3583711      1.5960527      1.5878916
H       0.8913239      2.6711344      0.1248859
H      -1.2856521      2.7338977     -2.0453212
H      -2.4913239      0.1385394      0.0117449
H      -1.7019772      0.6252975     -1.5051842
H      -2.6465493      1.8080982     -0.5815906
H       2.0509273      4.2211895     -0.8447887
H       3.1789515      6.0200011     -2.0718045
H       2.1813071      6.9466801     -4.1491884
H       0.0231473      6.0464540     -4.9940362
H      -1.1247017      4.2356284     -3.7671116
```

SEt_MN2_5.log

```
E =     -1209.660013
H =     -1209.216443
G =     -1209.290803
NImag=0
C      -1.0268249      1.7149852      1.8354122
N      -0.6252924      1.3402290      0.3442177
C       0.3831524      0.0090498      0.5965874
N      -0.0826480     -0.4916735      1.8503466
```

C	-0.7969607	0.3485392	2.4966563
S	-1.4690904	0.0807465	4.0913456
C	-0.7657142	-1.5709609	4.5281866
C	-1.5249918	-2.7499309	3.9295521
C	0.2201513	-1.0204241	-0.5204506
C	1.8519680	0.4387073	0.6734354
C	-0.0887605	2.7708722	2.4529734
C	-2.4638768	2.2425198	1.9447371
C	0.0453289	2.4335288	-0.3646755
C	-0.3545212	2.9790354	-1.5236887
C	-1.8443383	0.8645821	-0.3913010
C	0.3235947	4.0573218	-2.2496226
C	1.5585410	4.6078742	-1.8557239
C	2.1409485	5.6322955	-2.5923219
C	1.5061989	6.1281920	-3.7363159
C	0.2845673	5.5908548	-4.1410940
C	-0.2996492	4.5631636	-3.4045315
H	-0.8037565	-1.5765560	5.6207368
H	0.2816019	-1.5579178	4.2200029
H	-2.5740770	-2.7490498	4.2358816
H	-1.0709622	-3.6829583	4.2793409
H	-1.4739819	-2.7384622	2.8388504
H	0.9501345	-1.8107140	-0.3311253
H	0.4446512	-0.5911962	-1.5005421
H	-0.7632141	-1.4893253	-0.5307606
H	2.0472752	1.1830563	1.4441919
H	2.2171993	0.8005937	-0.2898889
H	2.4242313	-0.4531087	0.9387777
H	-0.2309113	3.7455493	1.9807040
H	-0.3543345	2.8662858	3.5078283
H	0.9655997	2.5009212	2.4139962
H	-3.2265713	1.5022035	1.7050562
H	-2.5994488	3.1281180	1.3169399
H	-2.6314902	2.5535018	2.9783948
H	0.9340395	2.7716092	0.1418069
H	-1.2611055	2.6272104	-2.0056578
H	-2.2974434	0.0473766	0.1653212
H	-1.5541939	0.5203237	-1.3794696
H	-2.5535281	1.6822889	-0.4875433
H	2.0756513	4.2344728	-0.9769365
H	3.0940530	6.0458904	-2.2786169
H	1.9662723	6.9274692	-4.3084390
H	-0.2120073	5.9690451	-5.0286968
H	-1.2513655	4.1470650	-3.7245778

SEt_MN2_6.log
E = -1209.660247
H = -1209.216681
G = -1209.292067
NImag=0

C	-0.8290296	1.7121917	1.8312519
N	-0.7060547	1.3785845	0.2828074
C	0.0977477	-0.1096405	0.3272207
N	-0.2611796	-0.6108939	1.6145972
C	-0.7348333	0.2865781	2.3924884
S	-1.2255802	0.0072189	4.0487357
C	-0.7446156	-1.7628799	4.2701400
C	-1.0860815	-2.2027254	5.6911524
C	-0.3889441	-1.0271323	-0.7930062
C	1.6121827	0.0826118	0.1988811
C	0.3450576	2.5664806	2.3480056
C	-2.1310597	2.4483967	2.1752267
C	0.0285754	2.3986701	-0.4702278
C	-0.4299045	3.0649453	-1.5411493
C	-2.0767386	1.1475787	-0.2836582
C	0.3033898	4.0764204	-2.3086043
C	1.6067172	4.5048233	-1.9908891
C	2.2405922	5.4692081	-2.7650381
C	1.5893513	6.0267011	-3.8708010
C	0.2980771	5.6135740	-4.1975468
C	-0.3385961	4.6473520	-3.4221303
H	0.3244229	-1.8387315	4.0614426
H	-1.2829947	-2.3435973	3.5185726
H	-0.5448621	-1.6164627	6.4387399
H	-0.7997269	-3.2509055	5.8157646
H	-2.1572621	-2.1218793	5.8950324
H	0.2218755	-1.9317153	-0.7482051
H	-0.2396479	-0.5759470	-1.7775637
H	-1.4266605	-1.3363248	-0.6729420
H	2.0322167	0.7355988	0.9626147
H	1.8896091	0.4470917	-0.7923323
H	2.0644703	-0.9021295	0.3365572
H	0.2981678	3.5812865	1.9465543
H	0.2489229	2.6300697	3.4338837
H	1.3257057	2.1405942	2.1401926
H	-3.0305625	1.8531153	2.0208757
H	-2.2063397	3.3832817	1.6120631
H	-2.0989460	2.7137423	3.2344416
H	1.0188712	2.5667131	-0.0809990
H	-1.4308908	2.8716533	-1.9132247
H	-2.5766525	0.3772721	0.2992318
H	-1.9870793	0.8252439	-1.3167030
H	-2.6482475	2.0710121	-0.2416538
H	2.1324856	4.0898282	-1.1362402
H	3.2449185	5.7905687	-2.5082945
H	2.0889147	6.7797965	-4.4717318
H	-0.2131650	6.0422071	-5.0533459
H	-1.3451169	4.3287544	-3.6805868

OIm_1.log
E = -807.720927
H = -807.347493
G = -807.416641
NImag=0

C	1.8388288	1.2125124	0.1091842
N	0.8943915	0.0762060	0.0316651
C	1.5563345	-1.2326564	-0.0773695
C	3.0377482	-0.8380083	-0.0543069
N	3.1278372	0.5175151	0.0590985
O	3.9741536	-1.6202546	-0.1227028
C	4.4098520	1.1931332	0.1089725
C	1.6912936	2.1621151	-1.0966760
C	1.6877391	1.9840699	1.4346584
C	1.2970232	-2.1615774	1.1267639
C	1.2693669	-1.9528817	-1.4099108
C	-0.4620578	0.3050227	0.0310665
C	-1.4864950	-0.5800357	-0.0002172
C	-2.9044763	-0.2108259	-0.0134679
C	-3.3778287	1.0809285	-0.3242411
C	-4.7376201	1.3771168	-0.2996138
C	-5.6757436	0.3913712	0.0171913
C	-5.2294896	-0.8990134	0.3063209
C	-3.8683106	-1.1932299	0.2901710
H	5.1750072	0.4171563	0.0608208
H	4.5306460	1.7579982	1.0393762
H	4.5401134	1.8757831	-0.7375772
H	1.8188135	1.6089468	-2.0304629
H	2.4352391	2.9631251	-1.0549642
H	0.7050305	2.6332089	-1.1041991
H	1.8277106	1.3082902	2.2819133
H	0.6934620	2.4318862	1.5100223
H	2.4187340	2.7953995	1.5029979
H	0.2769361	-2.5493404	1.1313131
H	1.4653727	-1.6304277	2.0678654
H	1.9923436	-3.0031559	1.0751237
H	1.4667199	-1.2897850	-2.2570771
H	0.2321780	-2.2865303	-1.4728056
H	1.9275259	-2.8214839	-1.4905110
H	-0.7076534	1.3620618	0.0715242
H	-1.2861504	-1.6451842	0.0144625
H	-2.6770668	1.8599542	-0.6111482
H	-5.0691241	2.3827403	-0.5445552
H	-6.7362962	0.6239079	0.0278598
H	-5.9440511	-1.6812202	0.5479829
H	-3.5348564	-2.2010426	0.5256081

OIm_2.log
E = -807.721014
H = -807.347496
G = -807.416577
NImag=0

C	-1.6073793	-1.1392735	-0.0572510
N	-0.8997694	0.1538411	0.0289946
C	-1.8044416	1.3156914	0.1015970
C	-3.1818186	0.6526564	0.0477988
N	-3.0093561	-0.6950238	-0.0472469
O	-4.2496909	1.2471118	0.0826535
C	-4.1388596	-1.6023890	-0.1134108
C	-1.2987857	-1.8748962	-1.3757648
C	-1.3326367	-2.0275565	1.1728704
C	-1.6736376	2.0951020	1.4233712
C	-1.6622689	2.2641596	-1.1036769
C	0.4645422	0.3266880	0.0294459
C	1.4724259	-0.5788992	-0.0087685
C	2.8987939	-0.2411895	-0.0154313
C	3.4031689	1.0562651	-0.2426599
C	4.7708675	1.3130024	-0.2213039
C	5.6864205	0.2835661	0.0119853
C	5.2088926	-1.0106680	0.2218653
C	3.8398865	-1.2663119	0.2076858
H	-4.1376449	-2.1792275	-1.0441056
H	-5.0383557	-0.9862981	-0.0770926
H	-4.1470714	-2.2975179	0.7326846
H	-1.5789157	-1.2461988	-2.2247171
H	-1.8565738	-2.8140361	-1.4326450
H	-0.2371636	-2.1097406	-1.4589940
H	-1.6431526	-1.5073253	2.0825089

H	-0.2723594	-2.2664125	1.2605808
H	-1.8835196	-2.9699828	1.1009018
H	-1.7512645	1.4224962	2.2821170
H	-2.4744896	2.8360013	1.4877805
H	-0.7123283	2.6149733	1.4799512
H	-2.4654605	3.0046787	-1.0756651
H	-1.7304025	1.7121908	-2.0453071
H	-0.7031774	2.7905086	-1.0813820
H	0.7322954	1.3779836	0.0758260
H	1.2581470	-1.6414717	-0.0124805
H	2.7218037	1.8746048	-0.4570343
H	5.1259891	2.3243943	-0.4002576
H	6.7529312	0.4869423	0.0215842
H	5.9046489	-1.8263238	0.3993343
H	3.4824759	-2.2790091	0.3791780

OIm_ME_1.log
E = -847.425189
H = -847.008809
G = -847.080572
NImag=0

C	-1.5540341	-1.1283554	-0.0130932
N	-0.8468683	0.1820074	0.3214396
C	-1.7126300	1.3865238	0.0084687
C	-3.0103287	0.7286342	-0.4610546
N	-2.8618048	-0.6281516	-0.4372234
O	-3.9988233	1.3510692	-0.7940899
C	-3.9525658	-1.5104041	-0.8421256
C	-1.6883534	-2.0032718	1.2416586
C	-0.8411947	-1.8406407	-1.1721007
C	-1.1043130	2.2164129	-1.1300635
C	-1.9813486	2.2282468	1.2615880
C	0.3370582	0.3378734	0.8098287
C	1.4108856	-0.6461936	1.1561358
C	1.7072819	-0.5759547	2.6738258
C	2.6167882	-0.2749041	0.2797999
C	3.3065434	0.9333353	0.4624227
C	4.4028769	1.2476091	-0.3395876
C	4.8221814	0.3605363	-1.3326402
C	4.1425464	-0.8438923	-1.5195806
C	3.0411635	-1.1562671	-0.7230378
H	-4.2791926	-2.1468831	-0.0154063
H	-3.6665426	-2.1349054	-1.6927710
H	-4.7788501	-0.8641684	-1.1391797
H	-2.1775476	-1.4503761	2.0470543
H	-0.7250208	-2.3747537	1.5928618
H	-2.3016123	-2.8751829	1.0030137
H	-0.7428458	-1.1761261	-2.0334263
H	-1.4309321	-2.7094334	-1.4732679
H	0.1471516	-2.2015461	-0.8876268
H	-1.8334884	2.9757448	-1.4204280
H	-0.8851668	1.6017108	-2.0069390
H	-0.1868252	2.7199239	-0.8114183
H	-1.0889983	2.7719005	1.5854989
H	-2.3490268	1.6154975	2.0889133
H	-2.7535953	2.9585673	1.0108624
H	0.6110066	1.3776593	0.9821739
H	1.1076334	-1.6619988	0.9069682
H	0.8419442	-0.8916718	3.2635942
H	1.9872637	0.4324678	2.9882907
H	2.5437689	-1.2419823	2.8973672
H	3.0102781	1.6292555	1.2430458
H	4.9331045	2.1815478	-0.1836187
H	5.6777766	0.6046588	-1.9538563
H	4.4707553	-1.5432065	-2.2818326
H	2.5295896	-2.1040906	-0.8677111

OIm_MO_1.log
E = -847.381511
H = -846.965225
G = -847.038547
NImag=0

C	1.4202179	1.3788206	0.1986711
N	0.5693664	0.2203039	-0.0822042
C	1.2911220	-1.0543435	-0.0554619
C	2.7155839	-0.5409747	-0.0742158
N	2.7951436	0.7523035	0.0945323
O	3.6431505	-1.4368092	-0.2298691
C	3.9880082	1.6094373	0.1843837
C	1.2871158	2.4670164	-0.8770785
C	1.2207171	1.9455621	1.6149877
C	1.1082115	-1.8898838	1.2412121
C	1.0242819	-1.9058765	-1.3153792
C	-0.8221195	0.3703964	-0.0589085
C	-1.7808468	-0.5717514	0.0237358
C	5.0781857	-1.2306975	-0.3722503
C	-3.2232396	-0.2933480	-0.0421063
C	-3.7566134	0.8635636	-0.6395781
C	-5.1294332	1.0959756	-0.6452966
C	-6.0048264	0.1707841	-0.0718892
C	-5.4934901	-0.9937233	0.5024893
C	-4.1196890	-1.2235070	0.5140107
H	4.7998627	1.0891745	0.6844542
H	3.7354654	2.4838681	0.7812485
H	4.3021903	1.9385457	-0.8084743
H	1.4568836	2.0484630	-1.8717347
H	0.2806437	2.8888343	-0.8511404
H	1.9809730	3.2933899	-0.7028466
H	1.8499168	2.8183107	1.8093518
H	1.4161226	1.1851959	2.3746874
H	0.1814545	2.2656837	1.7186851
H	1.3210122	-1.2931560	2.1321061
H	1.7729606	-2.7570175	1.2291024
H	0.0824887	-2.2488497	1.3168468
H	-0.0246061	-2.1990161	-1.3505019
H	1.6368867	-2.8095404	-1.3018332
H	1.2432320	-1.3352397	-2.2214276
H	-1.1269986	1.4075156	-0.1519701
H	-1.5209606	-1.6147045	0.1706389
H	5.4470910	-2.1862298	-0.7391318
H	5.5198490	-1.0128200	0.6006077
H	5.2858119	-0.4460459	-1.0990318
H	-3.0981996	1.5734716	-1.1323425
H	-5.5196742	1.9937646	-1.1152603
H	-7.0751857	0.3500252	-0.0857772
H	-6.1649662	-1.7249404	0.9421118
H	-3.7308699	-2.1304196	0.9703196

OIm_MO_2.log
E = -847.381789
H = -846.965488
G = -847.038276
NImag=0

C	1.2076303	1.2346994	-0.0919516
N	0.5706334	-0.0794185	-0.0986018
C	1.4991855	-1.1837507	0.1758445
C	2.8172332	-0.4561881	0.0395861
N	2.6747763	0.8345546	-0.0956862
O	3.8842624	-1.1969740	0.0853687
C	3.7041195	1.8760441	-0.2453233
C	0.9072047	2.0201450	-1.3765021
C	0.9371521	2.0468955	1.1883089
C	1.3794293	-1.7679475	1.6056645
C	1.4065867	-2.3077080	-0.8787463
C	-0.8093652	-0.3028085	-0.0505887
C	-1.8311857	0.5758789	-0.0430854
C	5.2727055	-0.7820086	-0.0630521
C	-3.2521351	0.1949430	-0.0534650
C	-3.7103825	-1.0603145	-0.4943788
C	-5.0647595	-1.3800456	-0.4555727
C	-5.9978237	-0.4490513	0.0070500
C	-5.5622828	0.8087481	0.4255466
C	-4.2063326	1.1265182	0.3929016
H	4.5664163	1.6630742	0.3805764
H	3.2800149	2.8228031	0.0822194
H	4.0059821	1.9696818	-1.2906443
H	1.2239113	1.4494277	-2.2526784
H	1.3956926	2.9976487	-1.3785181
H	-0.1667139	2.1871246	-1.4592046
H	1.2911130	1.5090864	2.0711144
H	-0.1355784	2.2016432	1.3021128
H	1.4078406	3.0329025	1.1577016
H	1.4824654	-0.9904834	2.3671368
H	2.1367621	-2.5367892	1.7764937
H	0.3944985	-2.2256386	1.7228362
H	1.4911195	-1.9038133	-1.8906040
H	0.4447754	-2.8175406	-0.7938215
H	2.1920182	-3.0491927	-0.7179565
H	-1.0433488	-1.3617952	-0.0414283
H	-1.6482698	1.6448719	-0.0056059
H	5.8167718	-1.7172037	-0.1772226
H	5.6048154	-0.2690076	0.8399650
H	5.3998721	-0.1654240	-0.9521793
H	-3.0090774	-1.7844598	-0.8994526
H	-5.3960146	-2.3537252	-0.8039543
H	-7.0539857	-0.6980691	0.0284169
H	-6.2787723	1.5443121	0.7782906
H	-3.8771938	2.1070207	0.7280877

OIm_MN_1.log
E = -847.387755
H = -846.970117
G = -847.038765
NImag=0

C	-1.6681444	1.3154402	-0.0374569
N	-0.8536880	0.0241473	0.4504472

C	-1.7098578	-1.2117590	-0.1484456
N	-3.0419048	-0.6282963	-0.1113410
C	-3.0813599	0.7389177	-0.1888294
O	-4.0703379	1.4292385	-0.3333065
C	-1.1749966	1.8818839	-1.3787834
C	-1.6572460	2.4582108	0.9839577
C	-4.2555641	-1.4243837	-0.2890395
C	0.5360429	0.0205020	-0.0391229
C	-0.9090535	-0.0460897	1.9550173
C	1.6353484	-0.0411165	0.7271634
C	-1.5813561	-2.4606252	0.7272114
C	-1.2734968	-1.5780458	-1.5750697
C	3.0209860	-0.0337087	0.2499036
C	3.3785573	0.0259071	-1.1111856
C	4.7157578	0.0299380	-1.4884380
C	5.7229051	-0.0252978	-0.5184916
C	5.3846092	-0.0850385	0.8331444
C	4.0446492	-0.0895353	1.2129758
H	-1.9097152	2.6320479	-1.6802090
H	-0.2078101	2.3766998	-1.2672445
H	-1.1144109	1.1549857	-2.1870964
H	-2.1319349	2.2100563	1.9325869
H	-2.2432874	3.2684452	0.5465265
H	-0.6426941	2.8272068	1.1582491
H	-4.2390208	-1.9762801	-1.2340431
H	-5.0901370	-0.7232505	-0.3066224
H	-4.3887433	-2.1249381	0.5383709
H	0.5937998	0.0903595	-1.1123436
H	-1.9504104	-0.0687655	2.2669646
H	-0.4149013	0.8273296	2.3684506
H	-0.4004211	-0.9437201	2.2921647
H	1.5439468	-0.0989593	1.8070170
H	-2.0504556	-2.3555309	1.7051968
H	-0.5303268	-2.7361598	0.8478262
H	-2.0704051	-3.2910636	0.2131362
H	-1.3141237	-0.7431134	-2.2720593
H	-1.9741778	-2.3327449	-1.9409699
H	-0.2775664	-2.0248193	-1.5931138
H	2.6162722	0.0692100	-1.8832756
H	4.9780743	0.0763327	-2.5404747
H	6.7656480	-0.0215887	-0.8192937
H	6.1609371	-0.1278570	1.5899556
H	3.7856736	-0.1357865	2.2675372

OIm_MN_2.log
E = -847.388867
H = -846.971752
G = -847.040698
NImag=0

C	-1.7891368	-1.0730855	0.7789283
N	-1.0030489	-0.6176765	-0.5346872
C	-1.8120989	0.6770004	-1.0503208
N	-2.2809221	1.1910407	0.2291201
C	-2.4039449	0.2593105	1.2272939
O	-2.9067570	0.4293952	2.3196302
C	-0.8544082	-1.6409448	1.8510156
C	-2.9013614	-2.1016841	0.5159263
C	-2.8501951	2.5293726	0.3779412
C	0.3511379	-0.1665778	-0.1331878
C	-0.9423837	-1.6986009	-1.5672752
C	1.5110025	-0.6568130	-0.5858891
C	-2.9733070	0.3106046	-1.9875849
C	-0.8815727	1.6503555	-1.7788599
C	2.8475455	-0.2095584	-0.1789075
C	3.0751910	0.7389499	0.8367844
C	4.3701487	1.1203793	1.1672207
C	5.4630221	0.5643998	0.4936079
C	5.2531594	-0.3793189	-0.5116590
C	3.9561037	-0.7642183	-0.8428348
H	-1.4786778	-1.8857297	2.7127954
H	-0.3641283	-2.5557370	1.5078619
H	-0.0953248	-0.9385263	2.1910425
H	-3.6115117	-1.8204373	-0.2603603
H	-3.4656365	-2.1812843	1.4481359
H	-2.4917271	-3.0886389	0.2889402
H	-2.0998497	3.2980731	0.1793503
H	-3.1891268	2.6127234	1.4107031
H	-3.7069478	2.6772984	-0.2868778
H	0.2974733	0.6336275	0.5904427
H	-1.9453571	-2.0067657	-1.8393844
H	-0.4024365	-2.5470472	-1.1494668
H	-0.4192019	-1.3248055	-2.4464902
H	1.5158428	-1.4533177	-1.3236276
H	-3.6692945	-0.4109778	-1.5635972
H	-2.6228774	-0.0438047	-2.9591422
H	-3.5389104	1.2277313	-2.1705946
H	-0.1281928	2.0943718	-1.1300494
H	-1.4883503	2.4560047	-2.1995673
H	-0.3790439	1.1561981	-2.6136802
H	2.2448098	1.1775372	1.3822208
H	4.5316436	1.8496633	1.9545122
H	6.4721338	0.8647797	0.7567288
H	6.0966416	-0.8168173	-1.0356096
H	3.7978520	-1.5008915	-1.6261646

SIm_1.log
E = -1130.677144
H = -1130.305565
G = -1130.375370
NImag=0

C	-1.2770494	1.2827978	-0.0755997
N	-0.6349424	-0.0322032	0.0226608
C	-1.5982722	-1.1544485	0.1089870
C	-2.9390224	-0.4236092	0.0433852
N	-2.7050601	0.8902873	-0.0753991
S	-4.4384109	-1.1450501	0.1077662
C	-3.7818662	1.8668995	-0.1531547
C	-0.9901681	2.1675039	1.1544468
C	-0.9298271	1.9938051	-1.3979348
C	-1.4660943	-2.1217619	-1.0838052
C	-1.4710954	-1.9203178	1.4400998
C	0.7212686	-0.2651866	0.0261861
C	1.7670134	0.5964745	0.0043406
C	3.1754732	0.1879595	-0.0138565
C	3.6116084	-1.1082996	-0.3565714
C	4.9624339	-1.4437004	-0.3366011
C	5.9271823	-0.4931801	0.0064023
C	5.5176254	0.8018976	0.3267794
C	4.1652997	1.1349705	0.3158341
H	-4.3606161	1.8717108	0.7755365
H	-4.4616317	1.6011627	-0.9667124
H	-3.3665049	2.8582676	-0.3305189
H	-1.3028278	1.6506363	2.0649055
H	-1.5278780	3.1174906	1.0858403
H	0.0738844	2.3916008	1.2365541
H	-1.4253113	2.9662821	-1.4649960
H	-1.2436820	1.3772208	-2.2435431
H	0.1452571	2.1597756	-1.4759296
H	-2.2637095	-2.8661768	-1.0395450
H	-0.5030853	-2.6405563	-1.0570823
H	-1.5434743	-1.5831076	-2.0320637
H	-1.5604112	-1.2398129	2.2911204
H	-0.5019827	-2.4247215	1.5018148
H	-2.2618908	-2.6701763	1.5092758
H	0.9458129	-1.3265988	0.0593960
H	1.6031574	1.6679365	0.0297228
H	2.8895294	-1.8587523	-0.6652568
H	5.2656471	-2.4518048	-0.6062057
H	6.9806301	-0.7560248	0.0130513
H	6.2536980	1.5570345	0.5889192
H	3.8605340	2.1458355	0.5762162

SIm_2.log
E = -1130.676354
H = -1130.304833
G = -1130.375142
NImag=0

C	1.4956406	1.4087888	0.1221395
N	0.6359083	0.2185540	0.0485647
C	1.3915785	-1.0481266	-0.0496244
C	2.8396815	-0.5473078	-0.0379106
N	2.8336096	0.7891572	0.0691980
S	4.1983829	-1.5037725	-0.1366111
C	4.0604620	1.5714609	0.1097852
C	1.2953350	2.3423733	-1.0887545
C	1.3057616	2.1688929	1.4490477
C	1.1657681	-1.9683439	1.1689466
C	1.1133812	-1.7927246	-1.3711962
C	-0.7343242	0.3610658	0.0390442
C	-1.7074630	-0.5795198	0.0158127
C	-3.1436620	-0.2872965	-0.0147756
C	-3.6829354	0.9678565	-0.3643459
C	-5.0570008	1.1903987	-0.3567623
C	-5.9425205	0.1639145	-0.0190484
C	-5.4297868	-1.0921000	0.3086207
C	-4.0545902	-1.3122402	0.3096566
H	4.6397084	1.4113901	-0.8040939
H	4.6785292	1.2542076	0.9542743
H	3.8180503	2.6289436	0.2096875
H	1.4439877	1.7898274	-2.0194540
H	1.9939005	3.1835504	-1.0599617
H	0.2846949	2.7588322	-1.0903368
H	1.9869064	3.0218437	1.5227273
H	1.4814954	1.4996995	2.2944832
H	0.2861717	2.5566618	1.5211572
H	1.8461328	-2.8199169	1.1042830
H	0.1395640	-2.3374966	1.2048765

```
H        1.3697637       -1.4318777        2.0996711
H        1.3081928       -1.1407793       -2.2271511
H        0.0761199       -2.1273263       -1.4249472
H        1.7729448       -2.6599948       -1.4404297
H       -1.0418933        1.4022034        0.0625170
H       -1.4516292       -1.6319130        0.0486901
H       -3.0229520        1.7748616       -0.6700037
H       -5.4407280        2.1691414       -0.6322362
H       -7.0141538        0.3385863       -0.0222148
H       -6.1030344       -1.9050159        0.5666985
H       -3.6687643       -2.2936834        0.5745633
```

SIm_ME_1.log

```
E =     -1170.378481
H =     -1169.964281
G =     -1170.036986
NImag=0
C        1.2325960        1.2879867       -0.0689641
N        0.6054799       -0.0033417        0.4095707
C        1.5667127       -1.1833299        0.2760793
C        2.8252950       -0.5022944       -0.2539146
N        2.5854203        0.8165161       -0.4031869
S        4.2368544       -1.2887813       -0.5844471
C        3.6120337        1.7245671       -0.9163753
C        1.2860659        2.3163062        1.0712022
C        0.5191845        1.8038800       -1.3279191
C        1.0115945       -2.1934990       -0.7387138
C        1.8185434       -1.8234583        1.6479974
C       -0.5827402       -0.1750023        0.8819416
C       -1.7275841        0.7717360        1.0693321
C       -2.0797595        0.8658417        2.5736823
C       -2.8722124        0.2184620        0.2067979
C       -3.4900673       -1.0044695        0.5098119
C       -4.5314592       -1.4838813       -0.2838049
C       -4.9668570       -0.7493777       -1.3882051
C       -4.3584295        0.4683694       -1.6955492
C       -3.3114849        0.9453321       -0.9072803
H        3.9277881        1.4023896       -1.9118781
H        4.4853138        1.7017815       -0.2600186
H        3.2204420        2.7394600       -0.9676682
H        1.7792143        1.8928508        1.9491088
H        0.2925344        2.6683885        1.3515696
H        1.8571210        3.1886489        0.7465547
H        0.4700410        1.0241388       -2.0910967
H        1.0766290        2.6494226       -1.7366503
H       -0.4906619        2.1521481       -1.1105897
H        0.8058364       -1.7226890       -1.7032503
H        0.0957954       -2.6638383       -0.3684446
H        1.7629547       -2.9701605       -0.8916185
H        2.1527913       -1.0842347        2.3803771
H        2.6078701       -2.5680710        1.5316982
H        0.9248739       -2.3287033        2.0262334
H       -0.7975410       -1.1997604        1.1793890
H       -1.4783456        1.7680616        0.7064236
H       -2.9666773        1.4935195        2.6860385
H       -1.2616924        1.3109406        3.1472734
H       -2.3048949       -0.1140418        3.0021713
H       -3.1809023       -1.5835514        1.3762497
H       -5.0063687       -2.4273968       -0.0349154
H       -5.7798958       -1.1220671       -2.0026820
H       -4.7001344        1.0500449       -2.5456374
H       -2.8559173        1.9022739       -1.1476073
```

SIm_MS_1.log

```
E =     -1170.356436
H =     -1169.943725
G =     -1170.018214
NImag=0
C        0.8850175        1.4797320       -0.1212163
N        0.3646615        0.1172817       -0.1439033
C        1.3870570       -0.9233324        0.0922548
C        2.6518312       -0.0768845       -0.0004915
N        2.3615585        1.1914261       -0.1245588
S        4.3283963       -0.5094226        0.1026977
C        3.3558851        2.2670786       -0.1985954
C        0.5159307        2.2490663       -1.3997076
C        0.5615812        2.2647002        1.1651461
C        1.2623851       -1.5545114        1.5017303
C        1.3323311       -1.9974290       -1.0166355
C       -0.9932431       -0.2159574       -0.0778515
C       -2.0785347        0.5822201       -0.0431590
C        4.4757306       -2.3338056        0.1030607
C       -3.4672043        0.0970051       -0.0376370
C       -3.8392209       -1.1853992       -0.4814606
C       -5.1663768       -1.6025320       -0.4285632
C       -6.1584685       -0.7442725        0.0516106
C       -5.8096376        0.5390177        0.4737205
C       -4.4806766        0.9541882        0.4269270
H        4.0466278        2.0725880       -1.0234024
H        3.9188786        2.3242435        0.7373628
H        2.8492517        3.2126034       -0.3722115
H        0.8778210        3.2798901       -1.3759839
H       -0.5690289        2.2823379       -1.4998453
H        0.9178200        1.7399197       -2.2785285
H        0.8899738        1.7120172        2.0486628
H       -0.5130782        2.4235746        1.2484419
H        1.0414373        3.2469688        1.1612443
H        0.2809746       -2.0257172        1.5920858
H        1.3451292       -0.7964071        2.2840292
H        2.0139723       -2.3261579        1.6794944
H        0.3318229       -2.4330299       -1.0471565
H        2.0285718       -2.8150336       -0.8294088
H        1.5365603       -1.5611003       -1.9973616
H       -1.1498883       -1.2887483       -0.0787499
H       -1.9763162        1.6612202        0.0055683
H        5.5378310       -2.4894548        0.3043392
H        4.2319016       -2.7485388       -0.8733054
H        3.8944788       -2.7946777        0.8988745
H       -3.0931322       -1.8548102       -0.9006398
H       -5.4308023       -2.5954135       -0.7798537
H       -7.1935792       -1.0689781        0.0835797
H       -6.5728349        1.2188993        0.8399152
H       -4.2192627        1.9541304        0.7643165
```

SIm_MS_2.log

```
E =     -1170.356321
H =     -1169.943578
G =     -1170.018082
NImag=0
C       -1.1159356        1.6643023        0.1327556
N       -0.3742548        0.4222968       -0.0882688
C       -1.2032799       -0.7918528       -0.0091165
C       -2.5930283       -0.1605462       -0.0293872
N       -2.5209484        1.1409908        0.0762784
S       -4.1700191       -0.8772712       -0.0854394
C       -3.6832060        2.0322974        0.1574034
C       -0.8615255        2.2997818        1.5111671
C       -0.9017377        2.6709044       -1.0092870
C       -0.9539309       -1.6924499       -1.2409585
C       -1.0331014       -1.5524256        1.3340514
C        1.0251072        0.4546072       -0.0667612
C        1.9153214       -0.5522973        0.0344307
C       -3.9874302       -2.6740101       -0.3828429
C        3.3743303       -0.3748260       -0.0295710
C        3.9894682        0.7374444       -0.6330256
C        5.3753443        0.8715317       -0.6376306
C        6.1821030       -0.1098751       -0.0570525
C        5.5884266       -1.2312095        0.5238156
C        4.2016904       -1.3623704        0.5343778
H       -3.3423071        3.0591361        0.2597182
H       -4.2950907        1.7689774        1.0245417
H       -4.2855655        1.9405343       -0.7507332
H       -1.4664068        3.1978017        1.6631449
H        0.1879803        2.5950198        1.5790547
H       -1.0672744        1.5899639        2.3156713
H       -1.1333983        2.2133902       -1.9735043
H        0.1418816        2.9914619       -1.0232509
H       -1.5070979        3.5710522       -0.8765572
H        0.1201170       -1.7891572       -1.3983804
H       -1.3783525       -1.2491315       -2.1455046
H       -1.3604377       -2.6944974       -1.1024065
H       -0.0014989       -1.8853633        1.4425297
H       -1.6741359       -2.4346475        1.3842508
H       -1.2659408       -0.9042939        2.1823505
H        1.4092131        1.4632965       -0.1769231
H        1.5881196       -1.5737409        0.1970795
H       -5.0177754       -3.0325612       -0.3325525
H       -3.4039815       -3.1621137        0.3954031
H       -3.5863793       -2.8685717       -1.3753620
H        3.3845853        1.4902934       -1.1308655
H        5.8289215        1.7366135       -1.1119920
H        7.2624770       -0.0071332       -0.0699535
H        6.2054059       -2.0054770        0.9695232
H        3.7489407       -2.2365390        0.9958063
```

SIm_MS_3.log

```
E =     -1170.356374
H =     -1169.943529
G =     -1170.018130
NImag=0
C        0.8821247        1.4858183        0.0695457
N        0.3634148        0.1335684       -0.1041542
C        1.3913699       -0.9255011       -0.0499084
C        2.6525563       -0.0707090       -0.0035687
N        2.3586649        1.2001968        0.0759091
S        4.3302389       -0.5106431        0.0365037
```

C	3.3502518	2.2759538	0.1834984
C	0.5639142	2.3891851	-1.1350525
C	0.5010162	2.1251358	1.4164734
C	1.2829682	-1.7741653	1.2426572
C	1.3288246	-1.8087906	-1.3156955
C	-0.9925782	-0.2105385	-0.0661830
C	-2.0827921	0.5812415	-0.0361726
C	4.4818502	-2.3067130	-0.2803640
C	-3.4688914	0.0900177	-0.0002140
C	-3.8192535	-1.2073055	0.4170837
C	-5.1465123	-1.6269968	0.4074141
C	-6.1599777	-0.7573016	-0.0027062
C	-5.8319193	0.5397521	-0.3981933
C	-4.5029976	0.9578300	-0.3940854
H	4.0394342	2.2294550	-0.6641117
H	3.9153391	2.1714232	1.1139427
H	2.8412238	3.2360606	0.1797174
H	0.9693213	3.3959698	-1.0079811
H	-0.5157471	2.4833698	-1.2480858
H	0.9598477	1.9501483	-2.0536462
H	-0.5830588	2.2143165	1.4871149
H	0.9262821	3.1265892	1.5201189
H	0.8417422	1.5030688	2.2476788
H	0.3142461	-2.2779017	1.2613045
H	1.3549956	-1.1460515	2.1337320
H	2.0516457	-2.5473043	1.2942849
H	0.3192139	-2.2120199	-1.4169131
H	2.0101195	-2.6580644	-1.2590483
H	1.5450264	-1.2260795	-2.2145344
H	-1.1427820	-1.2839370	-0.0895372
H	-1.9873340	1.6615284	-0.0694904
H	5.5492889	-2.4868971	-0.1357960
H	4.2138097	-2.5475748	-1.3072646
H	3.9229300	-2.9026185	0.4380659
H	-3.0546856	-1.8887601	0.7799472
H	-5.3936328	-2.6318331	0.7366347
H	-7.1947425	-1.0846467	-0.0018102
H	-6.6111896	1.2281698	-0.7106244
H	-4.2580346	1.9686929	-0.7105598

SIm_MN_1.log
E = -1170.341754
H = -1169.926623
G = -1169.996885
NImag=0

C	1.6620171	0.2731535	-1.2078344
N	0.7811465	-0.8668211	-0.4868307
C	1.5634880	-1.1606054	0.8696053
N	2.1281685	0.1712956	1.1086052
C	2.3134653	0.9399619	0.0059812
S	3.1251535	2.3729620	-0.0686630
C	0.7720522	1.2228649	-2.0174622
C	2.7301445	-0.3000839	-2.1565522
C	2.7408078	0.5178534	2.3924114
C	-0.5321360	-0.2752149	-0.1301750
C	0.6266759	-2.0928186	-1.3308207
C	-1.7302681	-0.7344880	-0.5098694
C	2.6654792	-2.2208680	0.7178501
C	0.5928374	-1.6155555	1.9663648
C	-3.0272951	-0.1477982	-0.1567673
C	-4.1874060	-0.8111876	-0.5946733
C	-5.4524795	-0.3147527	-0.2896300
C	-5.5777145	0.8565598	0.4569863
C	-4.4325099	1.5300706	0.8956951
C	-3.1693671	1.0357811	0.5930068
H	0.2252201	0.6801388	-2.7933592
H	1.4319091	1.9444685	-2.5014841
H	0.0656435	1.7852779	-1.4099201
H	3.4035736	-1.0231064	-1.6994233
H	3.3407540	0.5485116	-2.4715541
H	2.2797321	-0.7370466	-3.0506448
H	3.8188019	0.3268282	2.3756824
H	2.2748072	-0.0566922	3.1914426
H	2.5902746	1.5829536	2.5721283
H	-0.4091721	0.5985656	0.4930057
H	0.1074775	-1.8202858	-2.2483235
H	0.0440360	-2.8330826	-0.7838728
H	1.6013689	-2.4996031	-1.5741034
H	-1.8018451	-1.6197421	-1.1340011
H	3.3812869	-1.9987009	-0.0705984
H	2.2520235	-3.2206598	0.5683995
H	3.2211775	-2.2419750	1.6588814
H	-0.0182775	-2.4491727	1.6125562
H	-0.0693260	-0.8219907	2.3093824
H	1.1660937	-1.9833049	2.8198857
H	-4.0946754	-1.7238822	-1.1776031
H	-6.3366889	-0.8402622	-0.6351654
H	-6.5614888	1.2477665	0.6955703
H	-4.5283360	2.4443808	1.4723923
H	-2.2963846	1.5815966	0.9383827

SIm_MN_2.log
E = -1170.340504
H = -1169.925128
G = -1169.995917
NImag=0

C	1.4991459	-1.1415858	0.0994418
N	0.6082506	0.1581792	0.4714833
C	1.4029265	1.3612791	-0.2184043
N	2.7686179	0.8427265	-0.1116586
C	2.8811390	-0.5078696	-0.0685653
S	4.2730742	-1.3838515	-0.1874845
C	1.0351833	-1.8245076	-1.2001253
C	1.4551193	-2.1977780	1.2104430
C	3.9301807	1.6956731	-0.3720015
C	-0.7733659	0.0521268	-0.0276066
C	0.6451804	0.3525120	1.9667532
C	-1.8826217	0.1017483	0.7256931
C	1.1893861	2.6763852	0.5394708
C	0.9946031	1.5861216	-1.6827741
C	-3.2590846	-0.0250531	0.2392271
C	-3.5890019	-0.3361617	-1.0942090
C	-4.9183598	-0.4345017	-1.4862968
C	-5.9446826	-0.2266793	-0.5581056
C	-5.6335689	0.0768666	0.7671419
C	-4.3015978	0.1740983	1.1623275
H	0.0678757	-2.3124202	-1.0623848
H	0.9891849	-1.1696464	-2.0683569
H	1.7786469	-2.5928557	-1.4203389
H	0.4278339	-2.5254361	1.3952004
H	1.9221736	-1.8788507	2.1412877
H	2.0243995	-3.0567076	0.8534016
H	4.2298503	1.6379972	-1.4235713
H	4.7631269	1.3404270	0.2357176
H	3.6999505	2.7266415	-0.1081613
H	-0.8149276	-0.0890875	-1.0945235
H	0.1814956	-0.5033798	2.4456473
H	0.0971780	1.2523702	2.2273899
H	1.6817064	0.4401085	2.2836642
H	-1.8069523	0.2518634	1.7979122
H	1.5865850	3.4989316	-0.0580049
H	1.6794392	2.7006143	1.5125154
H	0.1195258	2.8678783	0.6587894
H	-0.0160643	1.9918415	-1.7599878
H	1.0822500	0.6975971	-2.3039774
H	1.6787859	2.3329048	-2.0935652
H	-2.8104640	-0.5147140	-1.8297821
H	-5.1593208	-0.6782914	-2.5159531
H	-6.9812788	-0.3064987	-0.8693636
H	-6.4251152	0.2352563	1.4921845
H	-4.0639248	0.4096653	2.1964047

10. References

[S1] a) E. Follet, G. Berionni, P. Mayer, H. Mayr, *J. Org. Chem.* **2015**, *80*, 8643–8656. b) H. Mayr, T. Bug, M. F. Gotta, N. Hering, B. Irrgang, B. Janker, B. Kempf, R. Loos, A. R. Ofial, G. Remennikov, H. Schimmel, *J. Am. Chem. Soc.* **2001**, *123*, 9500–9512.

[S2] a) A. C. Closs, E. Fuks, M. Bechtel, O. Trapp, *Chem. Eur. J.* **2020**, *26*, 10702–10706. b) C. Francavilla, E. D. Turtle, B. Kim; D. J. R. O'Mahony, T. P. Shiau, E. Low, N. J. Alvarez, C. E. Celeri, L. D'Lima, L. C. Friedman, F. S. Ruado, P. Xu, M. E. Zuck, M. B. Anderson, R. Najafi, R. K. Jain, *Bioorg. Med. Chem. Lett.* **2011**, *21*, 3029–3033. c) J. D. Christian, *J. Org. Chem.* **1957**, *22*, 396–399.

[S3] K. A. Ahrendt, C. J. Borths, D. W. C., MacMillan, *J. Am. Chem. Soc.* **2000**, *122*, 4243–4244.

[S4] X. Liang, J. Fan, F. Shi, W. Su, *Tetrahedron Lett*. **2010**, *51*, 2505–2507.

[S5] Product **2d** was previously synthesized via a different route and reported to be a yellow oil: F. Asinger, K. Hentschel, A. Saus, *Monatsh. Chem*. **1976**, *107*, 35–41.

[S6] G. R. Fulmer, A. J. M. Miller, N. H. Sherden, H. E. Gottlieb, A. Nudelman, B. M. Stolz, J. E. Bercaw, K. I. Goldberg, *Organometallics*, **2010**, *29*, 2176–2179.

[S7] D. S. Timofeeva, R. J. Mayer, P. Mayer, A. R. Ofial, H. Mayr, *Chem. Eur. J.* **2018**, 24, 5901–5910.

[S8] In analogy to: D. F. Bushey, F. C. Hoover, *J. Org. Chem.* **1980**, *45*, 4198–4206.

[S9] M. J. Hensinger, A. C. Closs, O. Trapp, A. R. Ofial, *Chem. Commun*. **2023**, *59*, 8091–8094.

[S10] Enamines were synthetized in analogy to: S. Lakhdar, B. Maji, H. Mayr, *Angew. Chem. Int. Ed.* **2012**, *51*, 5739–5742.

[S11] B. Kempf, N. Hampel, A. R. Ofial, H. Mayr, *Chem. Eur. J.* **2003**, *9*, 2209–2218.

[S12] H. Erdmann, F. An, P. Mayer, A. R. Ofial, S. Lakhdar, H. Mayr, *J. Am. Chem. Soc.* **2014**, *136*, 14263–14269.

[S13] P. G. Cozzi, F. Benfatti, L. Zoli, *Angew. Chem. Int. Ed.* **2009**, *48*, 1313–1316.

[S14] J. Ruiz Aranzaes, M.-C. Daniel, D. Astruc, *Can. J. Chem*. **2006**, *84*, 288–299.

[S15] D. Kadish, A. D. Mood, M. Tavakoli, E. S. Gutman, P. Baldi, D. L. Van Vranken, *J. Org. Chem.* **2021**, *86*, 3721–3729.

[S16] C. Lindner, R. Tandon, B. Maryasin, E. Larionov, H. Zipse, *Beilstein J. Org. Chem.* **2012**, *8*, 1406–1442.

[S17] Schrödinger Release 2021-4: MacroModel, Schrödinger, LLC, New York, NY, 2021.

[S18] Gaussian 09, Revision D.01, M. J. Frisch, G. W. Trucks, H. B. Schlegel, G. E. Scuseria, M. A. Robb, J. R. Cheeseman, G. Scalmani, V. Barone, G. A. Petersson, H. Nakatsuji, X. Li, M. Caricato, A. Marenich, J. Bloino, B. G. Janesko, R. Gomperts, B. Mennucci, H. P. Hratchian, J. V. Ortiz, A. F. Izmaylov, J. L. Sonnenberg, D. Williams-Young, F. Ding, F. Lipparini, F. Egidi, J. Goings, B. Peng, A. Petrone, T. Henderson, D. Ranasinghe, V. G. Zakrzewski, J. Gao, N. Rega, G. Zheng, W. Liang, M. Hada, M. Ehara, K. Toyota, R. Fukuda, J. Hasegawa, M. Ishida, T. Nakajima, Y. Honda, O. Kitao, H. Nakai, T. Vreven, K. Throssell, J. A. Montgomery, Jr., J. E. Peralta, F. Ogliaro, M. Bearpark, J. J. Heyd, E. Brothers, K. N. Kudin, V. N. Staroverov, T. Keith, R. Kobayashi, J. Normand, K. Raghavachari, A. Rendell, J. C. Burant, S. S. Iyengar, J. Tomasi, M. Cossi, J. M. Millam, M. Klene, C. Adamo, R. Cammi, J. W. Ochterski, R. L. Martin, K. Morokuma, O. Farkas, J. B. Foresman, and D. J. Fox, Gaussian, Inc., Wallingford CT, 2016.

[S19] A. D. Becke, *J. Chem. Phys.* **1993**, *98*, 5648–5652.

[S20] R. Ditchfield, W. J. Hehre, J. A. Pople, *J. Chem. Phys.* **1971**, *54*, 724–728.

[S21] R. Krishnan, J. S. Binkley, R. Seeger, J. A. Pople, *J. Chem. Phys.* **1980**, *72*, 650–654.

[S22] A. V. Marenich, C. J. Cramer, D. G. Truhlar, *J. Phys. Chem. B* **2009**, *113*, 6378–6396.

[S23] C. Adamo, V. Barone, *J. Chem. Phys.* **1999**, *110*, 6158–6170.

[S24] F. Weigend, R. Ahlrichs, *Phys. Chem. Chem. Phys.* **2005**, *7*, 3297–3305.

[S25] S. Grimme, *J. Comput. Chem.* **2006**, *27*, 1787–1799.

[S26] S. Grimme, J. Antony, S. Ehrlich, H. Krieg, *J. Chem. Phys.* **2010**, *132*, 154104.

[S27] G. Knizia, *J. Chem. Theory Comput.* **2013**, *9*, 4834–4843.
